# Phosphorylation tunes strain-specific protein condensation during rotavirus replication organelle assembly

**DOI:** 10.1038/s44318-026-00814-z

**Published:** 2026-05-26

**Authors:** Julia Acker, Xinyu Wang, Alonso J Pardal, Daniel Desirò, Tanushree Agarwal, Alice Colyer, Aidan Tollervey, Rob Scrutton, Cyril Haller, Lee Sherry, Kadi L Saar, Ksenia Fominykh, Margaret L L Y Johncock, Sai Hou Chong, Rosie Murray, Jamie Terry, Jeremy D Schmit, Antonio N Calabrese, Tuomas P J Knowles, Alexander Borodavka

**Affiliations:** 1 Department of Biochemistry and Department of Chemical Engineering and Biotechnology, University of Cambridge, Cambridge, UK; 2 Department of Chemistry, University of Cambridge, Cambridge, UK; 3 Astbury Centre for Structural Molecular Biology, School of Molecular and Cellular Biology, Faculty of Biological Sciences, University of Leeds, Leeds, UK; 4 The Sir William Dunn School of Pathology, University of Oxford, Oxford, UK; 5 Department of Physics, Kansas State University, Manhattan, KS USA; 6 Present Address: Robert Koch Institute, Berlin, Germany; 7 Present Address: School of Infection & Immunity, University of Glasgow, Glasgow, UK; 8 Present Address: The Sir William Dunn School of Pathology, University of Oxford, Oxford, UK

**Keywords:** Microbiology, Virology & Host Pathogen Interaction, Organelles, Post-translational Modifications & Proteolysis

## Abstract

In many viruses, intrinsically disordered proteins (IDPs) drive the formation of replicative organelles via liquid–liquid phase separation (LLPS). In species A rotaviruses, the disordered protein NSP5 forms condensates with NSP2, but its high sequence diversity raises questions about whether this mechanism is conserved across strains. Using a machine learning approach, we show that NSP5 variants differ significantly in LLPS propensity. We engineered an NSP5 variant with features derived from strains with low-LLPS propensity (low-LLPS). Despite lacking the ability to phase separate in vitro unless phosphorylated, this variant nevertheless supported condensate formation and viral replication in cells. We found that low-LLPS variants require phosphorylation to nucleate phase separation, whereas high-LLPS variants do not, suggesting distinct nucleation mechanisms between viral strains. Hydrogen–deuterium exchange mass spectrometry revealed a phosphorylation-driven allosteric switch that alters NSP2 interactions depending on the NSP5 variant. These findings suggest that phosphorylation plays a context-dependent role in condensate formation, tuning NSP5-NSP2 interactions in a strain-specific manner and highlighting the mechanistic diversity underpinning replicative organelle formation among viral strains.

## Synopsis

Rotavirus replication requires the formation of protein condensates; however, how this process is controlled remains incompletely understood. This study shows that phosphorylation of the rotavirus intrinsically disordered protein NSP5 tunes its propensity for condensation, regulating replication compartment assembly.

- Sequence diversity in NSP5 from species A rotaviruses encodes variable phase separation potential in vitro, while maintaining replication compartment formation in cells.
- NSP5 phosphorylation enables condensate formation in otherwise non-condensing variants.
- NSP5 phosphorylation alters its binding interface with NSP2.
- NSP5 phosphorylation may dynamically tune biomolecular  interactions as virus replication and assembly progress.

## Abstract (web summary)

Rotavirus strains encode NSP5 variants with variable phase separation potential, which is modulated by phosphorylation for successful replication compartment formation.

## Graphical abstract

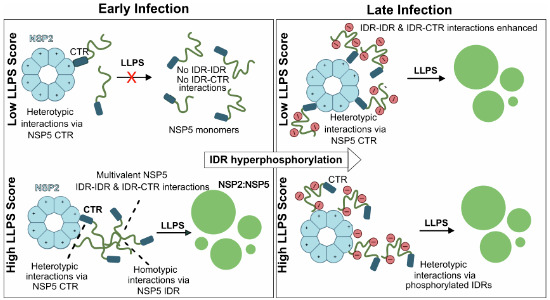

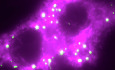

## Introduction

Liquid–liquid phase separation (LLPS) drives the formation of various membrane-less organelles (Hyman et al, 2014), including P-bodies, stress granules, and nucleoli (Banani et al, 2017; Brangwynne et al, 2011, 2009). LLPS is driven by weak interactions between multivalent, polymer-like molecules (Banani et al, 2017; Brangwynne et al, 2015). This mechanism is commonly explained using the “stickers and spacers” model (Choi et al, 2020), where “stickers” refer either to side chain interactions within intrinsically disordered regions (IDRs) (Martin et al, 2020; Wang et al, 2018) or to interactions involving folded domains that recognise specific binding motifs (Banani et al, 2016). Regions separating the stickers, known as spacers, modulate interactions mediated by the stickers (Ginell and Holehouse, 2023). The hub-and-driver model describes a system in which a “hub” molecule characterised by a low intrinsic propensity for LLPS binds to “driver” molecules whose IDRs confer a higher LLPS propensity (Galagedera et al, 2023).

Although LLPS is predominantly governed by the inherent physico-chemical characteristics of macromolecules, understanding the emergent properties of condensates presents a formidable task. Due to their minimalistic proteomes, viruses provide excellent models for dissecting the roles of a relatively small number of components that drive LLPS, and indeed, many viruses employ LLPS to form viral factories that support their replication (Borodavka and Acker, 2023; Heinrich et al, 2018; Monette et al, 2020; Nikolic et al, 2016; Zhou et al, 2022).

**Figure 1:**
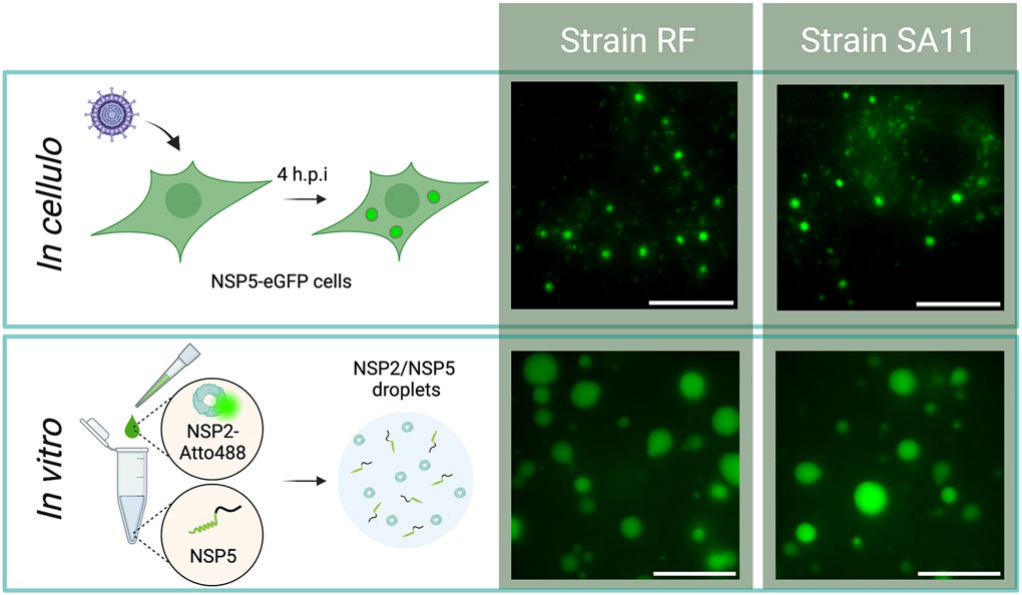
NSP2–NSP5 condensate formation in model RVA strains RF and SA11, observed both in cellulo and in vitro. Upper panel: MA104–NSP5–eGFP cells infected with either RF or SA11 RVAs contain viroplasms. Lower panel: In vitro phase separation properties of Atto-488 dye-labelled NSP5 from RF and SA11 strains in the presence of NSP2. Scale bars, 10 µm. Source data are available online for this figure.

Group A rotaviruses (RVA) represent a large and diverse class of important double-stranded RNA pathogens that infect humans and other animals worldwide. Several RVA strains that infect mammals, including simian SA11 and RRV, bovine RF, and porcine OSU strains, have all been shown to form cytoplasmic replication factories, termed viroplasms (Fig. 1) (Campagna et al, 2007; Carreño-Torres et al, 2010; Geiger et al, 2021). Viroplasms represent protein-RNA condensates formed via LLPS of the non-structural protein NSP5 and the RNA chaperone NSP2 (Fig. 1) (Geiger et al, 2021). Although both proteins are needed to initiate phase separation in vitro, NSP5 functions as the principal scaffold/driver and NSP2 as a hub: only NSP5 can independently form droplets in vitro, a property that is enhanced in the presence of polycations such as poly-L-arginine and poly-L-lysine (Geiger et al, 2021; Nichols et al, 2023). The onset of viroplasm formation depends on both the viral protein expression levels and the multiplicity and duration of infection (Geiger et al, 2021). The 35 kDa RNA chaperone NSP2 forms an octamer with positively charged grooves involved in NSP5/RNA binding (Bravo et al, 2021; Jayaram et al, 2002; Jiang et al, 2006). In contrast, NSP5 is an intrinsically disordered protein (IDP) harbouring an 18 amino acid C-terminal homo-oligomerisation region (CTR) (Geiger et al, 2021). Removal of the CTR renders the remaining IDR unable to drive LLPS when mixed with NSP2 in vitro (Geiger et al, 2021). Amino acids 1 - 33 and the CTR of NSP5 are involved in binding to NSP2 (Arnoldi et al, 2007; Eichwald et al, 2004b; Fabbretti et al, 1999). During infection, the 21 kDa NSP5 undergoes a phosphorylation cascade leading to the formation of phospho-isoforms ranging from 26 to 35 kDa (Afrikanova et al, 1998; Eichwald et al, 2004a). In the cell-culture-adapted strain SA11, nine phosphorylation sites, including Ser2/4, Ser30, Ser37, Ser42, Ser56, Ser67, Ser101, Ser127, and Ser164, have been reported (Sotelo et al, 2010). These residues undergo phosphorylation following the initial phosphorylation of the conserved Ser67 upon NSP2/NSP5 binding (Papa et al, 2019). It remains unclear how sequential NSP5 phosphorylation influences phase separation and the functional state of viroplasms across diverse RVA variants.

Phosphorylation can modulate the phase behaviour of IDPs in diverse ways. It may enhance liquid-like properties in condensates formed by the Adenovirus L1-52/55 kDa protein (Grams et al, 2024), or protect against aberrant liquid-to-solid transitions (Ranganathan et al, 2023). Conversely, phosphorylation may reduce condensate formation (Bressler et al, 2023), highlighting the system-dependent nature of phosphoregulation. Mechanistically, phosphorylation has been proposed to alter the conformational ensemble of IDPs, whereby it can influence subsequent interactions or modifications within the same disordered polypeptide chain through a process known as dynamic allostery (Berlow et al, 2018; Ferreon et al, 2013; Hilser and Thompson, 2007; Kern and Zuiderweg, 2003). However, identification of such allosteric events within IDPs that drive changes in the architecture and composition of condensates remains challenging.

It remains unclear whether all RVA strains utilise LLPS, and if they do, whether they rely on a common, conserved mechanism. Given the large number of RVA strains encoding diverse NSP5 sequences, assessing their individual propensities for LLPS is challenging. This difficulty is compounded by the limited ability of many RVA isolates to grow in cell culture, with strains SA11 and RF being the most characterised as they can be readily grown in cell culture (Arnoldi et al, 2007; Peña-Gil et al, 2023; To overcome these limitations, we employed a machine learning algorithm (Saar et al, 2021) to evaluate the phase-separation potential of NSP5 variants from diverse RVA strains and engineered an SA11-like strain expressing an NSP5 variant with the lowest predicted LLPS propensity, designated SC_Low_.

Here, we compare NSP5 variants with high and low-LLPS propensity to determine how sequence variation and phosphorylation influence phase separation. We test whether phosphorylation is required to enable phase separation in low-propensity variants and examine how it alters NSP5–NSP2 interaction modes. To address this, we combine comparative, biochemical and biophysical, and virological approaches.

Our analysis reveals that phosphorylation plays a dual role: it promotes phase separation in NSP5 variants with low intrinsic LLPS propensity and modulates binding interactions within condensates in high-propensity variants. These findings suggest that phosphorylation tunes NSP5-NSP2 interactions in a strain-dependent manner and acts as a key regulator of viroplasm assembly.

## Results

### Engineering an NSP5 variant with diminished LLPS potential

**Figure 2:**
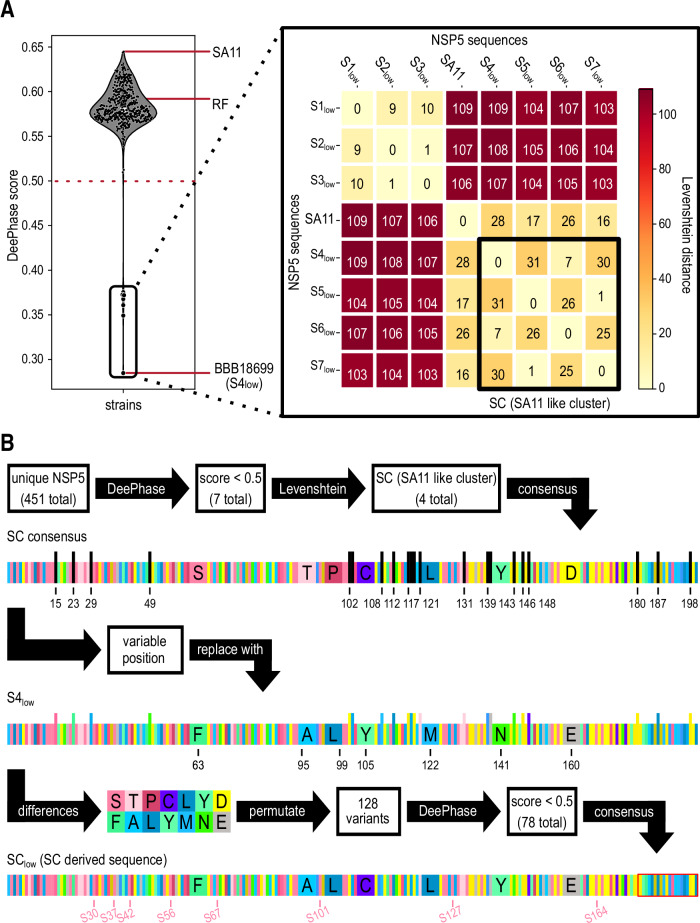
Computational identification and rational design of an NSP5 variant with reduced tendency to undergo LLPS. (**A**) LLPS propensity analysis of 451 unique full-length RVA NSP5 sequences using DeePhase. The violin plot shows most NSP5 variants prone to undergo LLPS exhibiting DeePhase scores above 0.5. The cell-culture-adapted RVA strains RF and SA11 highlighted, along with a naturally occurring strain BBB18699 (designated S4_Low_) that exhibited the lowest DeePhase score. A zoomed-in inset displays the seven low-scoring variants designated S1_Low_-S7_Low_. Sequence similarity was assessed by computing pairwise Levenshtein distances, ranging from 0 (identical sequence) to 110 (maximally divergent). Among these, NSP5 sequences from S4_Low_ - S7_Low_ show the greatest similarity with strain SA11 and are collectively referred to as the SA11-like cluster (SC). (**B**) In silico design of the SA11-like NSP5 variant with low-LLPS propensity (SC_Low_ variant) using DeePhase predictions and conservation analyses. A consensus sequence (SC consensus) was generated from the SA11-like NSP5 cluster, incorporating 21 degenerate positions, which were substituted with corresponding residues from the naturally occurring sequence with the lowest LLPS score (S4_Low_). The seven remaining differences between the SC consensus and S4_Low_ gave rise to 2^7^ (128) sequence permutations. Of these, 78 permutations had a DeePhase score below 0.5. A second consensus was derived from these low-scoring permutations, in which only a single ambiguous amino acid position was resolved by adopting the corresponding residue from the S4_Low_ variant. Phosphorylated serine residues reported by Sotelo et al, 2010 are marked in red, all of which were retained in the final SC_Low_ sequence. Amino acid residues are colour-coded based on their physicochemical properties: aromatic and polar uncharged residues (Y, N, F) are shown in green; charged residues (D, K, H) in yellow; charged residues forming stronger salt bridges (E, R) in grey; small or hydrophobic residues (A, M) are coloured light blue, bulkier aliphatic and polar side chains (I, Q, L) are shown in darker blue. Cysteine (C) is highlighted in purple, and Serine (S), threonine (T), and proline (P) are shown in distinct shades of pink/magenta to distinguish their roles in phosphorylation and secondary structure disruption. The C-terminal region (CTR, residues 181–198, red box) is required for NSP5 homo-oligomerisation (Torres-Vega et al, 2000), remains unperturbed in SC_Low_ and contains no known phosphorylation sites. Source data are available online for this figure.

To evaluate the condensate-forming potential of NSP5 across a range of RVA strains and isolates, we performed an in silico analysis of their LLPS propensities using DeePhase (Saar et al, 2021). RVA NSP5 sequences are ~78% identical overall, with variability observed primarily within the disordered region 102–156 (Blackhall et al, 1997). Of the 451 unique RVA NSP5 sequences retrieved from the NCBI virus database, most exhibited DeePhase scores above 0.5 (Fig. 2A), indicating a high propensity for LLPS (Saar et al, 2021). Notably, the NSP5 protein from the cell culture-adapted SA11 strain possessed the highest DeePhase score among all sequences analysed. Seven NSP5 variants (S1_Low_ to S7_Low_) displayed DeePhase scores below 0.4 (Appendix Table S1), suggesting a limited or absent ability to undergo phase separation. These low-scoring variants represent a minority within the NSP5 sequence space and provide a useful set of outliers to test how reduced intrinsic LLPS propensity affects condensate formation. We therefore focused on these variants to determine whether phosphorylation can compensate for diminished phase separation potential.

To estimate sequence divergence between the well-characterised reference strain SA11 and the identified low-propensity NSP5 variants, we calculated pairwise Levenshtein distances between each S1_Low_ to S7_Low_ sequence and SA11. The Levenshtein distance measures the minimum number of single-residue substitutions, insertions or deletions needed to transform one sequence into another, providing a numerical measure of their overall similarity (Levenshtein, 1966). This analysis identified four SA11‑like variants, designated S4_Low_ through S7_Low_, which form an SA11‑like cluster (SC) based on their similarity to each other and to SA11 (Fig. 2A).

**Figure EV1:**
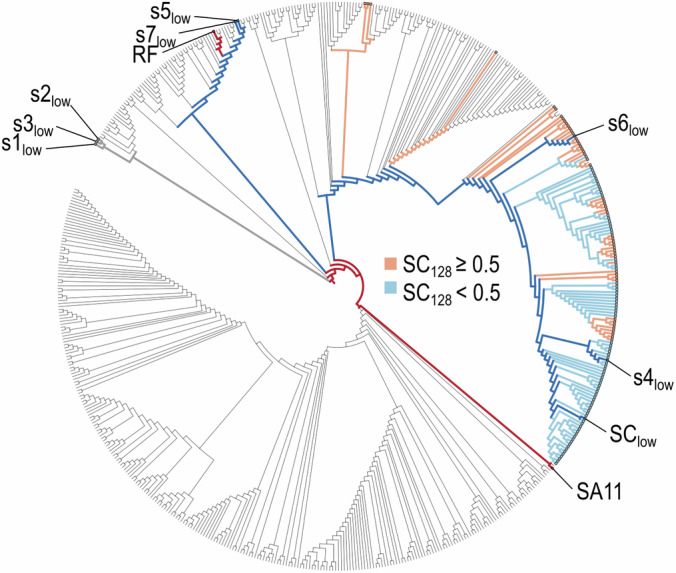
Phylogenetic analysis of NSP5 variants. Phylogenetic analysis of 451 unique NSP5 sequences alongside 128 computationally designed permutation variants derived from the SA11-like cluster (SC). Naturally occurring low-scoring variants (S1_Low_-S7_Low_; DeePhase <0.5) are indicated at their respective tips and represent a minority of NSP5 sequences with reduced predicted LLPS propensity that were selected for further analysis. Cell-culture-adapted reference strains SA11 and RF are highlighted in dark red. The SA11-like cluster (SC) consensus and the engineered SC_Low_ variant are marked in dark blue. Among the 128 permutation variants (SC_128_), tips are coloured orange for sequences with DeePhase scores > 0.5 (high LLPS propensity), and light blue for sequences with scores <0.5 (low-LLPS propensity). Light blue variants therefore denote low-propensity sequences within the SC used to examine how reduced intrinsic LLPS propensity affects condensate formation.

We then engineered an SA11-like NSP5 variant with reduced LLPS propensity by aligning the four naturally occurring SC variants (S4_Low_ - S7_Low_) (Katoh and Standley 2013), and deriving a consensus sequence that preserved conserved regions while introducing substitutions predicted to reduce its LLPS propensity. Given the 24 variable positions in the SC variants (Fig. 2B), we replaced each position with the corresponding residue from S4_Low_, which exhibited the lowest DeePhase score (Appendix Table S1). Since the resulting sequence diverged from S4_Low_ at seven positions, we generated all 2^7^, or 128 combinations of these substitutions and evaluated each variant using DeePhase. Seventy-eight of the 128 variants scored below 0.5 (Fig. 2B), demonstrating that substitutions at these positions significantly influence LLPS propensity (Figs. 2B and  EV1).

Notably, all substitutions mapped exclusively to the intrinsically disordered region (IDR) of NSP5, indicating that sequence variation within the IDR is sufficient to differentiate low‑ and high‑LLPS‑propensity variants. Similar variability in intrinsically disordered regions has been reported in other viral proteins, suggesting that differences in phase behaviour may represent a broader feature of viral evolution. We then generated a consensus sequence from the 78 low-scoring variants, resolving a single ambiguous amino acid position using the corresponding residue from S4_Low_ to obtain the final SC_Low_ sequence with a DeePhase score of 0.36.

### Low-LLPS propensity NSP5 variant SC_Low_ supports viroplasm formation and viral replication

**Figure EV2:**
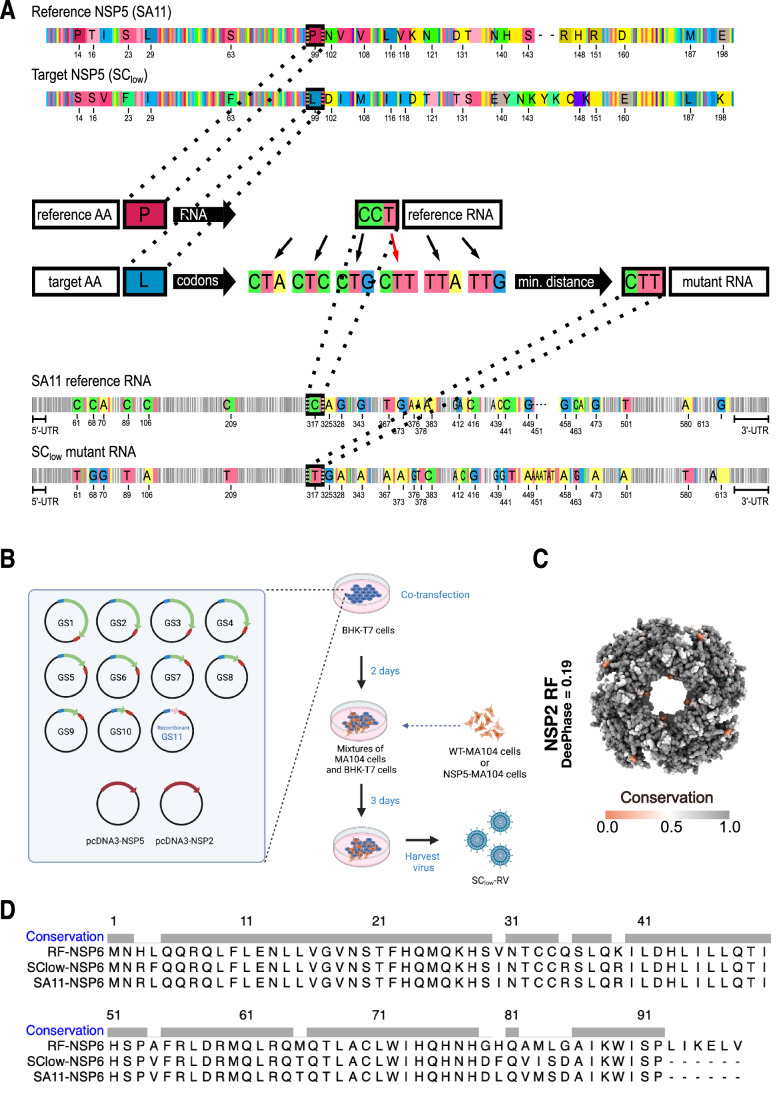
Design, rescue, and genetic stability of the SC_Low_-RV. (**A**) RNA sequence design for gene segment 11 SC_Low_ using the reference RNA sequence of NSP5 SA11. The coding sequence of NSP5 from SA11 was used as a reference, and synonymous codons were selected to minimise nucleotide divergence while preserving amino acid identity wherever possible. In cases of amino acid substitution (e.g., Proline to Leucine at position 99), the target codon employed required the smallest possible number of nucleotide changes. A total of 35 nucleotide changes were introduced in the SC_Low_ sequence, while untranslated regions (UTRs) were left unchanged. Positions of nucleotide substitutions are indicated. (**B**) Schematic overview of the reverse genetics strategy used to generate SC_Low_-RV. Ten pT7 plasmids encoding the SA11 gene segments (GS1–GS10) and one recombinant plasmid encoding SC_Low_, were co-transfected into BHK-T7 cells along with expression plasmids for NSP2 and NSP5 (pcDNA3-NSP2 and pcDNA3-NSP5). At 48 h post-transfection, either WT-MA104 or NSP5-expressing MA104 (NSP5-MA104) cells were overlaid. Recombinant virus was harvested after three freeze–thaw cycles upon observation of full cytopathic effect (CPE), as described in Methods (Papa et al, 2019). (**C**) Conservation analysis of NSP2 across rotavirus A strains. Full-length NSP2 sequences were aligned, and residue conservation was mapped onto the NSP2 octamer crystal structure (PDB: 1L9V; SA11 strain). Key NSP5-binding regions are conserved across strains (Jiang et al, 2006). (**D**) Multiple sequence alignment of the NSP6 open reading frame encoded by gene segment 11. The NSP6 sequences of SA11, RF, and SC_Low_ sequence were compared, confirming preservation of NSP6 in the engineered SC_Low_-RV genome.

We next evaluated whether the SC_Low_ variant could support replication of an SA11-like RVA in cell culture. Given the segmented nature of the RVA genome, we considered the possibility that sequence mismatches between the SC_Low_ NSP5 gene (gene segment 11) and the SA11 backbone might impair virus rescue or propagation. To minimise potential inter-segmental incompatibility, we synonymously substituted all nucleotides in the SC_Low_ NSP5 coding sequence that differed from the SA11 reference, selecting synonymous codons with the smallest evolutionary distance to the original sequence, as detailed in the “Methods” (Fig. EV2A).

**Figure 3:**
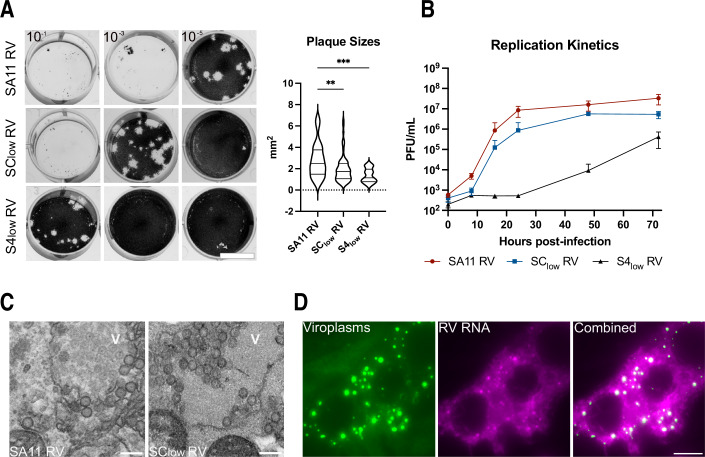
SC_Low_-RV supports replication, particle production, and viroplasm formation in cell culture. (**A**) Plaques formed by SA11-RV, SC_Low_-RV and S4_Low_-RV in MA104 NSP5 cells at 24 h.p.i. are shown at a 10^−1^ (left), 10^−3^ (middle) and 10^−5^ (right) dilutions. Scale bar, 10 mm. Violin plots show plaque areas measured for SA11-RV at 10^−5^, SC_Low_-RV at 10^−3^, and S4_Low_-RV at 10^−1^. Statistical significance was assessed by ordinary one-way ANOVA with a Dunnett’s multiple-comparisons test from three biological replicates (adjusted *P* values: **, 0.0012 and ***, 0.0005). (**B**) Replication kinetics of SA11-RV, SC_Low_-RV and S4_Low_-RV in MA104 cells infected at a multiplicity of infection (MOI) of 1. Viral titres were quantified at 8, 16, 24, 48 and 73 h.p.i. by TCID₅₀ assays. Data points represent the mean ± standard deviation (SD) from three independent experiments. (**C**) Transmission electron micrographs of MA104 cells infected with SA11-RV (left) and SC_Low_-RV (right), fixed at 8 h.p.i. Multiple viral particles are observed within viroplasms (V). Scale bar, 200 nm. (**D**) SC_Low_-RV forms viroplasms containing viral transcripts. Atto647N-labelled smFISH probes were used to visualise viral transcripts in EGFP-tagged viroplasms. Scale bar, 10 μm. Source data are available online for this figure.

We used a modified plasmid-based reverse genetics system (Fig. EV2B), incorporating the SC_Low_-encoding gene segment 11, to rescue SC_Low_-RV in an MA104 cell line stably expressing wild-type NSP5 (NSP5-MA104), a platform previously employed to recover NSP5-deficient viruses (Papa et al, 2019). Upon rescue, SC_Low_-RV could also replicate in wild-type MA104 cells, confirming that SC_Low_ NSP5 was functional (Fig. 3). Sequencing of the SC_Low_ NSP5 gene from recombinant rotavirus indicated that its sequence remained stable for at least ten passages in MA104 cells at a low multiplicity of infection (Appendix Fig. S1).

Regions of NSP2 previously implicated in NSP5 binding (residues 64–68, 179–183, 232–251 and 291–302 (Jiang et al, 2006)) were conserved in SC_Low_-RV and remained unchanged after ten sequential passages, suggesting that NSP2 retains its ability to interact with the low-scoring NSP5 variant (Fig. EV2C). As SA11 gene segment 11 also encodes NSP6, which enhances viral growth (Komoto et al, 2017), we analysed the modified segment and confirmed that NSP6 was retained in SC_Low_-RV (Fig. EV2D).

To further characterise SC_Low_-RV, we compared plaque morphologies of SC_Low_-RV and SA11-RV. Despite comparable end titres, plaques formed by SC_Low_-RV were significantly smaller than those of SA11-RV (2.0 ± 1.4 mm^2^ vs 3.0 ± 1.7 mm^2^, respectively) (Fig. 3A). We next assessed SC_Low_-RV by comparing its replication kinetics with the WT virus. SC_Low_-RV replicated more slowly than SA11-RV in MA104 cells (Fig. 3B), although this difference diminished at later stages of infection (48 h.p.i.), consistent with the similar end titres observed. We also rescued a recombinant SA11-based RVA, designated S4_Low_-RV that encodes the NSP5 from S4_Low_ (Fig. 2A; Appendix Table S1), a naturally occurring low-LLPS propensity NSP5 variant. Notably, S4_Low_-RV replicated more slowly than the SA11-optimised SC_Low_-RV and reached significantly lower end-point titres (Fig. 3B), underscoring the usefulness of our sequence- optimisation strategy for engineering the SC_Low_ variant. We confirmed viral particle production by SC_Low_-RV using electron microscopy (EM) imaging at 8 h.p.i., which revealed viral particles adjacent to viroplasms as also seen in SA11-infected cells (Fig. 3C). Single-molecule RNA fluorescence in situ hybridisation (smFISH) (Strauss et al, 2023) further confirmed that SC_Low_-RV viroplasms contained viral transcripts (Fig. 3D). Collectively, these data show that SC_Low_-RV supports viroplasm formation and robust replication in cell culture, and that both the engineered (SC_Low_-RV) and naturally occurring (S4_Low_-RV) low-LLPS NSP5 variants remain replication-competent, albeit with distinct replication kinetics and end-point titres.

### SC_Low_-RV displays delayed viroplasm maturation and reduced NSP5 hyperphosphorylation

**Figure 4:**
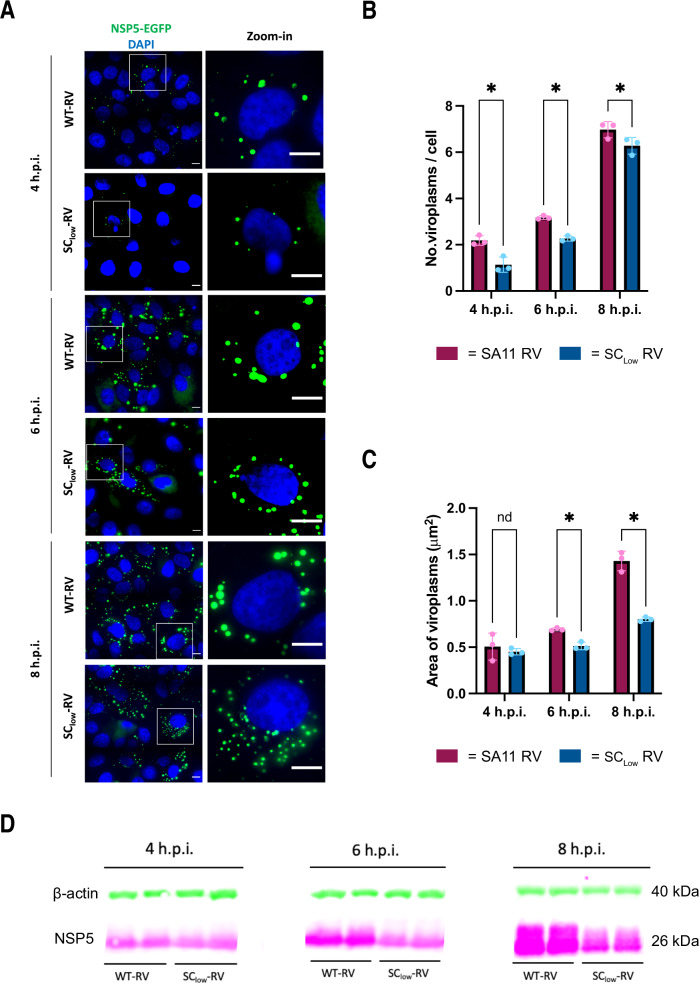
Delayed viroplasm formation and impaired NSP5 hyperphosphorylation in cells infected with SC_Low_-RV. (**A**) Wide-field fluorescence microscopy of MA104-NSP5-eGFP cells infected with either SA11-RV (WT-RV) or SC_low_-RV at MOI of 20. NSP5-eGFP–labelled viroplasms (green) and nuclei (DAPI, blue) are shown at 4-, 6-, and 8-hours post-infection (h.p.i.). Insets show enlarged regions of interest. Scale bar, 10 µm. (**B**, **C**). Quantification of viroplasm number (**B**) and area (**C**), in μm²) formed in cells infected by SA11-RV (burgundy) or SC_Low_-RV (dark blue). Data represent mean values from three independent experiments (*N* > 200 cells per condition). Error bars indicate the standard error of the mean (SEM). Two-way ANOVA was used to compare SA11-RV and SC_Low_-RV at each time point: viroplasm number at 4 h.p.i. (*P = 0.0004), 6 h.p.i. (*P = 0.0012), 8 h.p.i. (*P = 0.0071); viroplasm area (μm²) at 4 h.p.i. (ns = 0.9244), 6 h.p.i. (*P = 0.0278), 8 h.p.i. (*P = 0.0211). (**D**) Immunoblot analysis of NSP5 extracted from MA104 cells infected with SA11-RV or SC_Low_-RV at 4, 6 and 8 h.p.i. (MOI = 20). Note the absence of the strongly hyperphosphorylated 35 kDa NSP5 isoform in SC_Low_-RV-infected cells at later time points. Source data are available online for this figure.

Having established that SC_Low_-RV replicates and forms viroplasms in infected cells, we next examined the kinetics of viroplasm formation in cells infected with SC_Low_-RV compared to SA11-RV. To visualise viroplasms, we infected NSP5-eGFP MA104 cells with either SC_Low_-RV or SA11-RV. Infected cells were imaged at 4, 6, and 8 h post infection. Both SC_Low_-RV and SA11-RV produced spherical NSP5–eGFP-tagged condensates as early as 4 h.p.i. (Fig. 4A). However, a marked reduction in both the abundance and area (μm²) of viroplasms was observed in SC_Low_-RV-infected cells compared to SA11-infected cells at early time points (up to 8 h.p.i.) (Fig. 4B,C).

Given that hyperphosphorylation of NSP5 increases progressively during infection (Papa et al, 2019), we next investigated whether SC_Low_ -RV undergoes a similar phosphorylation trajectory. From 6 h.p.i. onward, a notable difference in NSP5 hyperphosphorylation emerged between the two strains. This was especially pronounced at 8 h.p.i., when the most highly phosphorylated isoform of NSP5 (migrating at ~35 kDa) was absent in SC_Low_-RV-infected cells (Fig. 4D). These findings suggest that the delayed and reduced viroplasm formation observed in SC_Low_-RV-infected cells may be linked to impaired NSP5 hyperphosphorylation. Despite these differences, viral replication proceeds efficiently, indicating that altered condensate size and abundance reflect delayed maturation rather than loss of function.

### Phosphorylation restores phase separation and viroplasm formation by a low-LLPS NSP5 variant

**Figure 5:**
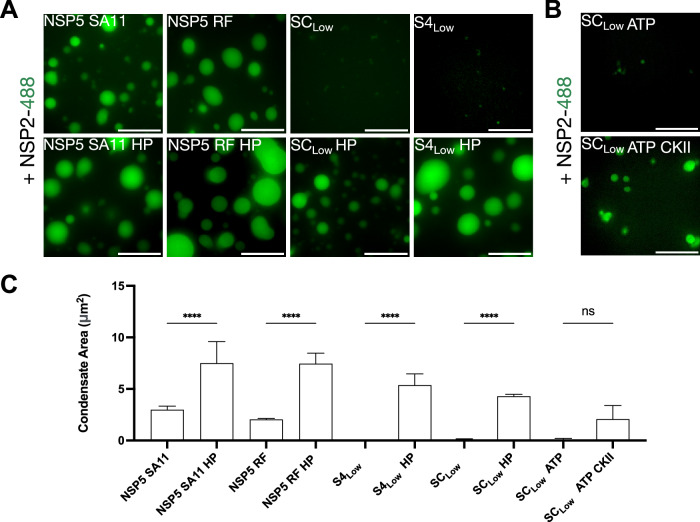
Hyperphosphorylation of SC_Low_ restores its ability to phase separate in vitro*.* (**A**) In vitro phase-separation assays of NSP5 variants in the presence of NSP2. Atto-488-labelled NSP2 (25 µM) was mixed with equimolar amounts of unlabelled NSP5 variants, as described in Methods. Images are arranged with NSP5 variants in the top row (left to right: NSP5 SA11, NSP5 RF, SC_Low_, S4_Low_) and the corresponding hyperphosphomimetic (HP) variants directly below (left to right: NSP5 SA11 HP, NSP5 RF HP, SC_Low_ HP, S4_Low_ HP). Scale bar, 10 μm. (**B**) In vitro phosphorylation assay of SC_Low_. ATP was added to reactions containing NSP2 and SC_Low_ (top), and ATP plus CKII was added to parallel reactions (bottom), as described in Methods. Fluorescence microscopy images show representative fields of view. Scale bar, 10 µm. (**C**) Quantification of mean condensate area (μm²) for each NSP5 variant mixed with NSP2. Condensates were imaged within a 500 µm × 800 µm region of interest in three biological replicates. Data were analysed by one-way ANOVA with Bonferroni and Sidak multiple comparisons test (α = 0.05). ****P < 0.0001. Source data are available online for this figure.

**Figure EV3:**
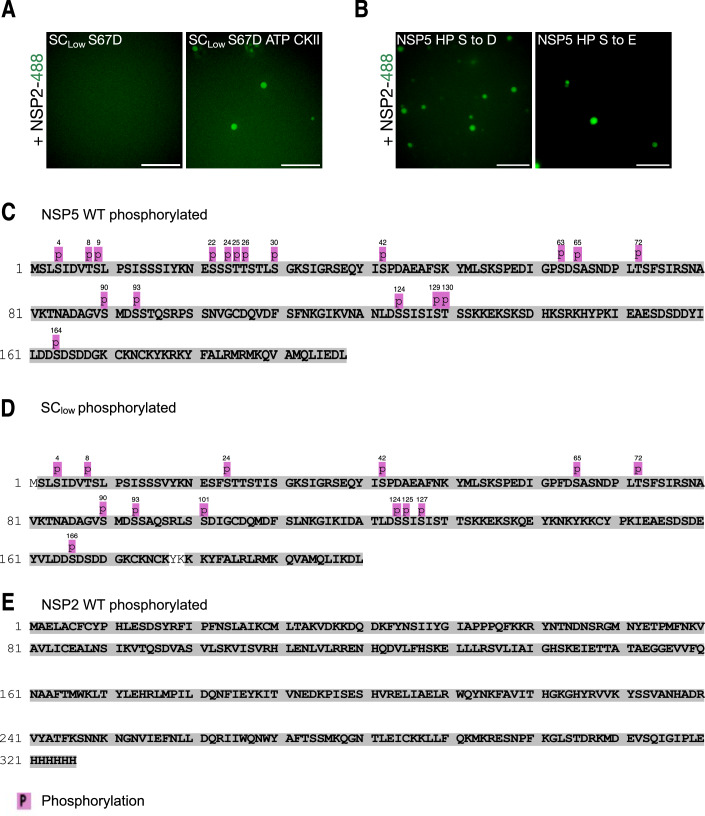
Analysis of phosphorylation of NSP5 variants in vitro. (**A**) Comparison of NSP5 HP variants S-to-D *vs* S-to-E. In vitro phase-separation assays of NSP5 in the presence of NSP2. Atto-488-labelled NSP2 (25 µM) was mixed with equimolar amounts of unlabelled NSP5 variants and imaged as described in Methods. Scale bar, 10 μm. (**B**) In vitro phase-separation assays of phosphomimetic S67D (strain SA11) in the presence of NSP2, without CKII + ATP incubation (left) and upon phosphorylation by CKII (right). Scale bar, 10 μm. (**C**–**E**) Sequence coverage maps showing phosphorylation sites of NSP5 variants and NSP2 after in vitro phosphorylation. Sequence coverage is shown for NSP5 RF (**C**) and SC_Low_ (**D**) and NSP2 (**E**). Confidently identified phosphorylation sites are indicated by pink boxes. No phosphorylation sites were confidently identified in the untreated samples.

To determine whether phosphorylation restores phase separation in low-LLPS variants, we analysed the behaviour of SC_Low_ in a minimal in vitro system containing NSP2 and SC_Low_. Unlike its wild-type counterpart, SC_Low_ failed to form condensates upon mixing with NSP2 (Fig. 5), in agreement with DeePhase predictions. Given that NSP5 undergoes progressive phosphorylation during RVA infection (Papa et al, 2019), and that we observed differences between WT-NSP5 and SC_Low_ NSP5 phosphorylation patterns, we next asked whether phosphorylation could restore the ability of SC_Low_ to phase separate in vitro. Since S67D substitution was previously shown to restore NSP5 hyperphosphorylation (Eichwald et al, 2004a), suggesting that this phosphomimetic is functional in this context, we introduced eight S-to-D mutations into both the WT-NSP5 (strain SA11) and the SC_Low_ sequence (Fig. 2C). All S-to-D substitutions targeted residues previously identified as phosphorylation sites in hyperphosphorylated NSP5 (Sotelo et al, 2010). As an additional control, we also generated an S-to-E mutant (Fig. EV3A), along with an NSP5 from strain RF that was previously shown to undergo phase separation both in vitro and in cells (Geiger et al, 2021). These phosphomimetic variants derived from SA11, RF, or SC_Low_ are referred to as NSP5 SA11 HP, NSP5 RF HP and SC_Low_ HP, respectively.

Condensates formed by the HP variants of both SA11 and RF strains were significantly larger than those formed by their non-phosphorylated counterparts, irrespective of the choice of phosphomimetic (Fig. 5). Importantly, unlike SC_Low_, the SC_Low_ HP variant formed condensates when mixed with NSP2 at low micromolar concentrations (Fig. 5). As the RV strain BBB18699 (S4_Low_) shows the lowest DeePhase score of all RVA variants tested (Fig. 2A), we then tested its ability to undergo LLPS, along with its HP mutant. Similarly to SC_low,_ S4_Low_ only forms condensates upon introduction of eight S-to-D mutations (S4_Low_ HP), confirming that phase separation of low-propensity variants is phosphorylation dependent (Fig. 5A,C). Similarly, SC_Low_ treated with ATP and casein kinase II (CKII) formed condensates unlike the SC_Low_ control treated with ATP only (Fig. 5B,C). Phosphorylation of SC_Low_ was confirmed by liquid chromatography-mass spectrometry (Fig. EV3). Interestingly, while S67D substitution was previously shown to restore NSP5 hyperphosphorylation (Eichwald, 2004a), a single S67D substitution failed to restore the ability of SC_Low_ to phase separate. In contrast, phosphorylation with CKII enabled efficient condensate formation (Fig. EV3B), indicating that multi-site phosphorylation across the IDR is required to reach the threshold necessary for phase separation.

**Figure 6:**
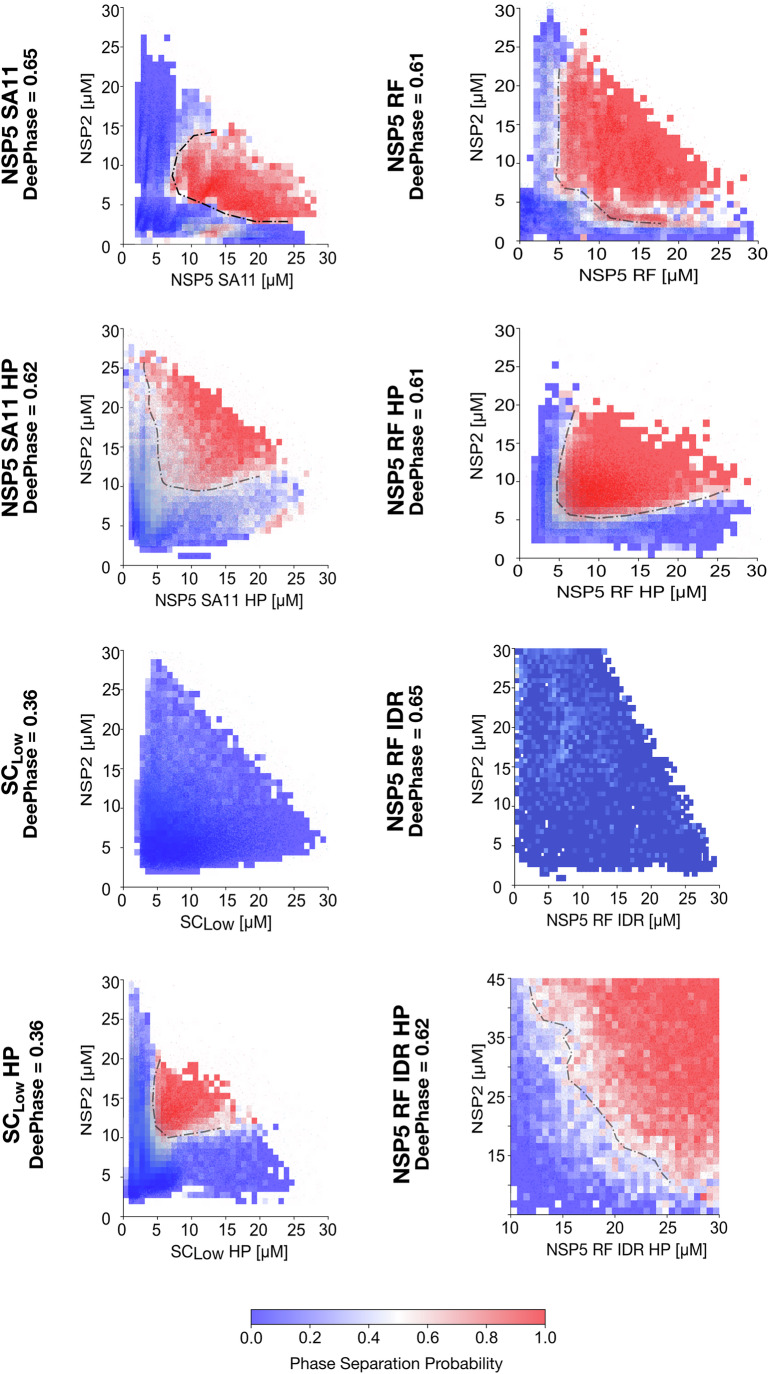
Phase behaviour of NSP5 variants in the presence of NSP2 reveals phosphorylation-dependent coacervation. PhaseScan-derived phase diagrams showing coacervation between NSP2 and different NSP5 variants. Combinatorial microfluidics was used to generate high-resolution phase diagrams for Atto-488-labelled NSP2 and AlexaFluor647-labelled NSP5 variants across a concentration matrix (2–25 µM for each protein), as detailed in Methods. LLPS probability heatmaps were reconstructed from the following number of droplets: NSP5 SA11 (*n* = 46,230), NSP5 SA11 HP (*n* = 87,053), SC_Low_ (*n* = 55,516), SC_Low_ HP (*n* = 101,659), NSP5 RF (*n* = 40071), NSP5 RF HP (*n* = 44,082), NSP5 IDR (*n* = 36,511), and NSP5 IDR HP (*n* = 66,455). LLPS probability is colour-coded from low (blue) to high (red), with black dotted lines indicating inferred phase boundaries. Source data are available online for this figure.

To quantify how phosphorylation alters NSP5–NSP2 phase behaviour, we next used PhaseScan to generate phase diagrams of NSP2 coacervation with different NSP5 variants (Fig. 6). These data show that both SA11 and RF HP variants formed condensates at lower concentrations (i.e., reduced saturation concentration, C_sat_) (Fig. 6).

LLPS driven by homotypic interactions is characterised by a well-defined C_sat_, above which dense phases grow continuously as more protein is added. This behaviour is evident in the phase diagram of non-phosphomimetic NSP5, which shows phase boundaries parallel to the concentration axes without re-entrant behaviour (Fig. 6). In contrast, LLPS driven by heterotypic interactions can be saturated by an excess of either component, leading to re-entrant behaviour, where condensates form and then dissolve as the stoichiometric ratio changes. This behaviour was observed in phosphomimetic NSP5 variants, including SC_Low_ HP, which display curved phase boundaries (Fig. 6).

**Figure 7:**
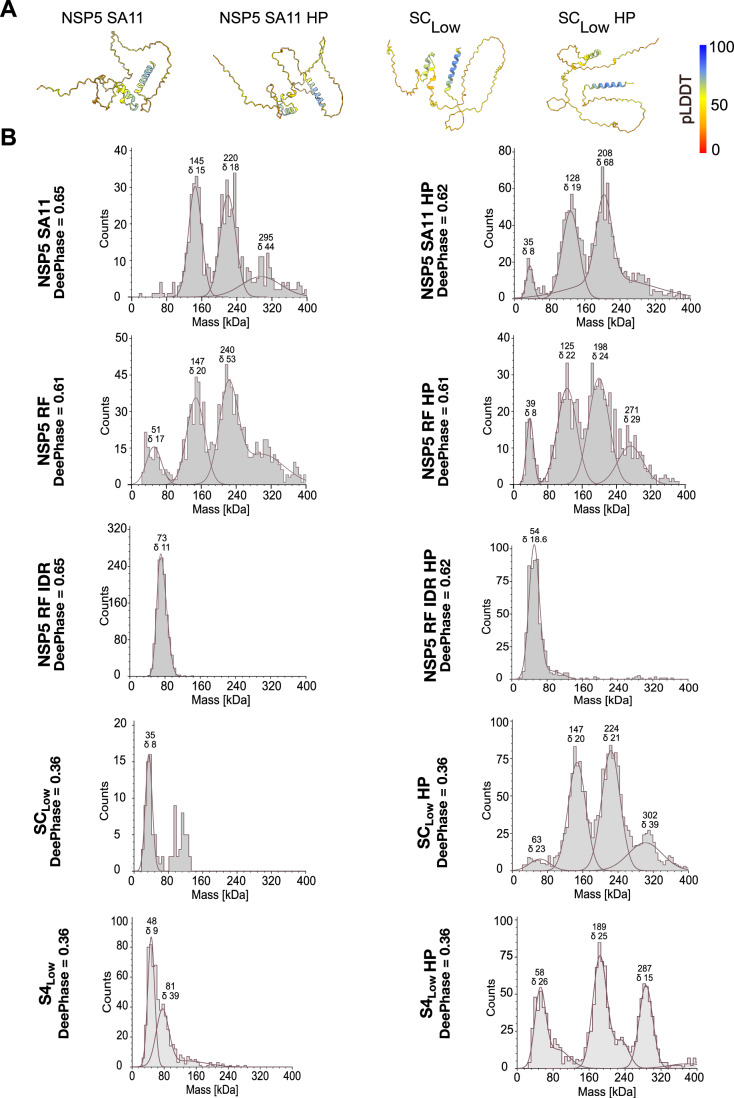
Hyperphosphorylation enables oligomerisation of low-LLPS propensity NSP5 variants. (**A**) AlphaFold2 structural models for NSP5 SA11, NSP5 SA11 HP, SC_Low_ and SC_Low_ HP. Models are coloured by per-residue confidence scores (pLDDT), with blue (pLDDT >90) indicating high confidence and orange-red (pLDDT <50) marking regions predicted to be intrinsically disordered. (**B**) Oligomeric size distributions of NSP5 variants determined by mass photometry. Recombinant NSP5 SA11, NSP5 SA11 HP, SC_Low_, SC_Low_ HP, NSP5 RF, NSP5 RF HP, or NSP5 RF IDR were analysed at 100 nM each, as described in Methods. Only events above the detection threshold were identified as major species, with Gaussian fits showing their molecular weights. Note that SC_Low_ does not exhibit formation of the characteristic ensemble of larger oligomers in solution. Approximate molecular weights (in kDa) and corresponding standard deviations (σ) are indicated. Source data are available online for this figure.

To better understand the mechanistic basis of these differences, we examined NSP5 structural models generated by AlphaFold2 (AF2) (Jumper et al, 2021) since no experimental structures of NSP5 are available. WT and SC_Low_ monomers displayed similar structural features: a putative C-terminal α-helical region (CTR) and a largely disordered N-terminal region (IDR), also preserved across all HP variants (Fig. 7A). All amino acid changes that reduced the DeePhase score of NSP5 mapped to the IDR, whereas the putative CTR was conserved among all strains analysed.

As monomeric AF2 models did not reveal differences between variants, we next asked whether differences in oligomerisation could account for their distinct phase behaviour. Since oligomerisation of multivalent scaffold proteins can nucleate LLPS by altering stoichiometry or affinity (Banani et al, 2017), we examined the oligomerisation of NSP5 variants using mass photometry. NSP5 from the SA11 and RF strains, as well as their hyperphosphomimetic (HP) variants formed a range of higher-order oligomers. In contrast, SC_Low_ behaved markedly differently, predominantly yielding a peak at 35 ± 5 kDa (Fig. 7B), consistent with a monomer/dimer species and similar to the IDR alone. Notably, hyperphosphomimetic variants of SC_Low_ and S4_Low_ restored both oligomerisation and condensate formation. Together, these data suggest that hyperphosphorylation of the NSP5 IDR promotes oligomerisation and phase separation in low-LLPS propensity variants.

**Figure 8:**
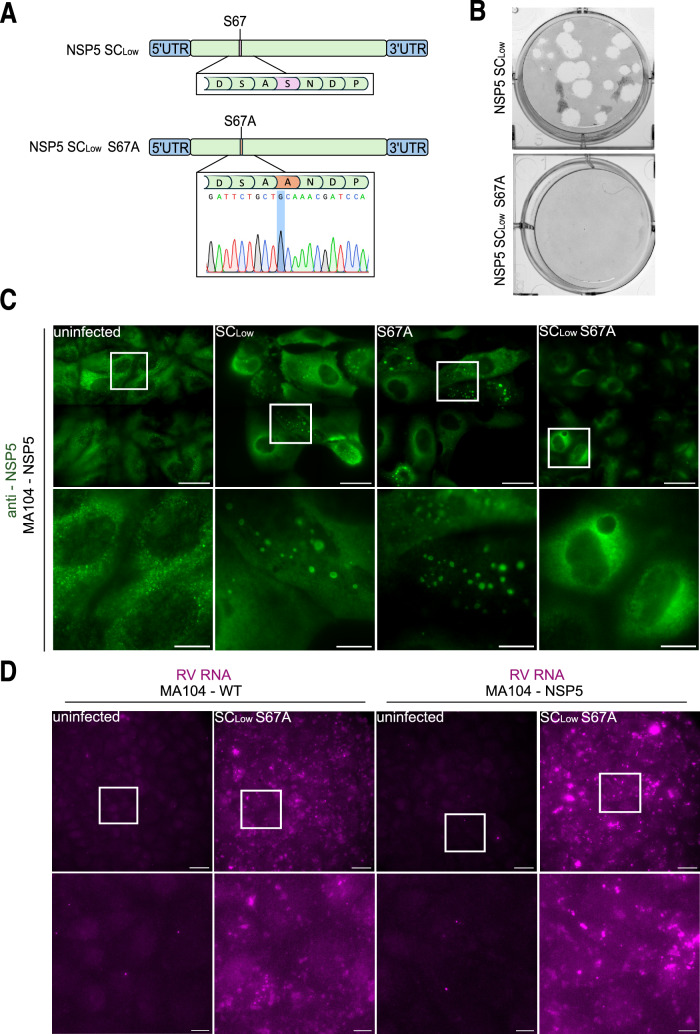
The SC_Low_ S67A-RV mutant fails to form viroplasms in infected cells despite active viral transcription. (**A**) Schematic representation of the S67A mutation introduced into gene segment 11 of SC_Low_, with corresponding Sanger sequencing chromatograms confirming the mutation. (**B**) Representative plaque assays in MA104-NSP5 cells infected with SC_Low_-RV or SC_Low_ S67A-RV. (**C**) Immunofluorescence (IF) imaging of NSP5 in NSP5-expressing cells infected with SC_Low_-RV, SA11 S67A-RV or SC_Low_ S67A-RV at an MOI of 10. Images were acquired at 24 h.p.i. Scale bars: overview: 30 µm; zoomed regions: 10 µm. (**D**) RNA FISH detection of viral transcripts (gene segment 1) in MA104 WT and MA104-NSP5 cells infected with SC_Low_ S67A-RV, using Atto647N-labelled probes. Viral RNA accumulates in both cell types, indicating transcriptional activity. Scale bars: overview: 30 µm; zoomed regions: 10 µm. Source data are available online for this figure.

Given that phosphorylation enables phase separation of SC_Low_, we hypothesised that abrogation of phosphorylation would impair viroplasm formation and viral propagation in cells infected with the SC_Low_-RV. To test this, we introduced the S67A mutation, previously shown to abrogate NSP5 hyperphosphorylation (Papa et al, 2019), into the SC_Low_ background to generate the SC_Low_ S67A recombinant RVA (Fig. 8A). While the SA11 S67A mutant was able to replicate and form viroplasms in MA104 cells, the SC_Low_ S67A virus could only propagate in MA104-NSP5 cells expressing NSP5 in trans and failed to form visible plaques or viroplasms (Fig. 8B,C). Nonetheless, RV transcripts accumulated in MA104 and MA104-NSP5 cells infected with SC_Low_ S67A-RV, indicating that viral transcription occurred in both contexts (Fig. 8D). Together, these findings support the conclusion that phosphorylation is critical for SC_Low_ NSP5 to undergo LLPS, form viroplasms, and support efficient rotavirus replication.

### Phosphorylation tunes NSP5 binding modes in condensates

Phosphorylation events in NSP5 are confined to the intrinsically disordered region (IDR) (Fig. 2C) (Sotelo et al, 2010). While the NSP5 IDR has been primarily implicated in mediating NSP2–NSP5 interactions (Eichwald et al, 2004b; Fabbretti et al, 1999), prior studies, including ours, have shown that the CTR is essential for NSP5 self-association (Geiger et al, 2021; Martin et al, 2011). However, the absence of efficient homo-oligomerisation in SC_Low_ (Fig. 7) suggests that the IDR also contributes to NSP5 self-association.

**Figure EV4:**
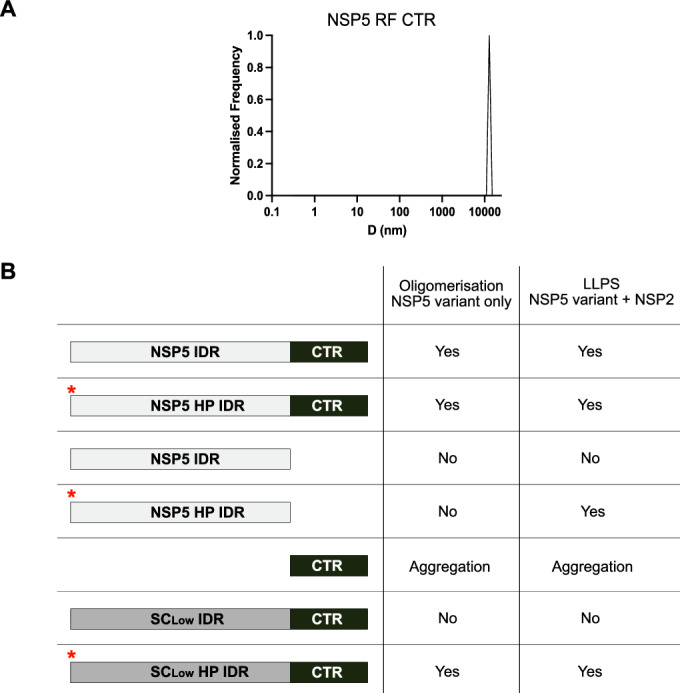
The C-terminal region (CTR) of NSP5 alone forms large aggregates. (**A**) Hydrodynamic diameters (D, in nm) of 1 μM NSP5-RF CTR peptide measured in PBS pH 7.4, as described in Methods, using dynamic light scattering (DLS). (**B**) Summary of the oligomeric states and LLPS capacities of examined NSP5 constructs. Schematic representations of the protein constructs are shown; phosphomimic variants are indicated with a red asterisk.

To investigate this further, we analysed the CTR peptide alone using dynamic light scattering, which revealed the formation of large aggregates (Fig. EV4A). This finding suggests that the IDR normally inhibits uncontrolled CTR aggregation, implying that both regions act cooperatively in oligomerisation and phase separation, with the IDR effectively solubilising the CTR. Consistent with this, deletion of the CTR (yielding the IDR-only construct) abolished homo-oligomerisation (Figs. 7B and  EV4B) and eliminated NSP2-driven phase separation (Fig. 6) (Geiger et al, 2021).

**Figure 9:**
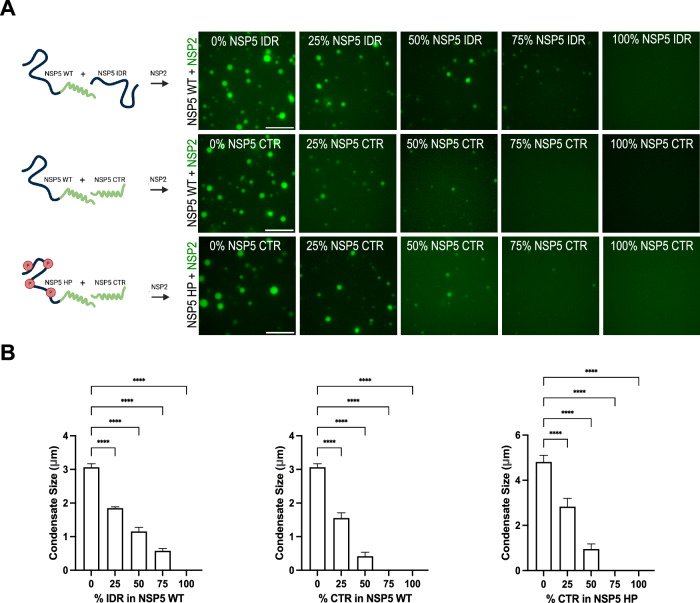
Both the NSP5 C-terminal region (CTR) and intrinsically disordered region (IDR) are critical for mediating LLPS. (**A**) Experimental setup to assess the individual contributions of the NSP5 IDR and CTR to phase separation. Wild-type NSP5 or its phosphomimetic variant (NSP5 HP) was mixed with increasing ratios of either the isolated IDR or CTR, followed by the addition of NSP2 to induce LLPS. Schematics illustrate NSP5 domains: IDR (black), CTR (green), and phosphomimetic sites (red ‘P’). Phase-separated condensates were imaged by wide-field microscopy following mixing of Atto-488-labelled NSP2 (25 μM) with total NSP5 (25 μM) at the indicated full-length: CTR or full-length:IDR ratios. Scale bar, 10 μm. (**B**) Quantification of condensate area (μm²) formed by NSP2 with wild-type or HP NSP5 in the presence or absence of IDR or CTR fragments. Nine regions of interest (50 µm × 80 µm) were analysed per condition, as described in “Methods”, error bars represent the standard deviation. Statistical analysis was performed using one-way ANOVA with Dunnett’s multiple comparisons test (α = 0.05): **P = 0.0082, ***P = 0.0003, ****P < 0.0001. Source data are available online for this figure.

To dissect the relative contributions of the NSP5 IDR and CTR to condensate integrity, we titrated isolated IDRs or CTRs into preformed NSP5/NSP2 condensates. The CTR strongly disrupted condensate formation, whereas the IDR was better tolerated, requiring approximately threefold higher concentration to achieve a comparable effect (Fig. 9A,B). This indicates that CTR-mediated interactions are more potent but also more disruptive to the condensate network, consistent with their aggregation-prone nature.

Notably, condensates formed with phosphomimetic NSP5 (NSP5 HP) displayed increased tolerance to CTR addition. This suggests that phosphorylation of the IDR rebalances the interaction network within condensates, reducing the dominance of CTR-mediated interactions. In this context, hyperphosphorylation does not simply enhance IDR-mediated interactions but instead shifts the relative contributions of CTR and IDR interaction modes. These data suggest that in the unphosphorylated state, condensate assembly is primarily driven by CTR-mediated interactions, with the IDR playing a modulatory role. Upon phosphorylation, the IDR acquires the ability to engage NSP2 more directly, redistributing interaction valency across the molecule. This results in a more distributed and robust interaction network that is less sensitive to perturbation by the CTR.

Given that SC_Low_ fails to phase separate unless it is hyperphosphorylated or phosphomimetic mutations are introduced, we hypothesised that phosphorylation of the IDR may also regulate its interaction with NSP2. To test this, we introduced phosphomimetic mutations into the NSP5 IDR (IDR HP). Like its unmodified counterpart, IDR HP failed to form higher-order oligomers (Figs. 7B and EV4B). However, in the presence of NSP2, IDR HP was able to phase separate, albeit requiring ~4-fold higher concentrations compared to the WT-NSP5 (Fig. 6). These data indicate that IDR phosphorylation is required for coacervation with NSP2, while the CTR remains essential for efficient condensation, as reflected in the elevated C_sat_ of IDR HP.

**Figure EV5:**
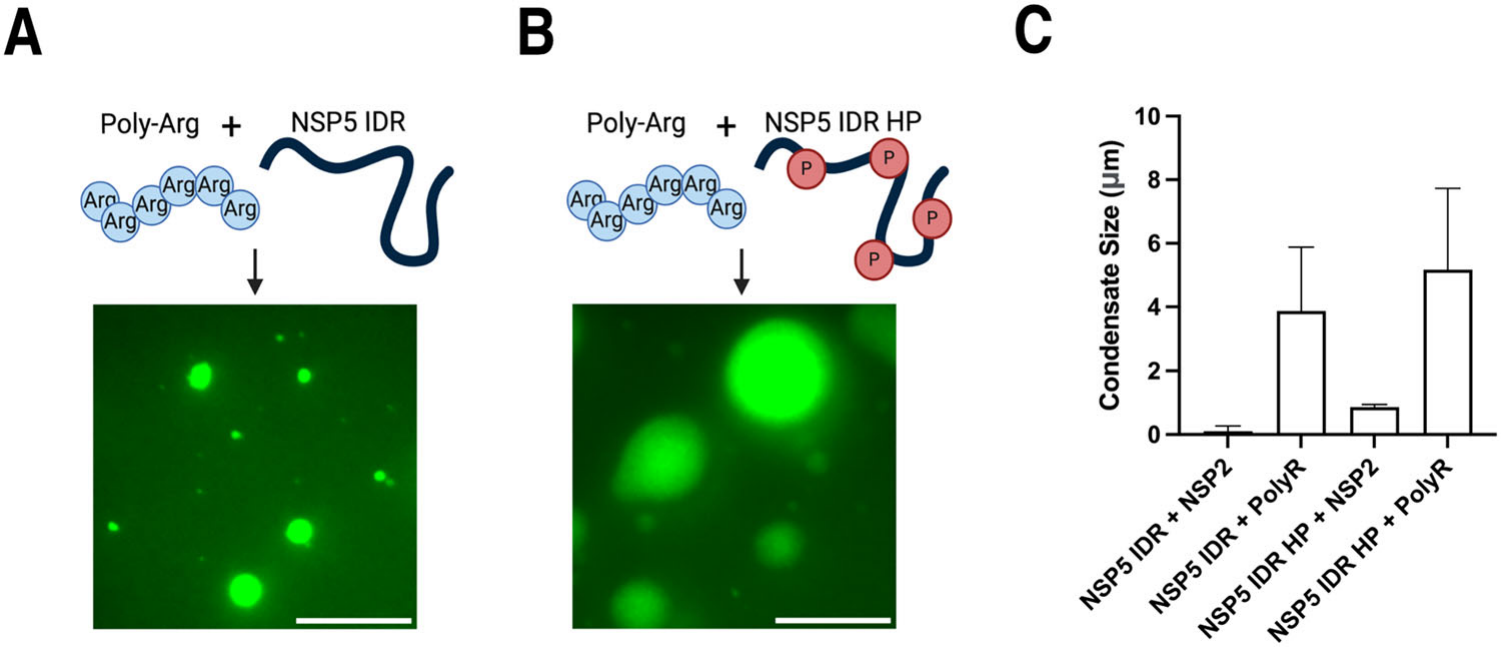
Both phosphomimetic and non-phosphomimetic NSP5 IDRs undergo phase separation with poly-arginine. (**A**, **B**). Phase separation of the NSP5 IDR (**A**) and its phosphomimetic variant IDR HP (**B**) in the presence of poly-L-arginine (poly-Arg, average molecular weight ~40 kDa). DyLight-488-labelled IDR or IDR HP (25 μM; schematically shown, with phosphomimetic sites indicated by red ‘P’) was mixed with poly-Arg (5 μM), and condensates were imaged by wide-field fluorescence microscopy. Scale bar, 10 μm. (**C**) Quantification of condensate area (μm²) formed by NSP5 IDR or IDR HP with poly-L-arginine (PolyR). Nine regions of interest (50 µm × 80 µm each) were analysed as described in “Methods”. Error bars represent standard deviation from three independent technical repeats. For comparison, the condensate area formed by NSP2 with NSP5 IDR or IDR HP (Fig. 9) are shown.

Since the non-phosphomimetic IDR did not measurably bind NSP2 (no droplets formed), we hypothesised that phosphorylation promotes phase separation via electrostatic interactions with the positively charged NSP2. To test whether positively charged polymers can drive condensation of the NSP5 IDRs, we examined the effect of poly-arginine, which has been reported to promote phase separation of NSP5 through electrostatic and cation–π interactions (Geiger et al, 2021). While NSP2 failed to induce condensation of the non-phosphomimetic IDR, poly-arginine efficiently promoted condensate formation for both IDR variants (Fig. EV5). These results support a model in which positively charged polymers can promote NSP5 IDR condensation, whereas NSP2 selectively engages the phosphorylated form, indicating that phosphorylation modulates NSP5 interaction modes in an NSP2-dependent manner.

**Figure 10:**
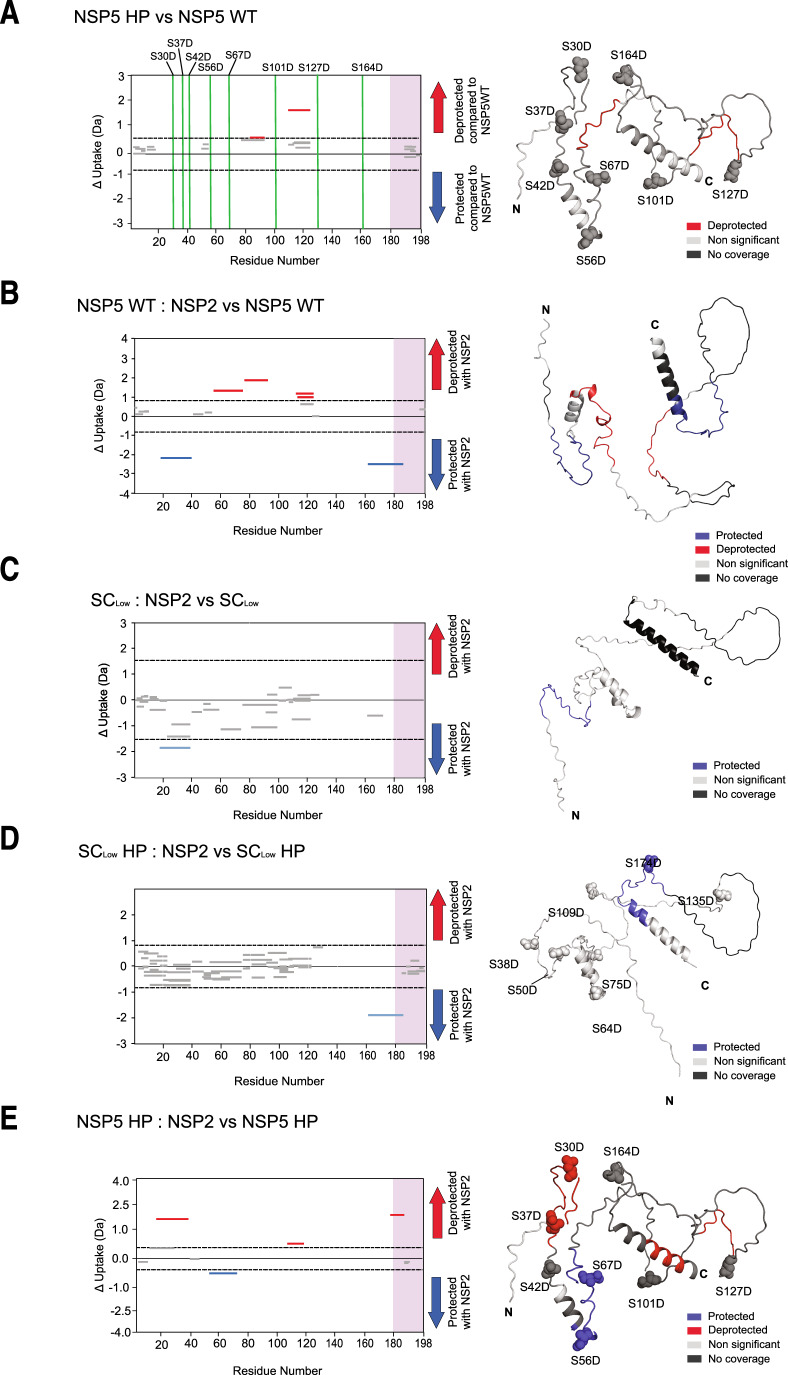
Distinct NSP5–NSP2 interaction interfaces revealed by HDX-MS upon introduction of phosphomimetic mutations in SA11 and SC_Low_ NSP5 variants. (**A**–**E**) Hydrogen–deuterium exchange mass spectrometry (HDX-MS) analysis of wild-type (WT) and hyperphosphorylation-mimetic (HP) NSP5, as well as SC_Low_ and SC_Low_ HP. Cumulative Woods plot comparing the extent of deuterium incorporation in NSP5 HP vs WT-NSP5 (**A**). Cumulative Woods plot comparing the extent of deuterium incorporation in NSP5 WT (**B**) and SC_Low_ (**C**), SC_Low_ HP (**D**) and NSP5 HP (**E**) in the absence/presence of NSP2. Protein regions showing protection (blue) or deprotection (red) are indicated based on the peptides with significant differences in deuterium uptake (determined using Deuteros with hybrid significance testing (*P* < 0.02; (Lau et al, 2019)). The C-terminal region (CTR) is marked by a purple box. AlphaFold2 models of monomeric NSP5 variants showing mapped HDX-MS data: protected regions (blue), deprotected regions (red), peptides with no significant change (light grey), and regions with no coverage (dark grey). S-to-D phosphomimetic mutations are indicated with green lines in Woods plots in (**A**) and are shown on the AlphaFold2 models in (**A**, **B**, **E**).

To map phosphorylation-dependent binding interfaces, we performed hydrogen–deuterium exchange mass spectrometry (HDX-MS) on WT-NSP5 (strain SA11) and its phosphomimetic variant in the presence of NSP2, to investigate how exposed (deprotected) or hidden (protected) NSP5’s amide hydrogens are in the presence or absence of a binding partner (Fig. 10) (Colyer et al, 2025). Comparison of deuterium uptake in NSP5 with NSP5 HP showed that phosphorylation induced deprotection in two regions of the IDR, suggesting altered conformational dynamics upon phosphorylation (Fig. 10A). In unmodified NSP5, condensation with NSP2 led to significant protection of both the CTR (residues 167–191) and an IDR region (residues 26–47), in agreement with previous reports of NSP2–NSP5 interaction sites (Eichwald et al, 2004b; Lee et al, 2024). In contrast, condensation of the NSP5 HP variant with NSP2 did not result in protection at these sites. Instead, a different region within the IDR (residues 53–73) became protected, suggesting that NSP5 uses distinct binding interfaces to interact with NSP2 depending on its phosphorylation state. Similarly to the wild-type NSP5, SC_Low_ shows protection in the IDR region (Fig. 10B,C), though we were unable to confirm the second protected region within the CTR (residues 167–191) observed in the WT variant but not in SC_Low_ likely due to poor peptide coverage (Fig. 10C). However, the same CTR region was protected in SC_Low_ HP (Fig. 10D), suggesting that the SC_Low_ CTR is a predominant NSP2 binding site, closely mirroring the WT-NSP5 binding mechanism of NSP2 (Fig. 10E).

## Discussion

Although many IDPs can spontaneously phase separate, others require post-translational modifications such as phosphorylation to facilitate condensation (Aumiller and Keating, 2016; Grams et al, 2024). Given the extensive sequence diversity across viral proteomes (Dyson, 2023; Mishra et al, 2020), we hypothesised that different viral variants might employ these mechanisms interchangeably depending on their amino acid composition of their IDPs.

We identified NSP5 sequences with markedly different condensate-forming abilities compared to the well-characterised rotavirus strain SA11. This allowed us to engineer a low-LLPS propensity NSP5 variant, SC_Low_, which retains conserved features of the SA11 sequence. SC_Low_ showed a significantly reduced DeePhase score achieved through combinatorial amino acid substitutions derived from four naturally occurring NSP5 variants with similarly low scores.

DeePhase accurately predicted LLPS potential: all four tested variants SA11, RF and SC_Low_ and S4_Low_ behaved as expected in vitro. Paradoxically, both SC_Low_ and a naturally occurring variant S4_Low_ fully supported viral replication, forming viroplasms in infected cells. While DeePhase and similar approaches are trained on proteins with known propensity to phase separate (Chu et al, 2022; Hatos et al, 2023; Saar et al, 2021), they do not reveal specific features or residues driving LLPS. Our combined use of in silico prediction and mutational analysis revealed that DeePhase is sensitive primarily to sequence differences within IDRs, but not to contributions from structured regions, which may still be critical for phase separation. Notably, introducing phosphomimetic substitutions at serines previously reported to be phosphorylated in infected cells restored SC_Low_’s ability to nucleate LLPS to levels comparable to the wild-type proteins. Consistently, in vitro phosphorylated SC_Low_ also formed condensates when combined with NSP2. In these assays, we used non-phosphorylated recombinantly expressed NSP2 because we initially found that this form is sufficient to drive LLPS. Although we did not directly test the functional role of the previously reported NSP2 phosphosite Ser313, in kinase-treated samples we detected phosphorylation at this residue in vitro (Fig. EV3). We also observed Ser232, Ser233, as well as Thr220 phosphosites in NSP2, which were also reported by Criglar et al (Criglar et al, 2014); however, the contribution of these sites to viroplasm formation and viral replication remains to be established.

**Figure 11:**
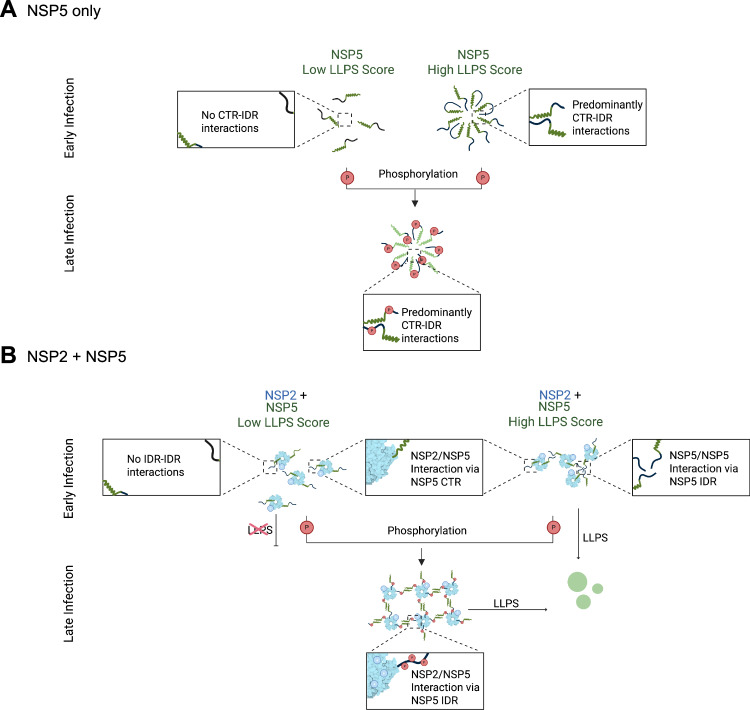
Phosphorylation-induced transition from non-saturating to saturating interactions in NSP5-NSP2 condensates. (**A**) Homotypic interactions of NSP5. NSP5 variants with low-LLPS propensity (black IDRs, green CTRs) self-associate poorly and do not efficiently form condensates. In contrast, variants with high LLPS propensity, as well as those with phosphorylated IDRs, readily undergo LLPS. In these cases, condensate formation is favoured by interactions between the IDRs and CTRs. Phosphorylation of the IDR enhances IDR–CTR interactions, thereby promoting homo-oligomerisation of low-propensity variants and condensate assembly. (**B**) Heterotypic interactions between NSP5 and NSP2. Early in infection, positively charged NSP2 octamers (light blue doughnuts) bind NSP5 via stoichiometric interactions with the CTR. For NSP5 variants with high LLPS propensity, the flexible IDRs additionally form a non-stoichiometric interaction network that drives phase separation. In contrast, low-LLPS NSP5 variants can still engage NSP2 via the CTR, but fail to phase separate because IDR-mediated interactions are insufficient. Late in infection, phosphorylation increases the net negative charge of the NSP5 IDR and enhances its interactions with NSP2. These phosphorylation-dependent contacts enable even low-LLPS NSP5 variants to undergo saturable binding and phase separation, supporting condensate assembly while largely retaining the predominant CTR–IDR interaction mode observed for non-phosphorylated WT-NSP5.

Previous studies identified the helical C-terminal region (CTR) of NSP5 as the key interface mediating its homo-oligomerisation (Martin et al, 2011; Sen et al, 2007; Torres-Vega et al, 2000). While coiled-coil interactions can be sufficient to drive phase separation in other systems (Ramirez et al, 2023; Ramšak et al, 2023), the inability of SC_low_ to undergo LLPS despite an intact CTR demonstrates that both the IDR and the CTR are required for phase separation. Indeed, deletion of the CTR abrogates formation of high molecular weight NSP5 species, while removal of the IDR results in aggregation of the CTR, indicating that both regions contribute to NSP5 oligomerisation, and that the IDR may contribute to solubilisation of the CTR (Fig. 11A). Importantly, oligomerisation of NSP5 appears to be dispensable for NSP5/NSP2 co-condensation, as the IDR HP alone can phase separate with NSP2. However, oligomerisation significantly reduces C_sat_, suggesting that interactions promoting NSP5 self-assembly are distinct from those driving its co-condensation with NSP2 (Choi et al, 2020). This implies that sequence features within the IDR not only prevent CTR aggregation but also modulate the cooperative assembly of NSP5 oligomers and NSP5 condensates.

We propose a model in which unphosphorylated NSP5 binds NSP2 primarily via the CTR, forming a hub–driver architecture (Galagedera et al, 2023), while the remaining IDRs mediate weak, non-stoichiometric interactions supporting condensate formation (Fig. 11A). This is consistent with our observation that the non-phosphorylated IDR alone does not engage NSP2, indicating that CTR-mediated interactions dominate in this state.

Upon phosphorylation, the IDR acquires a negative charge and engages NSP2 more directly, shifting NSP5 towards a distinct interaction mode (Fig. 11B). In this state, NSP5–NSP2 interactions become more specific and saturable, consistent with the re-entrant phase behaviour observed for phosphomimetic variants. This transition represents an allosteric switch from weak, non-saturating multivalent interactions to more specific, saturable ones.

Low-propensity NSP5 variants appear to undergo this transition incompletely, which may explain their smaller condensate phenotype. In this context, phosphorylation-dependent modulation of competing interaction modes provides a mechanism by which condensate architecture can be tuned without abolishing function. These findings align with theoretical models proposing that condensate function is governed by a balance of weak, competing interactions (Bhandari et al, 2021; Schmit et al, 2021). Our data highlight how phosphorylation dynamically regulates networks of weak, competing interactions within viral biomolecular condensates, thereby tuning NSP5-NSP2 interactions and condensate architecture.

In a cellular context, we propose that this phosphorylation-dependent switching between condensation mechanisms is critical for different stages of the rotavirus life cycle, enabling viroplasms to transition from early recruitment of replication components to later assembly steps. Although multiple serines in NSP5 have been identified as phosphorylated (Sotelo et al, 2010), the minimum number required to trigger an allosteric switch remains unknown.

Multi-site phosphorylation of intrinsically disordered proteins is often associated with threshold-like behaviour, where a critical number of phosphorylation events is required to enable functional switching. For example, the IDP Sic1 exhibits a dose-dependent response, requiring phosphorylation at multiple sites to engage its binding partner (Borg et al, 2007). Similarly, graded phosphorylation of ETS-1 acts as a rheostat to fine-tune DNA binding (Pufall et al, 2005). Consistent with such a mechanism, although S67D substitution has been shown to restore NSP5 hyperphosphorylation in the SA11 strain (Eichwald et al, 2004a), a single S67D substitution was insufficient to restore phase separation in SC_Low_ (Fig. EV3B), whereas CKII-mediated multi-site phosphorylation enabled condensate formation. These observations support a threshold-like model for NSP5 regulation.

Hyperphosphorylated NSP5 accumulates progressively during infection and is largely absent at early stages of infection (Blackhall et al, 1998; Campagna et al, 2007; Criglar et al, 2018; Eichwald et al, 2004a). This suggests that phosphorylation-dependent regulation becomes increasingly important as viral replication progresses and the concentration of NSP5, NSP2, and other viral components rises. In the SA11 strain, which has high intrinsic LLPS propensity, NSP5 phosphorylation is not required for in vitro condensate formation or early-stage viroplasm assembly (Papa et al, 2019). In contrast, SC_Low_, which lacks sufficient IDR-mediated interactions, cannot initiate condensate formation without phosphorylation. Thus, in low-LLPS variants, hyperphosphorylation acts as a prerequisite for condensate nucleation. Despite sequence divergence, phosphorylation enables these variants to form condensates, indicating a conserved regulatory strategy across rotavirus strains.

Together, our results support a model in which NSP5 condensate behaviour is governed by a balance between competing interaction modes mediated by the CTR and the IDR. In high-LLPS variants, condensate formation can be driven by intrinsic IDR-mediated interactions, whereas in low-propensity variants, phosphorylation is required to reach the threshold for phase separation. Beyond enabling condensate formation, phosphorylation also modulates how NSP5 engages NSP2, shifting the system from CTR-dominated interactions to a more distributed interaction network involving the IDR.

Our domain titration experiments further reveal that these interaction modes differ not only in strength but also in their impact on condensate stability. CTR-mediated interactions are potent but disruptive when unbalanced, whereas IDR-mediated interactions are weaker and more readily accommodated within the condensate network. Phosphorylation reduces the dominance of CTR-mediated interactions and redistributes interaction valency across the NSP5 molecule, resulting in a more robust and adaptable condensate architecture. In this way, phosphorylation acts not simply as a trigger for phase separation, but as a mechanism for tuning the internal organisation and material properties of viral condensates.

More broadly, these findings highlight a general principle for intrinsically disordered proteins: post-translational modifications can rewire interaction networks by shifting the balance between competing interaction modes. Such regulation enables access to distinct functional states without requiring large-scale structural changes. In the context of viral replication, this provides a mechanism by which condensates can transition between stages of assembly and activity, while remaining responsive to changes in protein concentration and modification state.

The ability of NSP5 to accommodate substantial sequence variation within its IDR, while maintaining function through phosphorylation-dependent regulation, suggests a flexible evolutionary strategy. By modulating the number and distribution of phosphorylation sites, viral proteins may compensate for mutations that weaken intrinsic phase separation propensity, ensuring robust condensate formation across diverse strains and host environments.

Finally, these findings point to intrinsically disordered regions as promising targets for antiviral intervention. Disrupting the balance between competing interaction modes, for example, by perturbing phosphorylation-dependent interactions or altering IDR-mediated binding, may provide a means to selectively interfere with condensate assembly and function (Krishnan et al, 2014). Our findings establish phosphorylation-dependent rewiring of IDR interactions as a tractable strategy to control biomolecular condensates in both viral and cellular systems.

## Methods

**Table:** Reagents and tools table

Reagent/resource	Reference or source	Identifier or catalogue number
**Experimental models**		
African green monkey kidney MA104 cells	ATCC	CRL-2378.1
MA104 stably expressing NSP5-eGFP (NSP5-eGFP-MA104)	Papa et al, 2019	10.1128/jvi.01110-19
Baby hamster kidney fibroblasts expressing T7 polymerase (BHK-T7)	Diebold et al, 2021	10.1128/jvi.00488-22
*E. coli* BL21(DE3)	NEB	C2527H
**Recombinant DNA**		
pT7-based RV-SA11 genome segments 1–11	provided by Dr. Takeshi Kobayashi, AddGene	89162 - 89172
pT7/SClow plasmid	This study, AddGene	220829
pT7/SClow S67A plasmid	This study	N/A
pCDNA NSP5	Papa et al, 2019	N/A
pCDNA NSP2	Papa et al, 2019	N/A
pET28 N-strep NSP5 SA11	This study	N/A
pET22 N-strep NSP5 SA11 HP	This study	N/A
pET28 N-strep NSP5 RF	This study	N/A
pET22 N-strep NSP5 RF HP	This study	N/A
p N-strep NSP5 RF IDR	This study	N/A
p N-strep NSP5 RF IDR HP	This study	N/A
pET28 NSP5 S4Low	This study	N/A
pET28 NSP5 S4Low HP	This study	N/A
NSP5 SCLow	This study	N/A
NSP5 SCLow HP	This study	N/A
pET28 NSP2 RF	Borodavka et al, 2017	N/A
pET22 NSP2 SA11	This study	N/A
**Antibodies**		
Guinea pig anti-NSP5	Papa et al, 2019	N/A
Goat anti-guinea pig IgG (H + L), DyLight 550	Invitrogen	SA5-10095
Goat anti-guinea pig IgG (H + L), DyLight 800	Invitrogen	SA5-10100
Anti-actin hFAB rhodamine antibody	Bio-Rad	12004163
**Oligonucleotides and other sequence-based reagents**		
FISH probes	Strauss et al, 2023	10.7554/eLife.68670
Sequencing primers	IDT, this study	Methods (Virus propagation)
**Chemicals, enzymes and other reagents**		
Acetonitrile	Macron	2856-25
Alexa Fluor™ 647 NHS	Thermo Scientific	A20006
ATP	Thermo Scientific	R0441
Atto NTA-Atto-488	Merck	39625
Avicel®	Sigma-Aldrich	435244
BDMA	TAAB	B036
Casein Kinase II (CK2)	NEB	P6010S
CaCl2 x 2 H2O	Sigma-Aldrich	C7902
cOmplete protease inhibitor	Roche	11873580001
DAPI	Thermo Fisher	62248
Desthiobiotin	IBA	2-1000-002
DMEM	Gibco	
DyLight 488 Maleimide dye	Thermo Fisher	46602
Ethanol	Merck	32221
Formaldehyde	Merck	F8775
Formic acid		
Glutaraldehyde	TAAB	G017
Glycine	Sigma-Aldrich	3570
Laemmli buffer	Bio-Rad	1610747
Maleic acid	Merck	8.00380.0500
MOPS	Melford	M92020
MNA	TAAB	M010
NSA	TAAB	N007
Phosphate-buffered saline	Oxoid	
Potassium ferricyanide	AnalaR	0153090
Nitrocellulose membrane	Milipore	
Osmium tetroxide	TAAB	O001/D/10
Paraformaldehyde	Merck	8.18715.1000
phosSTOP	Merck	4906845001
Quetol 651	TAAB	Q001
Sodium cacodylate trihydrate	Sigma-Aldrich	20840
Sodium dodecyl sulfate	Melford	L22010
TCEP	Sigma-Aldrich	C4706
Thiocarbohydrazide	Merck	223220
Triton X-100		
Tris-HCl		
Uranyl acetate	AnalaR	10288
**Software**		
AutoCAD		
Clustal 2.1	Madeira et al, 2022	
DeePhase	https://deephase.ch.cam.ac.uk/	
Deuteros	Lau et al, 2019	
DynamX	Waters Corporation	
DiscoveryMP	Refeyn	
GraphPad Prism v10	https://www.graphpad.com	
Fiji	Schindelin et al, 2012	
UCSF ChimeraX	Meng et al, 2023	
**Other**		
Amicon Spin column 3 K	Millipore	UFC900308
C18 column	Waters Corporation	
Corning cover glass	Merck	CLS2980245
Immobilised pepsin column	Waters Corporation	
Low Auto Fluorescence Immersion Oil	Olympus	
(PDMS)-on-glass devices	Arosio et al, 2016; Qin et al, 2010; Saar et al, 2017	
Slide-A-Lyze MINI Dialysis Device 3.5 K	Thermo Fisher Scientific	
Strep-tactin superflow column	Iba lifescience	
TEM grids	EM Resolutions	
VanGuard Pre-Column	Waters Corporation	
µ-slide 8-well microscope slide (high glass bottom)	Ibidi	
6-well silicon sample cassette	Refeyn	

### NSP5 sequences

A dataset comprising 89,417 *Rotavirus A* protein sequences was obtained from the NCBI Virus database (Taxonomy ID: 28875). FASTA headers were reformatted to include relevant metadata (Accession, GenBank Title, Species, Length, Segment, and Protein designation), which facilitated filtering for full-length NSP5 sequences. A custom Python script (described below) was used to exclude entries marked as “truncated” or “partial,” as well as sequences containing ambiguous amino acid codes: “B” (aspartic acid or asparagine), “Z” (glutamic acid or glutamine), “J” (leucine or isoleucine), and “X” (unspecified residues). Sequences annotated as “NSP5” or its variants were retained. After removing duplicates, a curated set of 451 unique full-length NSP5 sequences was obtained.

### In silico LLPS propensity analysis using DeePhase

LLPS propensities of NSP5 sequences were assessed using DeePhase (Saar et al, 2021). DeePhase employs two independent models: the first relies on sequence-derived biophysical features, including hydrophobicity, Shannon entropy, sequence length, the proportion of polar, aromatic, and positively charged residues, as well as the content of low-complexity and intrinsically disordered regions. The second model is based on word2vec embeddings (Mikolov et al, 2013b, 2013a), a natural language processing algorithm adapted to capture sequence patterns. Both models were trained on the publicly available LLPSDB (Li et al, 2020) and PDB (Berman et al, 2000) datasets. Predictions were performed for a curated set of 451 unique full-length NSP5 sequences, including those from the SA11 and RF strains (NCBI accession IDs BAW94621.1 and AHF49898.1), 128 permutation variants, and the engineered low-propensity sequence, SC_Low_. Each sequence was evaluated by both prediction models, and the final LLPS propensity score was calculated as the average of the biophysical and word2vec-based scores, following the authors’ recommendations (Appendix Table S1).

### Sequence distance calculation

Pairwise Levenshtein distances (Levenshtein, 1966) were computed between the SA11 NSP5 sequence (NCBI accession ID: BAW94621.1) and all NSP5 variants with a DeePhase propensity score below 0.5 (S1_Low_…S7_Low_; NCBI accession IDs QIN53369.1, QIN53336.1, ACC91692.1, BBB18699.1, QIJ58158.1, AGF92026.1, AKA40124.1). Distances were calculated using a custom Python script (v3.9.5) with the Python-Levenshtein package (v0.21.1) and plotted with matplotlib (v3.4.2). All custom scripts have been deposited and are available at https://github.com/desiro/SClow.

### Consensus sequence generation

Multiple sequence alignments were performed using MAFFT (v7.480) (Katoh and Standley 2013) with default parameters. The alignments were imported into Unipro UGENE (v42.0) (Golosova et al, 2014; Okonechnikov et al, 2012; Rose et al, 2019) to create consensus sequences. A consensus sequence was generated for (i) the four sequences from the SA11-like cluster (SC; S4_Low_, S5_Low_, S6_Low,_ and S7_Low_) and (ii) the subset of 78 SC_128_ permutation sequences with DeePhase liquid–liquid phase separation propensity scores below 0.5 (Saar et al, 2021).

### Phylogenetic tree construction

To construct the phylogenetic tree, all 451 unique rotavirus A NSP5 sequences retained after filtering, along with the 128 permutation sequences and the engineered SC_Low_ sequence (GenBank accession no. PP828582), were aligned using MAFFT (v7.480) (Katoh and Standley 2013) with default parameters. The resulting alignment was subjected to maximum likelihood phylogenetic analysis using PhyML 3.0 (v3.3.20200621) (Guindon et al, 2010) with the command-line options “--datatype aa --no_memory_check --leave_duplicates”. The phylogenetic tree was midpoint-rooted (Swofford et al, 1996) and visualised using the Interactive Tree of Life (iTOL v6) (Letunic and Bork, 2021).

### Genomic RNA sequence creation

The RNA sequence encoding SC_Low_ was generated using a custom Python script (v3.9.5; Python Software Foundation, 2001), with the SA11 NSP5 RNA sequence serving as the reference (see Fig. EV2). To ensure compatibility with the SA11 rotavirus genome, the algorithm preserved both 5′ and 3′ untranslated regions (UTRs) of gene segment 11, modifying only the NSP5 coding region through synonymous codon substitutions. For codons where the encoded amino acid remained unchanged, the nucleotide sequence from the SA11 reference was retained. For codons corresponding to amino acid substitutions, synonymous codons were selected based on minimal evolutionary distance to the reference codon, using the K3ST model (Kimura, 1981) based on the generalised time-reversible model (Tavaré et al, 2000). The K3ST parameters were set to *α* = 1.4, *β* = 1.24, and *γ* = 1.36.

### Cells and viruses

African green monkey kidney MA104 cells (ATCC CRL-2378.1) were used for virus propagation. For imaging experiments, a derivative MA104 cell line stably expressing NSP5-eGFP (NSP5-eGFP-MA104) was used (Papa et al, 2019). Both wild-type MA104 and NSP5-eGFP-MA104 cells were cultured as monolayers in Dulbecco’s Modified Eagle Medium (DMEM, Gibco), supplemented with 10% foetal bovine serum (FBS; Sigma-Aldrich), 1% penicillin–streptomycin (Gibco), 1% GlutaMAX (Gibco), and 1% non-essential amino acids (NEAA; Gibco). Baby hamster kidney fibroblasts expressing T7 polymerase (BHK-T7) were used for reverse genetics of SA11 RV, as described in Diebold et al (Diebold et al, 2021). These cells were maintained in Glasgow’s Minimum Essential Medium (GMEM; Sigma) supplemented with 5% FBS, 10% tryptose phosphate broth (TPB; Sigma-Aldrich), 1% NEAA, 1% GlutaMAX, and 1% penicillin–streptomycin. All cell lines were maintained at 37 °C in a humidified incubator with 5% CO₂.

### Plasmids

Plasmids encoding the complete genome of RV-SA11 (segments 1–11) were kindly provided by Dr. Takeshi Kobayashi. To generate the pT7/SC_Low_ plasmid, a gBlock (Integrated DNA Technologies) encoding the SC_Low_ open reading frame (ORF), flanked by a 5′ *AfeI* site and a 3′ *SacI* site, was synthesised and cloned into the pT7/NSP5 vector, replacing the wild-type *NSP5* ORF. To generate the pT7/S4_Low_ plasmid, a gBlock (Integrated DNA Technologies) encoding the ORF of the NCBI ID sequence BBB18699.1, was synthesised and cloned into the pT7/NSP5 vector, replacing the wild-type NSP5 ORF. The sequences of the pT7/SC_Low_ and pT7/S4_Low_ plasmids were confirmed by Sanger sequencing. Plasmids for transfection were prepared using a DNA Maxi Prep Kit (Qiagen) according to the manufacturer’s instructions, followed by ammonium acetate precipitation to obtain a final concentration exceeding 1 µg/µl.

### Reverse genetics and virus rescue

BHK-T7 cells were seeded in 12-well plates to reach ~70% confluency the following day. Twenty-four hours later, a plasmid mixture was prepared in 250 µl pre-warmed Opti-MEM (Gibco), containing 0.8 µg each of pT7/VP1, VP2, VP3, VP4, VP6, VP7, NSP1, NSP3, and NSP4, 2.4 µg each of pT7/NSP2 and pT7/SC_Low_ or pT7/S4_Low_, and 0.8 µg each of pcDNA3-NSP2 and pcDNA3-NSP5. To this, 34 µl TransIT-LT1 transfection reagent (Mirus Bio; Geneflow) was added. The mixture was incubated for 18 min at 25 °C. BHK-T7 cell monolayers were washed with Eagle’s Minimum Essential Medium (Joklik modification, Sigma-Aldrich) supplemented with 1% GlutaMAX. The transfection mixture was then added dropwise, and cells were incubated at 37 °C for 48 h.

After 48 h, confluent MA104 cells were harvested using trypsin-EDTA (Sigma-Aldrich), resuspended in DMEM containing 1 µg/ml trypsin, 1% GlutaMAX, and 1% NEAA, and overlaid onto the transfected BHK-T7 cells. Co-cultures were incubated at 37 °C for 4 days. On day 7, cells were subjected to three freeze–thaw cycles to release virus particles. Cell lysates were clarified by centrifugation at 10,000×*g* for 5 min, and the supernatants were treated with 2 µg/ml porcine trypsin at 37 °C for 30 min. Trypsin-activated virus preparations were used to infect fresh monolayers of MA104 cells in FBS-free DMEM. Cells were incubated at 37 °C until ~80% cytopathic effect (CPE) was observed, at which point virus was harvested.

### Virus propagation

For propagation, confluent MA104 cells were infected at an MOI of 0.01 using FBS-free DMEM supplemented with 0.5 µg/ml trypsin. Following infection, cells were lysed by three freeze–thaw cycles, and lysates were clarified by centrifugation at 10,000×g for 5 min. For each passage, virus-containing supernatants were activated by incubation with 1 µg/ml trypsin at 37 °C for 30 min prior to use.

To verify recombinant virus sequences, viral RNA was extracted from infected MA104 cells using the RNeasy Mini Kit (Qiagen), followed by reverse transcription with the SuperScript III Reverse Transcriptase Kit (Invitrogen) using the gs11 primers (GGCTTTTAAAGCGCTACAGTGATG and GGAGCTCCCTAGTGTGTTCCCAGG). Amplified cDNA was generated using OneTaq Quick-Load 2X Master Mix (New England Biolabs) and analysed by Sanger sequencing.

### Determination of viral titres

Plaque assays were performed to quantify viral titres. MA104 cells seeded in six-well plates were washed with FBS-free complete DMEM 30 min prior to infection. Serial tenfold dilutions of virus stocks were prepared in FBS-free medium and added to cell monolayers. Following a 2-h incubation at 37 °C, the inoculum was removed, and cells were overlaid with 1.2% Avicel (Sigma-Aldrich) in FBS-free DMEM containing 0.5 µg/ml trypsin. Plates were incubated at 37 °C for 4 days. To fix the cells, the overlay was removed and replaced with 10% formaldehyde. Plates were incubated at 37 °C for 1 h, then stained with Coomassie Brilliant Blue for 1 h at 37 °C. Plaques were counted, and viral titres were calculated using the equation:

$${Viral\; titre}=\frac{N}{V\times {DF}}$$

where *N* is the number of plaques, *V* is the volume of inoculum (ml), and *DF* is the dilution factor.

For TCID₅₀ assays, MA104 cells were seeded in 48-well plates (Sigma). Virus samples were serially diluted tenfold in serum-free DMEM. Each dilution was added to eight replicate wells. After a 96-h incubation at 37 °C, wells were examined for cytopathic effect (CPE). TCID₅₀ values were calculated using the Reed–Muench method (Reed and Muench 1938) and expressed in plaque-forming units (PFU) per millilitre using the standard conversion (Pourianfar et al, 2012): PFU/ml = 0.7 × TCID₅₀/ml.

### Characterisation of recombinant viruses

#### Replication kinetics

Confluent MA104 cells (African green monkey kidney; ATCC CRL-2378.1) were infected in duplicate with either SA11-RV or SC_Low_-RV at a multiplicity of infection (MOI) of 1. After a 60-min adsorption at 37 °C, the inoculum was removed, cells were washed once with 2 mM EGTA in PBS, and fresh serum-free DMEM containing 0.5 µg ml⁻¹ trypsin (Worthington) was added. Supernatants and cell lysates were harvested at 0, 8, 16, 24, 48 and 72 h post-infection (h.p.i.). Virus was released by three freeze–thaw cycles (–80 °C/37 °C), clarified by centrifugation (16,000×*g*, 5 min), and the resulting supernatant was activated with trypsin (1 µg ml⁻¹, 30 min, 37 °C). Infectious titres were quantified in three independent plaque assays and expressed as mean plaque-forming units per millilitre ±SD.

### Genetic stability

To assess serial-passage stability, MA104 cells were infected with SC_Low_-RV at MOI = 0.1 and passaged ten times (P1–P10). At complete cytopathic effect, cell culture medium was collected, clarified (16,000×*g*, 5 min), trypsin-activated as above, and titrated. Virus harvested from each passage served as inoculum for the next passage.

### Western blotting

RIPA buffer (150 mM NaCl, 1% Triton X-100, 0.1% sodium dodecyl sulphate, Tris-HCl pH 8, phosSTOP and cOmplete protease inhibitor cocktail (Roche)) was used to lyse RV-infected cells (MOI = 20) at 4, 6, and 8 h.p.i. Cell lysates were harvested and centrifuged at 15,000 rpm at 4 °C for 10 min. Supernatants were mixed with an equal amount of 2× Laemmli buffer (Bio-Rad) and heat-denatured at 98 °C for 10 min. Proteins were resolved by electrophoresis on 15% SDS polyacrylamide gels and transferred onto a nitrocellulose membrane (Millipore, Bedford, MA) using the Bio-Rad Trans-Blot Turbo Transfer System. Blots were then blocked with 5% (w/v) milk (MARVEL) dissolved in phosphate-buffered saline (PBS) buffer pH 7.4 (Oxoid). The membrane was incubated with primary antibodies (guinea pig anti-NSP5 (Papa et al, 2019) diluted 1:2500 in 5% (w/v) milk in PBS. The blots were next washed with 0.5% Tween-20 (Sigma-Aldrich) dissolved in PBS before incubation with anti-guinea pig IgG (H + L) cross-adsorbed secondary antibody DyLight 800 (1:10,000; Invitrogen) and anti-actin hFAB rhodamine antibody (1:2500; Bio-Rad) in 5% milk (w/v) in PBS. The Bio-Rad ChemiDoc MP imaging system was used for fluorescent signal detection (DyLight800/Rhodamine filters). The band intensities of viral NSP5 were measured in Image Lab (version 6.1.0) and normalised to beta-actin band intensities and NSP5-specific antibody sensitivity toward WT-NSP5 and SC_Low_ (400 ng WT-NSP5 band volume * 0.6687 = 400 ng SC_Low_ band volume; simple linear regression). Statistical analysis was conducted using GraphPad Prism v10.

### Sample processing for transmission electron microscopy (TEM) imaging

MA104 cells were cultured in 35 mm ø Ibidi dishes with plastic coverslips (Ibidi-treat), and specimen processing for EM was carried out in these culture dishes. Confluent cells were infected in FBS-free medium with WT- or SC_Low_-RVs at MOI = 10. Samples were fixed at 8 h.p.i. in fixative (2% glutaraldehyde (TAAB) and 2% formaldehyde (Merck) in 0.05 M sodium cacodylate buffer pH 7.4 (Sigma), containing 2 mM calcium chloride (Sigma)) overnight at 4 °C. After washing 5× with 0.05 M sodium cacodylate buffer pH 7.4, samples were osmicated (1% osmium tetroxide (TAAB), 1.5% potassium ferricyanide (AnalaR), 0.05 M sodium cacodylate buffer pH 7.4) for 3 days at 4 °C. After washing 5× in DIW (deionised water), samples were treated with 0.1% (w/v) thiocarbohydrazide (Merck) in DIW for 20 min at room temperature in the dark. After washing 5× in DIW, samples were osmicated a second time for 1 h at RT (2% osmium tetroxide/DIW). After washing 5× in DIW, samples were stained with uranyl acetate (2% uranyl acetate (Merck) in 0.05 M maleate buffer pH 5.5 (Merck)) for 3 days at 4 °C. Samples were washed 5x in DIW and then dehydrated in a graded series of ethanol (50%/70%/95%/100%/100% dry) (Merck) and 100% dry acetonitrile (Macron), 3× in each for at least 5 min. Samples were infiltrated with a 50/50 mixture of 100% dry acetonitrile/Quetol (TAAB) resin (without BDMA) overnight, followed by 3 days in 100% Quetol (without BDMA). Then, the sample was infiltrated for 5 days in 100% Quetol resin with BDMA (TAAB), exchanging the resin each day. The Quetol resin mixture is: 12 g Quetol 651, 15.7 g NSA (TAAB), 5.7 g MNA (TAAB) and 0.5 g BDMA (TAAB). The Ibidi dishes were filled with resin to the rim, covered with a sheet of Aclar and cured at 60 °C for 3 days. After curing, the Aclar sheets were removed, and small sample blocks were cut from the Ibidi dish. Thin sections (~ 70 nm) were prepared using an ultramicrotome (Leica Ultracut E). Resin blocks were orientated with the cell-side towards the knife, and sections were collected on bare 300 mesh copper grids (EM Resolutions) immediately when reaching the cell monolayer. Samples were imaged in a Tecnai G2 TEM (FEI/Thermo Fisher Scientific) run at 200 keV using a 20-µm objective aperture. Images were acquired using an ORCA HR high-resolution CCD camera (Advanced Microscopy Techniques Corp, Danvers, USA).

### Condensate and cell imaging

Cells were seeded in an µ-slide 8-well microscope slide (high glass bottom, Ibidi). At 90–100% confluency, cells were infected with RV. For immunofluorescence, cells were fixed with 4% Paraformaldehyde (PFA), permeabilised with 100 mM glycine (Sigma-Aldrich) and 0.2% Triton X-100 consecutively. NSP5 was labelled with primary antibody GP anti-NSP5 (1:500), followed by secondary antibody goat anti-GP IgG (H + L) DyLight 550 (1:1000; Invitrogen). Cells were then stained with 300 nM 4’,6-diamidino-2-phenylindole (DAPI) for nucleus visualisation and imaged using the ONI Nanoimager S microscope with a Olympus 100x super apochromatic oil immersion objective (NA 1.4). DAPI was imaged using a 405 nm laser at 19% power, while the NSP5-eGFP was detected with a 488 nm laser at 1% intensity. The fluorescence images of both live cells and fixed cells were acquired using a sCMOS camera (Andor Technology) with a pixel size of 0.117 µm. For quantification, automatic particle counting of viroplasms with sizes ranging from 0 to 10 µm^2^ (Yen) and nuclei with sizes ranging from 50 to 600 µm^2^ (Default) was performed in Fiji (Schindelin et al, 2012). Statistical analysis was performed using the GraphPad software (Prism v10).

In vitro condensates were imaged using the ONI Nanoimager. Before imaging, samples were diluted in PBS pH 7.4 (Merck KGaA). NSP2 was mixed with 0.5 µM NTA-Atto-488 (Merck KGaA) before the addition of an NSP5 construct (either NSP5 SA11 nStrep, NSP5-HP SA11 nStrep, SC_Low_ nStrep or SC_Low_ HP nStrep, S4_Low_ and S4_Low_ HP, NSP5 IDR, IDR-HP or the NSP5 CTR) at an equimolar concentration to NSP2, to a working concentration of 25 µM per protein. In total, 6 µL of this mixture was mounted on a Corning cover glass (CLS2980245; Merck KGaA, Darmstadt, Germany) prior to imaging using a 100x oil objective (Olympus 1.4NA 100x oil immersion super apochromatic).

Phosphorylation assays for SC_Low_ nStrep were conducted by the addition of 0.5 mM ATP, 0.5× Kinase buffer (25 mM Tris-HCl, 5 mM MgCl_2_, 50 µM EGTA, 1 mM DTT, 0.005% Brij 35, and the presence or absence of 1 µL of casein kinase II (CKII, 2000 U/µL, NEB) to equimolar concentrations of NSP2 and SC_Low_ (25 µM per protein). Samples were incubated for 30 min at 30 °C and then tested for condensate formation directly, retaining 10 µL of the mixture for phosphorylation analysis by liquid chromatography-mass spectrometry. Images were acquired by scanning three ROIs of 500 µm × 800 µm. Representative images were prepared using Fiji (Schindelin et al, 2012). Yen’s thresholding technique (Jui-Cheng Yen et al, 1995) allowed for subsequent automated particle analysis. Fluorescence images were processed and prepared in Fiji/ImageJ. Unless otherwise stated, images are shown as 16-bit intensity data displayed using a fixed linear display range across compared panels within each experiment/figure. For pseudocolour display, the display lookup table (LUT) used, and the quantitative mapping between the LUT and the underlying pixel intensities (display minimum and maximum) are reported in the relevant figure legends. Values below the stated display minimum were rendered as the minimum LUT colour, and values above the display maximum were rendered as the maximum LUT colour (saturated). No non-linear intensity transforms (e.g., gamma adjustment, histogram equalisation) were applied. If individual channel adjustments were applied to merged images, this is indicated in the corresponding legend. For transparency and reproducibility, the LUT(s) and fixed display ranges used for each figure are reported below and/or provided as Source Data, together with representative intensity histograms where relevant. GraphPad Prism version 9.3.1 was used for the creation of graphs and statistical analysis.

### Single-molecule fluorescence in situ hybridisation (smFISH)

MA105 WT cells or MA104 NSP5 were seeded in an µ-slide 8-well microscope slide (high glass bottom, Ibidi). At 90–100% confluency, cells were infected with SC_Low_-RV. For both cell lines, a mock-infected control was included. Cells were fixed at 8 h.p.i. with 4% (v/v) methanol-free paraformaldehyde in nuclease-free PBS for 10 min. smFISH was conducted as described in (Strauss et al, 2023). RNA FISH probes are listed in (Strauss et al, 2023). Subsequent imaging of cells infected with SC_Low_ RV was performed as described above. Imaging of cells infected with SC_Low_ S67A RV was performed on a custom-built wide-field microscope. The microscope frame (IX83, Olympus) is equipped with an LED light source (DC4100, Thorlabs) and a scientific complementary metal-oxide semiconductor camera (Zyla 4.2, Andor) controlled with the software Micro-Manager (Open Imaging). All images were acquired with a 60×/1.42 oil objective lens (PlanApoU, Olympus). Image analysis was performed as described above.

### Expression and purification of recombinant proteins

Recombinant NSP2 cHis (strain SA11 and strain RF) was expressed and purified as described previously (Schuck et al, 2001). Protein sequences were ordered as gBlocks from Integrated DNA Technologies, carrying restriction sites for *Xba*I at the 5-prime end and for *Xho*I at the 3-prime end. Using restriction enzymes and T4 DNA ligase obtained from New England Biolabs, these gene fragments were cloned into a pET22 vector for subsequent expression in BL21(DE3) cells (New England Biolabs). NSP5 HP and SC_Low_ HP carry S to D mutations at the following positions: 30, 37, 42, 56, 67, 101, 127 and 164. Expression and inclusion body isolation were conducted as described in (Borodavka et al, 2017), inclusion body wash, solubilisation and dialysis as in (Martin et al, 2011). After dialysis, cleared supernatant was loaded onto a pre-equilibrated Strep-tactin superflow column (iba lifescience), and washed with 20 mM MOPS pH 7.1, 1 M NaCl for 20 column volumes (CV). Protein was eluted using 20 mM MOPS pH 7.1, 1 M NaCl, 2.5 mM Desthiobiotin for 5 CV. Protein-containing fractions were concentrated, and buffer was exchanged to 20 mM MOPS pH 7.1, 150 mM NaCl using an Amicon Spin column (Millipore) with a molecular weight cut-off of 3 K. Protein concentrations were estimated spectrophotometrically using an extinction coefficient for NSP5 (HP) of 10,555 and SC_Low_ (HP) of 19,160. The CTR peptide (YILDDSDSDDGKCKNCKYKRKYFALRMRMKQVAMQLIEDL) was obtained from the GenScript Biotech.

### Fluorescent labelling of recombinant proteins

NSP5 IDR and IDR HP were prepared for fluorescent labelling by incubating both protein samples in 10 mM tris(2-carboxyethyl)phosphine (TCEP) for 30 min at room temperature. TCEP concentration was reduced to 500 μM in 50 mM Tris-HCl pH 8.5 and 150 mM NaCl by using an Amicon Spin column (Millipore) with a molecular weight cut-off of 3 K, and the samples were concentrated to a final volume of 250 μl each. A DyLight 488 Maleimide dye (Thermo Fisher Scientific) concentration of 1.25 mM was adjusted per sample. The labelling reaction was incubated for an hour at room temperature, protected from light. After labelling, free dye was removed using a Slide-A-Lyze MINI Dialysis Device with a molecular weight cut-off of 3.5 K (Thermo Fisher Scientific). Dialysis was performed against PBS pH 7.4 (Oxoid). Protein concentrations were assessed spectrophotometrically using the molar extinction coefficient for the IDR HP of 10,555 and a correction factor of 0.147 for DyLight 488 dye. Molar protein concentration (C) was determined using the equation: ${{\rm{C}}}=\frac{({A}_{{protein}-}\,-{A}_{{dye}}x\,0.147)}{{\mathrm{10,555}}}$.

### Dynamic light scattering (DLS)

A BeNano 180 Zeta Max (Bettersize Instruments) was used to perform dynamic light scattering (DLS) measurements on the NSP5 CTR peptide. Before measurement, the sample was centrifuged at 16,000 rpm for 10 min and diluted in sterile-filtered PBS at approximately pH 7.4 (Merck). A 70 µl aliquot of the sample, adjusted to a final concentration of 1 µM, was transferred into a Brand microcuvette (Merck). Autocorrelation functions were combined by averaging. GraphPad Prism version 9.3.1 was used to produce the graphical outputs.

### Mass photometry

Mass photometry measurements were conducted using a Refeyn Two^MP^ mass photometer and the Acquire^MP^ software (Refeyn Ltd, Oxford, UK, v 2023 R1.1) at 21 °C at a 50 Hz frame rate and a 10.9 μm × 4.3 μm instrument field of view (46.3 μm^2^ detection area). Before 3 movies (*n* ≥ 3) of 60 s each were recorded per sample, protein stock was diluted in sterile-filtered PBS at pH 7.4 (806552-1 L; Merck KGaA, Darmstadt, Germany). A ready-to-use sample carrier slide (Refeyn Ltd., Oxford, UK) was used in combination with a 6-well silicon sample cassette (Refeyn Ltd., Oxford, UK) for identification of the focal position (using 18 µl of buffer) and movie acquisition (by spiking the buffer with 2 µl of sample). The final sample volume was 20 µl with a working concentration of 100 nM. BSA, IgG and thyroglobulin were used for contrast-to-mass calibration. The Discovery^MP^ software (Refeyn Ltd, Oxford, UK, v 2023 R1.2) was used for data processing, with a bin width of 5.8 kDa.

### PhaseScan analysis

Microfluidic devices were formulated employing AutoCAD software and subsequently produced through traditional soft-photolithography techniques and Polydimethylsiloxane (PDMS)-on-glass devices (Arosio et al, 2016; Qin et al, 2010; Saar et al, 2017). PhaseScan was operated as described previously (Arter et al, 2022; Sneideris et al, 2023; Agarwal et al, 2025). Briefly, NSP2 was incubated with 5 µM Atto NTA-Atto-488 (39625; Merck KGaA, Darmstadt, Germany) and mixed with a version of NSP5 (HP), IDR (HP), SC_Low_ or SC_Low_ HP, supplemented with Alexa Fluor 647 dye (Thermo Fisher) at concentrations ranging from 2 µM to 25 µM in PBS pH 7.4. Protein mixtures at varying concentrations were encapsulated in water-in-oil droplets. An epifluorescence microscope (Cairn Research) equipped with a 10 × objective (Nikon CFI Plan Fluor 10×, NA 0.3) was used to take images of microfluidic droplets in the imaging chamber. An automated image analysis script was employed for the detection of condensates and the quantification of protein concentrations within individual droplets. The data was graphically represented as a scatter plot, accompanied by a colour-coded heat map overlay illustrating the estimated probability of phase separation (Sneideris et al, 2023). To determine the phase separation probability, the phase diagram was partitioned into grids with a bin size of 1 + 3.322 × log (total number of data points). Subsequently, the probability was calculated by dividing the total number of points labelled as phase-separated by the overall number of points within each grid (Sneideris et al, 2023).

### Phosphorylation analysis by liquid chromatography-mass spectrometry

Protein digestion was carried out using S-Trap micro columns (Protifi) following the manufacturer’s protocol and employing MS-grade reagents. Briefly, 25 μL of 10% (w/v) SDS was mixed with 25 μL of protein solution. To this, 5 μL of 220 mM DTT was added, and the mixture was incubated at 50 °C for 10 min before cooling to ambient temperature. Alkylation was performed by adding 5.5 μL of 440 mM iodoacetamide and incubating in the dark for 30 min at room temperature. The sample was then acidified with phosphoric acid to a final concentration of 1.2% (v/v). Next, the solution was diluted with S-Trap binding buffer (100 mM TEAB, pH 7.1 in methanol) at a 7:1 ratio. Trypsin (Promega) was introduced from a 0.02 μg μL⁻¹ stock at an enzyme-to-protein ratio of 1:20. The mixture was loaded onto the S-Trap column and centrifuged at 4000×*g* for 30 s to capture proteins within the column matrix. The column was washed with 130 μL of binding buffer and centrifuged again at 4000×*g* for 30 s. Subsequently, 30 μL of trypsin solution (0.02 μg μL⁻¹ in TEAB) was added to the column, which was loosely capped, placed in a 1.5 mL microcentrifuge tube, and incubated at 47 °C for 90 min. After digestion, 40 μL of 50 mM TEAB was added, and the column was centrifuged at 4000×*g* for 1 min. Peptides were eluted sequentially with 0.2% (v/v) formic acid, followed by 50% (v/v) acetonitrile, with centrifugation (4000×*g* for 30 s) after each step. Combined eluates were dried using a Concentrator Plus vacuum centrifuge (Eppendorf).

Dried peptides were reconstituted in 0.1% (v/v) formic acid and analysed using a Vanquish Neo LC system (Thermo Scientific) coupled to an Orbitrap Eclipse Tribrid mass spectrometer (Thermo Scientific). Chromatographic separation was performed on an EASY-Spray reversed-phase column (2 μm particle size, 75 μm diameter, 500 mm length) at 250 nL min⁻¹ and 45 °C. Peptides were eluted over a 65-min gradient from 0–40% acetonitrile. MS1 scans were acquired at 120,000 resolution with an AGC target of 3 × 10⁶, maximum injection time of 50 ms, and a scan range of 350–2000 *m/z* in profile mode. The cycle time was set to 2.5 s, and precursors with charge states z = 2–7 were isolated using a 1.2 *m/z* window and fragmented by HCD with normalised collision energy of 30%. MS2 scans were recorded at 30,000 resolution with an AGC target of 1 × 10⁵, maximum injection time of 54 ms. Dynamic exclusion was enabled for 60 s. Data analysis was conducted using PEAKS Studio 11.

### Hydrogen–deuterium exchange mass spectrometry (HDX-MS)

HDX-MS experiments were performed using an automated HDX robot (LEAP Technologies, USA) coupled to an Acquity M-class LC and HDX manager (Waters Corporation, UK). For each injection, a total of 5 µL of protein solution containing NSP2 (25 µM), NSP5 or NSP5 HP (25 µM), SC_Low_ HP (25 µM) or a mixture of both (25 µM each) was added to 95 µL of deuterated buffer (1× PBS pD 7, in D_2_O). Samples were incubated at 4 °C for 0, 0.5, 5 and 10 min. The exchange reaction was quenched post labelling by the addition of 75 µL of quench buffer (1X PBS pH 1.8, 0.1% DDM) to 75 µL of the sample. A total of 90 µL quenched sample was passed through an immobilised pepsin column (Enzymate, Waters Corporation, UK) and the resultant peptides were trapped on a VanGuard Pre-Column (Acquity UPLC BEH C18 [17 µm, 2.1 mm×5 mm], Waters Corporation, UK) for 3 min. Separation of peptide fragments was achieved with a C18 column (75 µm x 150 mm, Waters Corporation, UK) eluting over a linear gradient of 0–40% (v/v) acetonitrile, 0.1% (v/v) formic acid in H_2_O (0.3% (v/v) formic acid) over 7 min at 40 µL min^−1^. Peptide fragments were detected using a Synapt G2-Si mass spectrometer (Waters Corporation, UK), operated in mobility-assisted data-independent analysis with dynamic range extension enabled (HDMSe). Data was analysed using PLGS and DynamX software (Waters Corporation, UK). Pepsin was excluded from the analysis, and restrictions for peptides in DynamX were minimum intensity = 1000, maximum sequence length = 25, minimum products per amino acid = 0.3, max ppm error = 10, file threshold = 2/3 replicates. Deuteros (Lau et al, 2019) was used to identify statistically significant protected and deprotected peptides with an applied confidence interval of 98%, visualised in Woods plots.

### Conservation analysis

Sequences encoding NSP2 were selected as described in consensus sequence creation above, and then aligned using the Clustal 2.1 tool (Madeira et al, 2022). Protein sequence conservation and subsequent model creation were performed using UCSF ChimeraX version 1.7 using the AL2CO entropy measure (Pei and Grishin, 2001). The conservation scores for NSP2 sequences were mapped onto SA11 NSP2 structure PDB ID:1L9V. NCBI IDs of NSP2 sequences used for analysis were: Q86505.1 (strain RF), AFK09596.1 (strain SA11), AKA40121.1 (S7_Low_), AGF91999.1 (S6_Low_), QIJ58153.1 (S5_Low_), BBB18135.1 (S4_Low_), ALJ83250.1, AHZ33501.1, QWY12027.1, AGE47301.1, ALJ83232.1, BBJ34822.1, ALQ56903.1, QWY12017.1, ACV73806.1, QCL12358.1, QCD16951.1, AAY55957.1, QWY12010.1, AGE46905.1, AGV31551.1, BAV57881.1, AGT98571.1, ADW11141.1, QCL12357.1 and AGE47337.1. The EMBL-EBI Job Dispatcher sequence analysis tools framework was used for multiple sequence alignment (Madeira et al, 2024), which was visualised using UCSF ChimeraX (Meng et al, 2023).

## Supplementary information

Appendix

Peer Review File

Source data Fig. 1

Source data Fig. 2

Source data Fig. 3

Source data Fig. 4

Source data Fig. 5

Source data Fig. 6

Source data Fig. 7

Source data Fig. 8

Source data Fig. 9

Expanded View Figures

## Data Availability

Nucleotide sequences were deposited in GenBank and have the following accession numbers: PP828582 (SC_Low_): https://www.ncbi.nlm.nih.gov/nuccore/2736088137. The whole plasmid sequence of pT7/SC_Low_ is available in Addgene (ID:220289). Mass spectrometry phosphorylation data have been deposited to the ProteomeXchange Consortium via the PRIDE partner repository with the dataset identifier PXD071271. Cell imaging data shown in Figs. 4 and 8 are available via BioStudies under accession S-BSST2581. The source data of this paper are collected in the following database record: biostudies:S-SCDT-10_1038-S44318-026-00814-z.

## References

[CR1] Afrikanova I, Fabbretti E, Miozzo MC, Burrone OR (1998) Rotavirus NSP5 phosphorylation is up-regulated by interaction with NSP2. J Gen Virol 79(Pt 11):2679–2686 9820143 10.1099/0022-1317-79-11-2679

[CR2] Agarwal T, Sneideris T, Svara F, Jermakovs K, Coyle H, Qamar S, Kava E, Scrutton R, Pleschka N, Peres P et al (2025) Mapping high resolution, multidimensional phase diagrams of physiological protein condensates. Preprint at bioRxiv 10.1101/2025.11.24.690258

[CR3] Arnoldi F, Campagna M, Eichwald C, Desselberger U, Burrone OR (2007) Interaction of rotavirus polymerase VP1 with nonstructural protein NSP5 is stronger than that with NSP2. J Virol 81:2128–2137 17182692 10.1128/JVI.01494-06 PMC1865955

[CR4] Arosio P, Müller T, Rajah L, Yates EV, Aprile FA, Zhang Y, Cohen SIA, White DA, Herling TW, De Genst EJ et al (2016) Microfluidic diffusion analysis of the sizes and interactions of proteins under native solution conditions. ACS Nano 10:333–341 26678709 10.1021/acsnano.5b04713

[CR5] Arter WE, Qi R, Erkamp NA, Krainer G, Didi K, Welsh TJ, Acker J, Nixon-Abell J, Qamar S, Guillén-Boixet J et al (2022) Biomolecular condensate phase diagrams with a combinatorial microdroplet platform. Nat Commun 13:7845 36543777 10.1038/s41467-022-35265-7 PMC9768726

[CR6] Aumiller WM, Keating CD (2016) Phosphorylation-mediated RNA/peptide complex coacervation as a model for intracellular liquid organelles. Nat Chem 8:129–137 26791895 10.1038/nchem.2414

[CR7] Banani SF, Lee HO, Hyman AA, Rosen MK (2017) Biomolecular condensates: organizers of cellular biochemistry. Nat Rev Mol Cell Biol 18:285–298 28225081 10.1038/nrm.2017.7 PMC7434221

[CR8] Banani SF, Rice AM, Peeples WB, Lin Y, Jain S, Parker R, Rosen MK (2016) Compositional control of phase-separated cellular bodies. Cell 166:651–663 27374333 10.1016/j.cell.2016.06.010 PMC4967043

[CR9] Berlow RB, Dyson HJ, Wright PE (2018) Expanding the paradigm: intrinsically disordered proteins and allosteric regulation. J Mol Biol 430:2309–2320 29634920 10.1016/j.jmb.2018.04.003 PMC6045455

[CR10] Berman HM, Westbrook J, Feng Z, Gilliland G, Bhat TN, Weissig H, Shindyalov IN, Bourne PE (2000) The Protein Data Bank. Nucleic Acids Res 28:235–242 10592235 10.1093/nar/28.1.235 PMC102472

[CR11] Bhandari K, Cotten MA, Kim J, Rosen MK, Schmit JD (2021) Structure-function properties in disordered condensates. J Phys Chem B 125:467–476 33395293 10.1021/acs.jpcb.0c11057 PMC8194388

[CR12] Blackhall J, Fuentes A, Hansen K, Magnusson G (1997) Serine protein kinase activity associated with rotavirus phosphoprotein NSP5. J Virol 71(1):138–144 8985332 10.1128/jvi.71.1.138-144.1997 PMC191033

[CR13] Blackhall J, Muñoz M, Fuentes A, Magnusson G (1998) Analysis of rotavirus nonstructural protein NSP5 phosphorylation. J Virol 72:6398–6405 9658080 10.1128/jvi.72.8.6398-6405.1998 PMC109791

[CR14] Borg M, Mittag T, Pawson T, Tyers M, Forman-Kay JD, Chan HS (2007) Polyelectrostatic interactions of disordered ligands suggest a physical basis for ultrasensitivity. Proc Natl Acad Sci USA 104:9650–9655 17522259 10.1073/pnas.0702580104 PMC1887549

[CR15] Borodavka A, Acker J (2023) Seeing biomolecular condensates through the lens of viruses. Annu Rev Virol 10:163–182 37040799 10.1146/annurev-virology-111821-103226

[CR16] Borodavka A, Dykeman EC, Schrimpf W, Lamb DC (2017) Protein-mediated RNA folding governs sequence-specific interactions between rotavirus genome segments. Elife 6:e27453 28922109 10.7554/eLife.27453 PMC5621836

[CR17] Brangwynne CP, Eckmann CR, Courson DS, Rybarska A, Hoege C, Gharakhani J, Jülicher F, Hyman AA (2009) Germline P granules are liquid droplets that localize by controlled dissolution/condensation. Science 324:1729–1732 19460965 10.1126/science.1172046

[CR18] Brangwynne CP, Mitchison TJ, Hyman AA (2011) Active liquid-like behavior of nucleoli determines their size and shape in *Xenopus laevis* oocytes. Proc Natl Acad Sci 108:4334–4339 21368180 10.1073/pnas.1017150108 PMC3060270

[CR19] Brangwynne CP, Tompa P, Pappu RV (2015) Polymer physics of intracellular phase transitions. Nat Phys 11:899–904

[CR20] Bravo JPK, Bartnik K, Venditti L, Acker J, Gail EH, Colyer A, Davidovich C, Lamb DC, Tuma R, Calabrese AN et al (2021) Structural basis of rotavirus RNA chaperone displacement and RNA annealing. Proc Natl Acad Sci USA 118:e2100198118 34615715 10.1073/pnas.2100198118 PMC8521686

[CR21] Bressler SG, Mitrany A, Wenger A, Näthke I, Friedler A (2023) The oligomerization domains of the APC protein mediate liquid-liquid phase separation that is phosphorylation controlled. Int J Mol Sci 24:6478 37047451 10.3390/ijms24076478 PMC10095272

[CR22] Campagna M, Budini M, Arnoldi F, Desselberger U, Allende JE, Burrone OR (2007) Impaired hyperphosphorylation of rotavirus NSP5 in cells depleted of casein kinase 1α is associated with the formation of viroplasms with altered morphology and a moderate decrease in virus replication. J Gen Virol 88:2800–2810 17872534 10.1099/vir.0.82922-0

[CR23] Choi J-M, Holehouse AS, Pappu RV (2020) Physical principles underlying the complex biology of intracellular phase transitions. Annu Rev Biophys 49:107–133 32004090 10.1146/annurev-biophys-121219-081629 PMC10715172

[CR24] Chu X, Sun T, Li Q, Xu Y, Zhang Z, Lai L, Pei J (2022) Prediction of liquid–liquid phase separating proteins using machine learning. BMC Bioinforma 23:72 10.1186/s12859-022-04599-w PMC8845408 35168563

[CR25] Colyer A, Acker J, Borodavka A, Calabrese AN (2025) Uncovering protein conformational dynamics within two-component viral biomolecular condensates. Protein Sci 34:e70181 40521608 10.1002/pro.70181 PMC12168090

[CR61] Carreño-Torres JJ, Gutiérrez M, Arias CF, López S, Isa P (2010) Characterization of viroplasm formation during the earlys tages of rotavirus infection. Virol J. 29;7:350 10.1186/1743-422X-7-350 PMC3009706 21114853

[CR26] Criglar JM, Anish R, Hu L, Crawford SE, Sankaran B, Prasad BVV, Estes MK (2018) Phosphorylation cascade regulates the formation and maturation of rotaviral replication factories. Proc Natl Acad Sci USA 115:E12015–E12023 30509975 10.1073/pnas.1717944115 PMC6304940

[CR27] Criglar JM, Hu L, Crawford SE, Hyser JM, Broughman JR, Prasad BV, Estes MK (2014) A novel form of rotavirus NSP2 and phosphorylation-dependent NSP2-NSP5 interactions are associated with viroplasm assembly. J Virol 88(2):786–798 24198401 10.1128/JVI.03022-13 PMC3911676

[CR95] Diebold O, Gonzalez V, Venditti L, Sharp C, Blake RA, Tan WS, Stevens J, Caddy S, Digard P, Borodavka A, Gaunt E (2022) Using Species a Rotavirus Reverse Genetics to Engineer Chimeric Viruses Expressing SARS-CoV-2 Spike Epitopes. J Virol 96:e0048822 10.1128/jvi.00488-22 PMC9327695 35758692

[CR28] Dyson HJ (2023) Vital for viruses: intrinsically disordered proteins. J Mol Biol 435:167860 37330280 10.1016/j.jmb.2022.167860 PMC10656058

[CR29] Eichwald C, Jacob G, Muszynski B, Allende JE, Burrone OR (2004a) Uncoupling substrate and activation functions of rotavirus NSP5: phosphorylation of Ser-67 by casein kinase 1 is essential for hyperphosphorylation. Proc Natl Acad Sci USA 101:16304–16309 15520389 10.1073/pnas.0406691101 PMC528968

[CR30] Eichwald C, Rodriguez JF, Burrone OR (2004b) Characterization of rotavirus NSP2/NSP5 interactions and the dynamics of viroplasm formation. J Gen Virol 85:625–634 14993647 10.1099/vir.0.19611-0

[CR31] Fabbretti E, Afrikanova I, Vascotto F, Burrone OR (1999) Two non-structural rotavirus proteins, NSP2 and NSP5, form viroplasm-like structures in vivo. J Gen Virol 80:333–339 10073692 10.1099/0022-1317-80-2-333

[CR32] Ferreon ACM, Ferreon JC, Wright PE, Deniz AA (2013) Modulation of allostery by protein intrinsic disorder. Nature 498:390–394 23783631 10.1038/nature12294 PMC3718496

[CR33] Galagedera SKK, Dao TP, Enos SE, Chaudhuri A, Schmit JD, Castañeda CA (2023) Polyubiquitin ligand-induced phase transitions are optimized by spacing between ubiquitin units. Proc Natl Acad Sci USA 120:e2306638120 37824531 10.1073/pnas.2306638120 PMC10589717

[CR34] Geiger F, Acker J, Papa G, Wang X, Arter WE, Saar KL, Erkamp NA, Qi R, Bravo JP, Strauss S et al (2021) Liquid-liquid phase separation underpins the formation of replication factories in rotaviruses. EMBO J 40:e107711 34524703 10.15252/embj.2021107711 PMC8561643

[CR35] Ginell GM, Holehouse AS (2023) An introduction to the stickers-and-spacers framework as applied to biomolecular condensates. Methods Mol Biol 2563:95–116 36227469 10.1007/978-1-0716-2663-4_4

[CR36] Golosova O, Henderson R, Vaskin Y, Gabrielian A, Grekhov G, Nagarajan V, Oler AJ, Quiñones M, Hurt D, Fursov M et al (2014) Unipro UGENE NGS pipelines and components for variant calling, RNA-seq and ChIP-seq data analyses. PeerJ 2:e644 25392756 10.7717/peerj.644 PMC4226638

[CR37] Grams N, Charman M, Halko E, Lauman R, Garcia BA, Weitzman MD (2024) Phosphorylation regulates viral biomolecular condensates to promote infectious progeny production. EMBO J 43:277–303 38177504 10.1038/s44318-023-00021-0 PMC10897327

[CR38] Guindon S, Dufayard J-F, Lefort V, Anisimova M, Hordijk W, Gascuel O (2010) New algorithms and methods to estimate maximum-likelihood phylogenies: assessing the performance of PhyML 3.0. Syst Biol 59:307–321 20525638 10.1093/sysbio/syq010

[CR39] Hatos A, Teixeira JMC, Barrera-Vilarmau S, Horvath A, Tosatto SCE, Vendruscolo M, Fuxreiter M (2023) FuzPred: a web server for the sequence-based prediction of the context-dependent binding modes of proteins. Nucleic Acids Res 51:W198–W206 36987846 10.1093/nar/gkad214 PMC10320189

[CR40] Heinrich BS, Maliga Z, Stein DA, Hyman AA, Whelan SPJ (2018) Phase transitions drive the formation of vesicular stomatitis virus replication compartments. mBio 9:e02290–17 30181255 10.1128/mBio.02290-17 PMC6123442

[CR41] Hilser VJ, Thompson EB (2007) Intrinsic disorder as a mechanism to optimize allosteric coupling in proteins. Proc Natl Acad Sci USA 104:8311–8315 17494761 10.1073/pnas.0700329104 PMC1895946

[CR42] Hyman AA, Weber CA, Jülicher F (2014) Liquid-liquid phase separation in biology. Annu Rev Cell Dev Biol 30:39–58 25288112 10.1146/annurev-cellbio-100913-013325

[CR43] Jayaram H, Taraporewala Z, Patton JT, Prasad BVV (2002) Rotavirus protein involved in genome replication and packaging exhibits a HIT-like fold. Nature 417:311–315 12015608 10.1038/417311a

[CR44] Jiang X, Jayaram H, Kumar M, Ludtke SJ, Estes MK, Prasad BVV (2006) Cryoelectron microscopy structures of rotavirus NSP2-NSP5 and NSP2-RNA complexes: implications for genome replication. J Virol 80:10829–10835 16928740 10.1128/JVI.01347-06 PMC1641785

[CR45] Jumper J, Evans R, Pritzel A, Green T, Figurnov M, Ronneberger O, Tunyasuvunakool K, Bates R, Žídek A, Potapenko A et al (2021) Highly accurate protein structure prediction with AlphaFold. Nature 596:583–589 34265844 10.1038/s41586-021-03819-2 PMC8371605

[CR46] Katoh K, Standley DM (2013) MAFFT multiple sequence alignment software version 7: improvements in performance and usability. Mol Biol Evol 30:772–780 23329690 10.1093/molbev/mst010 PMC3603318

[CR47] Kern D, Zuiderweg ER (2003) The role of dynamics in allosteric regulation. Curr Opin Struct Biol 13:748–757 14675554 10.1016/j.sbi.2003.10.008

[CR48] Kimura M (1981) Estimation of evolutionary distances between homologous nucleotide sequences. Proc Natl Acad Sci USA 78:454–458 6165991 10.1073/pnas.78.1.454 PMC319072

[CR49] Komoto S, Kanai Y, Fukuda S, Kugita M, Kawagishi T, Ito N, Sugiyama M, Matsuura Y, Kobayashi T, Taniguchi K (2017) Reverse genetics system demonstrates that rotavirus nonstructural protein NSP6 is not essential for viral replication in cell culture. J Virol. 10.1128/jvi.00695-17. 10.1128/JVI.00695-17 PMC5640853 28794037

[CR50] Krishnan N, Koveal D, Miller DH, Xue B, Akshinthala SD, Kragelj J, Jensen MR, Gauss C-M, Page R, Blackledge M et al (2014) Targeting the disordered C terminus of PTP1B with an allosteric inhibitor. Nat Chem Biol 10:558–566 24845231 10.1038/nchembio.1528 PMC4062594

[CR51] Lau AMC, Ahdash Z, Martens C, Politis A (2019) Deuteros: software for rapid analysis and visualization of data from differential hydrogen deuterium exchange-mass spectrometry. Bioinformatics 35:3171–3173 30649183 10.1093/bioinformatics/btz022 PMC6736138

[CR52] Lee M, Cosic A, Tobler K, Aguilar C, Fraefel C, Eichwald C (2024) Characterization of viroplasm-like structures by co-expression of NSP5 and NSP2 across rotavirus species A to J. J Virol 98:e0097524 39194242 10.1128/jvi.00975-24 PMC11423710

[CR53] Letunic I, Bork P (2021) Interactive Tree Of Life (iTOL) v5: an online tool for phylogenetic tree display and annotation. Nucleic Acids Res 49:W293–W296 33885785 10.1093/nar/gkab301 PMC8265157

[CR54] Levenshtein VI (1966) Binary codes capable of correcting deletions, insertions and reversals. BibSonomy Sov Phys Dokl 10:707–710

[CR55] Li Q, Peng X, Li Y, Tang W, Zhu J, Huang J, Qi Y, Zhang Z (2020) LLPSDB: a database of proteins undergoing liquid–liquid phase separation in vitro. Nucleic Acids Res 48:D320–D327 31906602 10.1093/nar/gkz778 PMC6943074

[CR56] Madeira F, Madhusoodanan N, Lee J, Eusebi A, Niewielska A, Tivey ARN, Lopez R, Butcher S (2024) The EMBL-EBI Job Dispatcher sequence analysis tools framework in 2024. Nucleic Acids Res 52:W521–W525 38597606 10.1093/nar/gkae241 PMC11223882

[CR57] Madeira F, Pearce M, Tivey ARN, Basutkar P, Lee J, Edbali O, Madhusoodanan N, Kolesnikov A, Lopez R (2022) Search and sequence analysis tools services from EMBL-EBI in 2022. Nucleic Acids Res 50:W276–W279 35412617 10.1093/nar/gkac240 PMC9252731

[CR58] Martin D, Ouldali M, Ménétrey J, Poncet D (2011) Structural organisation of the rotavirus nonstructural protein NSP5. J Mol Biol 413:209–221 21864538 10.1016/j.jmb.2011.08.008

[CR59] Martin EW, Holehouse AS, Peran I, Farag M, Incicco JJ, Bremer A, Grace CR, Soranno A, Pappu RV, Mittag T (2020) Valence and patterning of aromatic residues determine the phase behavior of prion-like domains. Science 367:694–699 32029630 10.1126/science.aaw8653 PMC7297187

[CR60] Meng EC, Goddard TD, Pettersen EF, Couch GS, Pearson ZJ, Morris JH, Ferrin TE (2023) UCSF ChimeraX: tools for structure building and analysis. Protein Sci 32:e4792 37774136 10.1002/pro.4792 PMC10588335

[CR62] Mikolov T, Chen K, Corrado G, Dean J (2013a) Efficient estimation of word representations in vector space. Preprint at 10.48550/arXiv.1310.4546

[CR63] Mikolov T, Sutskever I, Chen K, Corrado G, Dean J (2013b) Distributed representations of words and phrases and their compositionality. Preprint at 10.48550/arXiv.1310.4546

[CR64] Mishra PM, Verma NC, Rao C, Uversky VN, Nandi CK (2020) Intrinsically disordered proteins of viruses: Involvement in the mechanism of cell regulation and pathogenesis. Prog Mol Biol Transl Sci 174:1–78 32828463 10.1016/bs.pmbts.2020.03.001 PMC7129803

[CR65] Monette A, Niu M, Chen L, Rao S, Gorelick RJ, Mouland AJ (2020) Pan-retroviral nucleocapsid-mediated phase separation regulates genomic RNA positioning and trafficking. Cell Rep 31:107520 32320662 10.1016/j.celrep.2020.03.084 PMC8965748

[CR66] Nichols SL, Nilsson EM, Brown-Harding H, LaConte LEW, Acker J, Borodavka A, McDonald Esstman S (2023) Flexibility of the rotavirus NSP2 C-terminal region supports factory formation via liquid-liquid phase separation. J Virol 97:e0003923 36749077 10.1128/jvi.00039-23 PMC9973012

[CR67] Nikolic J, Civas A, Lama Z, Lagaudrière-Gesbert C, Blondel D (2016) Rabies virus infection induces the formation of stress granules closely connected to the viral factories. PLoS Pathog 12:e1005942 27749929 10.1371/journal.ppat.1005942 PMC5066959

[CR68] Okonechnikov K, Golosova O, Fursov M, the UGENE team (2012) Unipro UGENE: a unified bioinformatics toolkit. Bioinformatics 28:1166–1167 22368248 10.1093/bioinformatics/bts091

[CR94] Peña-Gil N, Randazzo W, Carmona-Vicente N, Santiso-Bellón C, Cárcamo-Cálvo R, Navarro-Lleó N, Monedero V, Yebra MJ, Buesa J, Gozalbo-Rovira R et al. (2023) Culture of Human Rotaviruses in Relevant Models Shows Differences in Culture-Adapted and Nonculture-Adapted Strains. Int J Mol Sci 24:17362 10.3390/ijms242417362 PMC10743750 38139191

[CR69] Papa G, Venditti L, Arnoldi F, Schraner EM, Potgieter C, Borodavka A, Eichwald C, Burrone OR (2019) Recombinant rotaviruses rescued by reverse genetics reveal the role of NSP5 hyperphosphorylation in the assembly of viral factories. J Virol 94:e01110–e01119 31619556 10.1128/JVI.01110-19 PMC6912106

[CR70] Pei J, Grishin NV (2001) AL2CO: calculation of positional conservation in a protein sequence alignment. Bioinformatics 17:700–712 11524371 10.1093/bioinformatics/17.8.700

[CR71] Pourianfar HR, Javadi A, Grollo L (2012) A colorimetric-based accurate method for the determination of enterovirus 71 titer. Indian J Virol 23:303–310 24293817 10.1007/s13337-012-0105-0 PMC3550799

[CR72] Pufall MA, Lee GM, Nelson ML, Kang H-S, Velyvis A, Kay LE, McIntosh LP, Graves BJ (2005) Variable control of Ets-1 DNA binding by multiple phosphates in an unstructured region. Science 309:142–145 15994560 10.1126/science.1111915

[CR73] Qin D, Xia Y, Whitesides GM (2010) Soft lithography for micro- and nanoscale patterning. Nat Protoc 5:491–502 20203666 10.1038/nprot.2009.234

[CR74] Ramirez DA, Hough LE, Shirts MR (2023) Coiled-coi domains are sufficient to drive liquid-liquid phase separation of proteins in molecular models. Preprint at bioRxiv 10.1101/2023.05.31.543124

[CR75] Ramšak M, Ramirez DA, Hough LE, Shirts MR, Vidmar S, Eleršič Filipič K, Anderluh G, Jerala R (2023) Programmable de novo designed coiled coil-mediated phase separation in mammalian cells. Nat Commun 14:7973 38042897 10.1038/s41467-023-43742-w PMC10693550

[CR76] Ranganathan S, Dasmeh P, Furniss S, Shakhnovich E (2023) Phosphorylation sites are evolutionary checkpoints against liquid–solid transition in protein condensates. Proc Natl Acad Sci USA 120:e2215828120 37155880 10.1073/pnas.2215828120 PMC10193986

[CR77] Reed LJ, Muench H (1938) A simple method of estimating fifty per cent endpoints. Am J Epidemiol 27:493–497

[CR78] Rose R, Golosova O, Sukhomlinov D, Tiunov A, Prosperi M (2019) Flexible design of multiple metagenomics classification pipelines with UGENE. Bioinformatics 35:1963–1965 30358807 10.1093/bioinformatics/bty901

[CR79] Saar KL, Morgunov AS, Qi R, Arter WE, Krainer G, Lee AA, Knowles TPJ (2021) Learning the molecular grammar of protein condensates from sequence determinants and embeddings. Proc Natl Acad Sci USA 118:e2019053118 33827920 10.1073/pnas.2019053118 PMC8053968

[CR80] Saar KL, Zhang Y, Müller T, Kumar CP, Devenish S, Lynn A, Łapińska U, Yang X, Linse S, Knowles TPJ (2017) On-chip label-free protein analysis with downstream electrodes for direct removal of electrolysis products. Lab Chip 18:162–170 29192926 10.1039/c7lc00797c

[CR81] Schindelin J, Arganda-Carreras I, Frise E, Kaynig V, Longair M, Pietzsch T, Preibisch S, Rueden C, Saalfeld S, Schmid B et al (2012) Fiji - an open source platform for biological image analysis. Nat Methods. 10.1038/nmeth.2019. 10.1038/nmeth.2019 PMC3855844 22743772

[CR82] Schmit JD, Feric M, Dundr M (2021) How fierarchical interactions make membraneless organelles tick like clockwork. Trends Biochem Sci 46:525–534 33483232 10.1016/j.tibs.2020.12.011 PMC8195823

[CR83] Schuck P, Taraporewala Z, McPhie P, Patton JT (2001) Rotavirus nonstructural protein NSP2 self-assembles into octamers that undergo ligand-induced conformational changes. J Biol Chem 276:9679–9687 11121414 10.1074/jbc.M009398200

[CR84] Sen A, Sen N, Mackow ER (2007) The formation of viroplasm-like structures by the rotavirus NSP5 protein is calcium regulated and directed by a C-terminal helical domain. J Virol 81:11758–11767 17699573 10.1128/JVI.01124-07 PMC2168809

[CR85] Sneideris T, Erkamp NA, Ausserwöger H, Saar KL, Welsh TJ, Qian D, Katsuya-Gaviria K, Johncock MLLY, Krainer G, Borodavka A et al (2023) Targeting nucleic acid phase transitions as a mechanism of action for antimicrobial peptides. Nat Commun 14:7170 37935659 10.1038/s41467-023-42374-4 PMC10630377

[CR86] Sotelo PH, Schümann M, Krause E, Chnaiderman J (2010) Analysis of rotavirus non-structural protein NSP5 by mass spectrometry reveals a complex phosphorylation pattern. Virus Res 149:104–108 20036292 10.1016/j.virusres.2009.12.006

[CR87] Strauss S, Acker J, Papa G, Desirò D, Schueder F, Borodavka A, Jungmann R (2023) Principles of RNA recruitment to viral ribonucleoprotein condensates in a segmented dsRNA virus. eLife 12:e68670 36700549 10.7554/eLife.68670 PMC9925054

[CR88] Swofford DL, Olsen GJ, Waddell PJ, Hillis DM (1996) Phylogenetic inference. In: Hillis DM, Moritz C, Mable BK editors. Molecular systematics. Sunderland, MA: Sinnauer Associates, pp. 407–514

[CR89] Tavaré S, Adams DC, Fedrigo O, Naylor GJ (2000) A model for phylogenetic inference using structural and chemical covariates. Pac Symp Biocomput 2001:215–225 10.1142/9789814447362_0022 11262942

[CR90] Torres-Vega MA, González RA, Duarte M, Poncet D, López S, Arias CF (2000) The C-terminal domain of rotavirus NSP5 is essential for its multimerization, hyperphosphorylation and interaction with NSP6. J Gen Virol 81:821–830 10675420 10.1099/0022-1317-81-3-821

[CR91] Wang J, Choi J-M, Holehouse AS, Lee HO, Zhang X, Jahnel M, Maharana S, Lemaitre R, Pozniakovsky A, Drechsel D et al (2018) A molecular grammar governing the driving forces for phase separation of prion-like RNA binding proteins. Cell 174:688–699.e16 29961577 10.1016/j.cell.2018.06.006 PMC6063760

[CR92] Yen JC, Chang FJ, Chang S (1995) A new criterion for automatic multilevel thresholding. IEEE Trans Image Process 4:370–378 18289986 10.1109/83.366472

[CR93] Zhou S, Fu Z, Zhang Z, Jia X, Xu G, Sun L, Sun F, Gao P, Xu P, Deng H (2022) Liquid–liquid phase separation mediates the formation of herpesvirus assembly compartments. J Cell Biol 222:e202201088 36250941 10.1083/jcb.202201088 PMC9579985

