# Supplementary material for: Phosphorylation tunes strain-specific protein condensation during rotavirus replication organelle assembly
Source: EMBO J. 2026 May 26;45(13):4733–65. doi: 10.1038/s44318-026-00814-z (PMC13324165; doi:10.1038/s44318-026-00814-z)
Supplement: Supplementary file 1 — Appendix [file 44318_2026_814_MOESM1_ESM.pdf]

Appendix for

Phosphorylation tunes strain-specific protein condensation during rotavirus replication organelle assembly

Table of contents

Appendix Figure S1 Page 2

Appendix Table S1 Page 3

```
SCLow-P10       ATGTCTCTCAGTATTGACGTGACGAGTCTTCCTTCTATTTCTTCAAGTGTATATAAGAAT  60
reference       ATGTCTCTCAGTATTGACGTGACGAGTCTTCCTTCTATTTCTTCAAGTGTATATAAGAAT  60
                ************************************************************

SCLow-P10       GAATCGTTTTCAACAACGTCAACTATTTCTGGAAAATCTATTGGTAGGAGTGAACAGTAC  120
reference       GAATCGTTTTCAACAACGTCAACTATTTCTGGAAAATCTATTGGTAGGAGTGAACAGTAC  120
                ************************************************************

SCLow-P10       ATTTCACCAGATGCAGAAGCATTCAATAAATACATGCTGTCGAAGTCTCCAGAGGATATT  180
reference       ATTTCACCAGATGCAGAAGCATTCAATAAATACATGCTGTCGAAGTCTCCAGAGGATATT  180
                ************************************************************

SCLow-P10       GGACCATTTGATTCTGCTTCAAACGATCCACTCACCAGTTTTTCGATTAGATCGAATGCA  240
reference       GGACCATTTGATTCTGCTTCAAACGATCCACTCACCAGTTTTTCGATTAGATCGAATGCA  240
                ************************************************************

SCLow-P10       GTTAAGACAAACGCAGACGCTGGCGTGTCTATGGATTCATCAGCACAATCACGACTTTCA  300
reference       GTTAAGACAAACGCAGACGCTGGCGTGTCTATGGATTCATCAGCACAATCACGACTTTCA  300
                ************************************************************

SCLow-P10       AGTGATATTGGATGCGATCAAATGGATTTCTCCTTAAATAAAGGCATAAAAATAGATGCT  360
reference       AGTGATATCGGATGCGATCAAATGGATTTCTCCTTAAATAAAGGCATAAAAATAGATGCT  360
                ******** ***************************************************

SCLow-P10       ACTTTGGACTCATCAATATCAATATCTACGACTAGTAAAAAGGAGAAATCAAAACAAGAG  420
reference       ACTTTGGACTCATCAATATCAATATCTACGACTAGTAAAAAGGAGAAATCAAAACAAGAG  420
                ************************************************************

SCLow-P10       TATAAAAATAAATATAAGAAGTGCTACCCAAAAATTGAAGCAGAGTCTGATTCAGATGAA  480
reference       TATAAAAATAAATATAAGAAGTGCTACCCAAAAATTGAAGCAGAGTCTGATTCAGATGAA  480
                ************************************************************

SCLow-P10       TATGTACTGGATGATTCAGATAGTGATGATGGTAAATGTAAGAACTGTAAATATAAGAAG  540
reference       TATGTACTGGATGATTCAGATAGTGATGATGGTAAATGTAAGAACTGTAAATATAAGAAG  540
                ************************************************************

SCLow-P10       AAATACTTCGCATTAAGATTGAGAATGAAACAAGTCGCAATGCAATTGATTAAAGATTTG  600
reference       AAATACTTCGCATTAAGATTGAGAATGAAACAAGTCGCAATGCAATTGATTAAAGATTTG  600
                ************************************************************

SCLow-P10       TAA   603
reference       TAA   603
                ***
```

Appendix Figure S1. CLUSTAL O (1.2.4) multiple sequence alignment (MSA) of gene segment 11 RNA extracted from SC_{Low} – RV-infected cells and amplified by RT-PCR. SCLow-P10 denotes the sequence of the RNA extracted after 10 sequential passages in MA104 cells, as described in Methods. After 10 passages, the protein sequence remains identical between the reference and the P10 passage, confirming that the NSP5 sequence remained stable for at least ten passages in MA104 cells.

Name	NCBI ID	biophysical	word2vec	Average DeePhase score
SA11	BAW94621.1	0.729	0.560	0.645
RF	AHF49898.1	0.629	0.554	0.592
SC_{Low}	PP828582.1	0.200	0.520	0.360
$S1_{Low}$	QIN53369.1	0.199	0.370	0.285
$S2_{Low}$	QIN53336.1	0.199	0.499	0.349
$S3_{Low}$	ACC91692.1	0.212	0.510	0.361
$S4_{Low}$	BBB18699.1	0.200	0.535	0.368
$S5_{Low}$	QIJ58158.1	0.191	0.553	0.372
$S6_{Low}$	AGF92026.1	0.198	0.547	0.373
$S7_{Low}$	AKA40124.1	0.191	0.560	0.375

Appendix Table S1. DeePhase scores and associated metadata for all major NSP5 sequences analysed in this study. DeePhase scores were computed and represent the average of the biophysical (physico-chemical features, hydrophobicity and disorder) and word2vec-based (trained to identify sequence features associated with phase separation) components, as described by Saar et al. (2021). The Name column lists the identifier used throughout this study for each sequence, with the corresponding GenBank accession number provided.
