# Supplementary figures and images for: Phosphorylation tunes strain-specific protein condensation during rotavirus replication organelle assembly (part 1 of 3)
Source: EMBO J. 2026 May 26;45(13):4733–65. doi: 10.1038/s44318-026-00814-z (PMC13324165; doi:10.1038/s44318-026-00814-z)

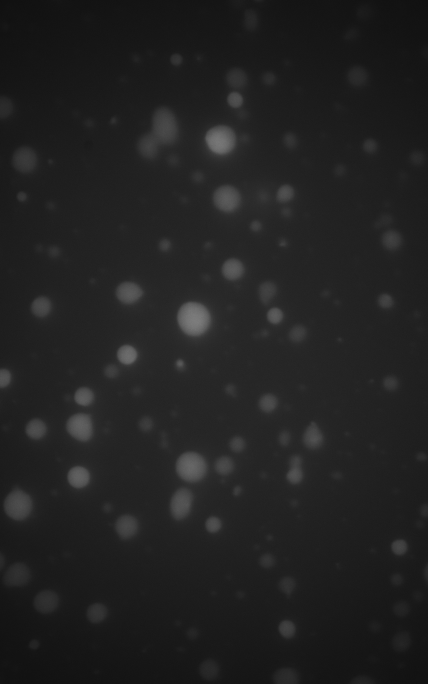

Supplement: Supplementary file 3 — Source data Fig. 1 [file 44318_2026_814_MOESM3_ESM.zip › raw data/NSP5 SA11.tif]

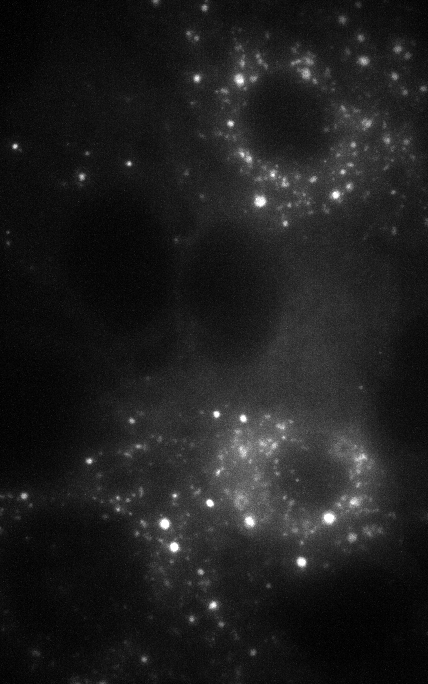

Supplement: Supplementary file 3 — Source data Fig. 1 [file 44318_2026_814_MOESM3_ESM.zip › raw data/Fig1SA11.tif]

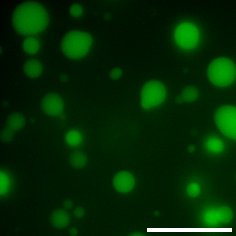

Supplement: Supplementary file 3 — Source data Fig. 1 [file 44318_2026_814_MOESM3_ESM.zip › raw data/NSP5 RF-1.jpg]

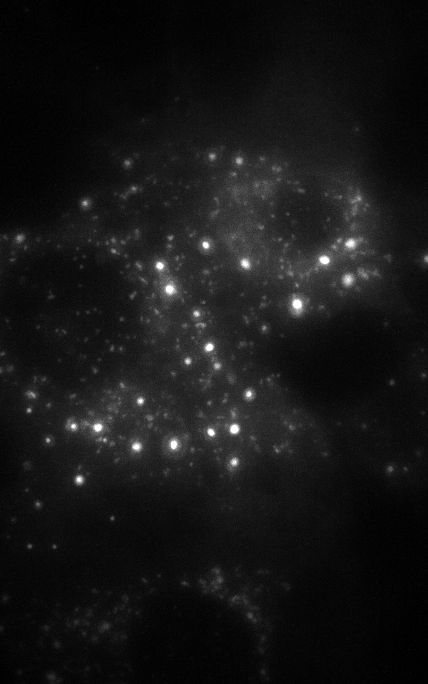

Supplement: Supplementary file 3 — Source data Fig. 1 [file 44318_2026_814_MOESM3_ESM.zip › raw data/Fig1RF.tif]

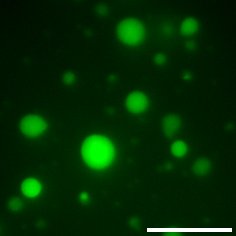

Supplement: Supplementary file 3 — Source data Fig. 1 [file 44318_2026_814_MOESM3_ESM.zip › raw data/NSP5 SA11-1.jpg]

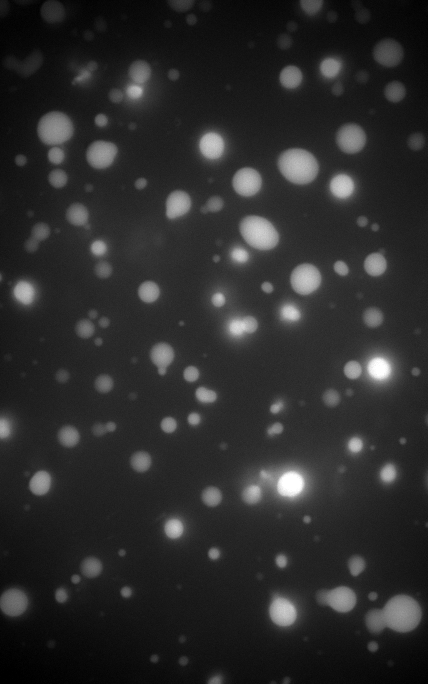

Supplement: Supplementary file 3 — Source data Fig. 1 [file 44318_2026_814_MOESM3_ESM.zip › raw data/NSP5 RF.tif]

# Replication Kinetics

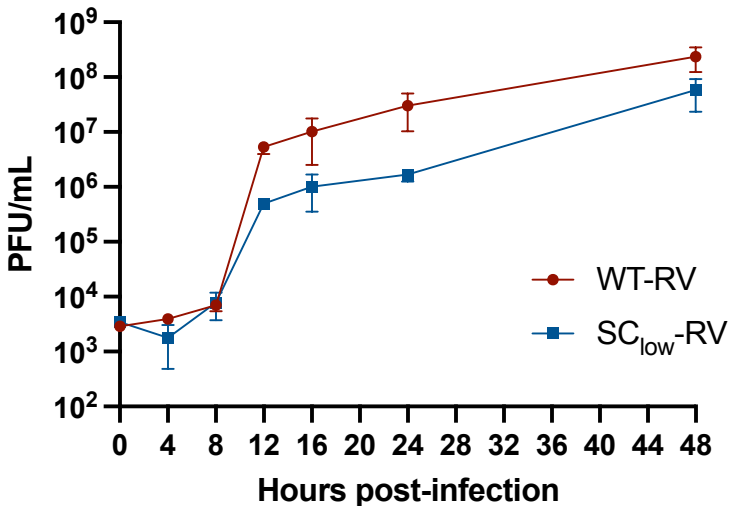

Supplement: Supplementary file 5 — Source data Fig. 3 [file 44318_2026_814_MOESM5_ESM.zip › Figure 3/Figure 3B/Figure 3B-replication kinetics MOI1.pdf]

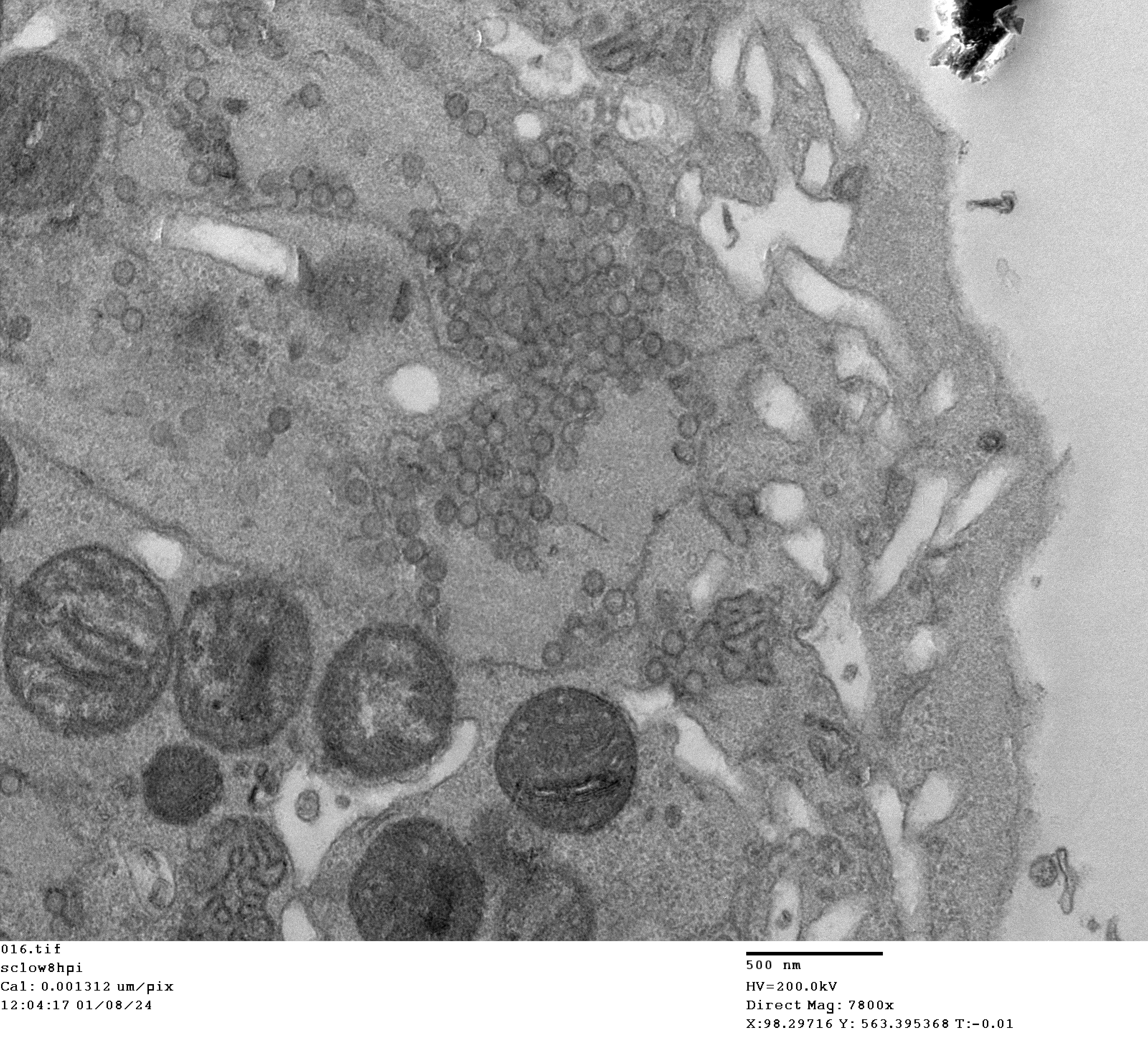

Supplement: Supplementary file 5 — Source data Fig. 3 [file 44318_2026_814_MOESM5_ESM.zip › Figure 3/Figure 3C/SClow 8hpi TEM.tif]

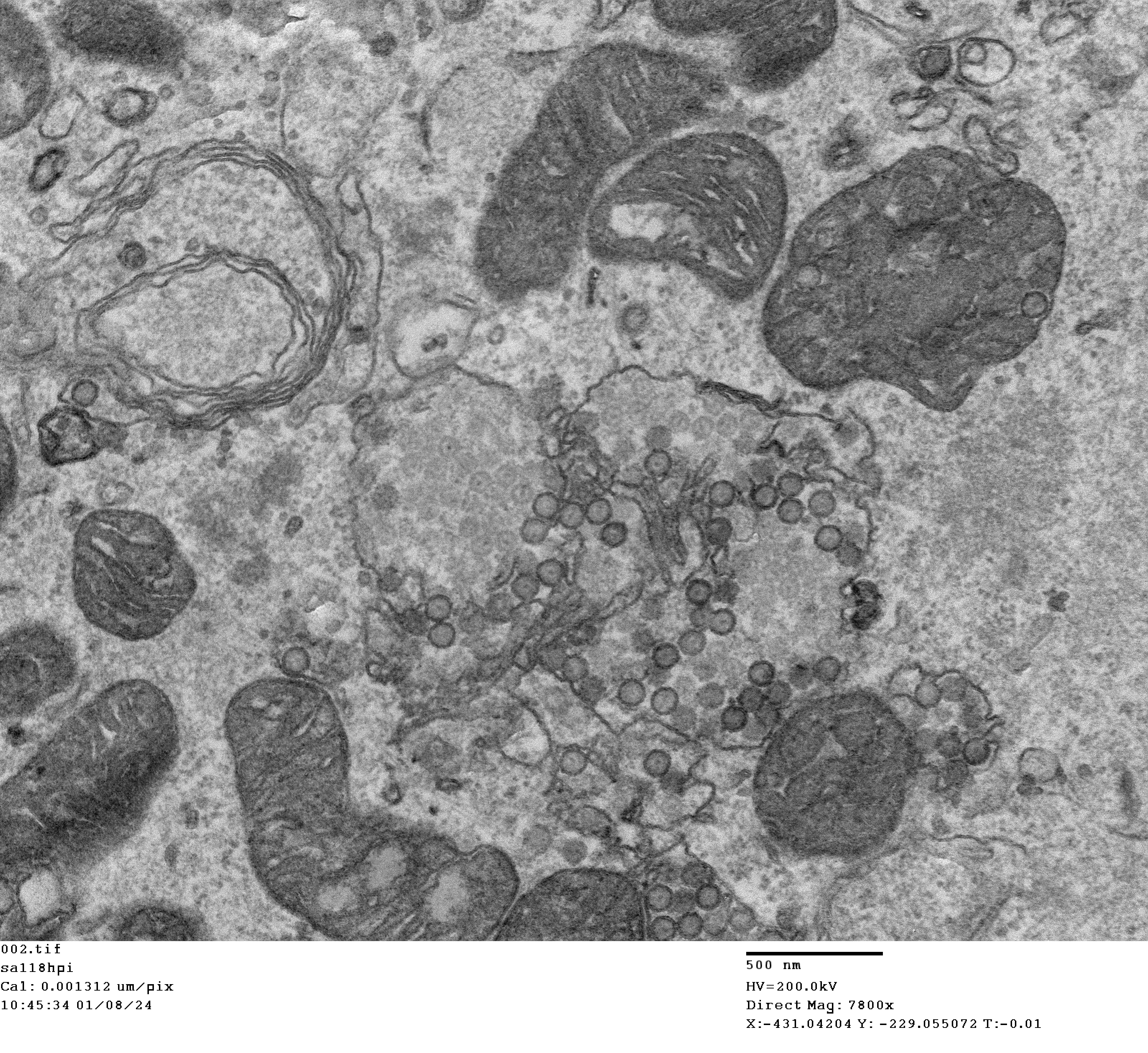

Supplement: Supplementary file 5 — Source data Fig. 3 [file 44318_2026_814_MOESM5_ESM.zip › Figure 3/Figure 3C/SA11 8 hpi TEM.tif]

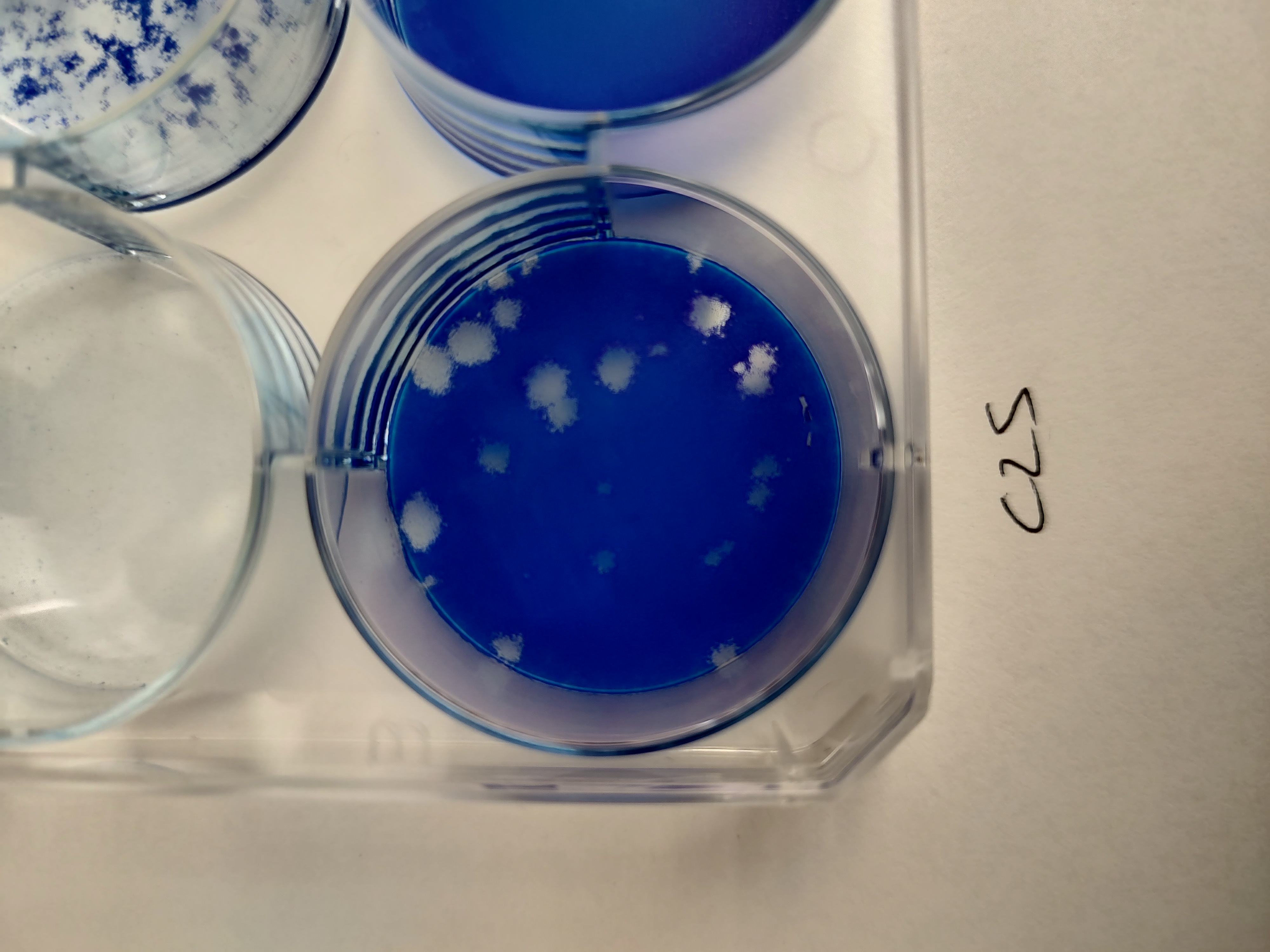

Supplement: Supplementary file 5 — Source data Fig. 3 [file 44318_2026_814_MOESM5_ESM.zip › Figure 3/Figure 3A/C2S.jpg]

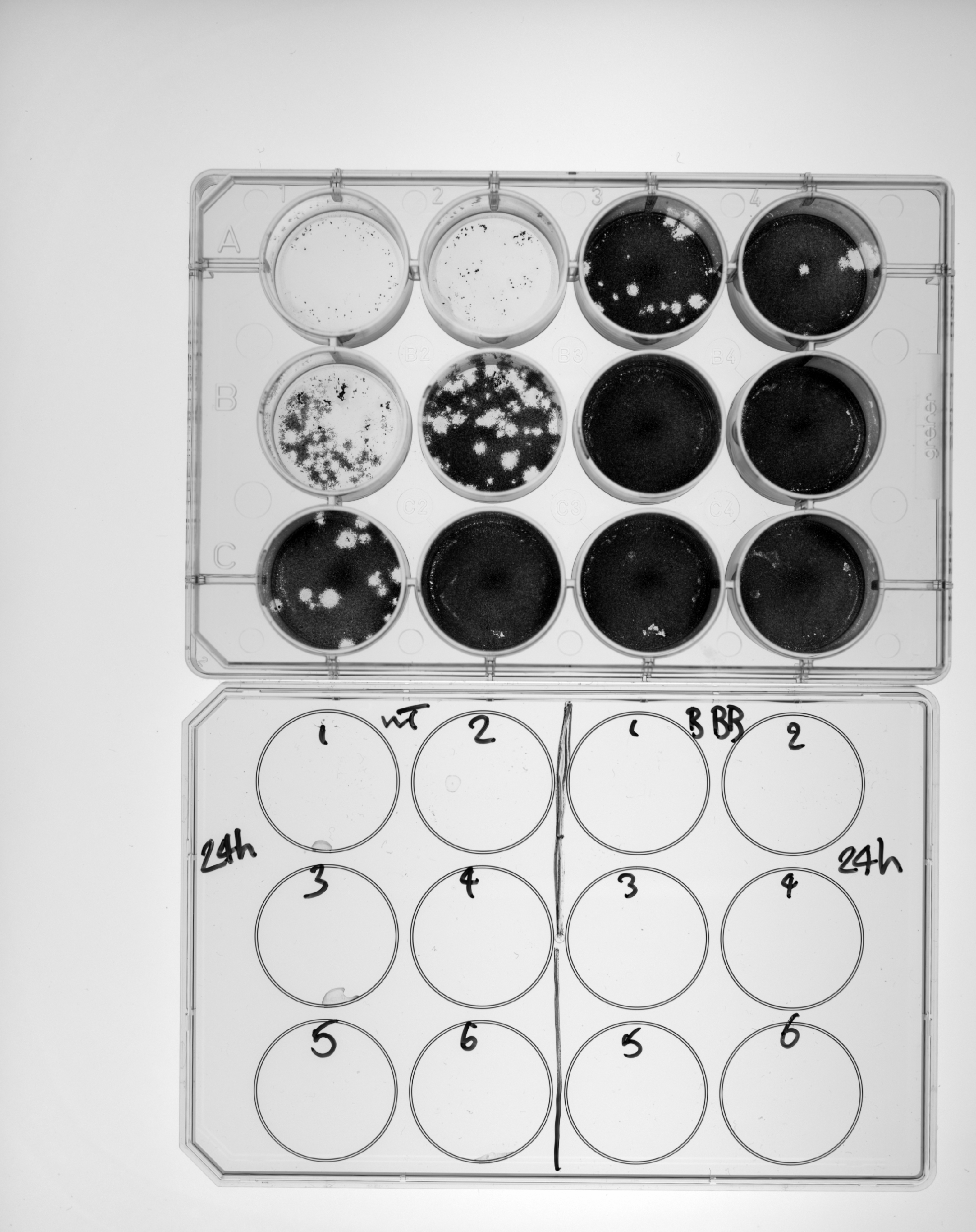

Supplement: Supplementary file 5 — Source data Fig. 3 [file 44318_2026_814_MOESM5_ESM.zip › Figure 3/Figure 3A/25.11.25 WT_BBB 24h representative plaques.tiff]

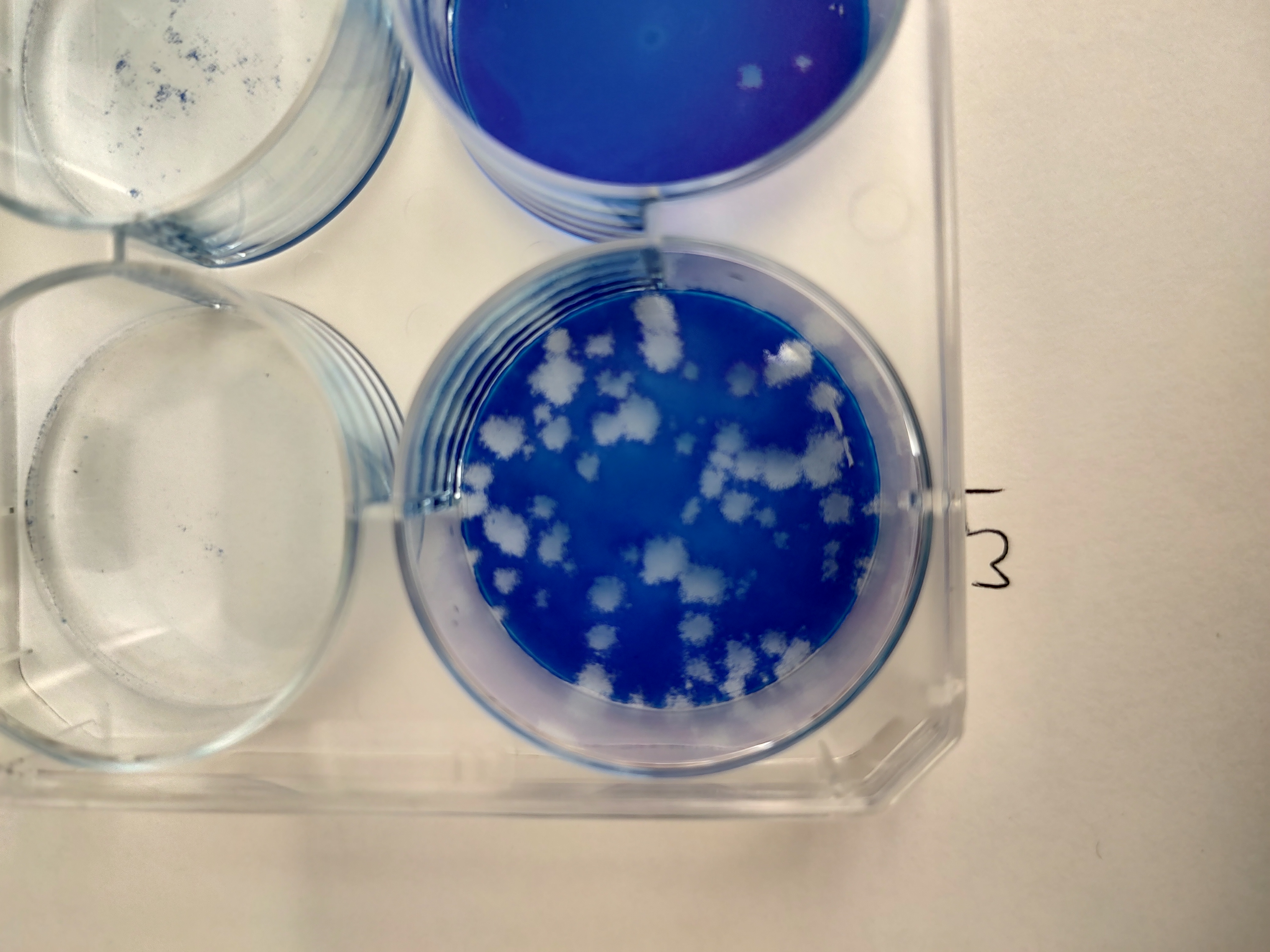

Supplement: Supplementary file 5 — Source data Fig. 3 [file 44318_2026_814_MOESM5_ESM.zip › Figure 3/Figure 3A/WT.jpg]

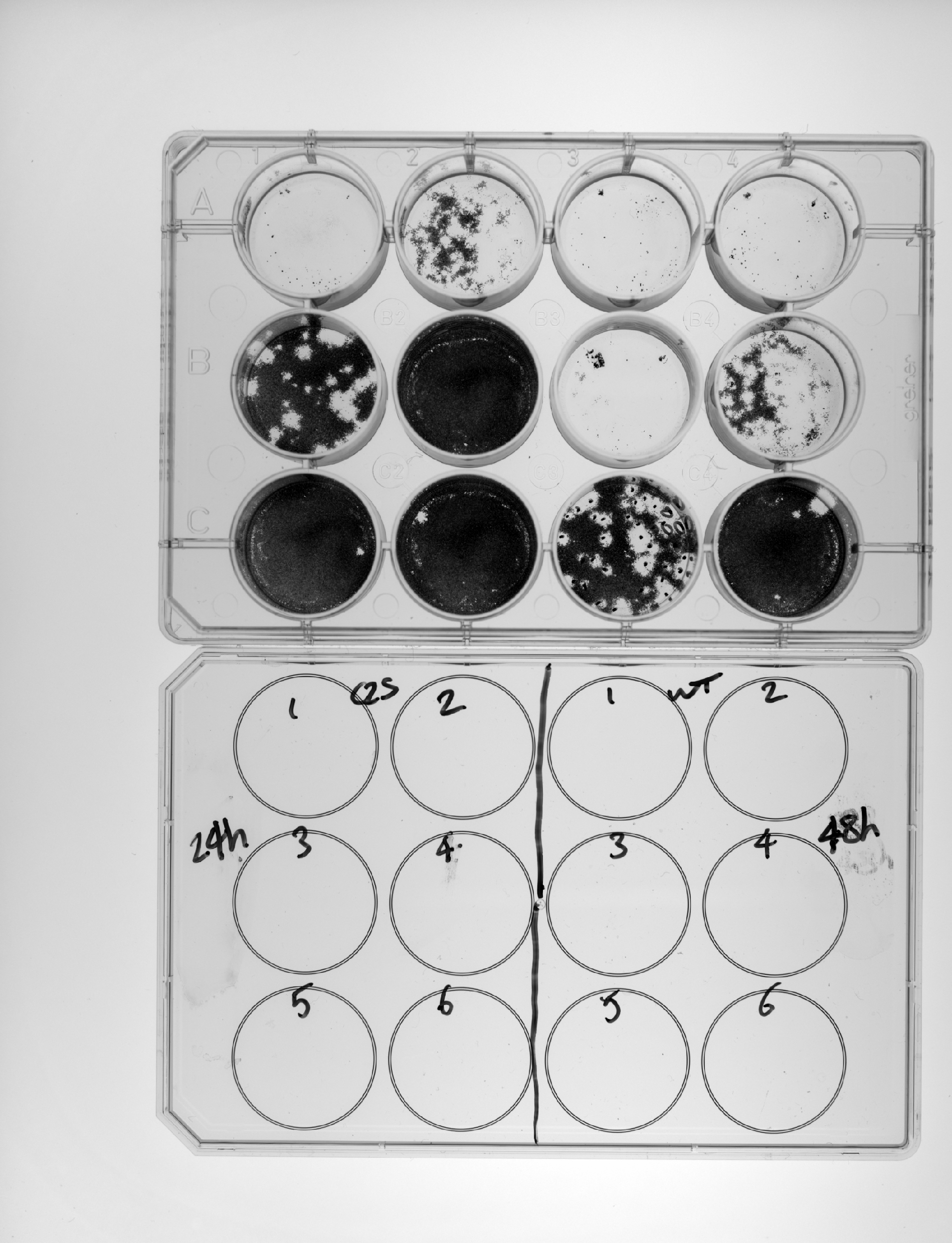

Supplement: Supplementary file 5 — Source data Fig. 3 [file 44318_2026_814_MOESM5_ESM.zip › Figure 3/Figure 3A/25.11.25 CSLow 24 h representative plaque.tiff]

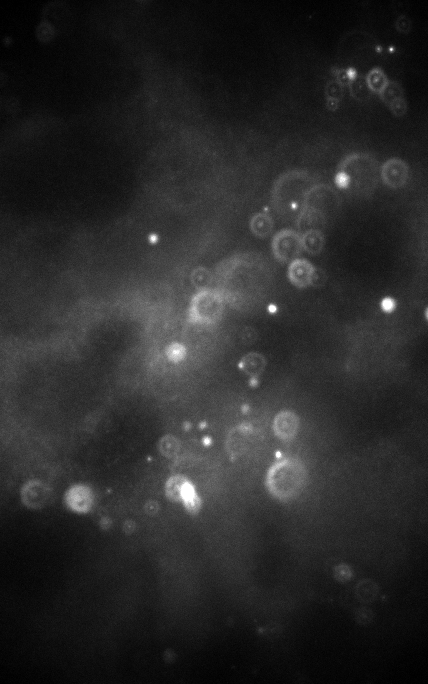

Supplement: Supplementary file 5 — Source data Fig. 3 [file 44318_2026_814_MOESM5_ESM.zip › Figure 3/Figure 3D/originals/2024_11_21_smFISH_S67A_2024_11_21_MA104-eGFP_C2S-RV_6hpi_FISH-VP1-P3-2_combined_0C.tif]

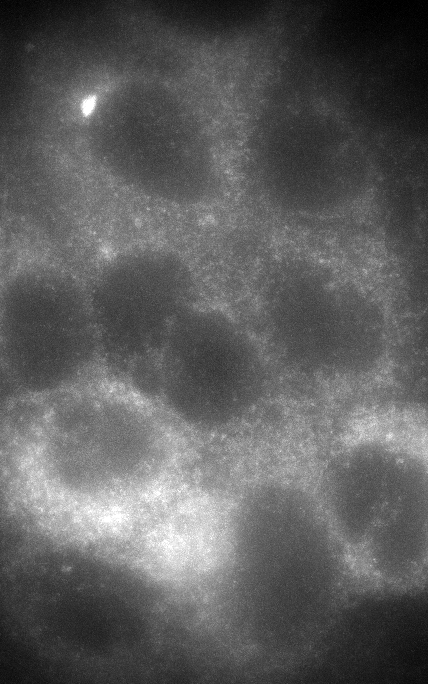

Supplement: Supplementary file 5 — Source data Fig. 3 [file 44318_2026_814_MOESM5_ESM.zip › Figure 3/Figure 3D/originals/C1-MAX_2024_11_21_smFISH_S67A_2024_11_21_MA104-eGFP_FISH-VP1-P3-2_combined_0C.tif]

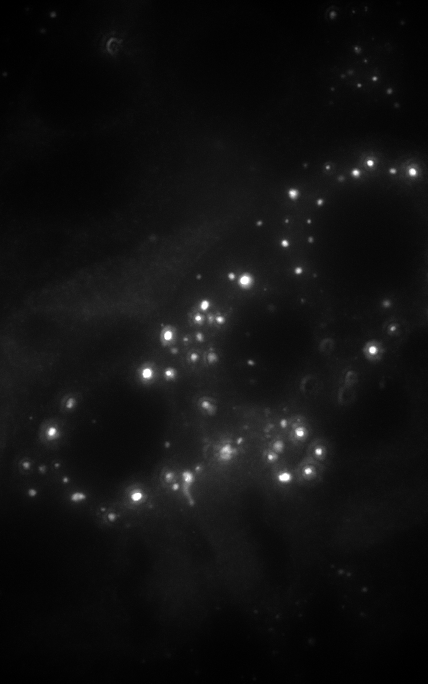

Supplement: Supplementary file 5 — Source data Fig. 3 [file 44318_2026_814_MOESM5_ESM.zip › Figure 3/Figure 3D/originals/C1-MAX_2024_11_21_smFISH_S67A_2024_11_21_MA104-eGFP_C2S-RV_6hpi_FISH-VP1-P3-2_combined_0C.tif]

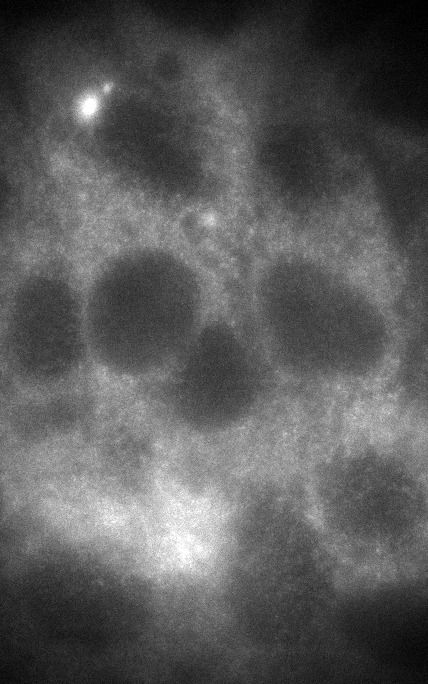

Supplement: Supplementary file 5 — Source data Fig. 3 [file 44318_2026_814_MOESM5_ESM.zip › Figure 3/Figure 3D/originals/2024_11_21_smFISH_S67A_2024_11_21_MA104-eGFP_FISH-VP1-P3-2_combined_0C.tif]

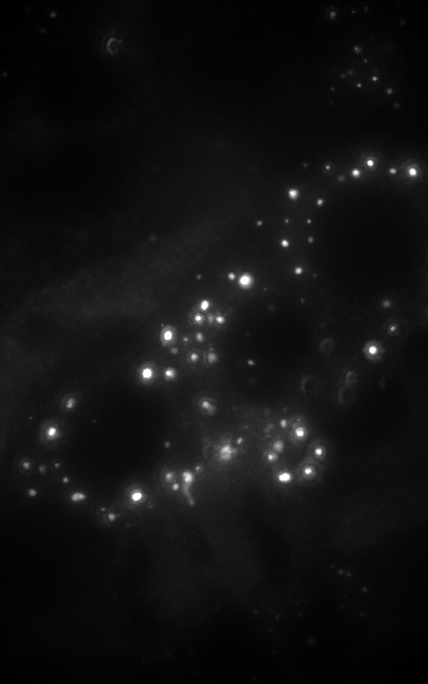

Supplement: Supplementary file 5 — Source data Fig. 3 [file 44318_2026_814_MOESM5_ESM.zip › Figure 3/Figure 3D/originals/MAX_2024_11_21_smFISH_S67A_2024_11_21_MA104-eGFP_C2S-RV_6hpi_FISH-VP1-P3-2_combined_0C.tif]

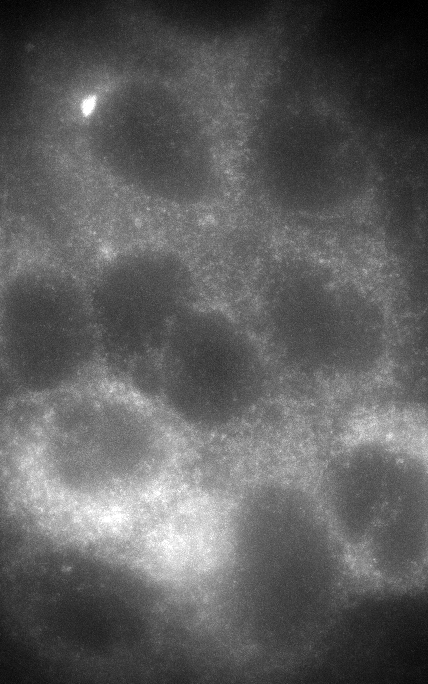

Supplement: Supplementary file 5 — Source data Fig. 3 [file 44318_2026_814_MOESM5_ESM.zip › Figure 3/Figure 3D/originals/MAX_2024_11_21_smFISH_S67A_2024_11_21_MA104-eGFP_FISH-VP1-P3-2_combined_0C.tif]

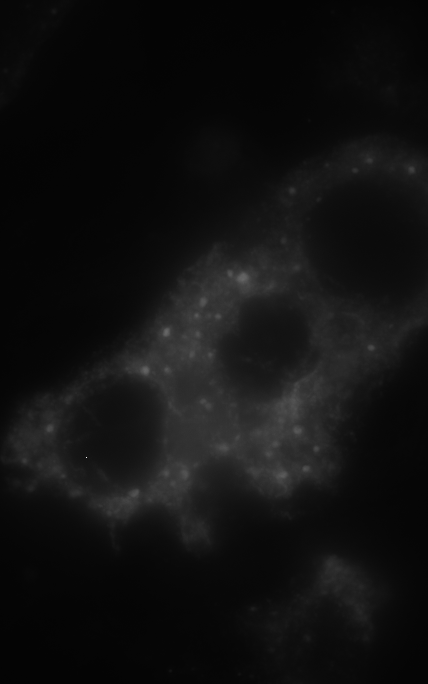

Supplement: Supplementary file 5 — Source data Fig. 3 [file 44318_2026_814_MOESM5_ESM.zip › Figure 3/Figure 3D/originals/C2-MAX_2024_11_21_smFISH_S67A_2024_11_21_MA104-eGFP_C2S-RV_6hpi_FISH-VP1-P3-2_combined_0C.tif]

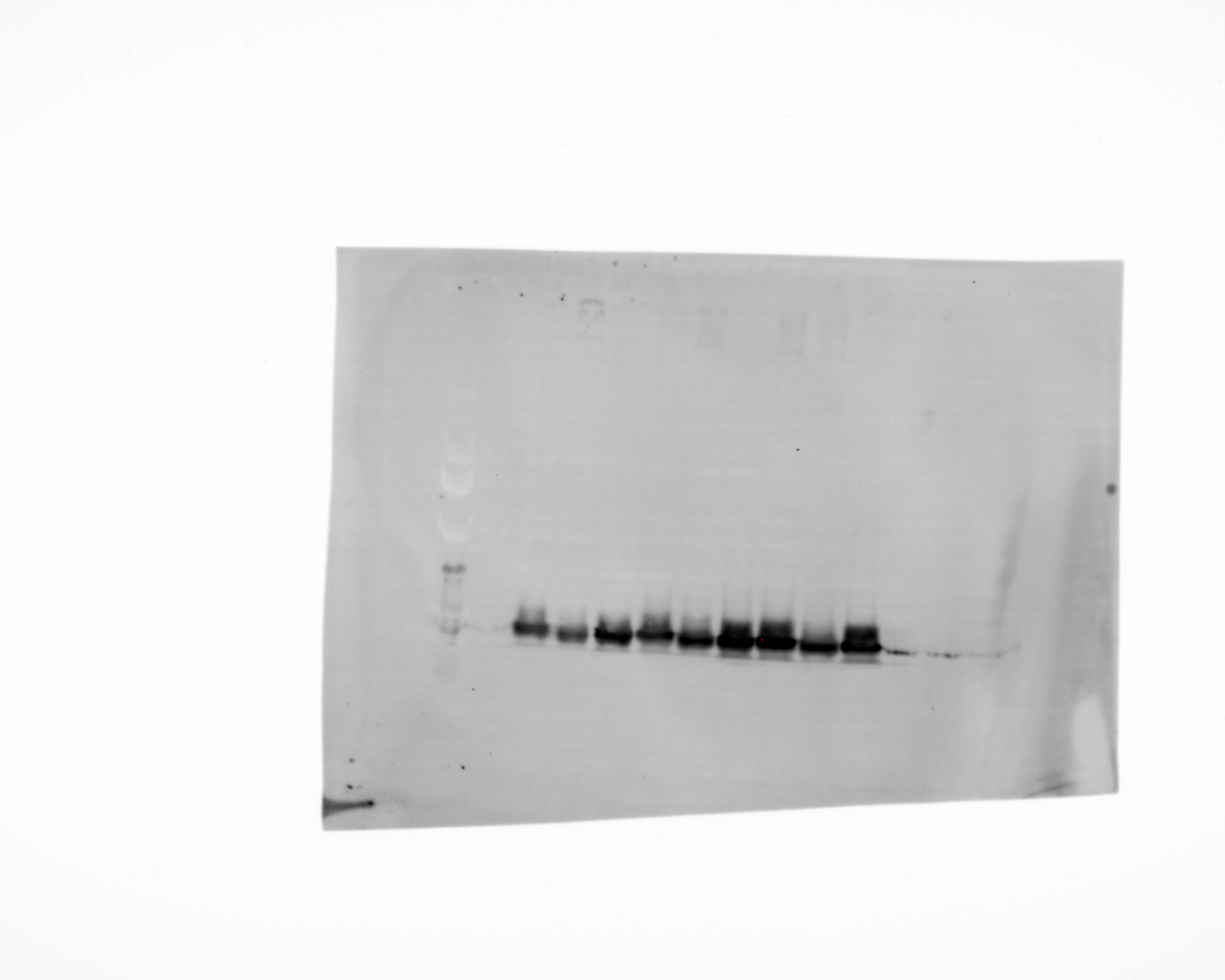

Supplement: Supplementary file 6 — Source data Fig. 4 [file 44318_2026_814_MOESM6_ESM.zip › Figure_4(1)/Figure 4 D/8 h.p.i. and 12 h.p.i.-NSP5/admi 2022-12-05 17h29m25s(DyLight 800).tif]

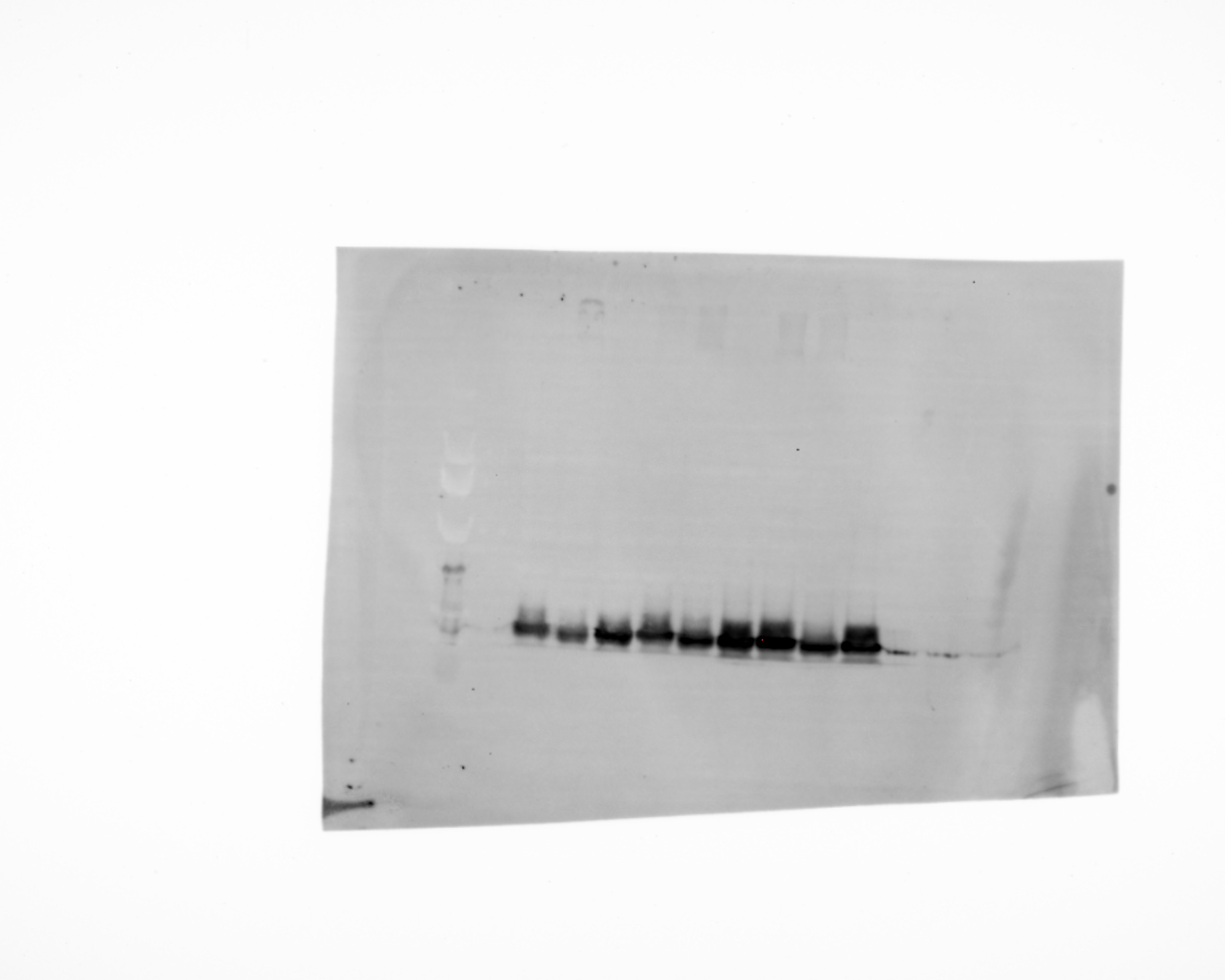

Supplement: Supplementary file 6 — Source data Fig. 4 [file 44318_2026_814_MOESM6_ESM.zip › Figure_4(1)/Figure 4 D/8 h.p.i. and 12 h.p.i.-NSP5/admi 2022-12-05 17h29m25s(DyLight 800).jpg]

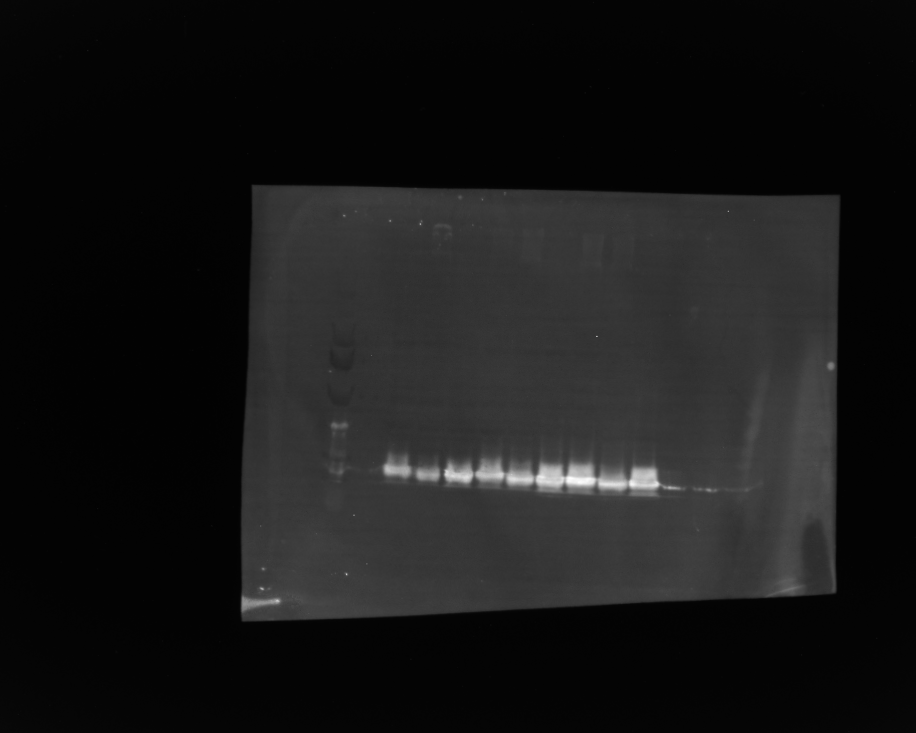

Supplement: Supplementary file 6 — Source data Fig. 4 [file 44318_2026_814_MOESM6_ESM.zip › Figure_4(1)/Figure 4 D/8 h.p.i. and 12 h.p.i.-NSP5/admi 2022-12-05 17h29m25s(DyLight 800).raw16.tif]

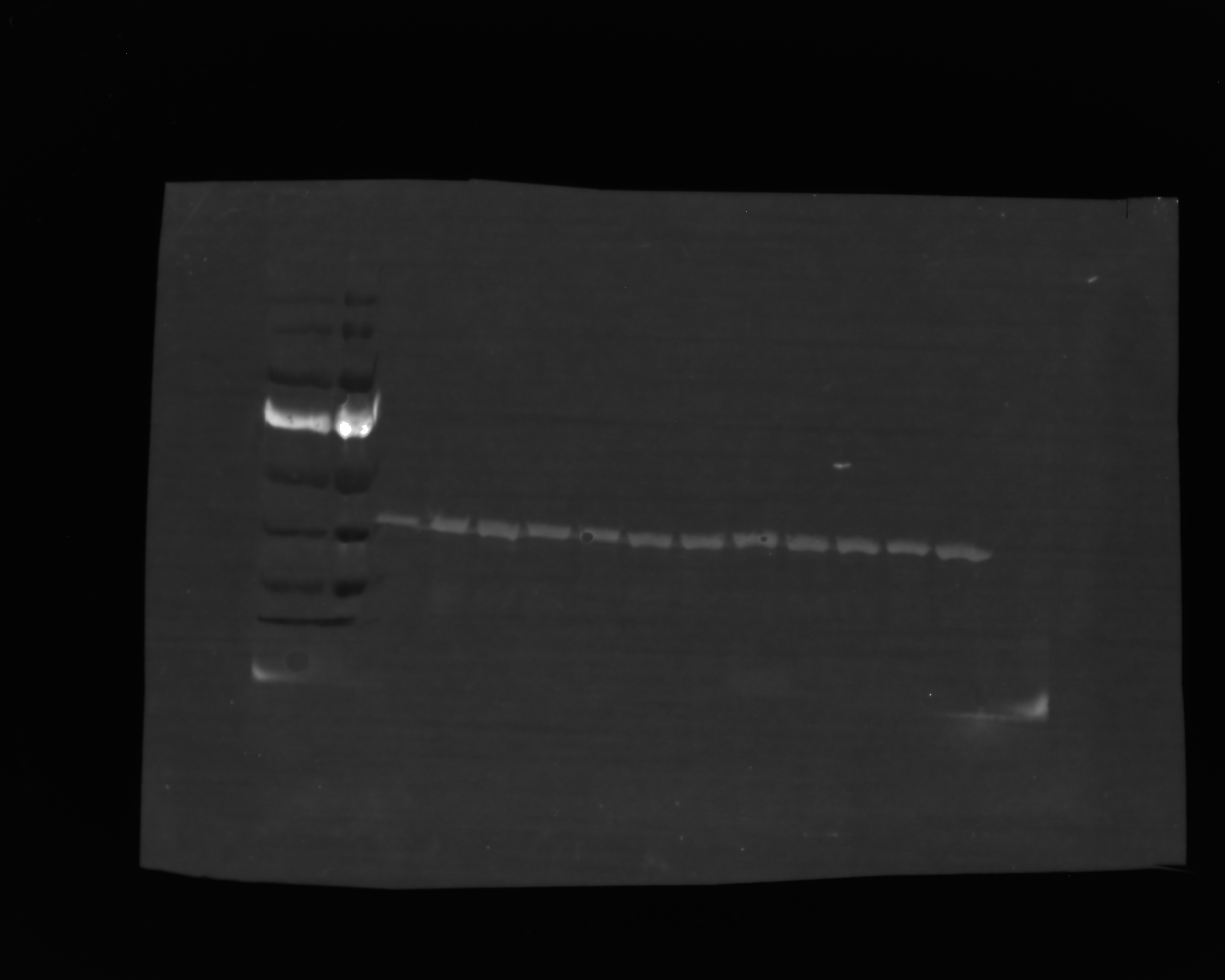

Supplement: Supplementary file 6 — Source data Fig. 4 [file 44318_2026_814_MOESM6_ESM.zip › Figure_4(1)/Figure 4 D/4 h.p.i. and 6 h.p.i.-beta-actin/admi 2022-11-28 17h41m35s(Rhodamine).raw16.tif]

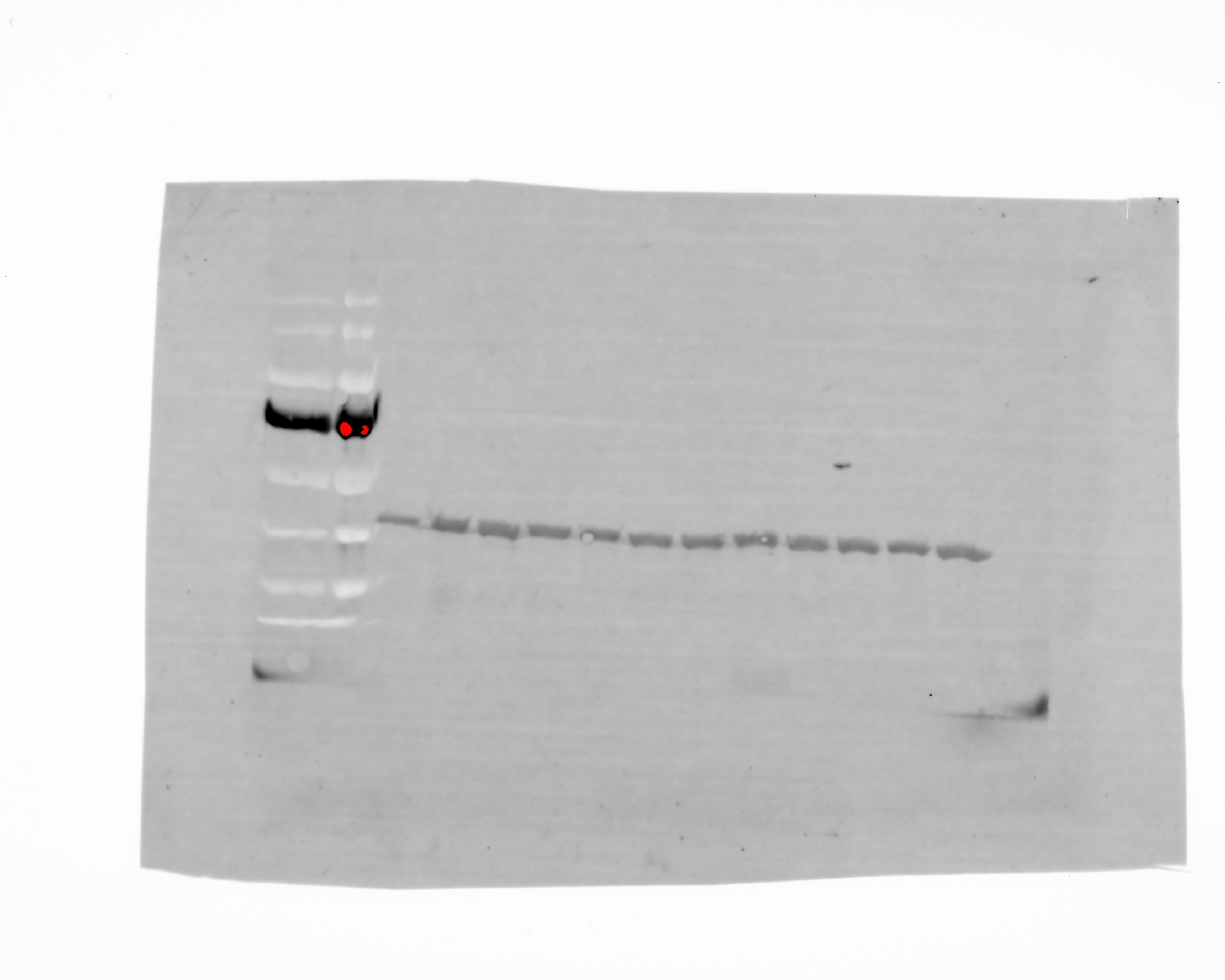

Supplement: Supplementary file 6 — Source data Fig. 4 [file 44318_2026_814_MOESM6_ESM.zip › Figure_4(1)/Figure 4 D/4 h.p.i. and 6 h.p.i.-beta-actin/admi 2022-11-28 17h41m35s(Rhodamine).jpg]

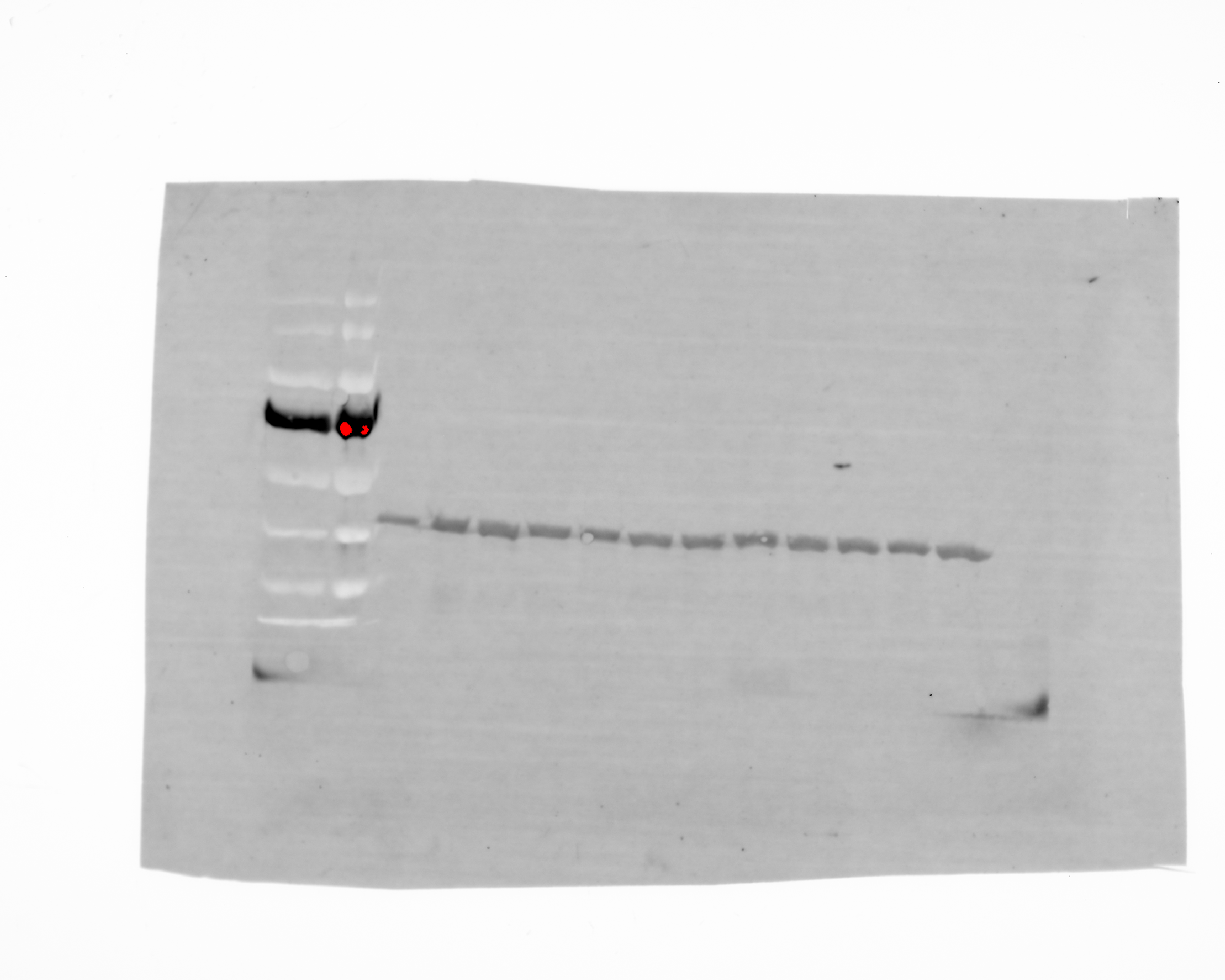

Supplement: Supplementary file 6 — Source data Fig. 4 [file 44318_2026_814_MOESM6_ESM.zip › Figure_4(1)/Figure 4 D/4 h.p.i. and 6 h.p.i.-beta-actin/admi 2022-11-28 17h41m35s(Rhodamine).tif]

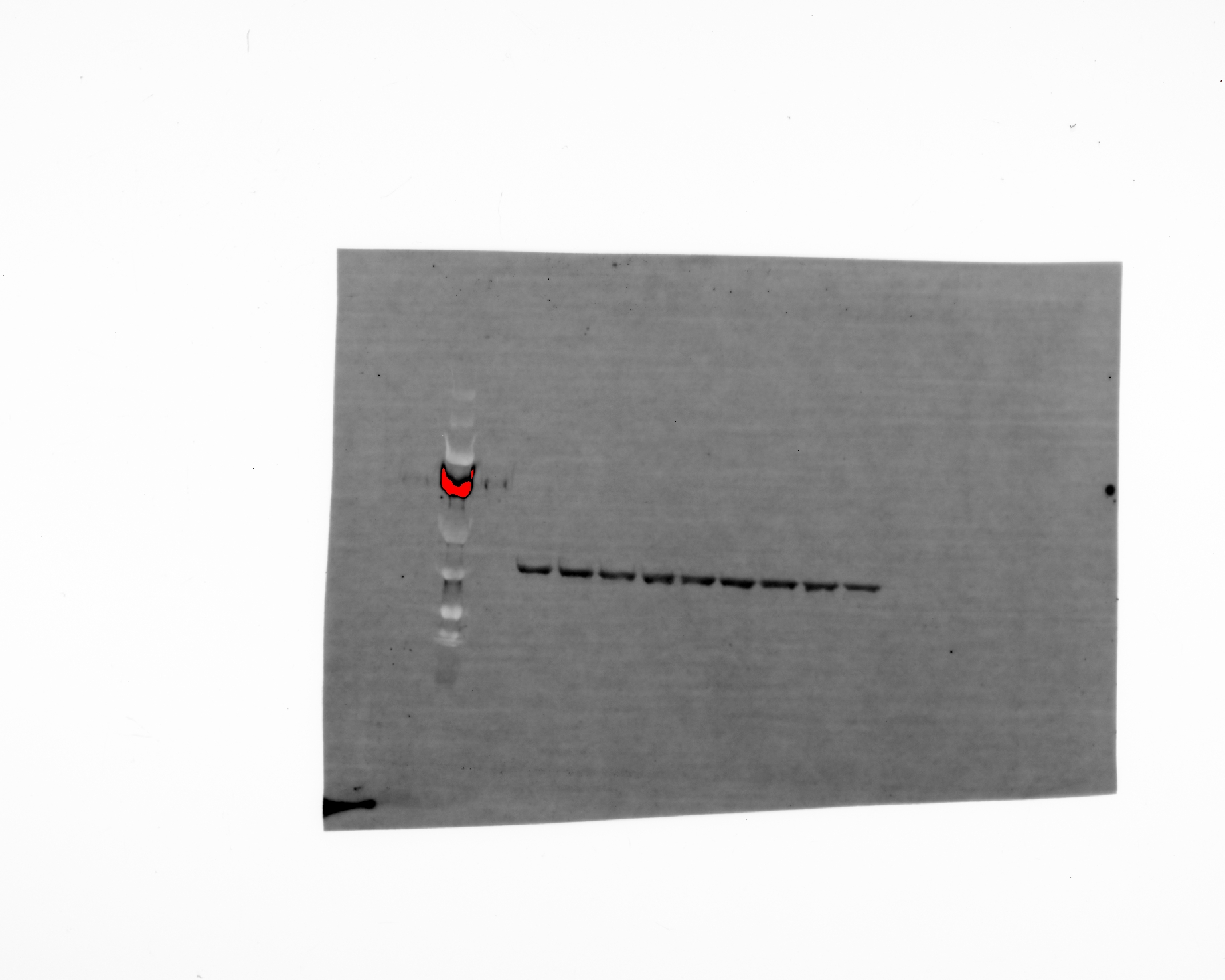

Supplement: Supplementary file 6 — Source data Fig. 4 [file 44318_2026_814_MOESM6_ESM.zip › Figure_4(1)/Figure 4 D/8 h.p.i. and 12 h.p.i.-beta-actin/admi 2022-12-05 17h33m04s(Rhodamine).jpg]

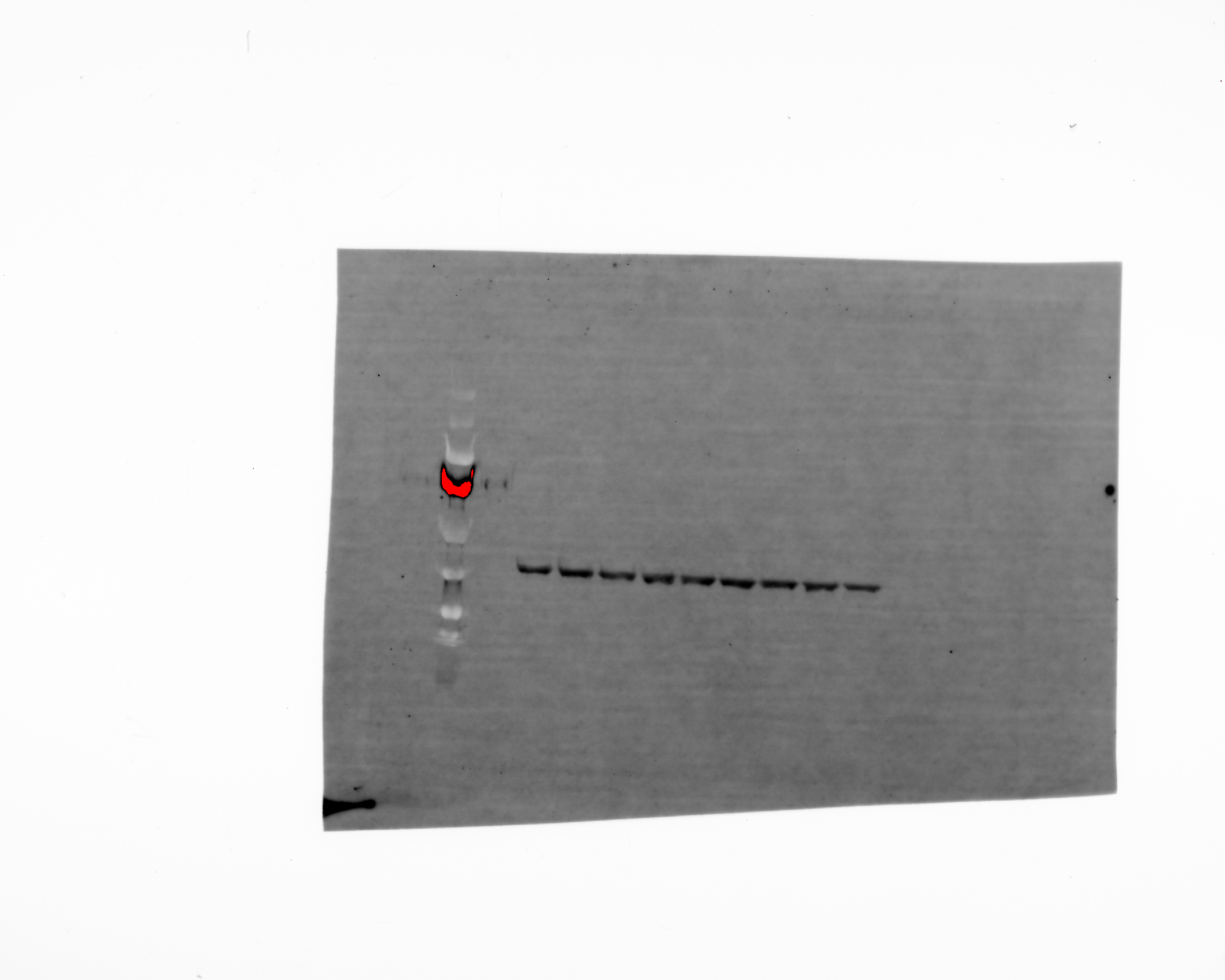

Supplement: Supplementary file 6 — Source data Fig. 4 [file 44318_2026_814_MOESM6_ESM.zip › Figure_4(1)/Figure 4 D/8 h.p.i. and 12 h.p.i.-beta-actin/admi 2022-12-05 17h33m04s(Rhodamine).tif]

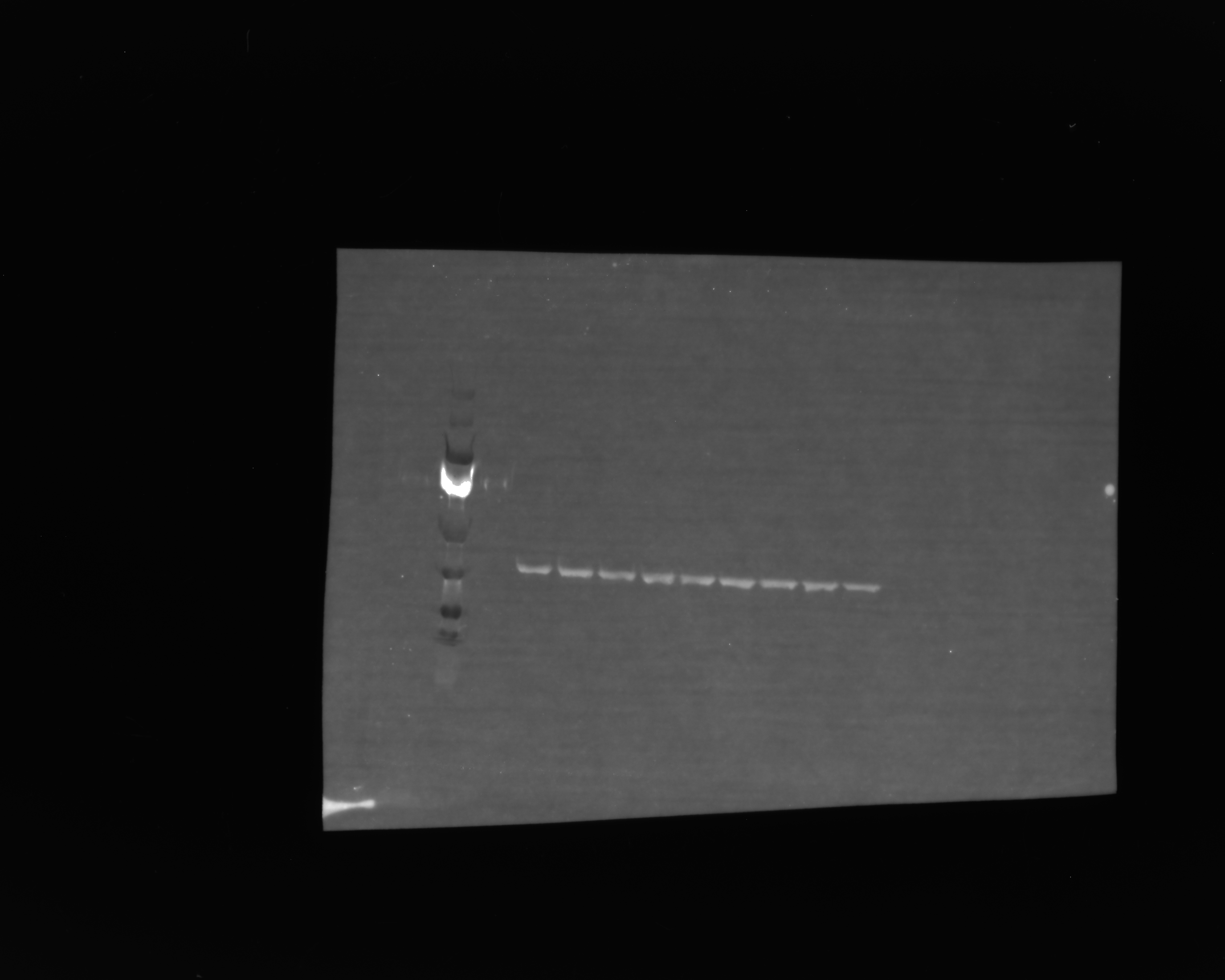

Supplement: Supplementary file 6 — Source data Fig. 4 [file 44318_2026_814_MOESM6_ESM.zip › Figure_4(1)/Figure 4 D/8 h.p.i. and 12 h.p.i.-beta-actin/admi 2022-12-05 17h33m04s(Rhodamine).raw16.tif]

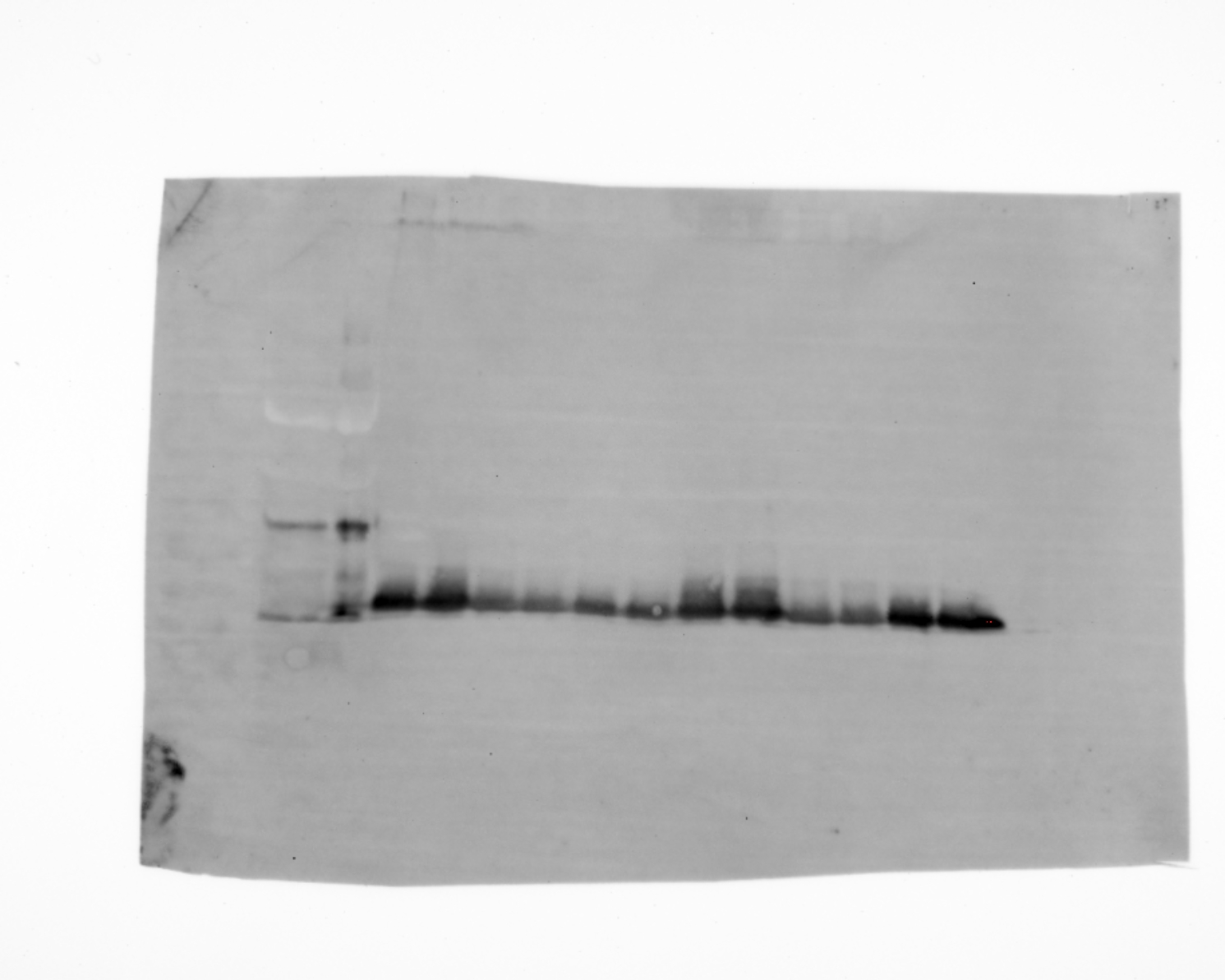

Supplement: Supplementary file 6 — Source data Fig. 4 [file 44318_2026_814_MOESM6_ESM.zip › Figure_4(1)/Figure 4 D/4 h.p.i. and 6 h.p.i.-NSP5/ChemiDoc Images 2022-11-28_17.42.03/admi 2022-11-28 17h38m55s(DyLight 800).jpg]

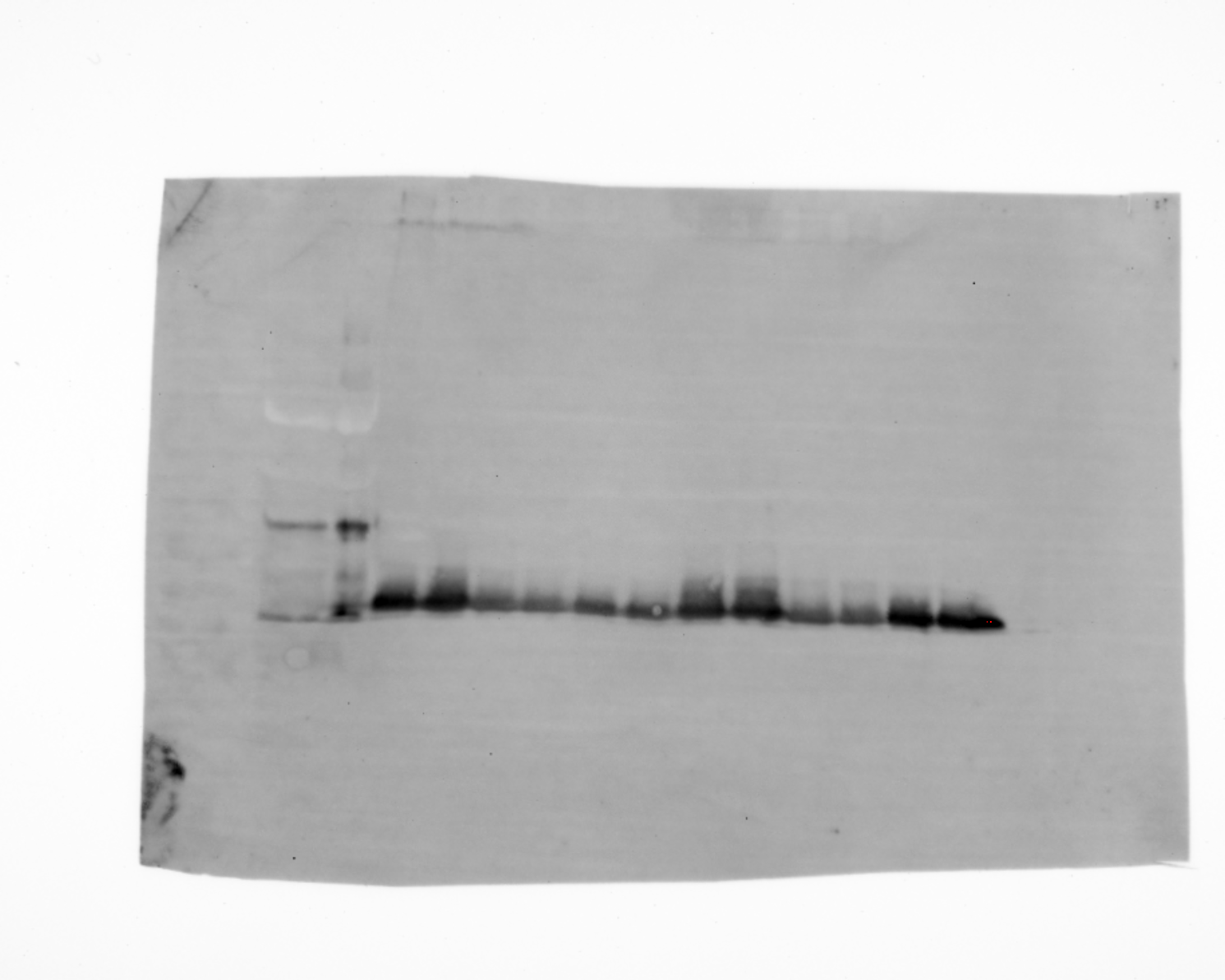

Supplement: Supplementary file 6 — Source data Fig. 4 [file 44318_2026_814_MOESM6_ESM.zip › Figure_4(1)/Figure 4 D/4 h.p.i. and 6 h.p.i.-NSP5/ChemiDoc Images 2022-11-28_17.42.03/admi 2022-11-28 17h38m55s(DyLight 800).tif]

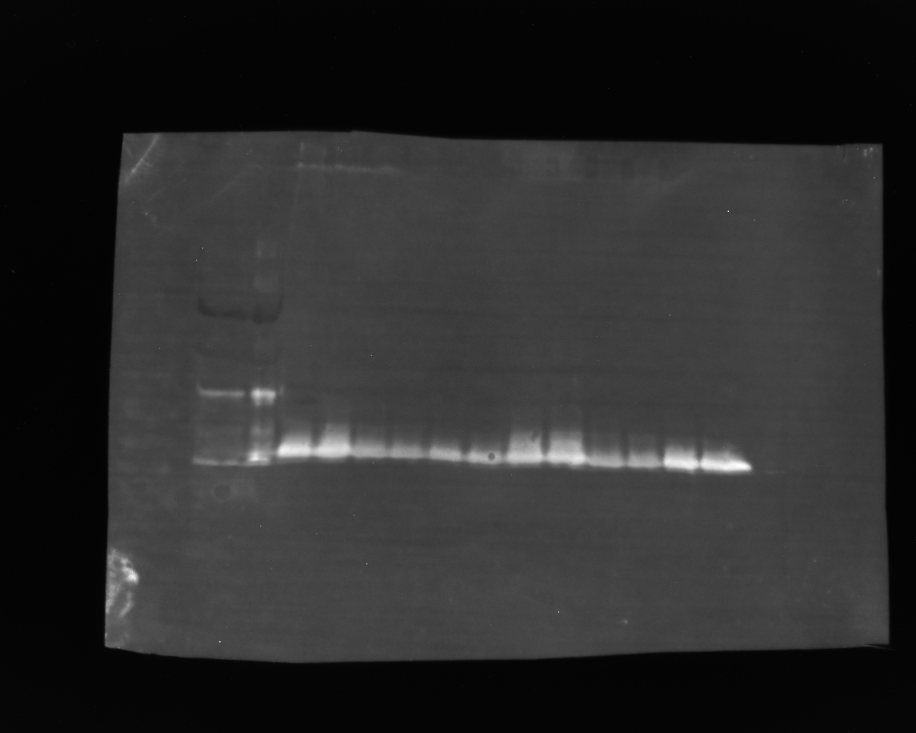

Supplement: Supplementary file 6 — Source data Fig. 4 [file 44318_2026_814_MOESM6_ESM.zip › Figure_4(1)/Figure 4 D/4 h.p.i. and 6 h.p.i.-NSP5/ChemiDoc Images 2022-11-28_17.42.03/admi 2022-11-28 17h38m55s(DyLight 800).raw16.tif]

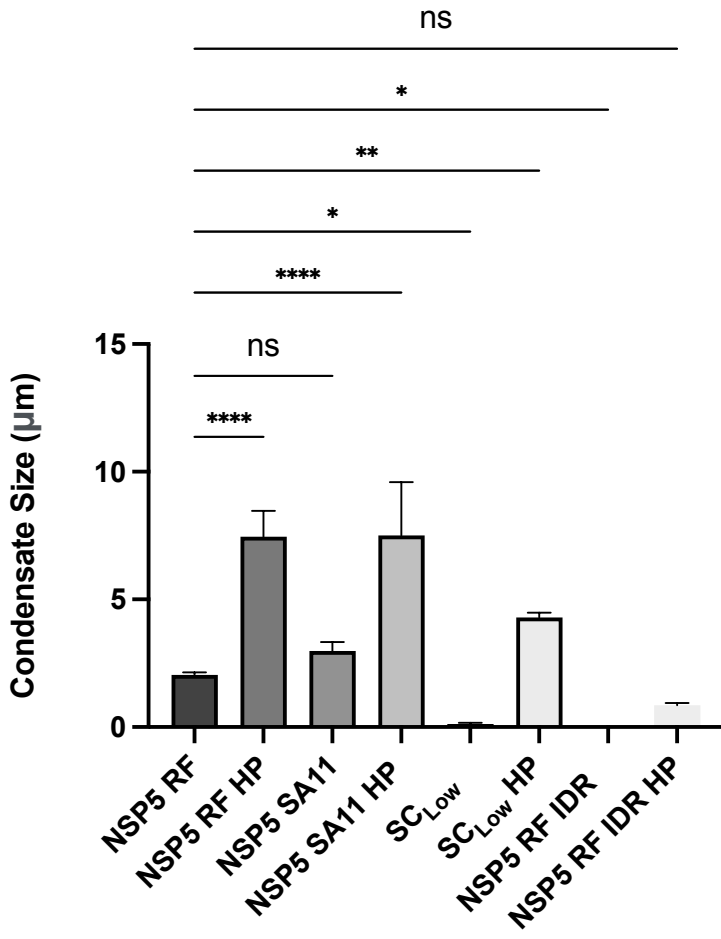

Supplement: Supplementary file 7 — Source data Fig. 5 [file 44318_2026_814_MOESM7_ESM.zip › Figure 5 Raw data/Figure 5 B/Copy of Copy of Column - Average Size - pair comparison1.pdf]

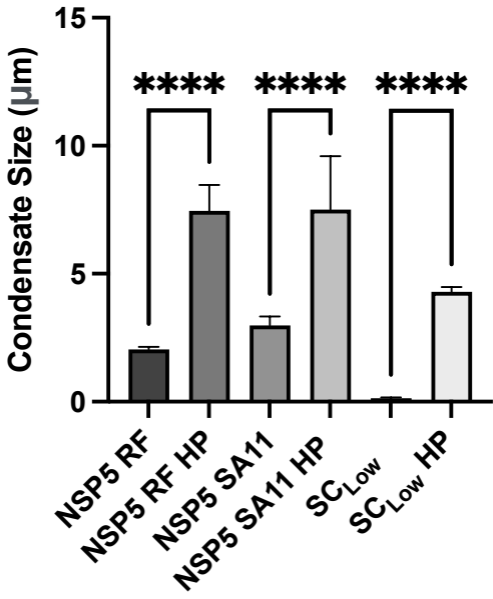

Supplement: Supplementary file 7 — Source data Fig. 5 [file 44318_2026_814_MOESM7_ESM.zip › Figure 5 Raw data/Figure 5 B/Copy of Column - Average Size - pair comparison.pdf]

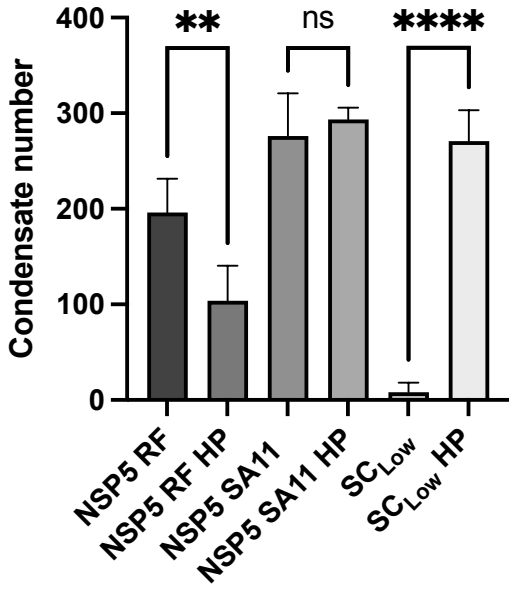

Supplement: Supplementary file 7 — Source data Fig. 5 [file 44318_2026_814_MOESM7_ESM.zip › Figure 5 Raw data/Figure 5 B/Column - Count.pdf]

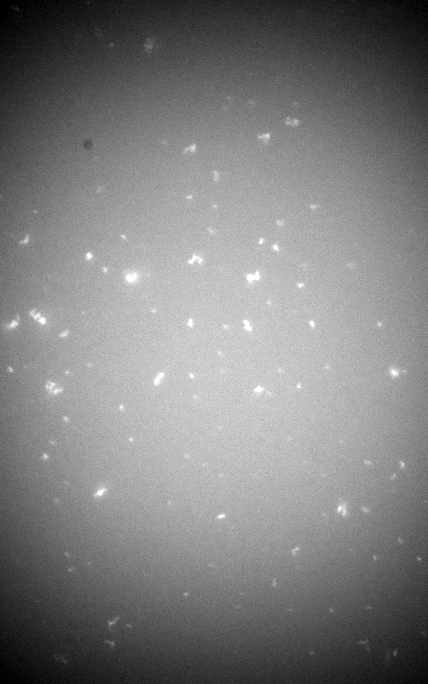

Supplement: Supplementary file 7 — Source data Fig. 5 [file 44318_2026_814_MOESM7_ESM.zip › Figure 5 Raw data/Figure 5 A/SClow.tif]

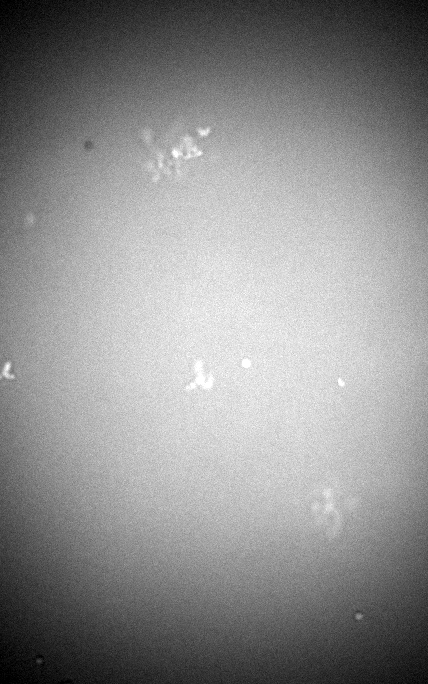

Supplement: Supplementary file 7 — Source data Fig. 5 [file 44318_2026_814_MOESM7_ESM.zip › Figure 5 Raw data/Figure 5 A/SClow ATP.tif]

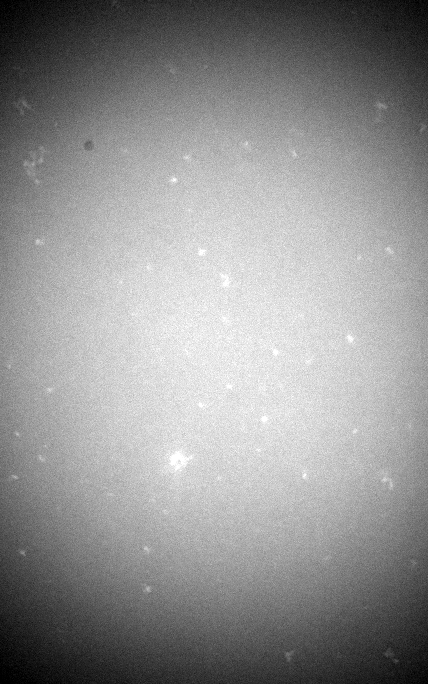

Supplement: Supplementary file 7 — Source data Fig. 5 [file 44318_2026_814_MOESM7_ESM.zip › Figure 5 Raw data/Figure 5 A/BBB.tif]

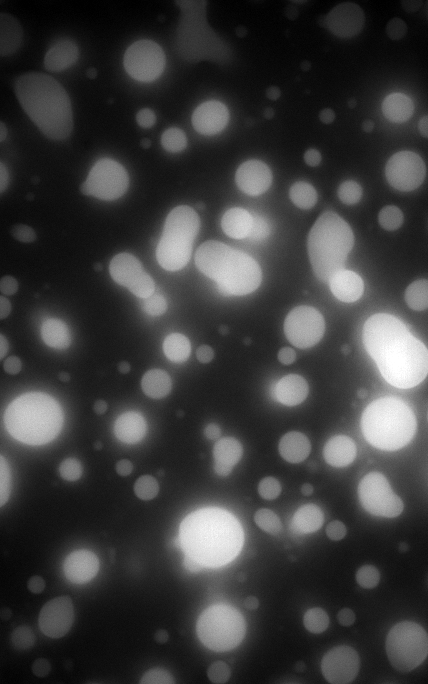

Supplement: Supplementary file 7 — Source data Fig. 5 [file 44318_2026_814_MOESM7_ESM.zip › Figure 5 Raw data/Figure 5 A/NSP5 RF HP.tif]

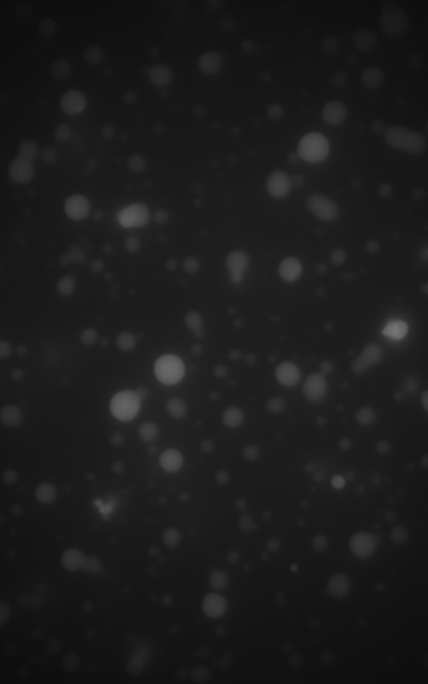

Supplement: Supplementary file 7 — Source data Fig. 5 [file 44318_2026_814_MOESM7_ESM.zip › Figure 5 Raw data/Figure 5 A/SClow HP.tif]

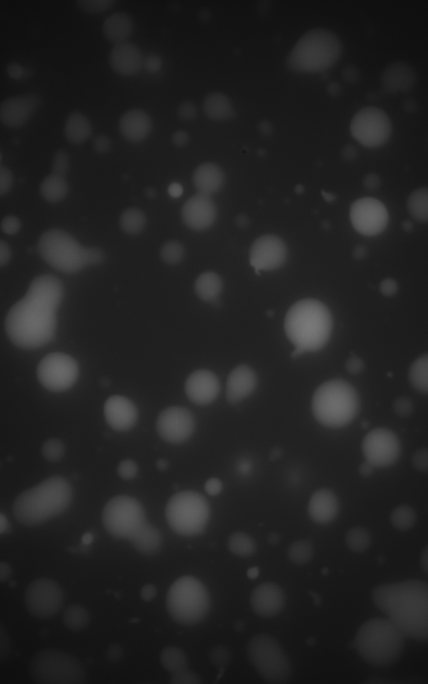

Supplement: Supplementary file 7 — Source data Fig. 5 [file 44318_2026_814_MOESM7_ESM.zip › Figure 5 Raw data/Figure 5 A/NSP5 SA11 HP.tif]

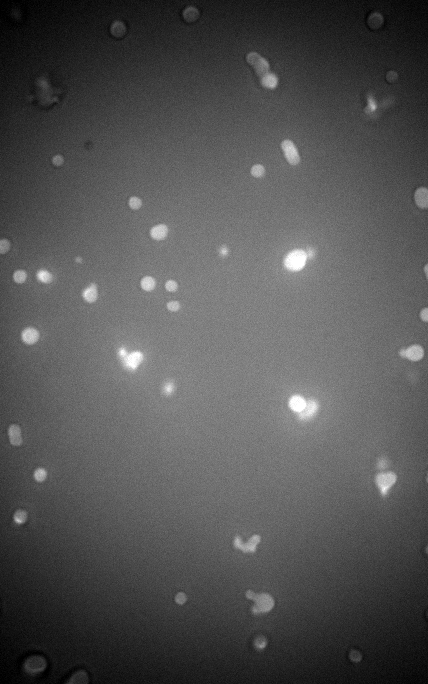

Supplement: Supplementary file 7 — Source data Fig. 5 [file 44318_2026_814_MOESM7_ESM.zip › Figure 5 Raw data/Figure 5 A/SClow ATP CKII.tif]

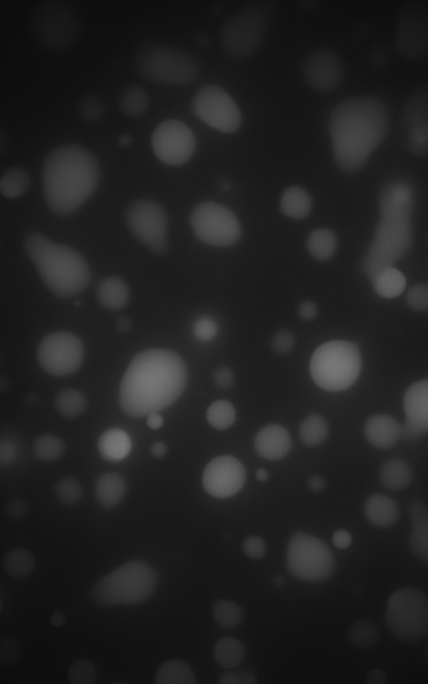

Supplement: Supplementary file 7 — Source data Fig. 5 [file 44318_2026_814_MOESM7_ESM.zip › Figure 5 Raw data/Figure 5 A/BBB HP.tif]

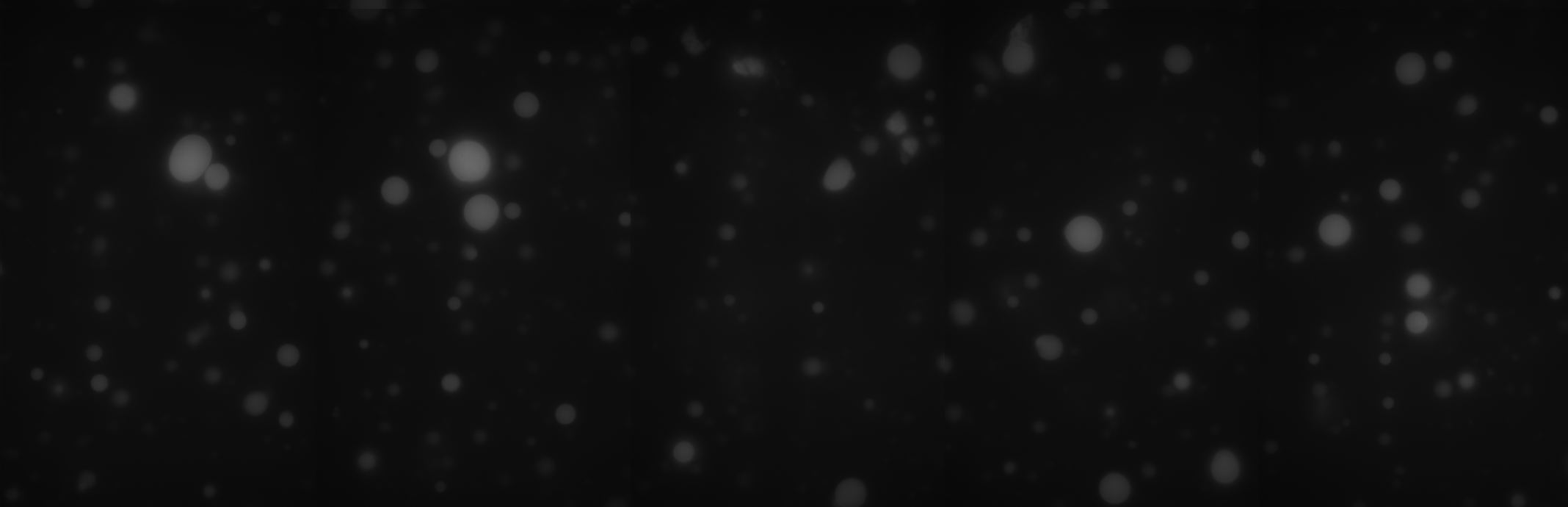

Supplement: Supplementary file 7 — Source data Fig. 5 [file 44318_2026_814_MOESM7_ESM.zip › Figure 5 Raw data/Figure 5 B/RF HP scans/20uM-NSP2-RF-A488_20uM-NSP5-HP-scan-4.tif]

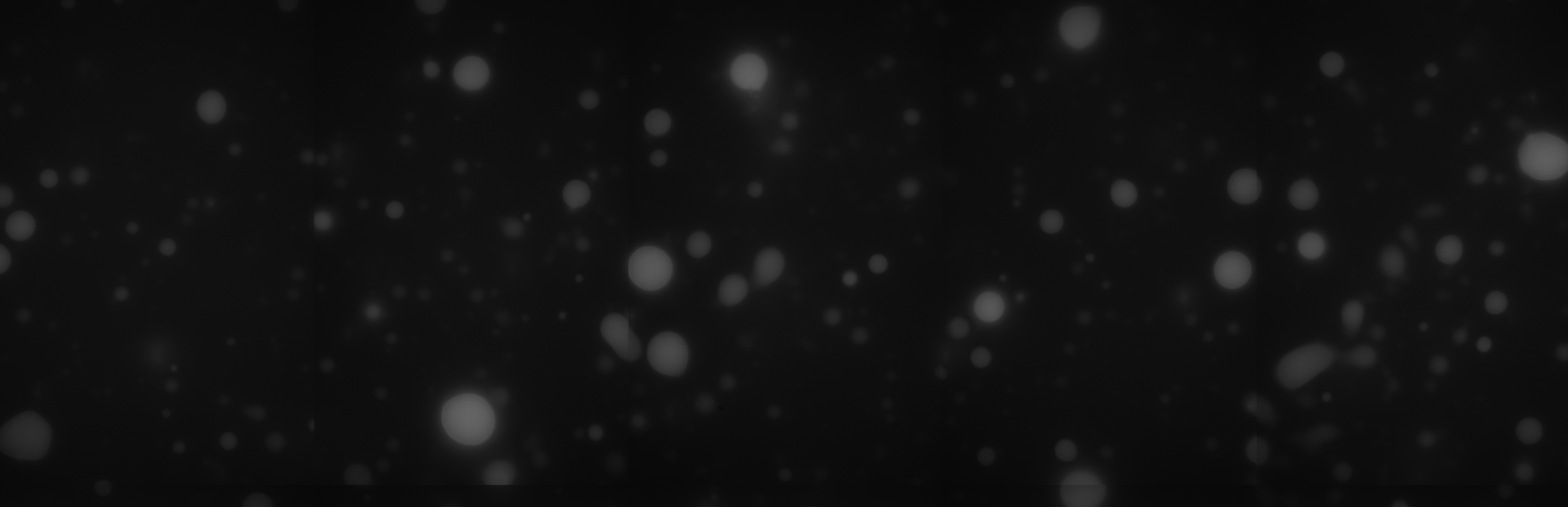

Supplement: Supplementary file 7 — Source data Fig. 5 [file 44318_2026_814_MOESM7_ESM.zip › Figure 5 Raw data/Figure 5 B/RF HP scans/20uM-NSP2-RF-A488_20uM-NSP5-HP-scan-2.tif]

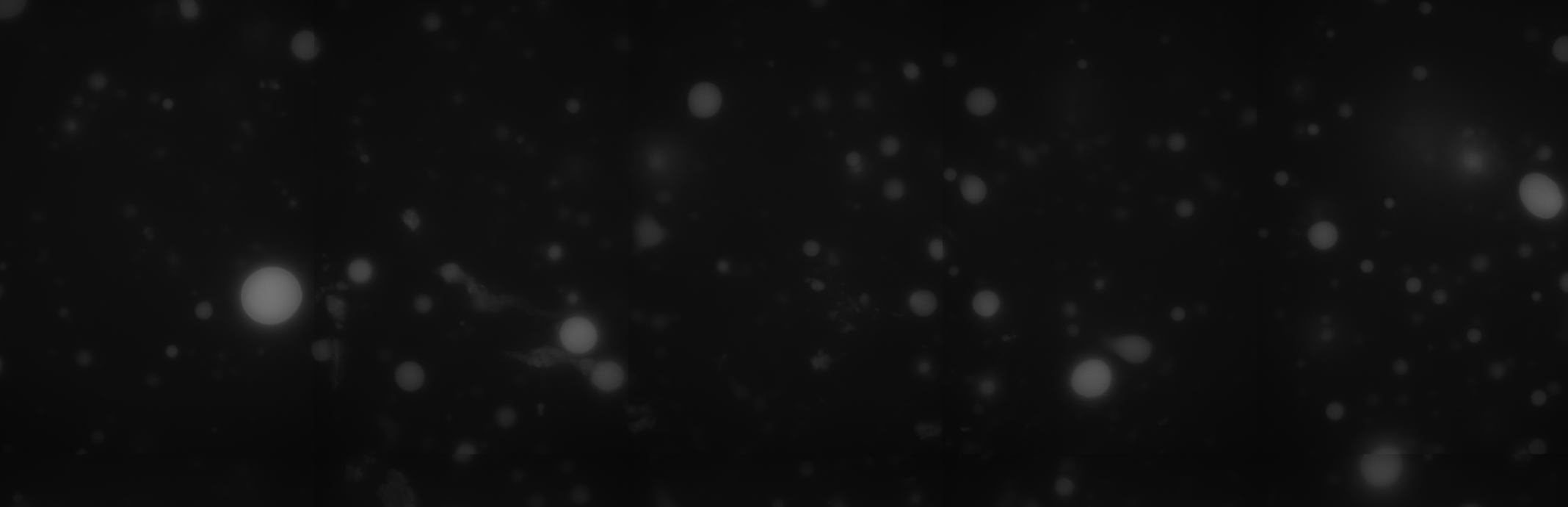

Supplement: Supplementary file 7 — Source data Fig. 5 [file 44318_2026_814_MOESM7_ESM.zip › Figure 5 Raw data/Figure 5 B/RF HP scans/20uM-NSP2-RF-A488_20uM-NSP5-HP-scan-3.tif]

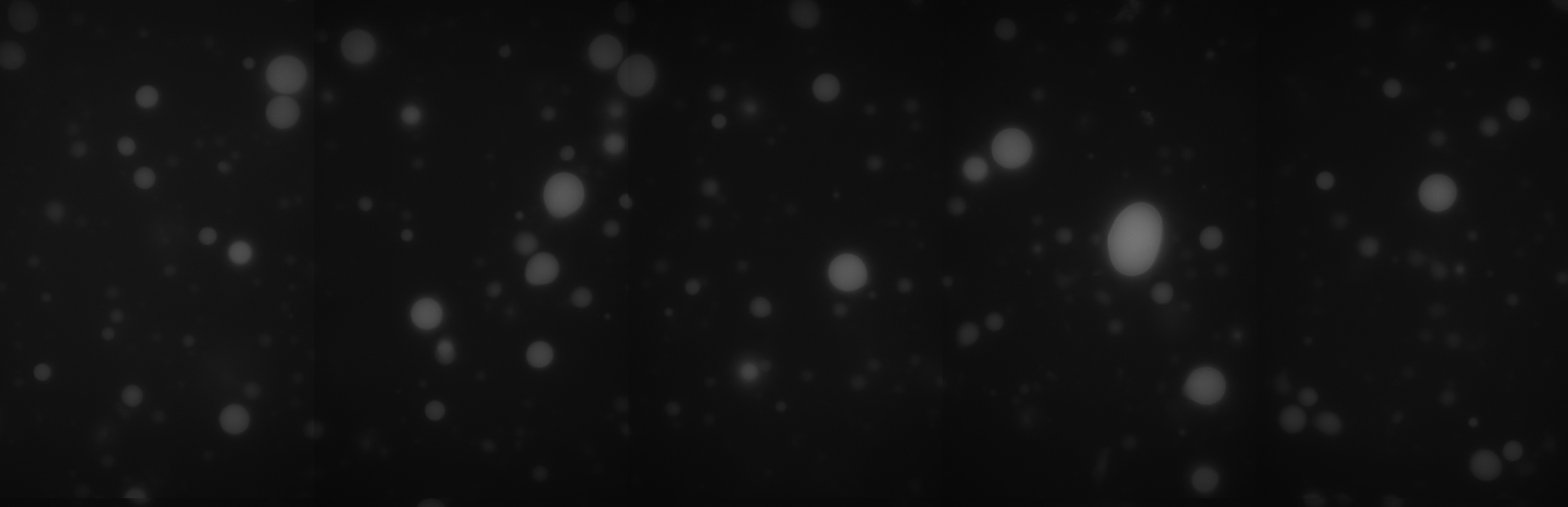

Supplement: Supplementary file 7 — Source data Fig. 5 [file 44318_2026_814_MOESM7_ESM.zip › Figure 5 Raw data/Figure 5 B/RF HP scans/20uM-NSP2-RF-A488_20uM-NSP5-HP-scan-1.tif]

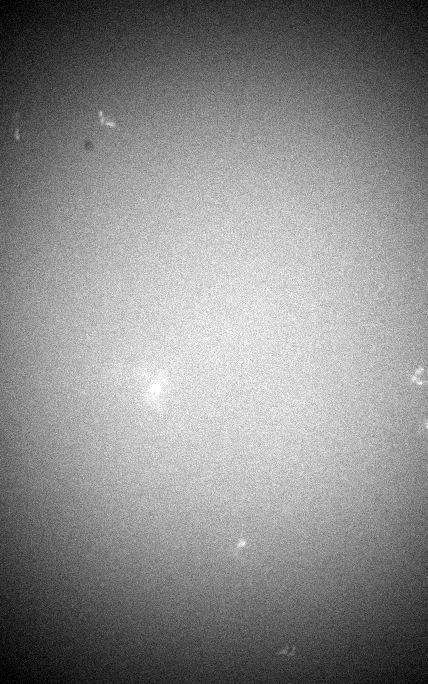

Supplement: Supplementary file 7 — Source data Fig. 5 [file 44318_2026_814_MOESM7_ESM.zip › Figure 5 Raw data/Figure 5 B/SClow ATP/Alonso_SClow_CKii_sample_25uM_NSP2_25uM_SClowi_ATP_sampleC_posXY6_channels_t1_posZ0.tif]

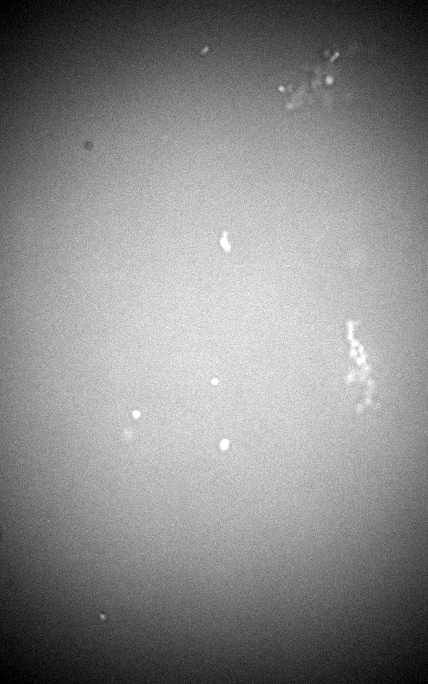

Supplement: Supplementary file 7 — Source data Fig. 5 [file 44318_2026_814_MOESM7_ESM.zip › Figure 5 Raw data/Figure 5 B/SClow ATP/Alonso_SClow_CKii_NSP2sec_sample_25uM_NSP2sec_25uM_SClow_CKii_ATP_sampleAprime_posXY5_channels_t1_posZ0.tif]

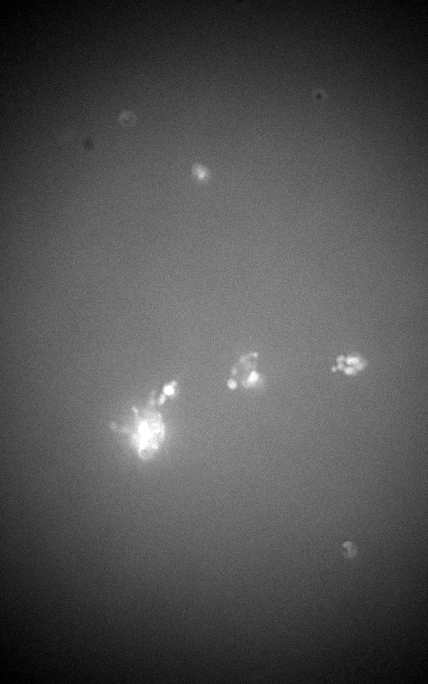

Supplement: Supplementary file 7 — Source data Fig. 5 [file 44318_2026_814_MOESM7_ESM.zip › Figure 5 Raw data/Figure 5 B/SClow ATP/Alonso_CKi_CKii_test_sample_12.5uM_NSP2_12.5uM_SClow__CKii_C_30min_time_posXY2_channels_t1_posZ0.tif]

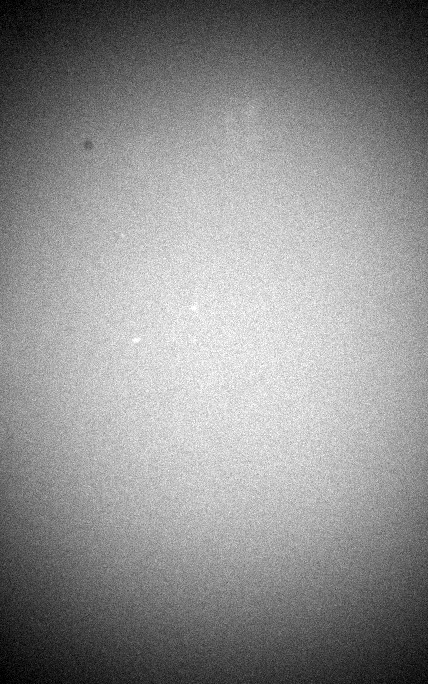

Supplement: Supplementary file 7 — Source data Fig. 5 [file 44318_2026_814_MOESM7_ESM.zip › Figure 5 Raw data/Figure 5 B/SClow ATP/Alonso_SClow_CKii_sample_25uM_NSP2_25uM_SClowi_ATP_sampleC_posXY4_channels_t1_posZ0.tif]

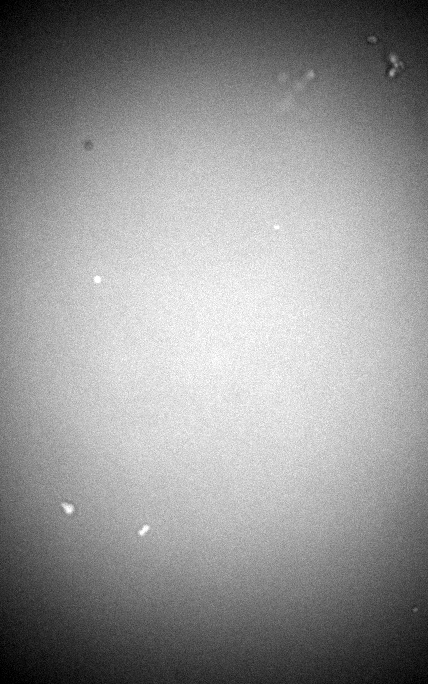

Supplement: Supplementary file 7 — Source data Fig. 5 [file 44318_2026_814_MOESM7_ESM.zip › Figure 5 Raw data/Figure 5 B/SClow ATP/Alonso_SClow_CKii_NSP2sec_sample_25uM_NSP2sec_25uM_SClow_CKii_ATP_sampleAprime_posXY7_channels_t1_posZ0.tif]

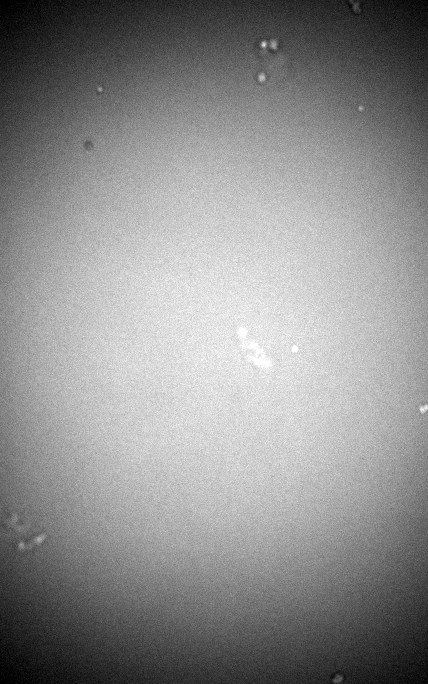

Supplement: Supplementary file 7 — Source data Fig. 5 [file 44318_2026_814_MOESM7_ESM.zip › Figure 5 Raw data/Figure 5 B/SClow ATP/Alonso_SClow_CKii_NSP2sec_sample_25uM_NSP2sec_25uM_SClow_CKii_ATP_sampleAprime_posXY1_channels_t1_posZ0.tif]

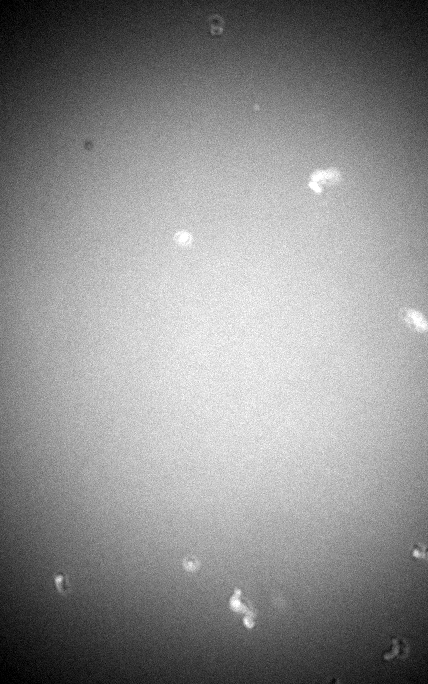

Supplement: Supplementary file 7 — Source data Fig. 5 [file 44318_2026_814_MOESM7_ESM.zip › Figure 5 Raw data/Figure 5 B/SClow ATP/Alonso_CKi_CKii_test_sample_12.5uM_NSP2_12.5uM_SClow__CKii_C_30min_time_posXY6_channels_t1_posZ0.tif]

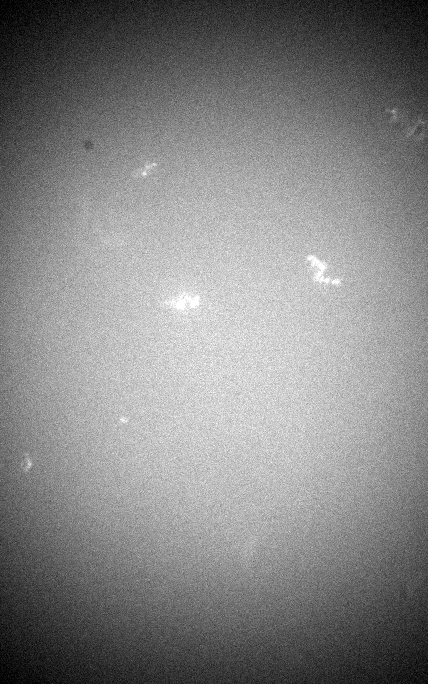

Supplement: Supplementary file 7 — Source data Fig. 5 [file 44318_2026_814_MOESM7_ESM.zip › Figure 5 Raw data/Figure 5 B/SClow ATP/Alonso_SClow_CKii_sample_25uM_NSP2_25uM_SClowi_ATP_sampleC_posXY2_channels_t1_posZ0.tif]

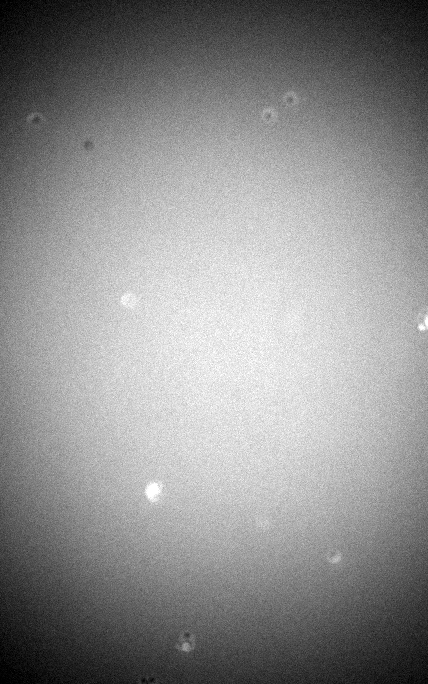

Supplement: Supplementary file 7 — Source data Fig. 5 [file 44318_2026_814_MOESM7_ESM.zip › Figure 5 Raw data/Figure 5 B/SClow ATP/Alonso_CKi_CKii_test_sample_12.5uM_NSP2_12.5uM_SClow__CKii_C_30min_time_posXY4_channels_t1_posZ0.tif]

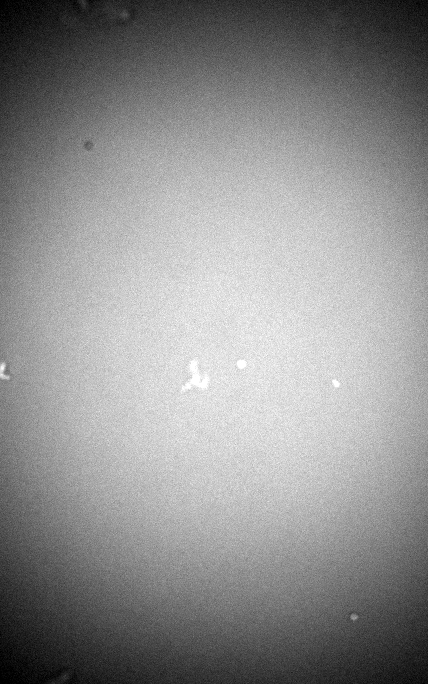

Supplement: Supplementary file 7 — Source data Fig. 5 [file 44318_2026_814_MOESM7_ESM.zip › Figure 5 Raw data/Figure 5 B/SClow ATP/Alonso_SClow_CKii_NSP2sec_sample_25uM_NSP2sec_25uM_SClow_CKii_ATP_sampleAprime_posXY3_channels_t1_posZ0.tif]

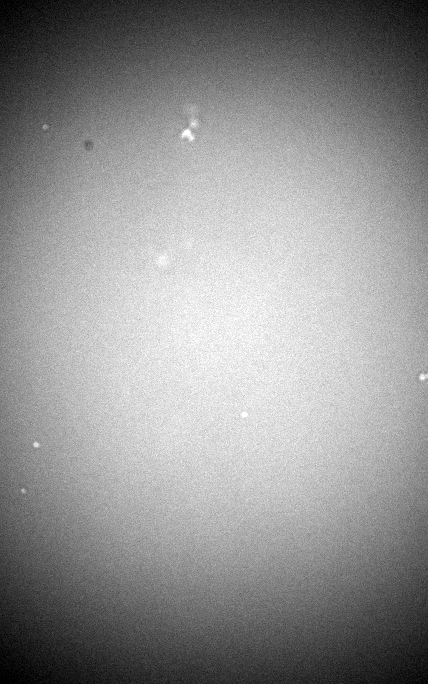

Supplement: Supplementary file 7 — Source data Fig. 5 [file 44318_2026_814_MOESM7_ESM.zip › Figure 5 Raw data/Figure 5 B/SClow ATP/Alonso_SClow_CKii_NSP2sec_sample_25uM_NSP2sec_25uM_SClow_CKii_ATP_sampleAprime_posXY4_channels_t1_posZ0.tif]

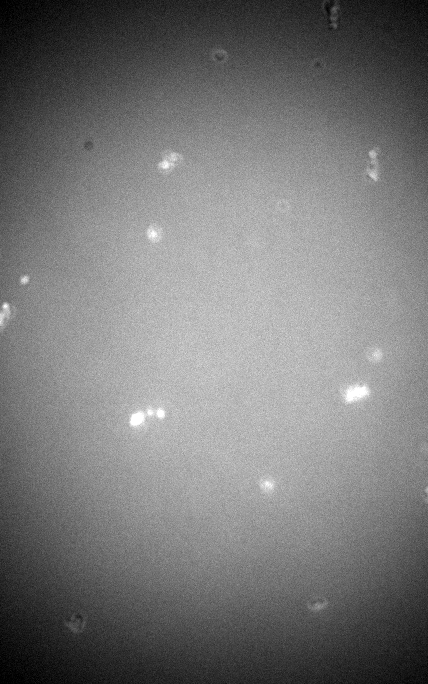

Supplement: Supplementary file 7 — Source data Fig. 5 [file 44318_2026_814_MOESM7_ESM.zip › Figure 5 Raw data/Figure 5 B/SClow ATP/Alonso_CKi_CKii_test_sample_12.5uM_NSP2_12.5uM_SClow__CKii_C_30min_time_posXY3_channels_t1_posZ0.tif]

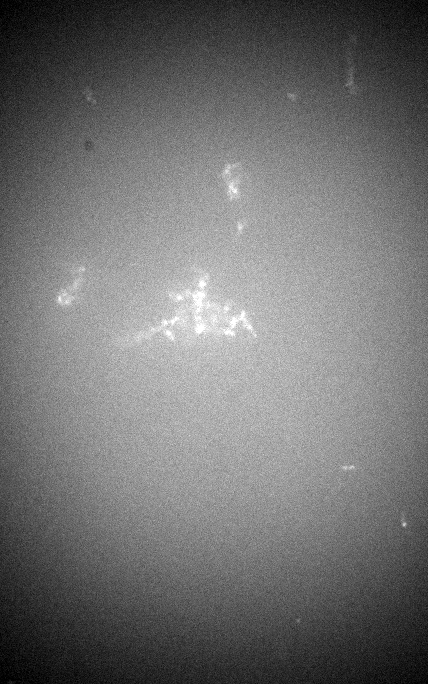

Supplement: Supplementary file 7 — Source data Fig. 5 [file 44318_2026_814_MOESM7_ESM.zip › Figure 5 Raw data/Figure 5 B/SClow ATP/Alonso_SClow_CKii_sample_25uM_NSP2_25uM_SClowi_ATP_sampleC_posXY7_channels_t1_posZ0.tif]

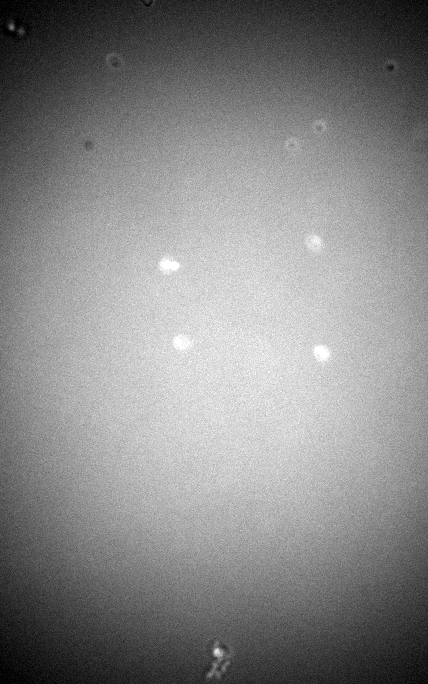

Supplement: Supplementary file 7 — Source data Fig. 5 [file 44318_2026_814_MOESM7_ESM.zip › Figure 5 Raw data/Figure 5 B/SClow ATP/Alonso_CKi_CKii_test_sample_12.5uM_NSP2_12.5uM_SClow__CKii_C_30min_time_posXY1_channels_t1_posZ0.tif]

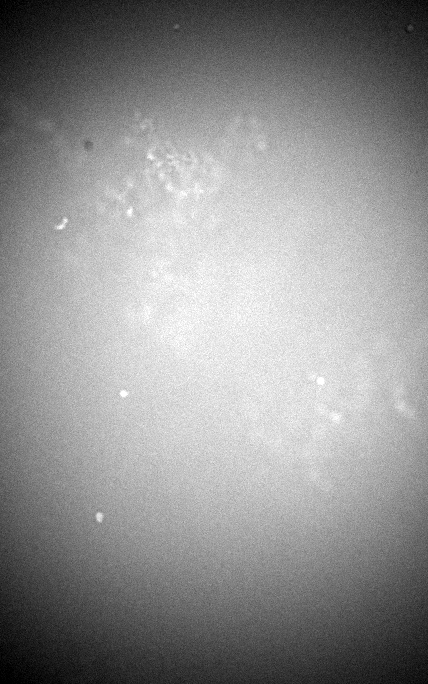

Supplement: Supplementary file 7 — Source data Fig. 5 [file 44318_2026_814_MOESM7_ESM.zip › Figure 5 Raw data/Figure 5 B/SClow ATP/Alonso_SClow_CKii_NSP2sec_sample_25uM_NSP2sec_25uM_SClow_CKii_ATP_sampleAprime_posXY6_channels_t1_posZ0.tif]

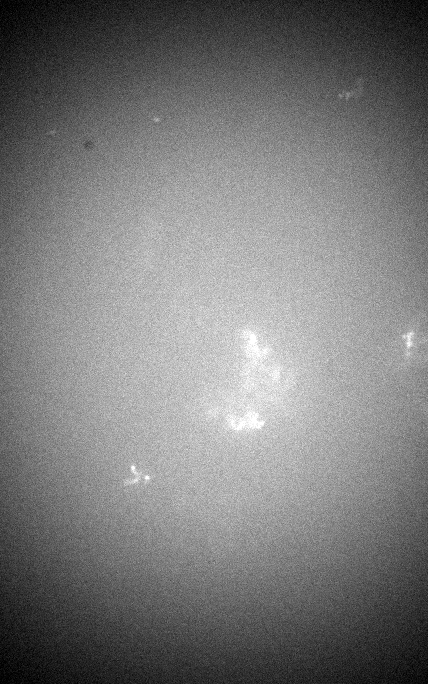

Supplement: Supplementary file 7 — Source data Fig. 5 [file 44318_2026_814_MOESM7_ESM.zip › Figure 5 Raw data/Figure 5 B/SClow ATP/Alonso_SClow_CKii_sample_25uM_NSP2_25uM_SClowi_ATP_sampleC_posXY5_channels_t1_posZ0.tif]

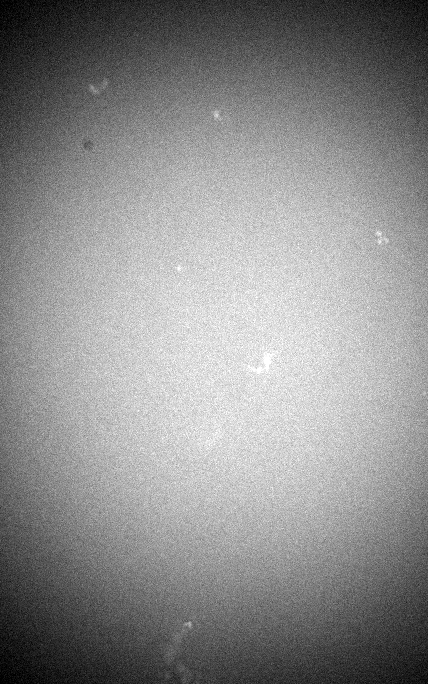

Supplement: Supplementary file 7 — Source data Fig. 5 [file 44318_2026_814_MOESM7_ESM.zip › Figure 5 Raw data/Figure 5 B/SClow ATP/Alonso_SClow_CKii_sample_25uM_NSP2_25uM_SClowi_ATP_sampleC_posXY3_channels_t1_posZ0.tif]

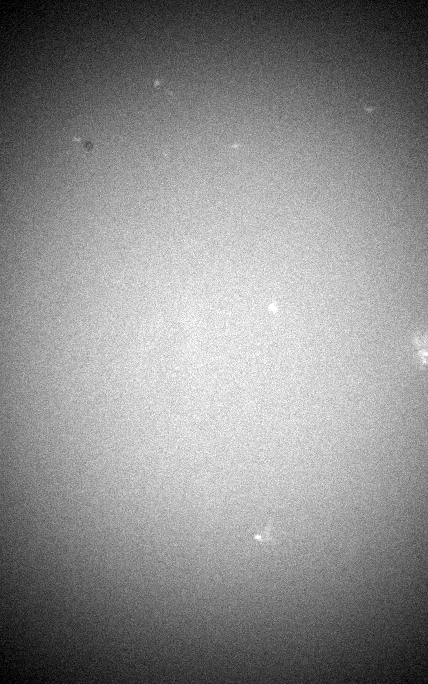

Supplement: Supplementary file 7 — Source data Fig. 5 [file 44318_2026_814_MOESM7_ESM.zip › Figure 5 Raw data/Figure 5 B/SClow ATP/Alonso_SClow_CKii_sample_25uM_NSP2_25uM_SClowi_ATP_sampleC_posXY1_channels_t1_posZ0.tif]

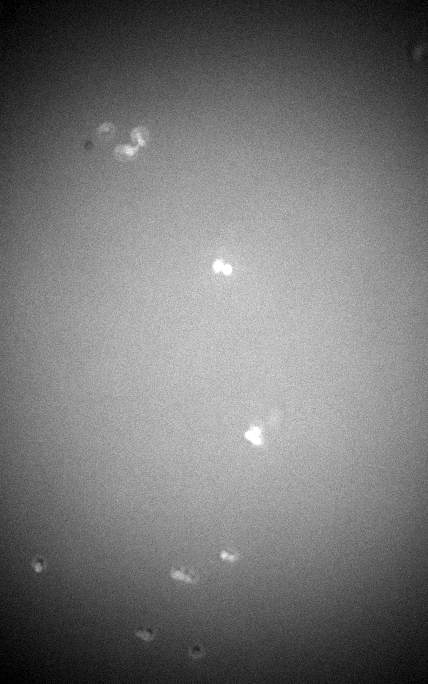

Supplement: Supplementary file 7 — Source data Fig. 5 [file 44318_2026_814_MOESM7_ESM.zip › Figure 5 Raw data/Figure 5 B/SClow ATP/Alonso_CKi_CKii_test_sample_12.5uM_NSP2_12.5uM_SClow__CKii_C_30min_time_posXY5_channels_t1_posZ0.tif]

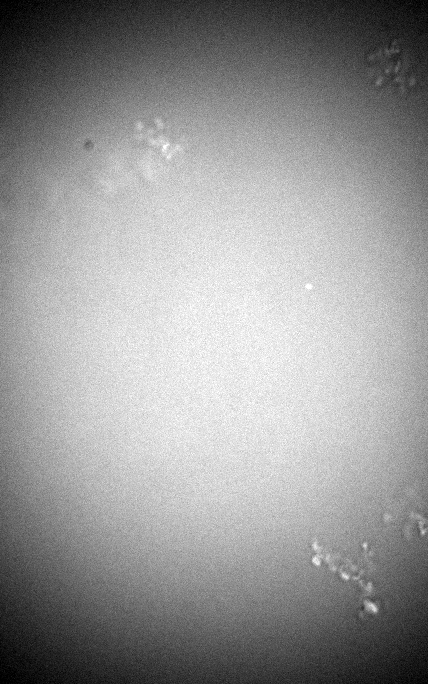

Supplement: Supplementary file 7 — Source data Fig. 5 [file 44318_2026_814_MOESM7_ESM.zip › Figure 5 Raw data/Figure 5 B/SClow ATP/Alonso_SClow_CKii_NSP2sec_sample_25uM_NSP2sec_25uM_SClow_CKii_ATP_sampleAprime_posXY2_channels_t1_posZ0.tif]

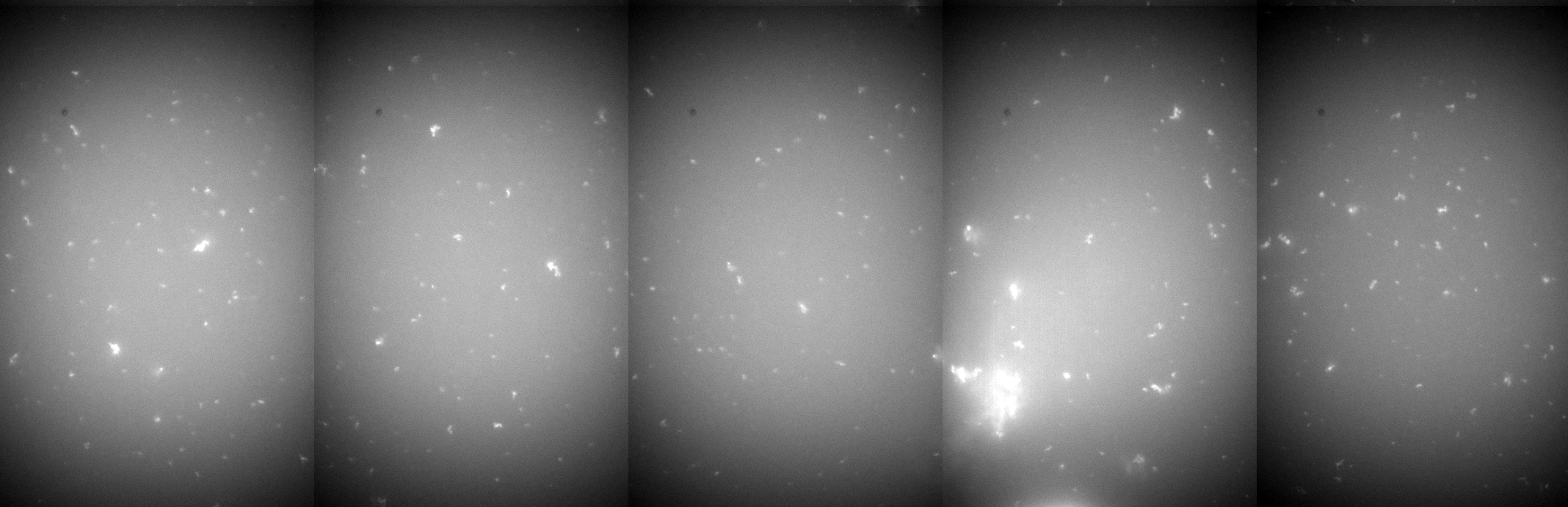

Supplement: Supplementary file 7 — Source data Fig. 5 [file 44318_2026_814_MOESM7_ESM.zip › Figure 5 Raw data/Figure 5 B/SClow scans/2023_08_25_20uM-NSP2-A488_20uM_NSP5-C2S-5min-scan-4.tif]

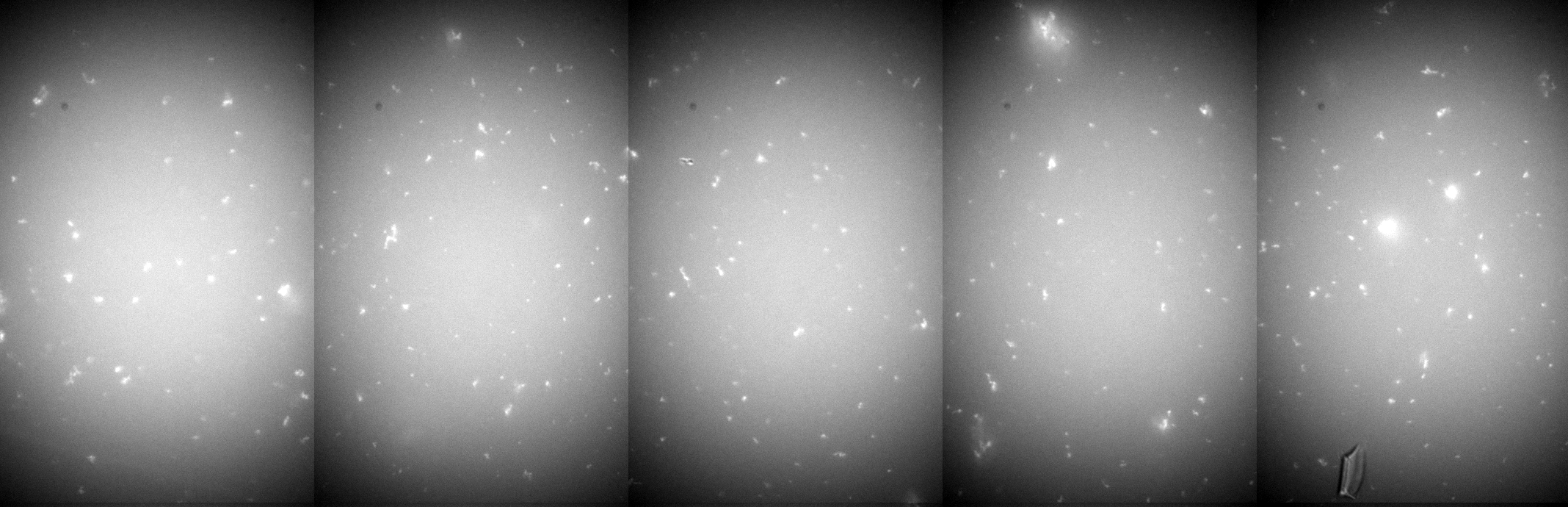

Supplement: Supplementary file 7 — Source data Fig. 5 [file 44318_2026_814_MOESM7_ESM.zip › Figure 5 Raw data/Figure 5 B/SClow scans/2023_08_25_20uM-NSP2-A488_20uM_NSP5-C2S-5min-scan-3.tif]

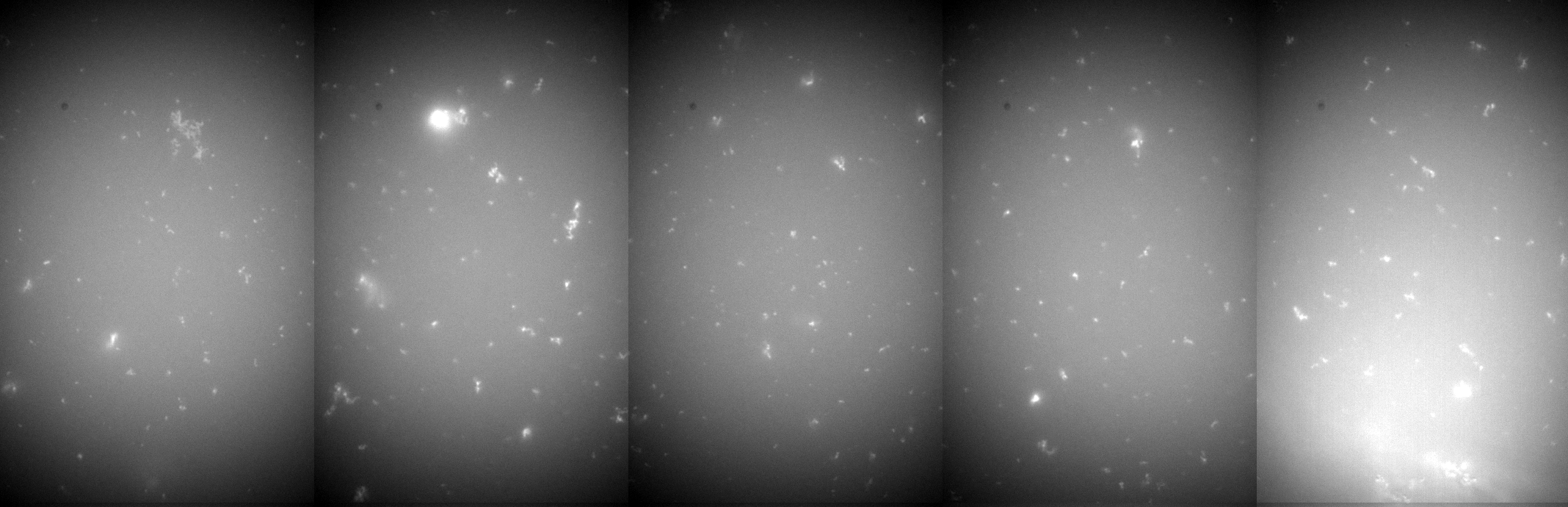

Supplement: Supplementary file 7 — Source data Fig. 5 [file 44318_2026_814_MOESM7_ESM.zip › Figure 5 Raw data/Figure 5 B/SClow scans/2023_08_25_20uM-NSP2-A488_20uM_NSP5-C2S-5min-scan-2.tif]

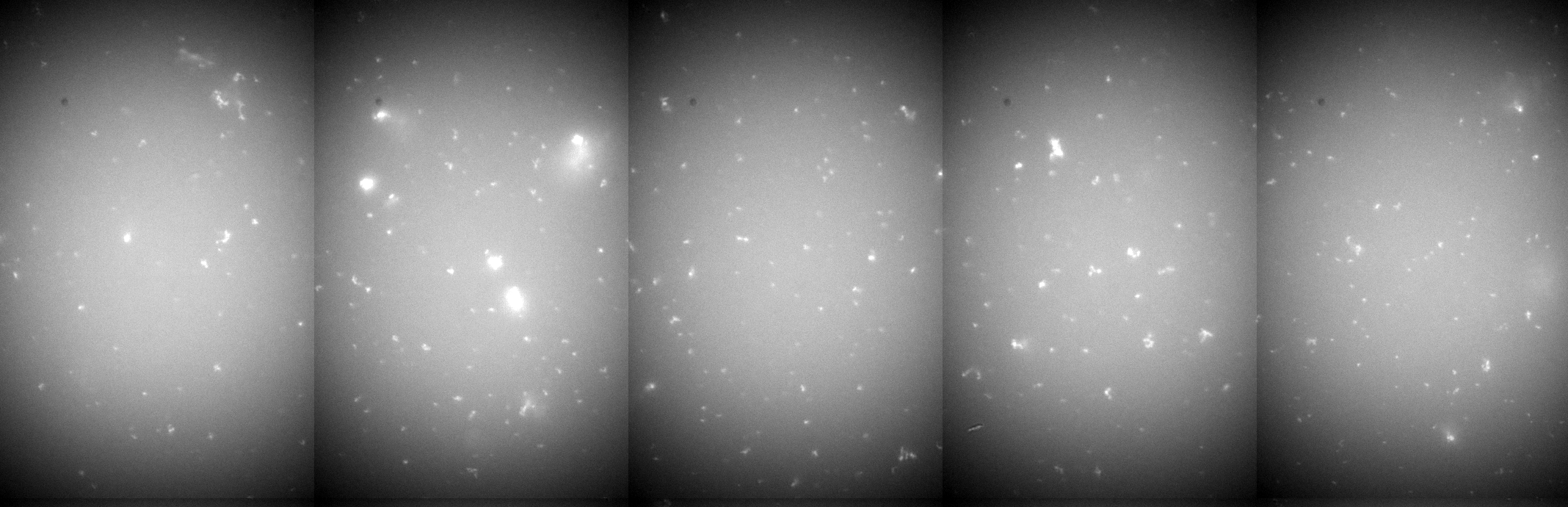

Supplement: Supplementary file 7 — Source data Fig. 5 [file 44318_2026_814_MOESM7_ESM.zip › Figure 5 Raw data/Figure 5 B/SClow scans/2023_08_25_20uM-NSP2-A488_20uM_NSP5-C2S-5min-scan-1.tif]

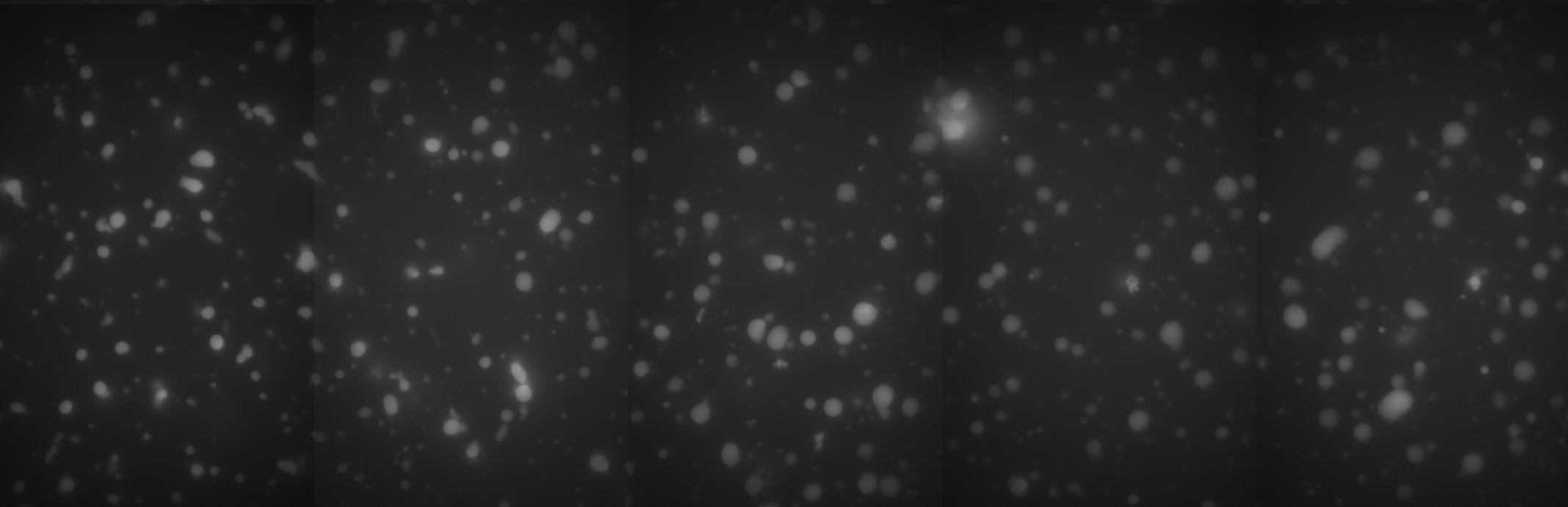

Supplement: Supplementary file 7 — Source data Fig. 5 [file 44318_2026_814_MOESM7_ESM.zip › Figure 5 Raw data/Figure 5 B/SA 11 scan/2023_08_25_20uM-NSP2-A488_20uM_NSP5-SA11-5min-scan-3-2.tif]

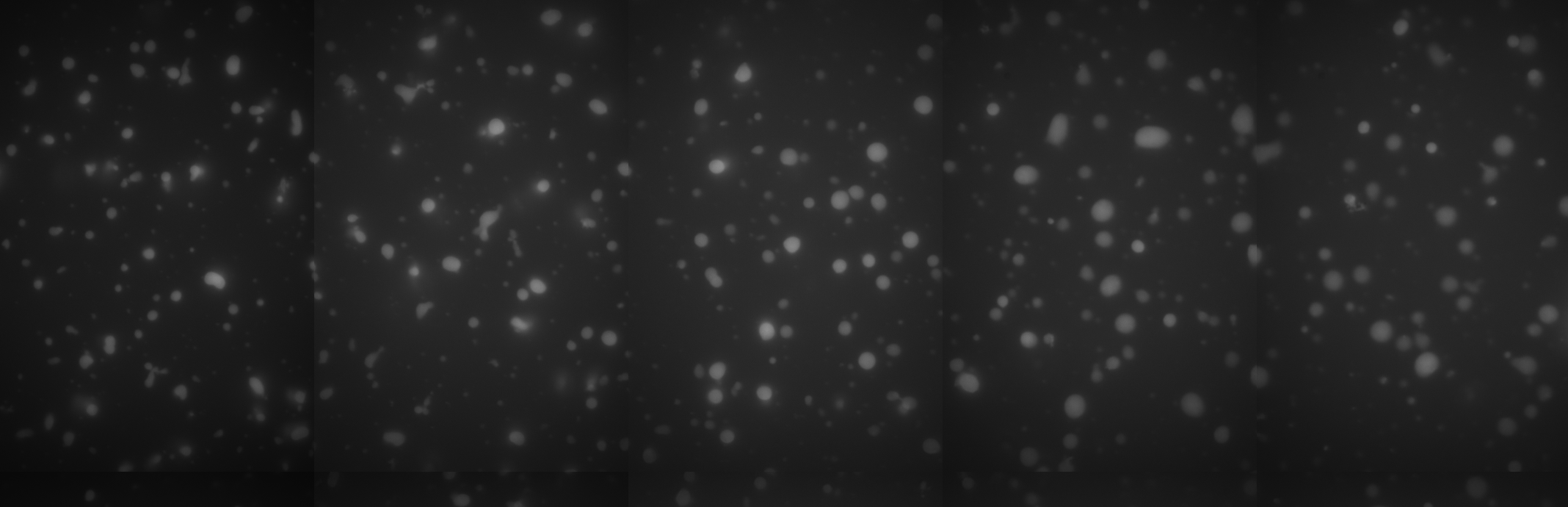

Supplement: Supplementary file 7 — Source data Fig. 5 [file 44318_2026_814_MOESM7_ESM.zip › Figure 5 Raw data/Figure 5 B/SA 11 scan/2023_08_25_20uM-NSP2-A488_20uM_NSP5-SA11-5min-scan-3-3.tif]

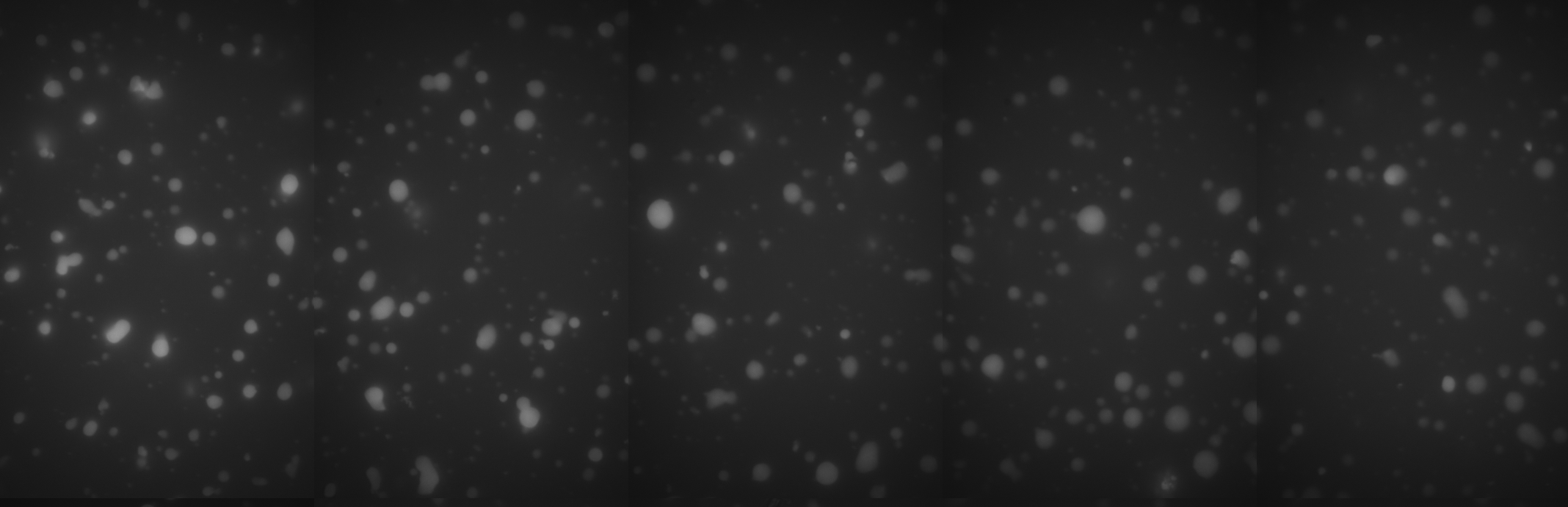

Supplement: Supplementary file 7 — Source data Fig. 5 [file 44318_2026_814_MOESM7_ESM.zip › Figure 5 Raw data/Figure 5 B/SA 11 scan/2023_08_25_20uM-NSP2-A488_20uM_NSP5-SA11-5min-scan-3-1.tif]

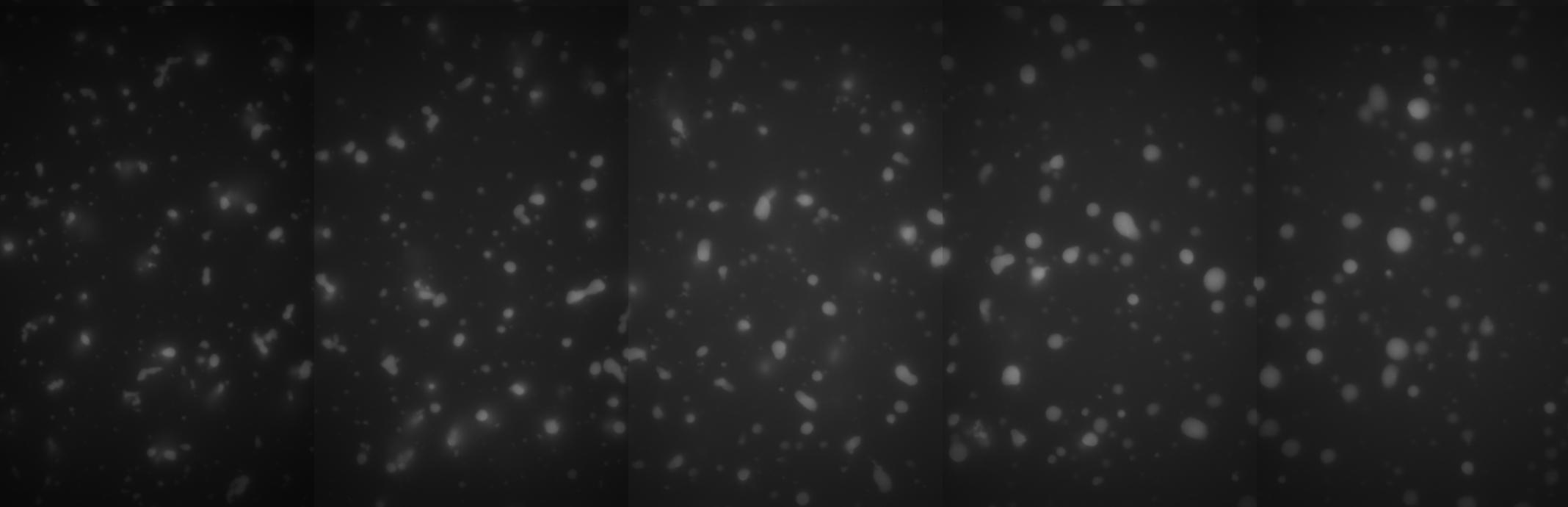

Supplement: Supplementary file 7 — Source data Fig. 5 [file 44318_2026_814_MOESM7_ESM.zip › Figure 5 Raw data/Figure 5 B/SA 11 scan/2023_08_25_20uM-NSP2-A488_20uM_NSP5-SA11-5min-scan-3-4.tif]

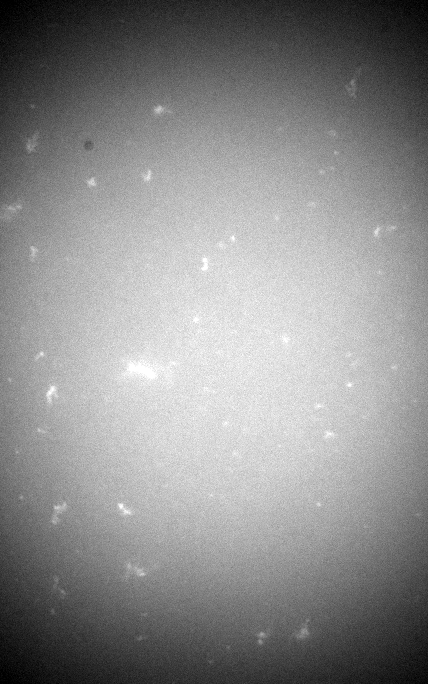

Supplement: Supplementary file 7 — Source data Fig. 5 [file 44318_2026_814_MOESM7_ESM.zip › Figure 5 Raw data/Figure 5 B/S4low/Alonso_BBB_BBBhp_rep_sample_25uM_NSP2_25uM_BBBwt_laserON_posXY2_channels_t1_posZ0.tif]

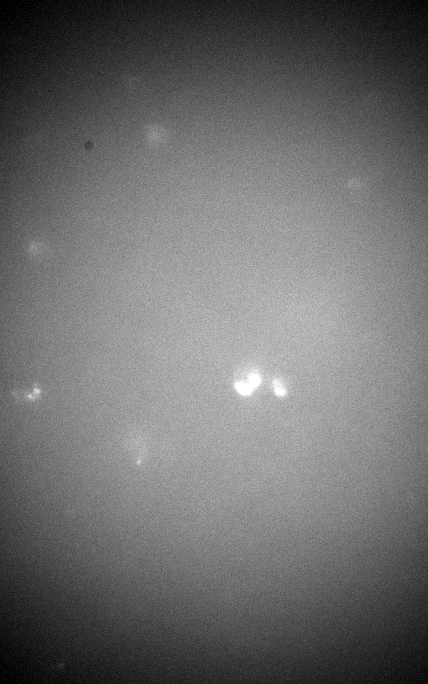

Supplement: Supplementary file 7 — Source data Fig. 5 [file 44318_2026_814_MOESM7_ESM.zip › Figure 5 Raw data/Figure 5 B/S4low/20250821_Alonso_BBB_sample_25uM_NSP2_25uM_BBBwt_posXY1_channels_t1_posZ0.tif]

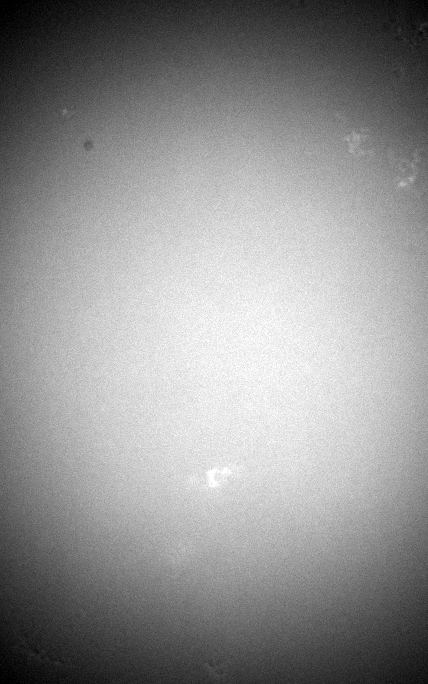

Supplement: Supplementary file 7 — Source data Fig. 5 [file 44318_2026_814_MOESM7_ESM.zip › Figure 5 Raw data/Figure 5 B/S4low/Alonso_BBB_rep_with_NSP2sec_sample_25uM_NSP2sec_25uM_BBB_posXY1_channels_t1_posZ0.tif]

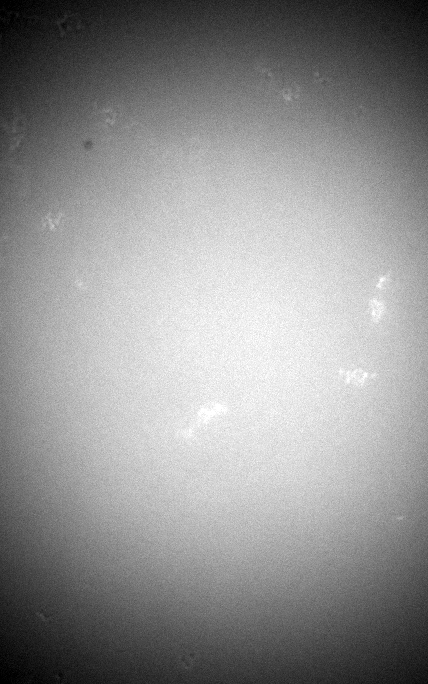

Supplement: Supplementary file 7 — Source data Fig. 5 [file 44318_2026_814_MOESM7_ESM.zip › Figure 5 Raw data/Figure 5 B/S4low/Alonso_BBB_rep_with_NSP2sec_sample_25uM_NSP2sec_25uM_BBB_posXY3_channels_t1_posZ0.tif]

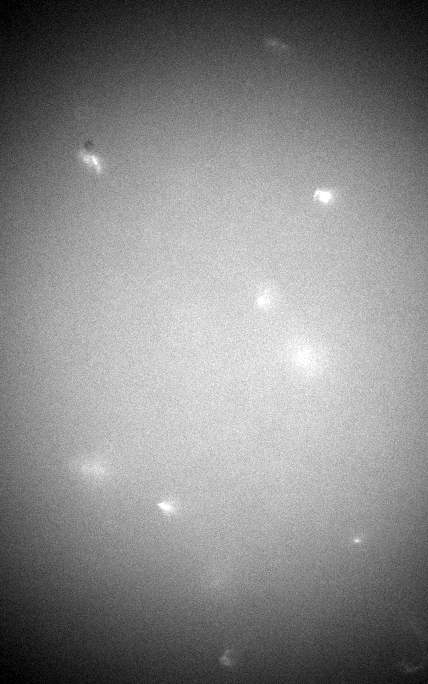

Supplement: Supplementary file 7 — Source data Fig. 5 [file 44318_2026_814_MOESM7_ESM.zip › Figure 5 Raw data/Figure 5 B/S4low/20250821_Alonso_BBB_sample_25uM_NSP2_25uM_BBBwt_posXY3_channels_t1_posZ0.tif]

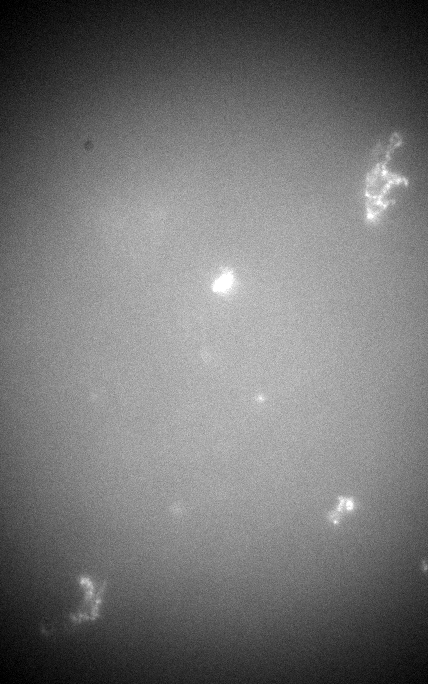

Supplement: Supplementary file 7 — Source data Fig. 5 [file 44318_2026_814_MOESM7_ESM.zip › Figure 5 Raw data/Figure 5 B/S4low/20250821_Alonso_BBB_sample_25uM_NSP2_25uM_BBBwt_posXY5_channels_t1_posZ0.tif]

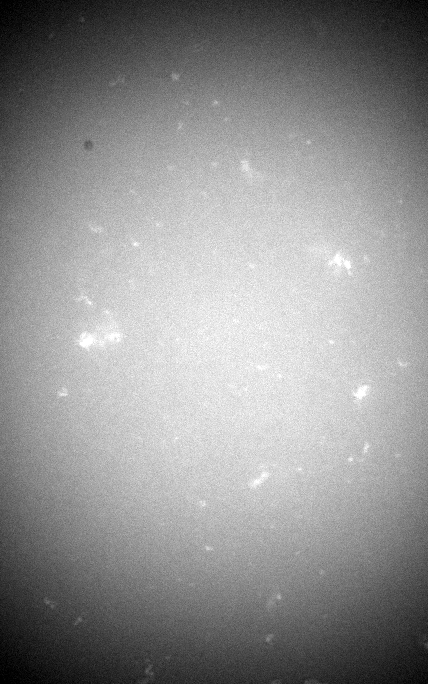

Supplement: Supplementary file 7 — Source data Fig. 5 [file 44318_2026_814_MOESM7_ESM.zip › Figure 5 Raw data/Figure 5 B/S4low/Alonso_BBB_BBBhp_rep_sample_25uM_NSP2_25uM_BBBwt_laserON_posXY6_channels_t1_posZ0.tif]

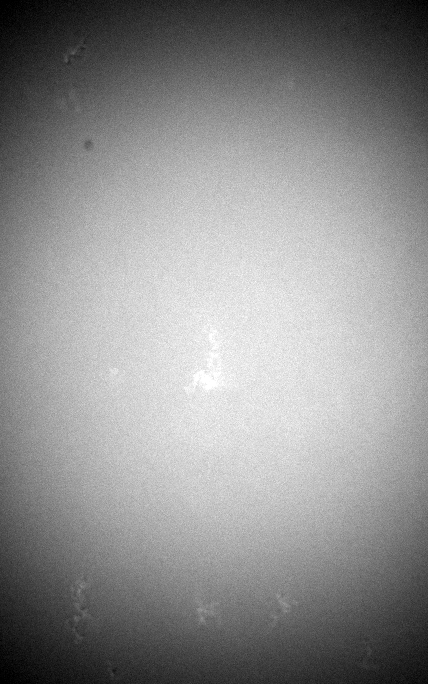

Supplement: Supplementary file 7 — Source data Fig. 5 [file 44318_2026_814_MOESM7_ESM.zip › Figure 5 Raw data/Figure 5 B/S4low/Alonso_BBB_rep_with_NSP2sec_sample_25uM_NSP2sec_25uM_BBB_posXY5_channels_t1_posZ0.tif]

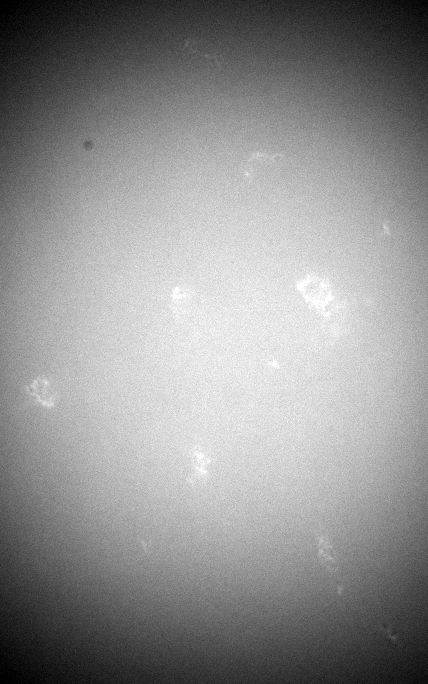

Supplement: Supplementary file 7 — Source data Fig. 5 [file 44318_2026_814_MOESM7_ESM.zip › Figure 5 Raw data/Figure 5 B/S4low/Alonso_BBB_rep_with_NSP2sec_sample_25uM_NSP2sec_25uM_BBB_posXY7_channels_t1_posZ0.tif]

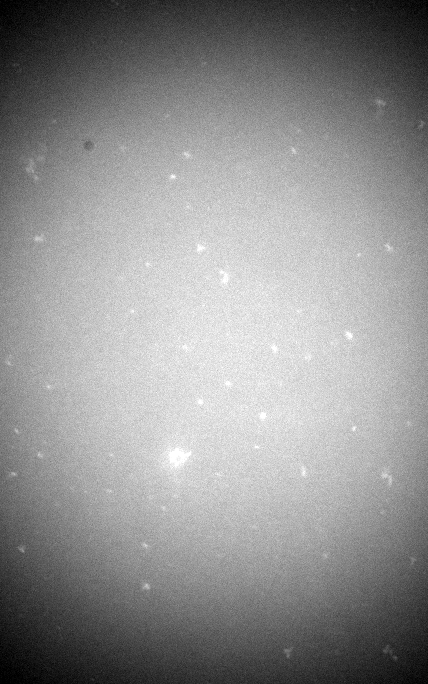

Supplement: Supplementary file 7 — Source data Fig. 5 [file 44318_2026_814_MOESM7_ESM.zip › Figure 5 Raw data/Figure 5 B/S4low/Alonso_BBB_BBBhp_rep_sample_25uM_NSP2_25uM_BBBwt_laserON_posXY4_channels_t1_posZ0.tif]

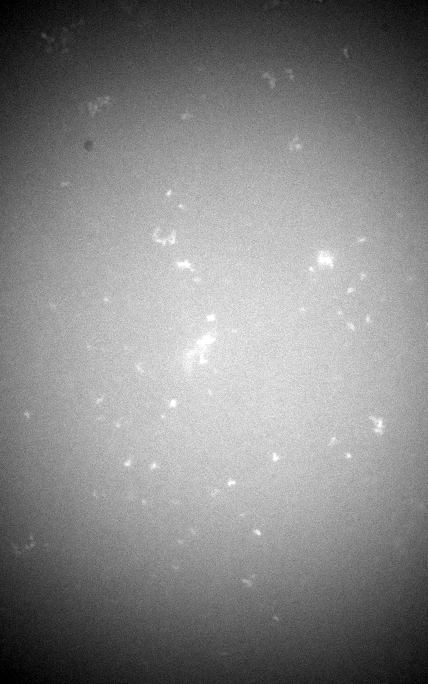

Supplement: Supplementary file 7 — Source data Fig. 5 [file 44318_2026_814_MOESM7_ESM.zip › Figure 5 Raw data/Figure 5 B/S4low/Alonso_BBB_BBBhp_rep_sample_25uM_NSP2_25uM_BBBwt_laserON_posXY3_channels_t1_posZ0.tif]

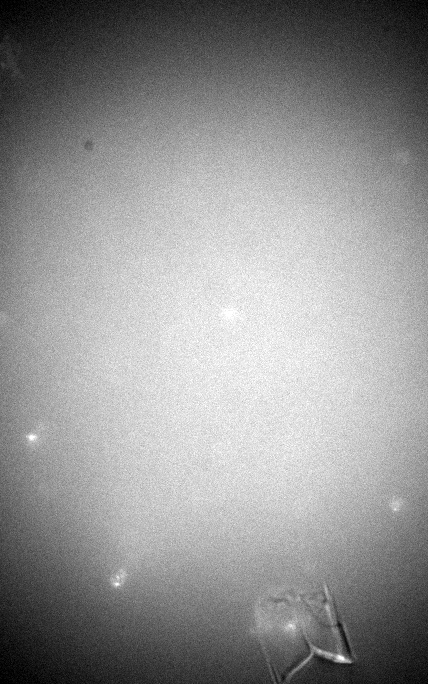

Supplement: Supplementary file 7 — Source data Fig. 5 [file 44318_2026_814_MOESM7_ESM.zip › Figure 5 Raw data/Figure 5 B/S4low/20250821_Alonso_BBB_sample_25uM_NSP2_25uM_BBBwt_posXY2_channels_t1_posZ0.tif]

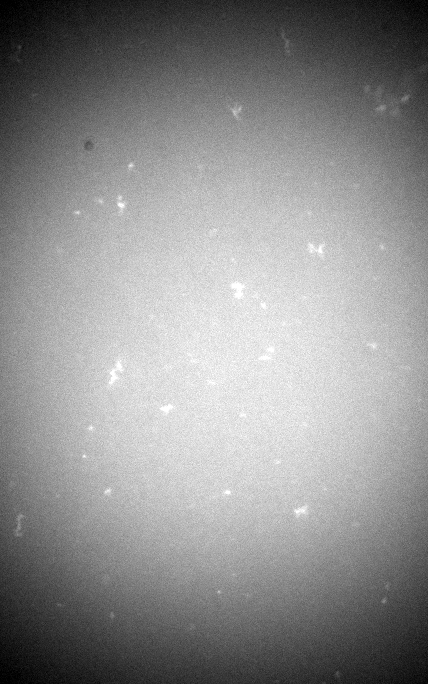

Supplement: Supplementary file 7 — Source data Fig. 5 [file 44318_2026_814_MOESM7_ESM.zip › Figure 5 Raw data/Figure 5 B/S4low/Alonso_BBB_BBBhp_rep_sample_25uM_NSP2_25uM_BBBwt_laserON_posXY1_channels_t1_posZ0.tif]

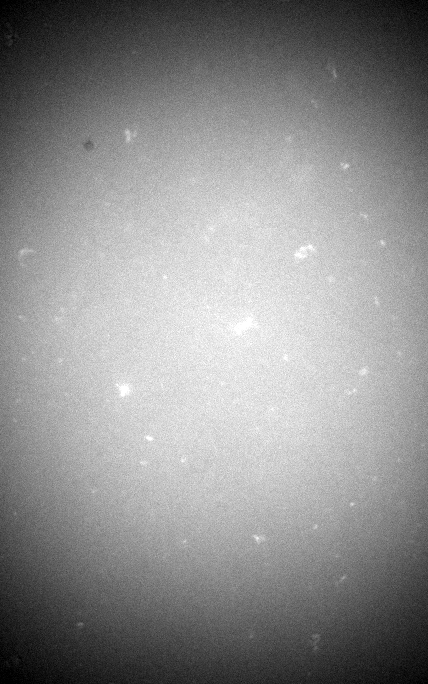

Supplement: Supplementary file 7 — Source data Fig. 5 [file 44318_2026_814_MOESM7_ESM.zip › Figure 5 Raw data/Figure 5 B/S4low/Alonso_BBB_BBBhp_rep_sample_25uM_NSP2_25uM_BBBwt_laserON_posXY7_channels_t1_posZ0.tif]

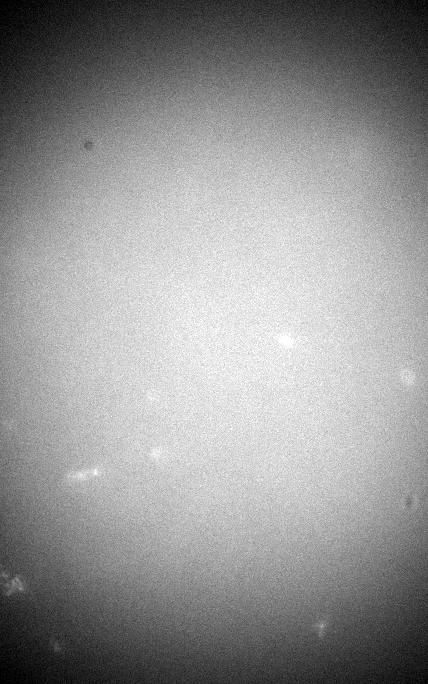

Supplement: Supplementary file 7 — Source data Fig. 5 [file 44318_2026_814_MOESM7_ESM.zip › Figure 5 Raw data/Figure 5 B/S4low/20250821_Alonso_BBB_sample_25uM_NSP2_25uM_BBBwt_posXY4_channels_t1_posZ0.tif]

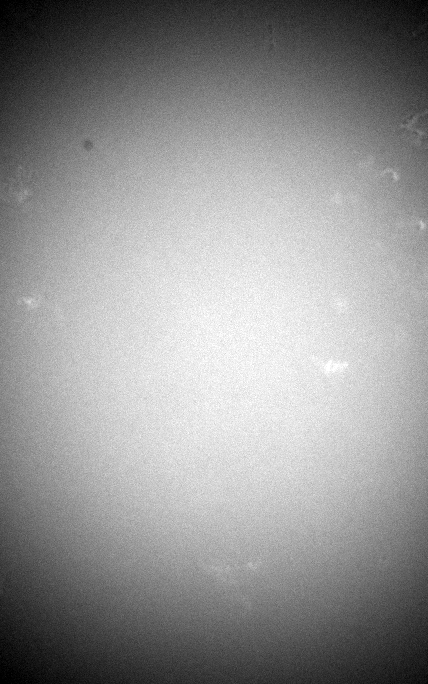

Supplement: Supplementary file 7 — Source data Fig. 5 [file 44318_2026_814_MOESM7_ESM.zip › Figure 5 Raw data/Figure 5 B/S4low/Alonso_BBB_rep_with_NSP2sec_sample_25uM_NSP2sec_25uM_BBB_posXY4_channels_t1_posZ0.tif]

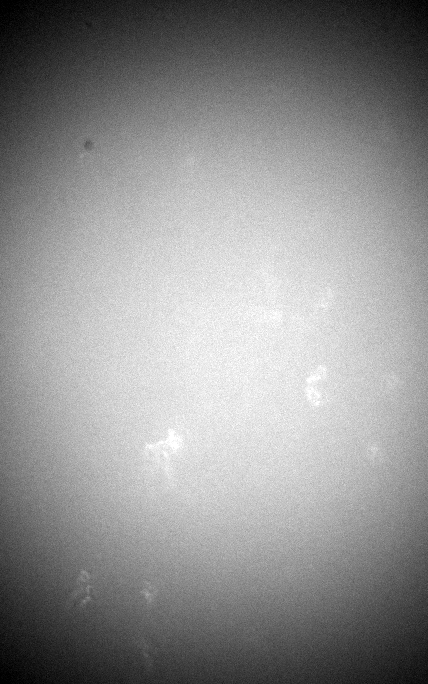

Supplement: Supplementary file 7 — Source data Fig. 5 [file 44318_2026_814_MOESM7_ESM.zip › Figure 5 Raw data/Figure 5 B/S4low/Alonso_BBB_rep_with_NSP2sec_sample_25uM_NSP2sec_25uM_BBB_posXY2_channels_t0_posZ0.tif]

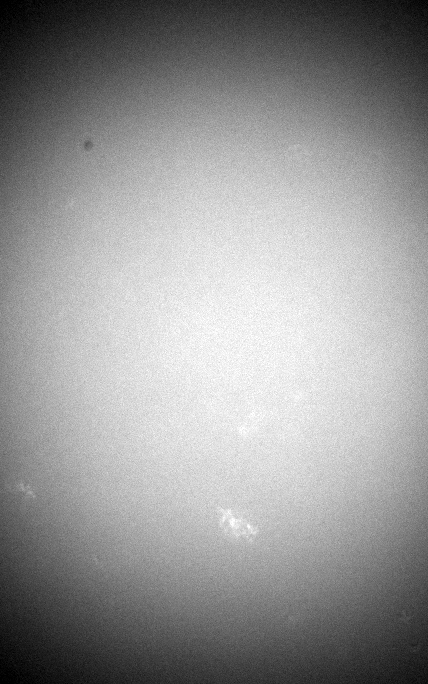

Supplement: Supplementary file 7 — Source data Fig. 5 [file 44318_2026_814_MOESM7_ESM.zip › Figure 5 Raw data/Figure 5 B/S4low/Alonso_BBB_rep_with_NSP2sec_sample_25uM_NSP2sec_25uM_BBB_posXY6_channels_t1_posZ0.tif]

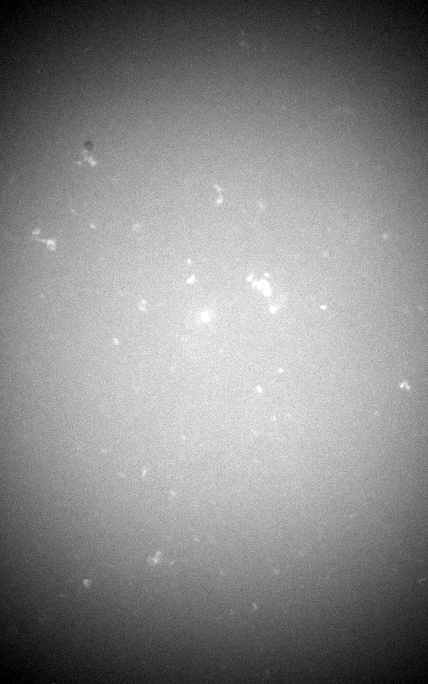

Supplement: Supplementary file 7 — Source data Fig. 5 [file 44318_2026_814_MOESM7_ESM.zip › Figure 5 Raw data/Figure 5 B/S4low/Alonso_BBB_BBBhp_rep_sample_25uM_NSP2_25uM_BBBwt_laserON_posXY5_channels_t1_posZ0.tif]

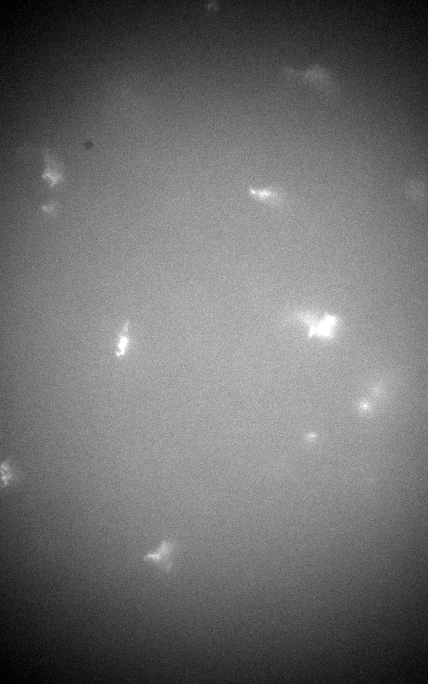

Supplement: Supplementary file 7 — Source data Fig. 5 [file 44318_2026_814_MOESM7_ESM.zip › Figure 5 Raw data/Figure 5 B/S4low/20250821_Alonso_BBB_sample_25uM_NSP2_25uM_BBBwt_posXY6_channels_t1_posZ0.tif]

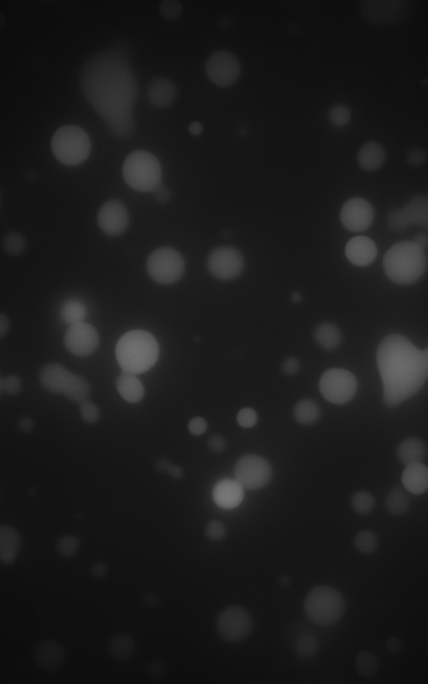

Supplement: Supplementary file 7 — Source data Fig. 5 [file 44318_2026_814_MOESM7_ESM.zip › Figure 5 Raw data/Figure 5 B/S4low HP/Alonso_BBB_BBBhp_rep_sample_25uM_NSP2_25uM_BBBhp_posXY6_channels_t1_posZ0.tif]

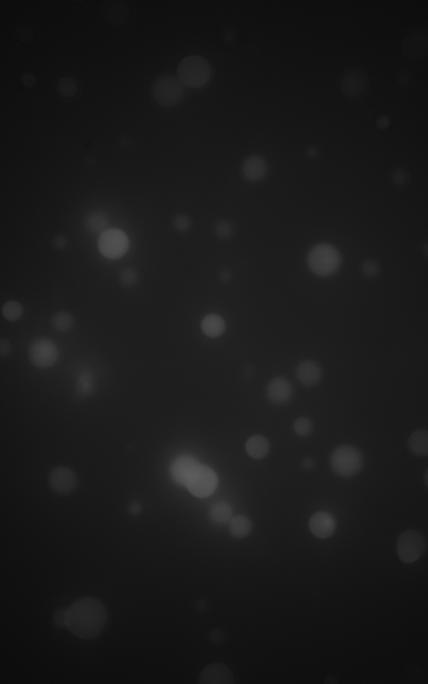

Supplement: Supplementary file 7 — Source data Fig. 5 [file 44318_2026_814_MOESM7_ESM.zip › Figure 5 Raw data/Figure 5 B/S4low HP/Alonso_BBB_rep_with_NSP2sec_sample_25uM_NSP2sec_25uM_BBBhp_posXY3_channels_t1_posZ0.tif]

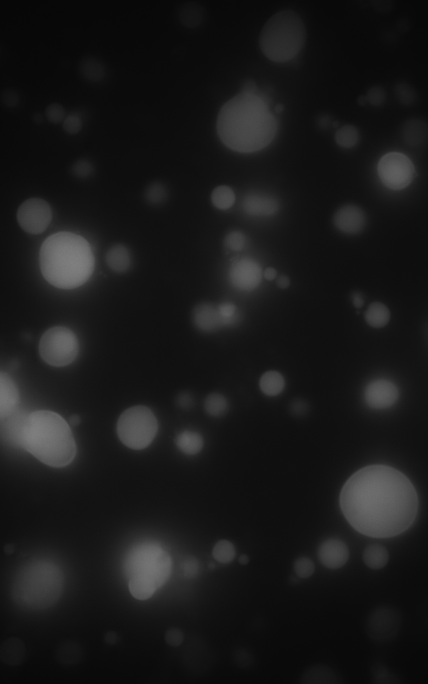

Supplement: Supplementary file 7 — Source data Fig. 5 [file 44318_2026_814_MOESM7_ESM.zip › Figure 5 Raw data/Figure 5 B/S4low HP/20250821_Alonso_BBB_sample_25uM_NSP2_25uM_BBBhp_posXY7_channels_t1_posZ0.tif]

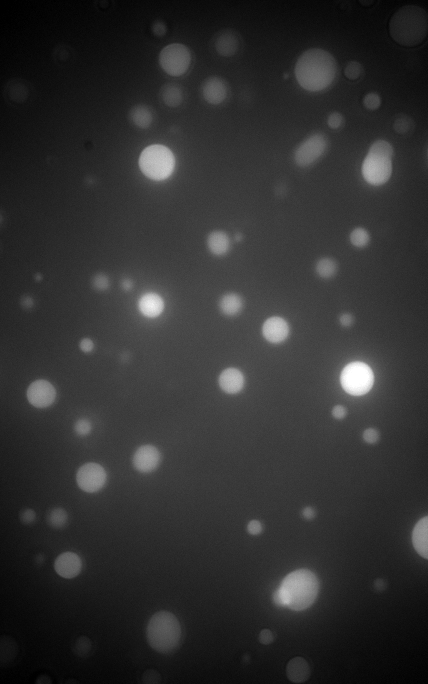

Supplement: Supplementary file 7 — Source data Fig. 5 [file 44318_2026_814_MOESM7_ESM.zip › Figure 5 Raw data/Figure 5 B/S4low HP/Alonso_BBB_rep_with_NSP2sec_sample_25uM_NSP2sec_25uM_BBBhp_posXY1_channels_t1_posZ0.tif]

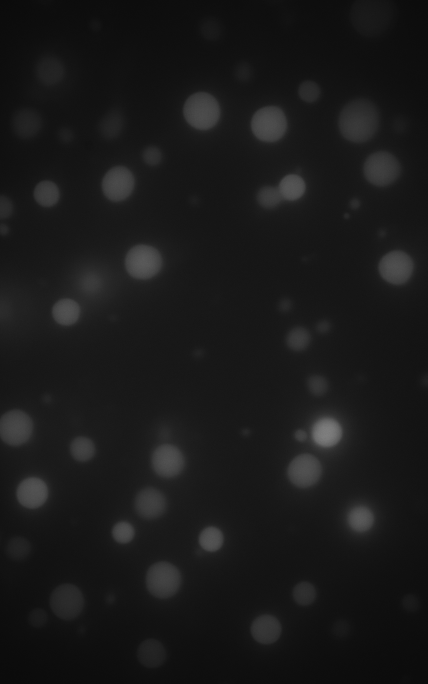

Supplement: Supplementary file 7 — Source data Fig. 5 [file 44318_2026_814_MOESM7_ESM.zip › Figure 5 Raw data/Figure 5 B/S4low HP/Alonso_BBB_BBBhp_rep_sample_25uM_NSP2_25uM_BBBhp_posXY4_channels_t1_posZ0.tif]
