# Supplementary material for: Phosphorylation tunes strain-specific protein condensation during rotavirus replication organelle assembly
Source: EMBO J. 2026 May 26;45(13):4733–65. doi: 10.1038/s44318-026-00814-z (PMC13324165; doi:10.1038/s44318-026-00814-z)
Supplement: Supplementary file 5 — Source data Fig. 3 [file 44318_2026_814_MOESM5_ESM.zip › Figure 3/Figure 3A/25.11.25 replication kinetic plaque assay protocol.docx]

Viral replication

- MA104 WT cells were plated in 6 well plates in DMEM (10% FBS, P/S, NEAA and GlutaMax) and grown overnight at 37 ^o^C
- Cells were washed once with 2 mL PBS and twice with serum free DMEM (P/S, NEAA and GlutaMax) (SFM)
- Virus (stock previously titred by plaque assay in triplicate) was added to cells in final MOI of 0.03 (assuming a final cell count of 1.2 x 10^6) in 800 µL of SFM
- Following 1 hour of adsorption (tilting every 15 mins), media was removed and 2 mL of SFM added supplemented with trypsin (final concentration 0.6 µg/mL)
- Incubated for stated time (0h,8h,16h,24h,48h or 72h)
- Cells were then scraped and mixture removed
- Mixture was freeze/thawed three times before being spun at 7k x g for 10 minutes before 1.9 mL of clarified lysate taken into fresh 2 mL eppendorf
- Trypsin was added to activate virus (2 µg/mL final) and incubated at 37 ^o^C for 30 mins

Plaque assay

- MA104 -NSP5 cells were plated in 12 well plates in DMEM and grown overnight at 37 ^o^C
- Plates for plaque assay were washed once with 1 mL PBS and twice with 1 mL SFM and incubated at 37 ^o^C whilst dilutions were prepared.
- To make the serial dilutions 450 µl SFM added to wells of a dilution plate (I used 24-well plates for this) and 50µl of activated virus stock added to the first well (this is 10^-1^ dilution). The diluted virus was pipetted up and down 20-30x to mix well, and tip was discarded. This was repeated down the dilution series using a fresh tip between each dilution and mixing the virus each time.
- SFM was removed from 12 well plates was removed and 400 µL of serially diluted virus was added to each well
- Following 1 hour of adsorption (tilting every 15 mins), media was removed and 1 mL of overlay was added per well (Overlay: 50 % SFM, 1.2 % Avicell, 0.5 µg/mL trypsin)
- Plaque assays were incubated for 4 days at 37 ^o^C
- Overlay was removed and wells were washed twice with PBS to remove remaining overlay
- Staining was conducted for at least 15 minutes with 0.5 mL crystal violet (0.2% CV, 20% methanol)
