# Supplementary figures and images for: Phosphorylation tunes strain-specific protein condensation during rotavirus replication organelle assembly (part 2 of 3)
Source: EMBO J. 2026 May 26;45(13):4733–65. doi: 10.1038/s44318-026-00814-z (PMC13324165; doi:10.1038/s44318-026-00814-z)

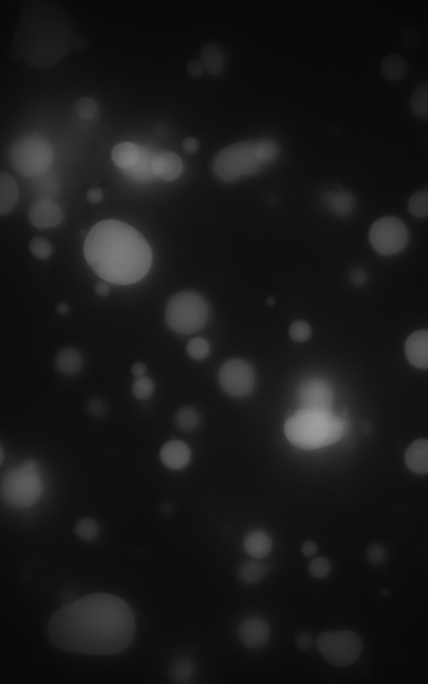

Supplement: Supplementary file 7 — Source data Fig. 5 [file 44318_2026_814_MOESM7_ESM.zip › Figure 5 Raw data/Figure 5 B/S4low HP/20250821_Alonso_BBB_sample_25uM_NSP2_25uM_BBBhp_posXY5_channels_t1_posZ0.tif]

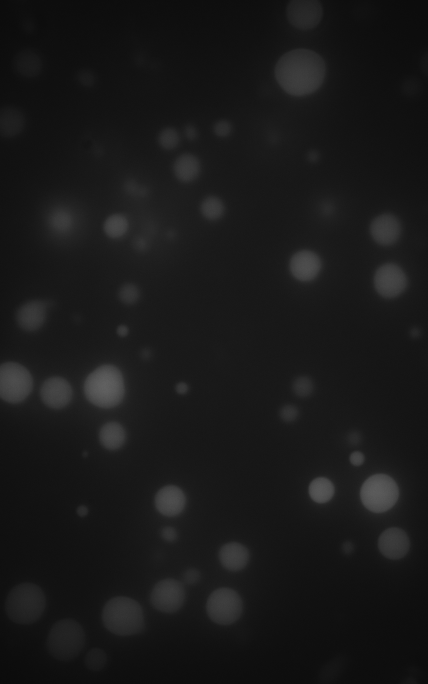

Supplement: Supplementary file 7 — Source data Fig. 5 [file 44318_2026_814_MOESM7_ESM.zip › Figure 5 Raw data/Figure 5 B/S4low HP/Alonso_BBB_BBBhp_rep_sample_25uM_NSP2_25uM_BBBhp_posXY2_channels_t1_posZ0.tif]

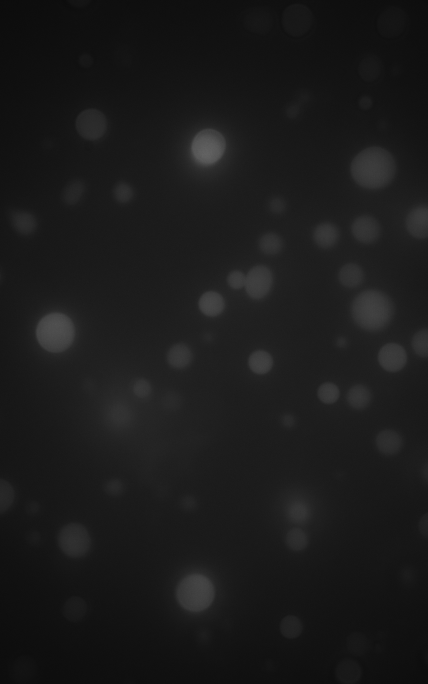

Supplement: Supplementary file 7 — Source data Fig. 5 [file 44318_2026_814_MOESM7_ESM.zip › Figure 5 Raw data/Figure 5 B/S4low HP/Alonso_BBB_rep_with_NSP2sec_sample_25uM_NSP2sec_25uM_BBBhp_posXY7_channels_t1_posZ0.tif]

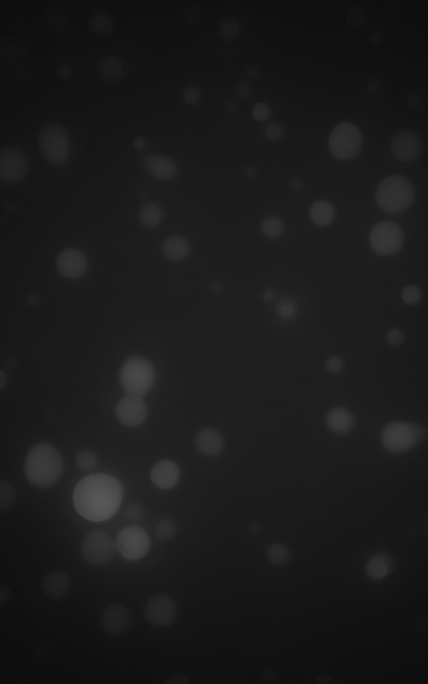

Supplement: Supplementary file 7 — Source data Fig. 5 [file 44318_2026_814_MOESM7_ESM.zip › Figure 5 Raw data/Figure 5 B/S4low HP/Alonso_BBB_rep_with_NSP2sec_sample_25uM_NSP2sec_25uM_BBBhp_posXY5_channels_t1_posZ0.tif]

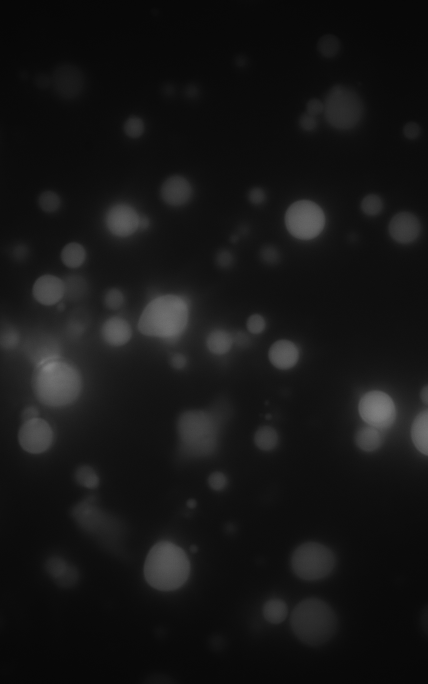

Supplement: Supplementary file 7 — Source data Fig. 5 [file 44318_2026_814_MOESM7_ESM.zip › Figure 5 Raw data/Figure 5 B/S4low HP/20250821_Alonso_BBB_sample_25uM_NSP2_25uM_BBBhp_posXY6_channels_t1_posZ0.tif]

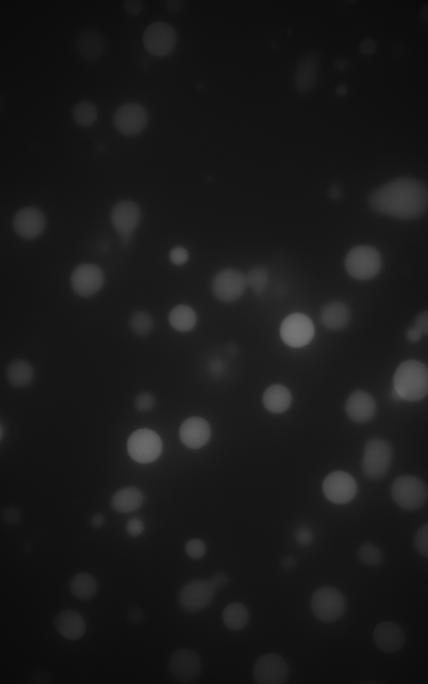

Supplement: Supplementary file 7 — Source data Fig. 5 [file 44318_2026_814_MOESM7_ESM.zip › Figure 5 Raw data/Figure 5 B/S4low HP/Alonso_BBB_BBBhp_rep_sample_25uM_NSP2_25uM_BBBhp_posXY7_channels_t1_posZ0.tif]

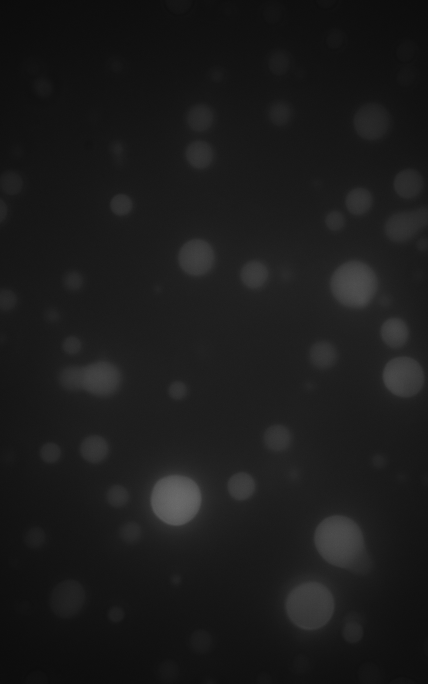

Supplement: Supplementary file 7 — Source data Fig. 5 [file 44318_2026_814_MOESM7_ESM.zip › Figure 5 Raw data/Figure 5 B/S4low HP/Alonso_BBB_rep_with_NSP2sec_sample_25uM_NSP2sec_25uM_BBBhp_posXY2_channels_t1_posZ0.tif]

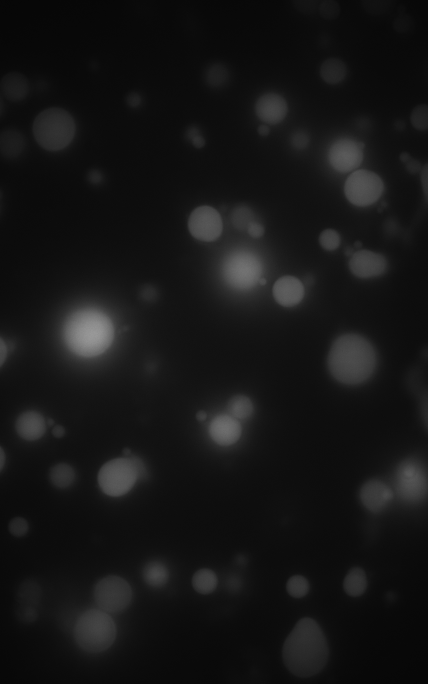

Supplement: Supplementary file 7 — Source data Fig. 5 [file 44318_2026_814_MOESM7_ESM.zip › Figure 5 Raw data/Figure 5 B/S4low HP/20250821_Alonso_BBB_sample_25uM_NSP2_25uM_BBBhp_posXY4_channels_t1_posZ0.tif]

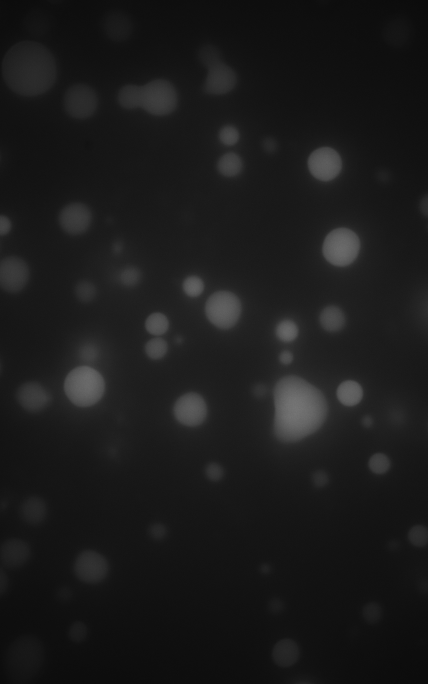

Supplement: Supplementary file 7 — Source data Fig. 5 [file 44318_2026_814_MOESM7_ESM.zip › Figure 5 Raw data/Figure 5 B/S4low HP/Alonso_BBB_BBBhp_rep_sample_25uM_NSP2_25uM_BBBhp_posXY5_channels_t1_posZ0.tif]

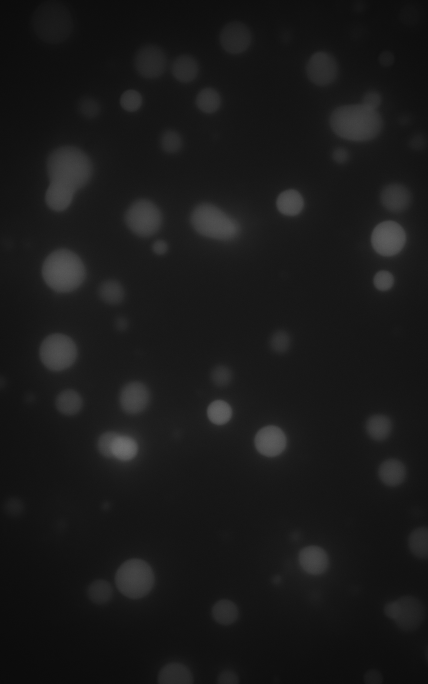

Supplement: Supplementary file 7 — Source data Fig. 5 [file 44318_2026_814_MOESM7_ESM.zip › Figure 5 Raw data/Figure 5 B/S4low HP/Alonso_BBB_BBBhp_rep_sample_25uM_NSP2_25uM_BBBhp_posXY3_channels_t1_posZ0.tif]

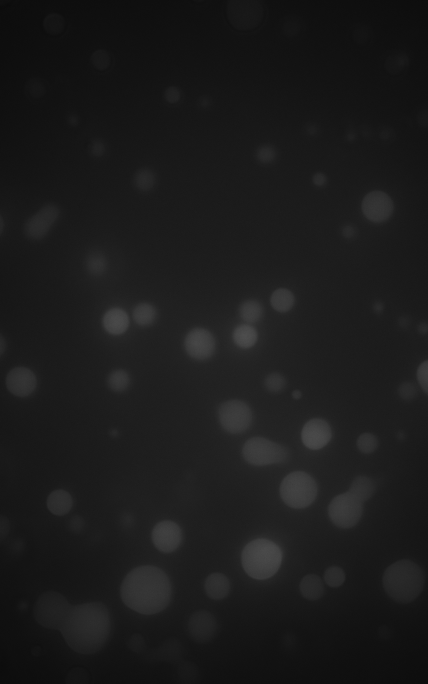

Supplement: Supplementary file 7 — Source data Fig. 5 [file 44318_2026_814_MOESM7_ESM.zip › Figure 5 Raw data/Figure 5 B/S4low HP/Alonso_BBB_rep_with_NSP2sec_sample_25uM_NSP2sec_25uM_BBBhp_posXY6_channels_t1_posZ0.tif]

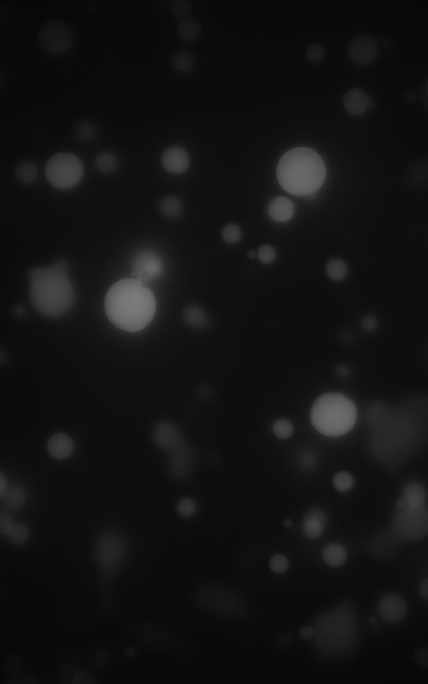

Supplement: Supplementary file 7 — Source data Fig. 5 [file 44318_2026_814_MOESM7_ESM.zip › Figure 5 Raw data/Figure 5 B/S4low HP/20250821_Alonso_BBB_sample_25uM_NSP2_25uM_BBBhp_posXY2_channels_t1_posZ0.tif]

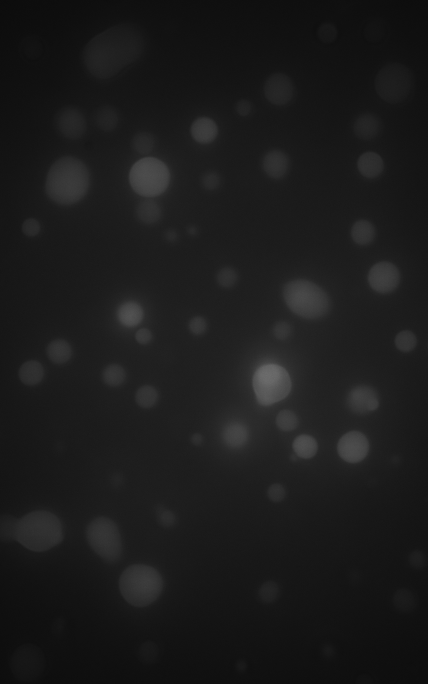

Supplement: Supplementary file 7 — Source data Fig. 5 [file 44318_2026_814_MOESM7_ESM.zip › Figure 5 Raw data/Figure 5 B/S4low HP/Alonso_BBB_rep_with_NSP2sec_sample_25uM_NSP2sec_25uM_BBBhp_posXY4_channels_t1_posZ0.tif]

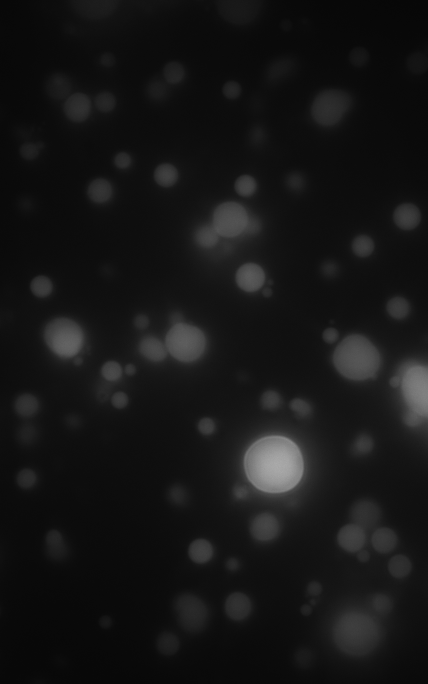

Supplement: Supplementary file 7 — Source data Fig. 5 [file 44318_2026_814_MOESM7_ESM.zip › Figure 5 Raw data/Figure 5 B/S4low HP/20250821_Alonso_BBB_sample_25uM_NSP2_25uM_BBBhp_posXY8_channels_t1_posZ0.tif]

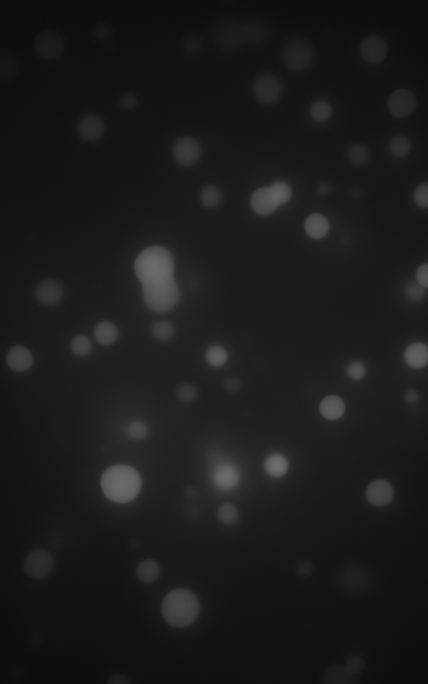

Supplement: Supplementary file 7 — Source data Fig. 5 [file 44318_2026_814_MOESM7_ESM.zip › Figure 5 Raw data/Figure 5 B/S4low HP/Alonso_BBB_BBBhp_rep_sample_25uM_NSP2_25uM_BBBhp_posXY1_channels_t1_posZ0.tif]

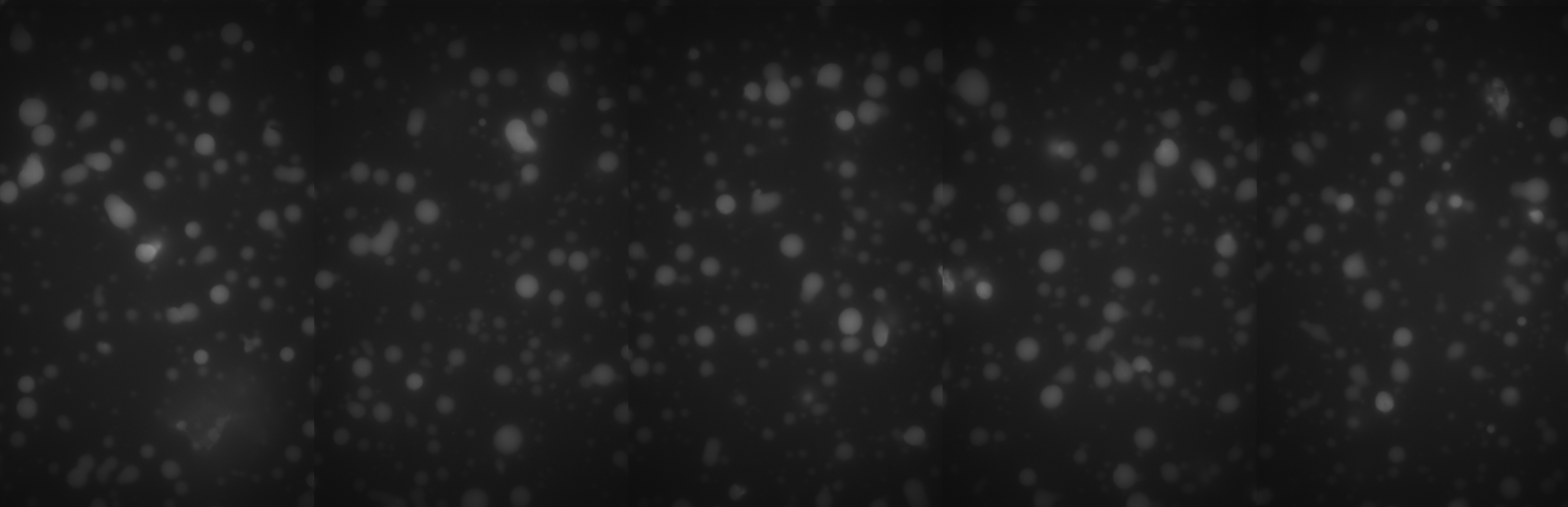

Supplement: Supplementary file 7 — Source data Fig. 5 [file 44318_2026_814_MOESM7_ESM.zip › Figure 5 Raw data/Figure 5 B/SClow HP scans/2023_08_25_20uM-NSP2-A488_20uM_NSP5-C2S-HP-5min-scan-4.tif]

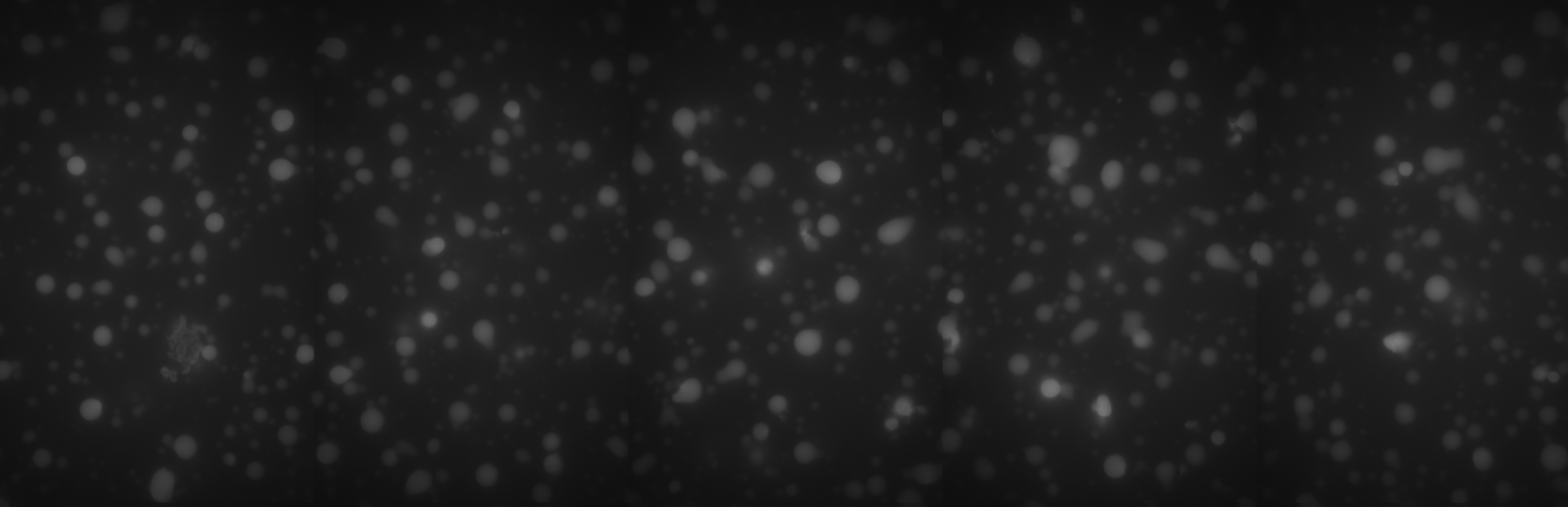

Supplement: Supplementary file 7 — Source data Fig. 5 [file 44318_2026_814_MOESM7_ESM.zip › Figure 5 Raw data/Figure 5 B/SClow HP scans/2023_08_25_20uM-NSP2-A488_20uM_NSP5-C2S-HP-5min-scan-1.tif]

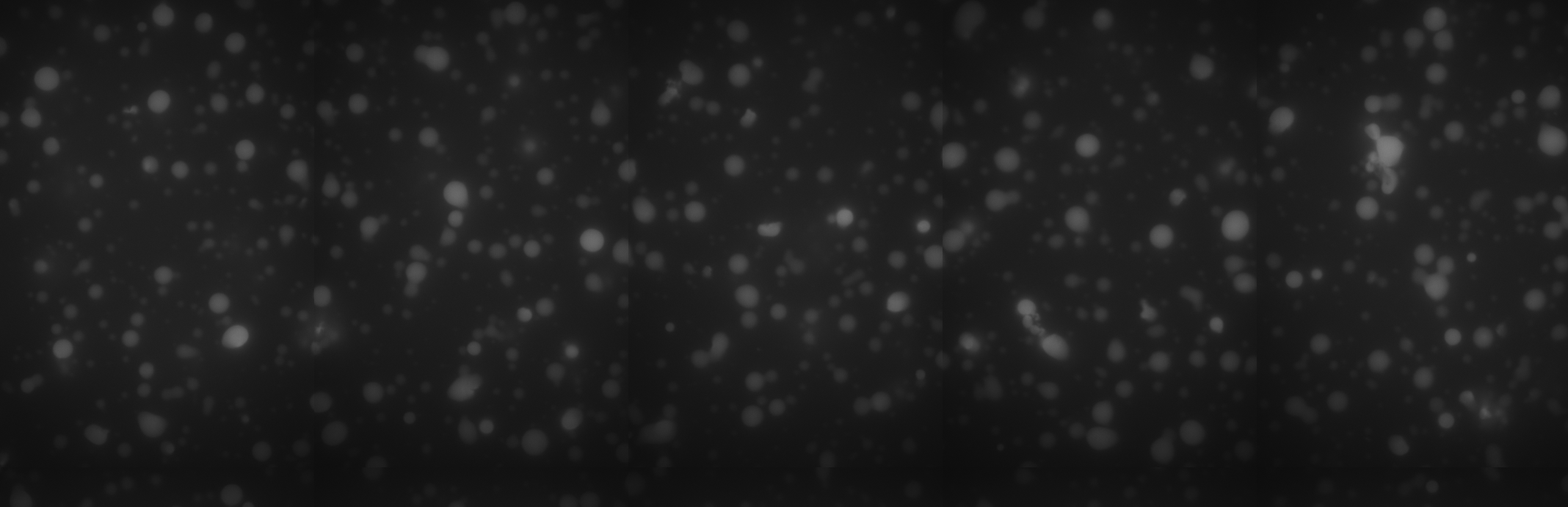

Supplement: Supplementary file 7 — Source data Fig. 5 [file 44318_2026_814_MOESM7_ESM.zip › Figure 5 Raw data/Figure 5 B/SClow HP scans/2023_08_25_20uM-NSP2-A488_20uM_NSP5-C2S-HP-5min-scan-3.tif]

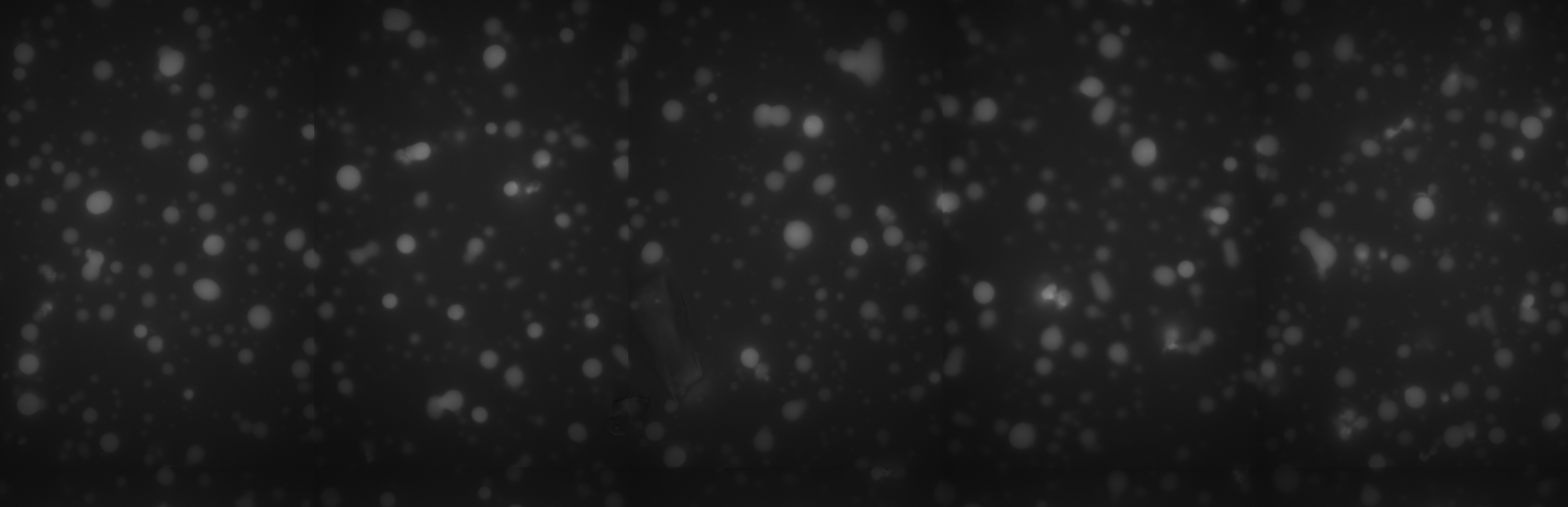

Supplement: Supplementary file 7 — Source data Fig. 5 [file 44318_2026_814_MOESM7_ESM.zip › Figure 5 Raw data/Figure 5 B/SClow HP scans/2023_08_25_20uM-NSP2-A488_20uM_NSP5-C2S-HP-5min-scan-2.tif]

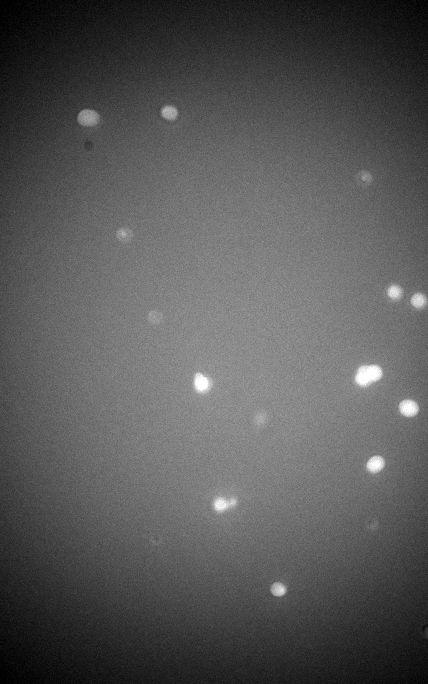

Supplement: Supplementary file 7 — Source data Fig. 5 [file 44318_2026_814_MOESM7_ESM.zip › Figure 5 Raw data/Figure 5 B/SClow ATP CKII/Alonso_CKi_CKii_test_sample_12.5uM_NSP2sec_12.5uM_SClow__CKii_A_30min_time_posXY0_channels_t1_posZ0.tif]

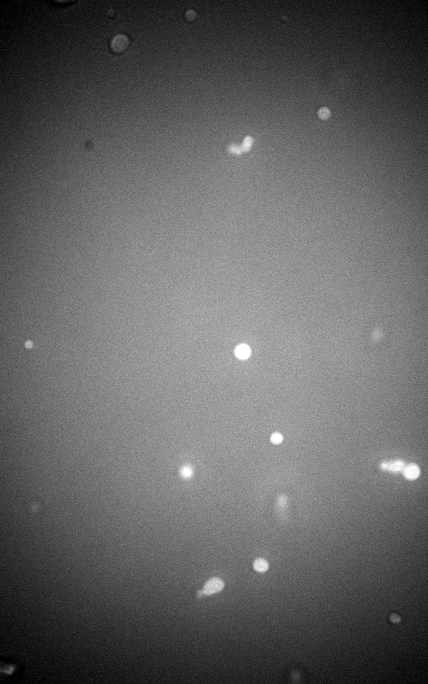

Supplement: Supplementary file 7 — Source data Fig. 5 [file 44318_2026_814_MOESM7_ESM.zip › Figure 5 Raw data/Figure 5 B/SClow ATP CKII/Alonso_SClow_CKii_NSP2sec_sample_25uM_NSP2sec_25uM_SClow_CKii_ATP_sampleA2_posXY1_channels_t1_posZ0.tif]

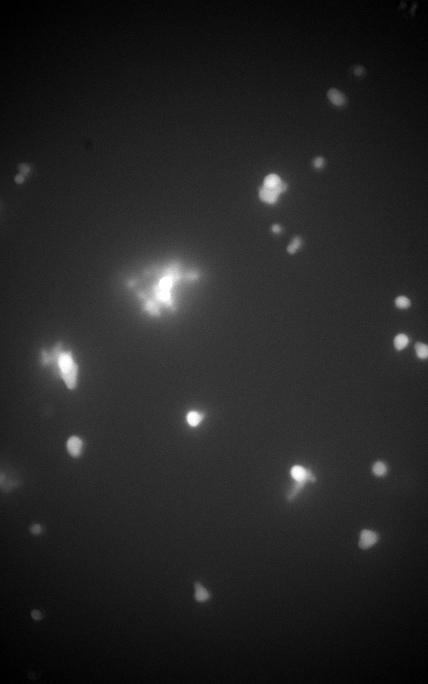

Supplement: Supplementary file 7 — Source data Fig. 5 [file 44318_2026_814_MOESM7_ESM.zip › Figure 5 Raw data/Figure 5 B/SClow ATP CKII/Alonso_SClow_CKii_sample_25uM_NSP2_25uM_SClow_CKii_ATP_sampleA_posXY2_channels_t1_posZ0.tif]

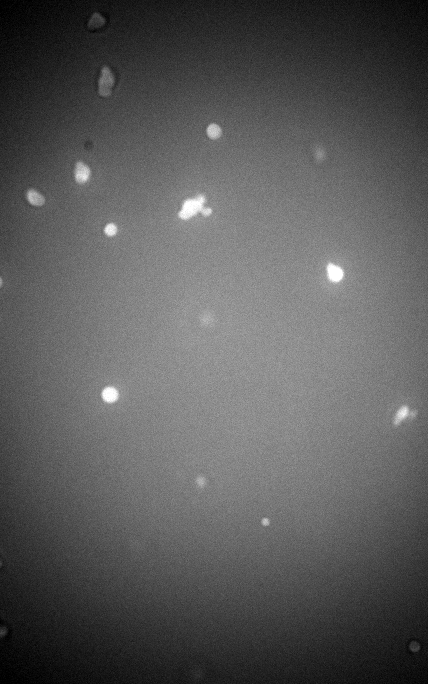

Supplement: Supplementary file 7 — Source data Fig. 5 [file 44318_2026_814_MOESM7_ESM.zip › Figure 5 Raw data/Figure 5 B/SClow ATP CKII/Alonso_SClow_CKii_NSP2sec_sample_25uM_NSP2sec_25uM_SClow_CKii_ATP_sampleA2_posXY3_channels_t1_posZ0.tif]

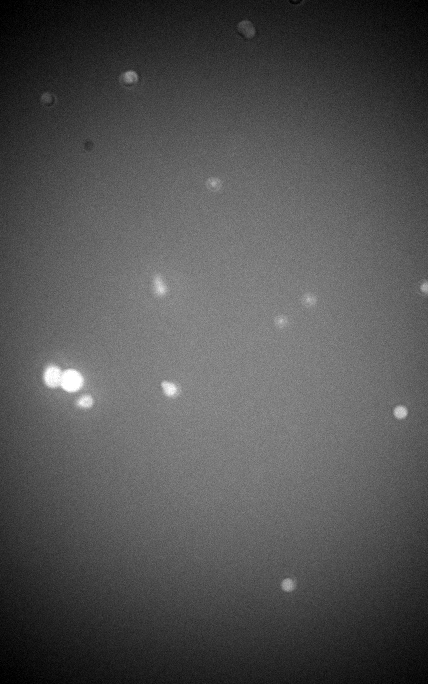

Supplement: Supplementary file 7 — Source data Fig. 5 [file 44318_2026_814_MOESM7_ESM.zip › Figure 5 Raw data/Figure 5 B/SClow ATP CKII/Alonso_CKi_CKii_test_sample_12.5uM_NSP2sec_12.5uM_SClow__CKii_A_30min_time_posXY2_channels_t1_posZ0.tif]

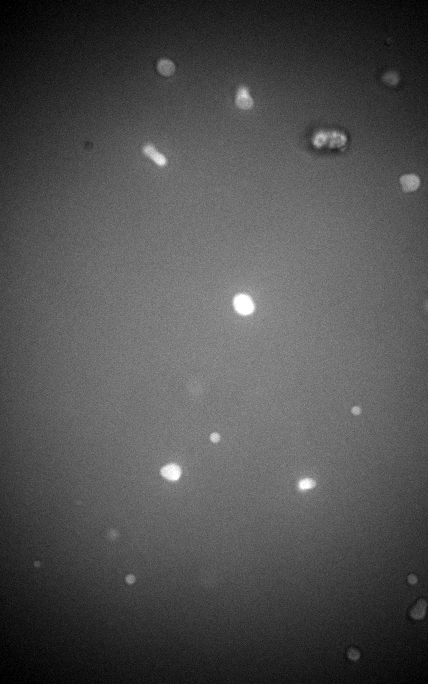

Supplement: Supplementary file 7 — Source data Fig. 5 [file 44318_2026_814_MOESM7_ESM.zip › Figure 5 Raw data/Figure 5 B/SClow ATP CKII/Alonso_SClow_CKii_NSP2sec_sample_25uM_NSP2sec_25uM_SClow_CKii_ATP_sampleA2_posXY5_channels_t1_posZ0.tif]

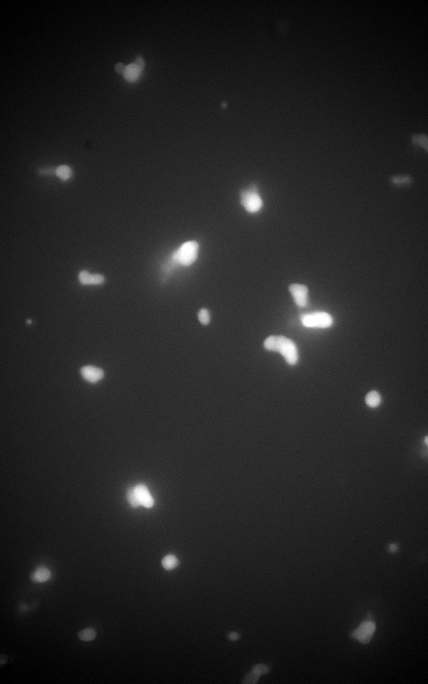

Supplement: Supplementary file 7 — Source data Fig. 5 [file 44318_2026_814_MOESM7_ESM.zip › Figure 5 Raw data/Figure 5 B/SClow ATP CKII/Alonso_SClow_CKii_sample_25uM_NSP2_25uM_SClow_CKii_ATP_sampleA_posXY4_channels_t1_posZ0.tif]

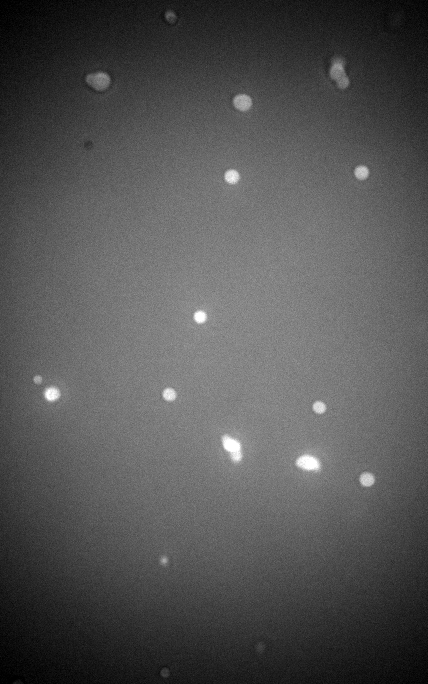

Supplement: Supplementary file 7 — Source data Fig. 5 [file 44318_2026_814_MOESM7_ESM.zip › Figure 5 Raw data/Figure 5 B/SClow ATP CKII/Alonso_SClow_CKii_NSP2sec_sample_25uM_NSP2sec_25uM_SClow_CKii_ATP_sampleA2_posXY7_channels_t1_posZ0.tif]

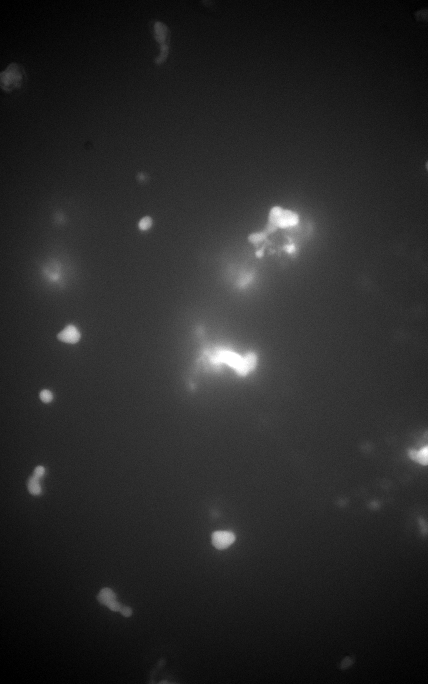

Supplement: Supplementary file 7 — Source data Fig. 5 [file 44318_2026_814_MOESM7_ESM.zip › Figure 5 Raw data/Figure 5 B/SClow ATP CKII/Alonso_SClow_CKii_sample_25uM_NSP2_25uM_SClow_CKii_ATP_sampleA_posXY6_channels_t1_posZ0.tif]

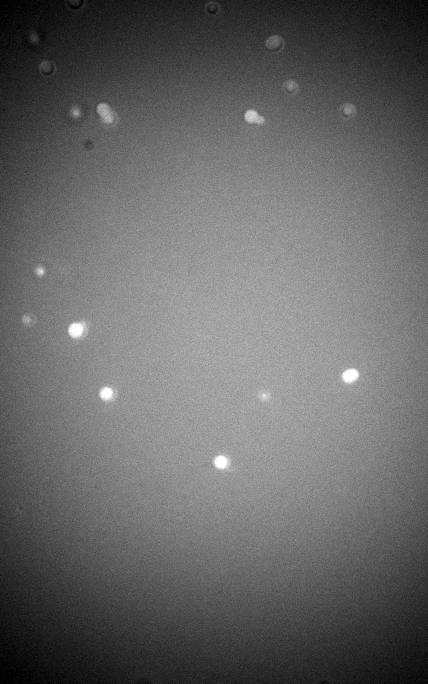

Supplement: Supplementary file 7 — Source data Fig. 5 [file 44318_2026_814_MOESM7_ESM.zip › Figure 5 Raw data/Figure 5 B/SClow ATP CKII/Alonso_CKi_CKii_test_sample_12.5uM_NSP2sec_12.5uM_SClow__CKii_A_30min_time_posXY6_channels_t1_posZ0.tif]

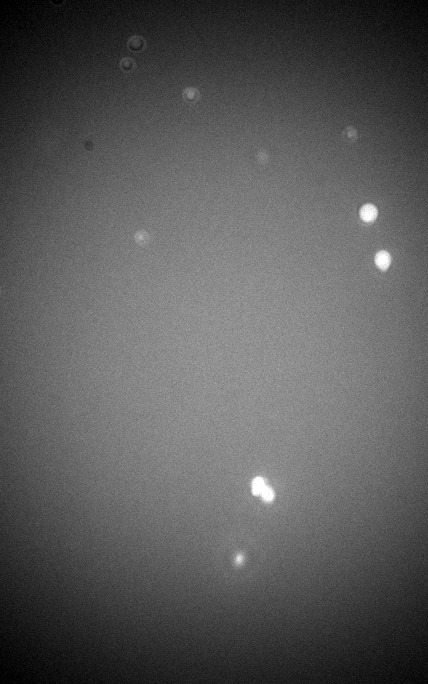

Supplement: Supplementary file 7 — Source data Fig. 5 [file 44318_2026_814_MOESM7_ESM.zip › Figure 5 Raw data/Figure 5 B/SClow ATP CKII/Alonso_CKi_CKii_test_sample_12.5uM_NSP2sec_12.5uM_SClow__CKii_A_30min_time_posXY1_channels_t1_posZ0.tif]

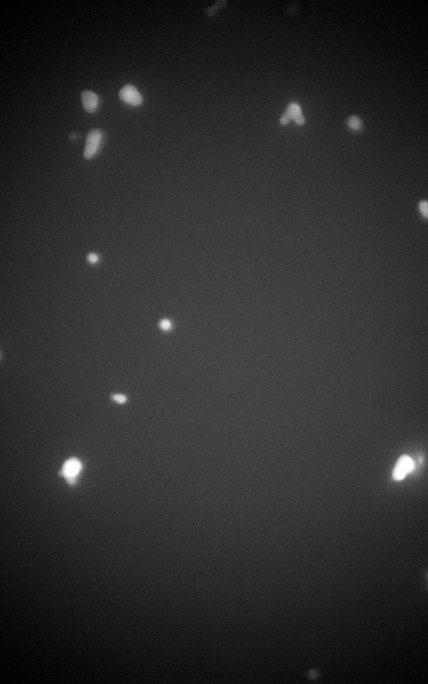

Supplement: Supplementary file 7 — Source data Fig. 5 [file 44318_2026_814_MOESM7_ESM.zip › Figure 5 Raw data/Figure 5 B/SClow ATP CKII/Alonso_SClow_CKii_sample_25uM_NSP2_25uM_SClow_CKii_ATP_sampleA_posXY1_channels_t1_posZ0.tif]

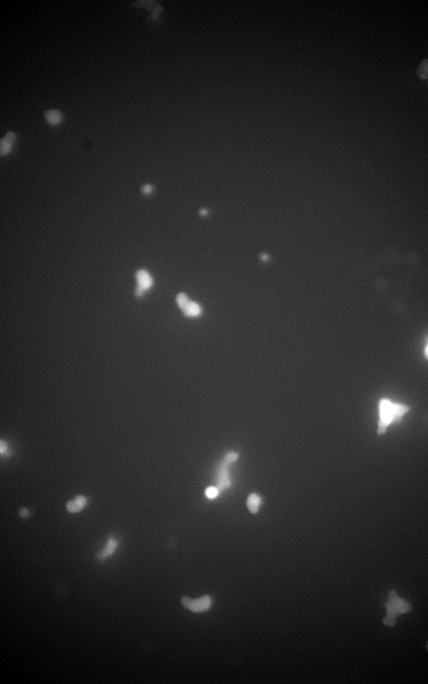

Supplement: Supplementary file 7 — Source data Fig. 5 [file 44318_2026_814_MOESM7_ESM.zip › Figure 5 Raw data/Figure 5 B/SClow ATP CKII/Alonso_SClow_CKii_sample_25uM_NSP2_25uM_SClow_CKii_ATP_sampleA_posXY3_channels_t1_posZ0.tif]

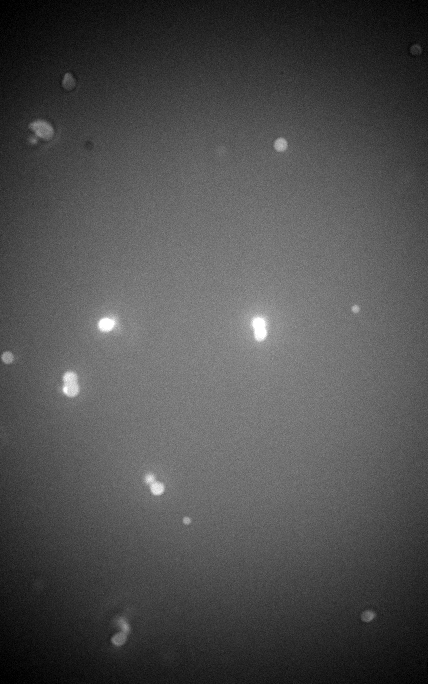

Supplement: Supplementary file 7 — Source data Fig. 5 [file 44318_2026_814_MOESM7_ESM.zip › Figure 5 Raw data/Figure 5 B/SClow ATP CKII/Alonso_SClow_CKii_NSP2sec_sample_25uM_NSP2sec_25uM_SClow_CKii_ATP_sampleA2_posXY2_channels_t1_posZ0.tif]

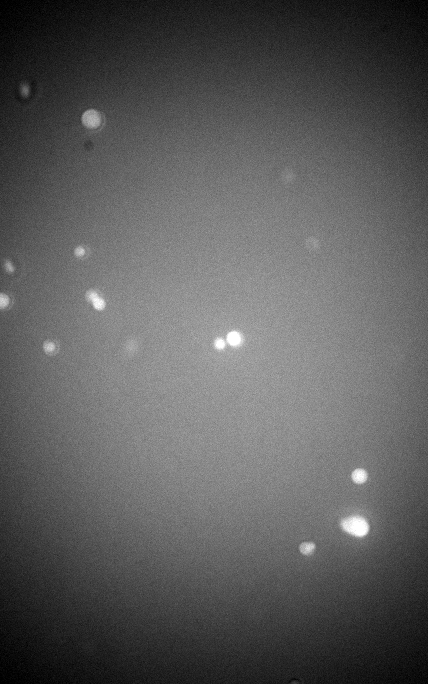

Supplement: Supplementary file 7 — Source data Fig. 5 [file 44318_2026_814_MOESM7_ESM.zip › Figure 5 Raw data/Figure 5 B/SClow ATP CKII/Alonso_CKi_CKii_test_sample_12.5uM_NSP2sec_12.5uM_SClow__CKii_A_30min_time_posXY5_channels_t1_posZ0.tif]

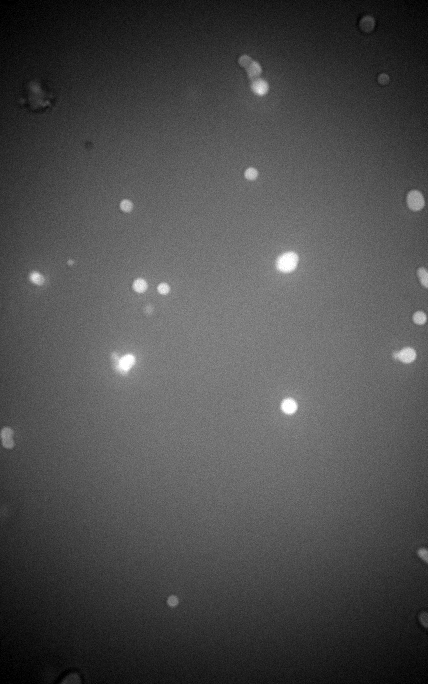

Supplement: Supplementary file 7 — Source data Fig. 5 [file 44318_2026_814_MOESM7_ESM.zip › Figure 5 Raw data/Figure 5 B/SClow ATP CKII/Alonso_SClow_CKii_NSP2sec_sample_25uM_NSP2sec_25uM_SClow_CKii_ATP_sampleA2_posXY4_channels_t1_posZ0.tif]

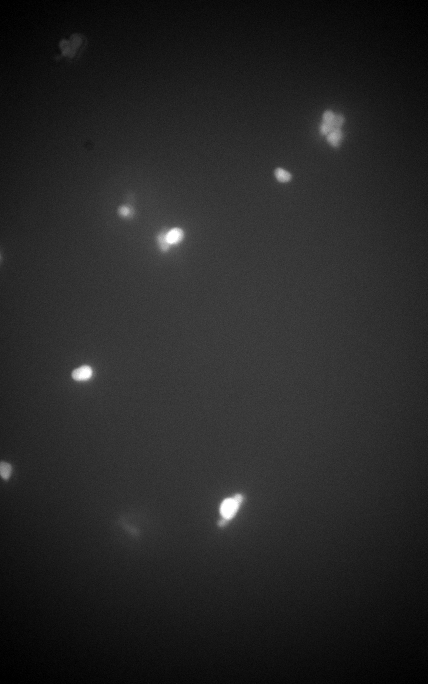

Supplement: Supplementary file 7 — Source data Fig. 5 [file 44318_2026_814_MOESM7_ESM.zip › Figure 5 Raw data/Figure 5 B/SClow ATP CKII/Alonso_SClow_CKii_sample_25uM_NSP2_25uM_SClow_CKii_ATP_sampleA_posXY5_channels_t1_posZ0.tif]

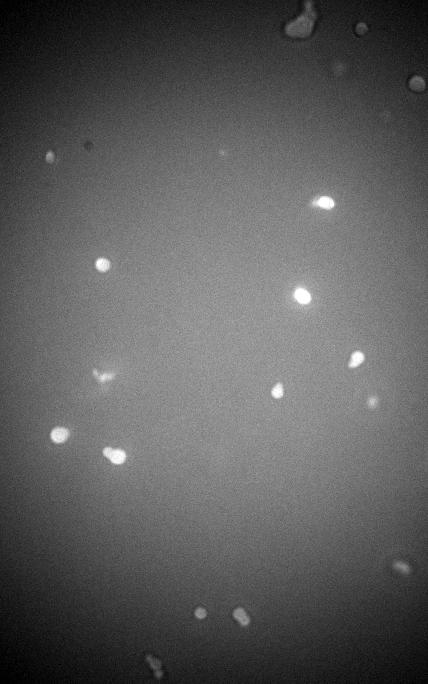

Supplement: Supplementary file 7 — Source data Fig. 5 [file 44318_2026_814_MOESM7_ESM.zip › Figure 5 Raw data/Figure 5 B/SClow ATP CKII/Alonso_SClow_CKii_NSP2sec_sample_25uM_NSP2sec_25uM_SClow_CKii_ATP_sampleA2_posXY6_channels_t1_posZ0.tif]

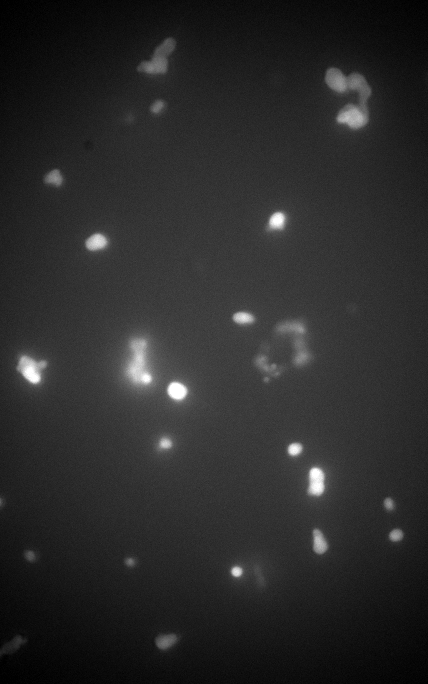

Supplement: Supplementary file 7 — Source data Fig. 5 [file 44318_2026_814_MOESM7_ESM.zip › Figure 5 Raw data/Figure 5 B/SClow ATP CKII/Alonso_SClow_CKii_sample_25uM_NSP2_25uM_SClow_CKii_ATP_sampleA_posXY7_channels_t1_posZ0.tif]

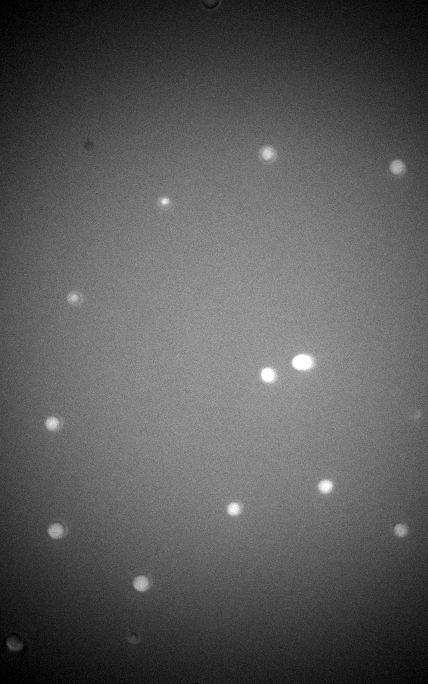

Supplement: Supplementary file 7 — Source data Fig. 5 [file 44318_2026_814_MOESM7_ESM.zip › Figure 5 Raw data/Figure 5 B/SClow ATP CKII/Alonso_CKi_CKii_test_sample_12.5uM_NSP2sec_12.5uM_SClow__CKii_A_30min_time_posXY7_channels_t1_posZ0.tif]

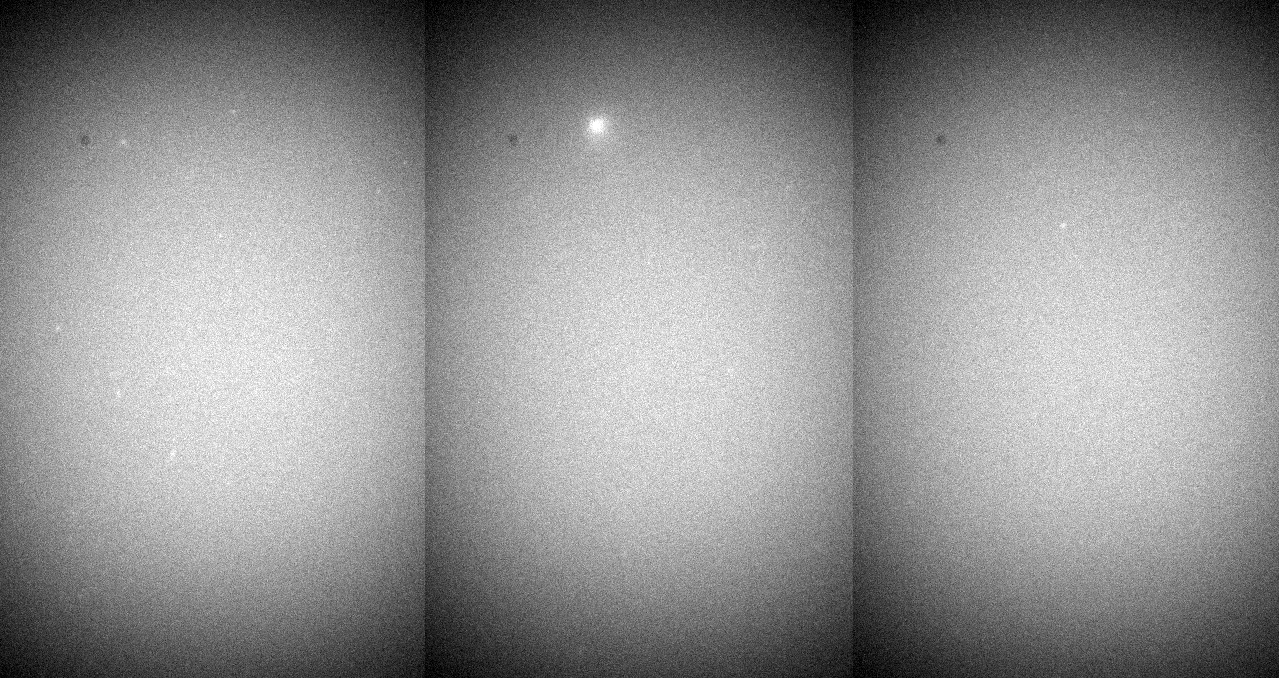

Supplement: Supplementary file 7 — Source data Fig. 5 [file 44318_2026_814_MOESM7_ESM.zip › Figure 5 Raw data/Figure 5 B/RF IDR scans/25uM-NSP2-RF-WT-A488_0uM-NSP5-RF-WT_50uM-NSP5-RF-DeltaC-10min-scan-3.tif]

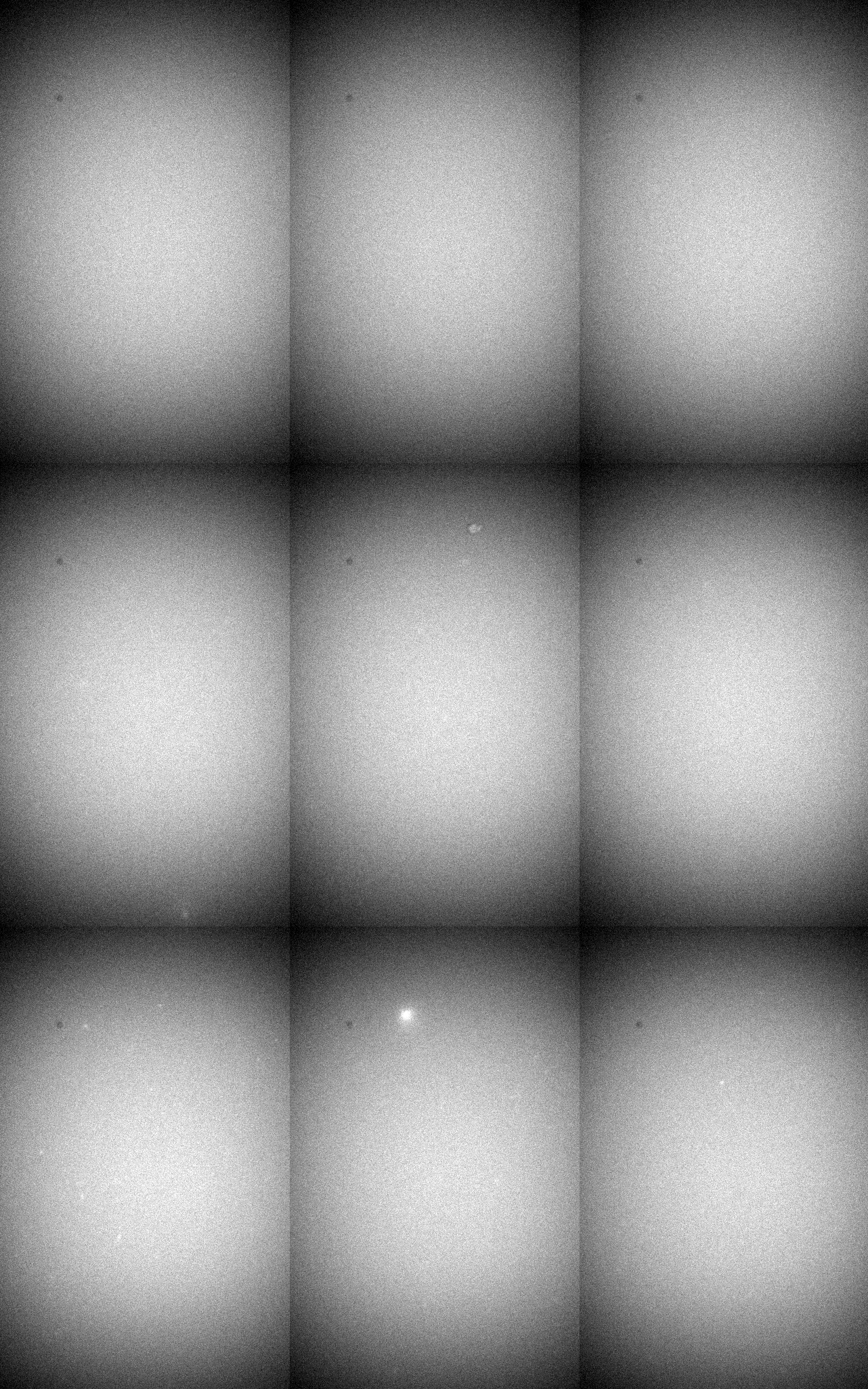

Supplement: Supplementary file 7 — Source data Fig. 5 [file 44318_2026_814_MOESM7_ESM.zip › Figure 5 Raw data/Figure 5 B/RF IDR scans/25uM-NSP2-RF-WT-A488_0uM-NSP5-RF-WT_50uM-NSP5-RF-DeltaC-10min-scan.tiff]

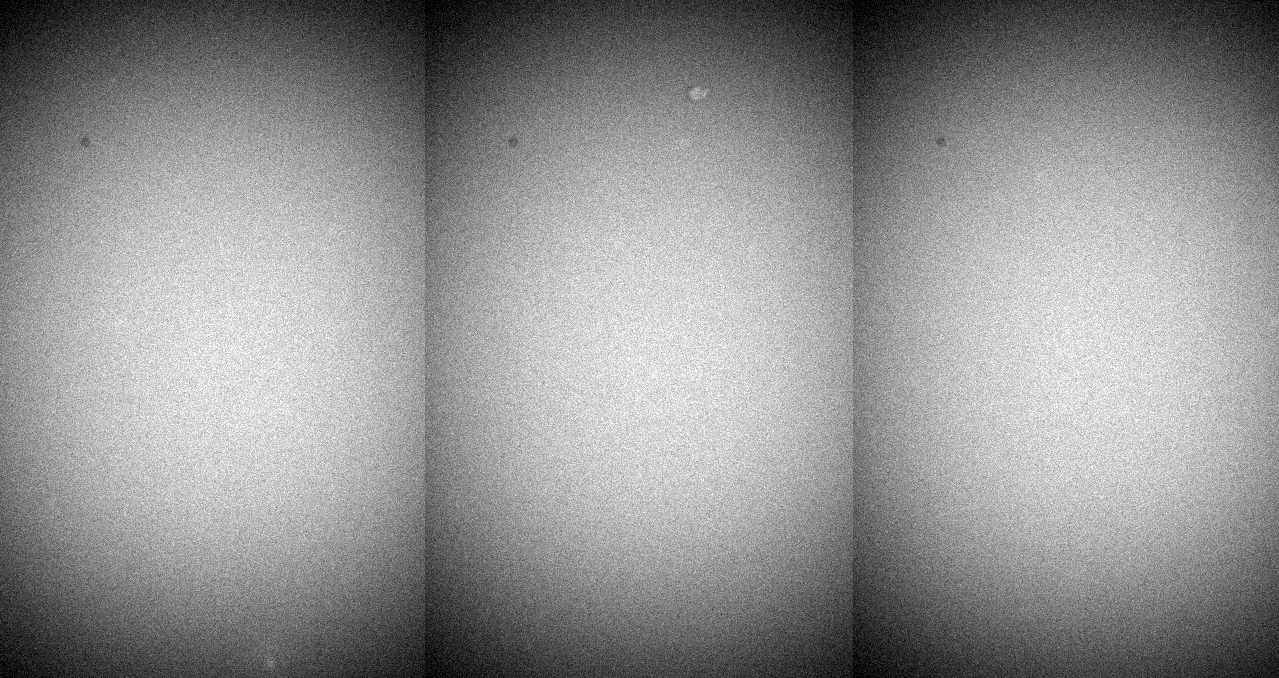

Supplement: Supplementary file 7 — Source data Fig. 5 [file 44318_2026_814_MOESM7_ESM.zip › Figure 5 Raw data/Figure 5 B/RF IDR scans/25uM-NSP2-RF-WT-A488_0uM-NSP5-RF-WT_25uM-NSP5-RF-DeltaC-10min-scan-2.tif]

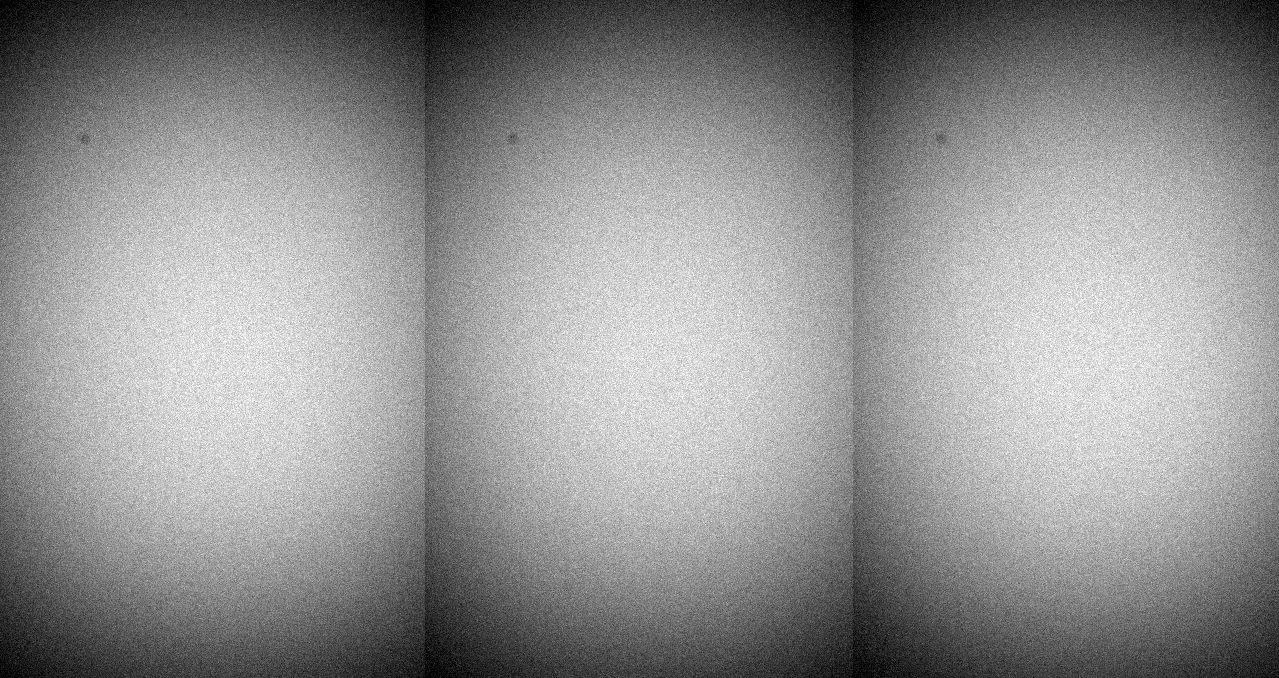

Supplement: Supplementary file 7 — Source data Fig. 5 [file 44318_2026_814_MOESM7_ESM.zip › Figure 5 Raw data/Figure 5 B/RF IDR scans/25uM-NSP2-RF-WT-A488_0uM-NSP5-RF-WT_25uM-NSP5-RF-DeltaC-10min-scan-1.tif]

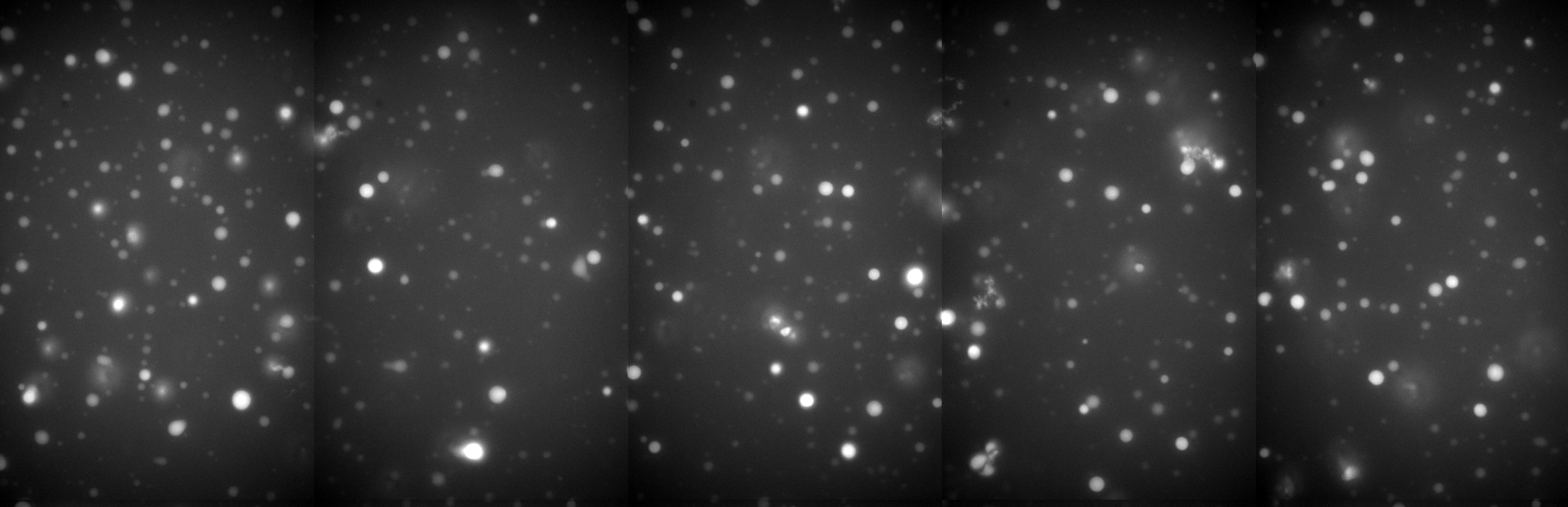

Supplement: Supplementary file 7 — Source data Fig. 5 [file 44318_2026_814_MOESM7_ESM.zip › Figure 5 Raw data/Figure 5 B/RF Scans/20uM-NSP2-RF-A488_20uM-NSP5-RF-scan-4.tif]

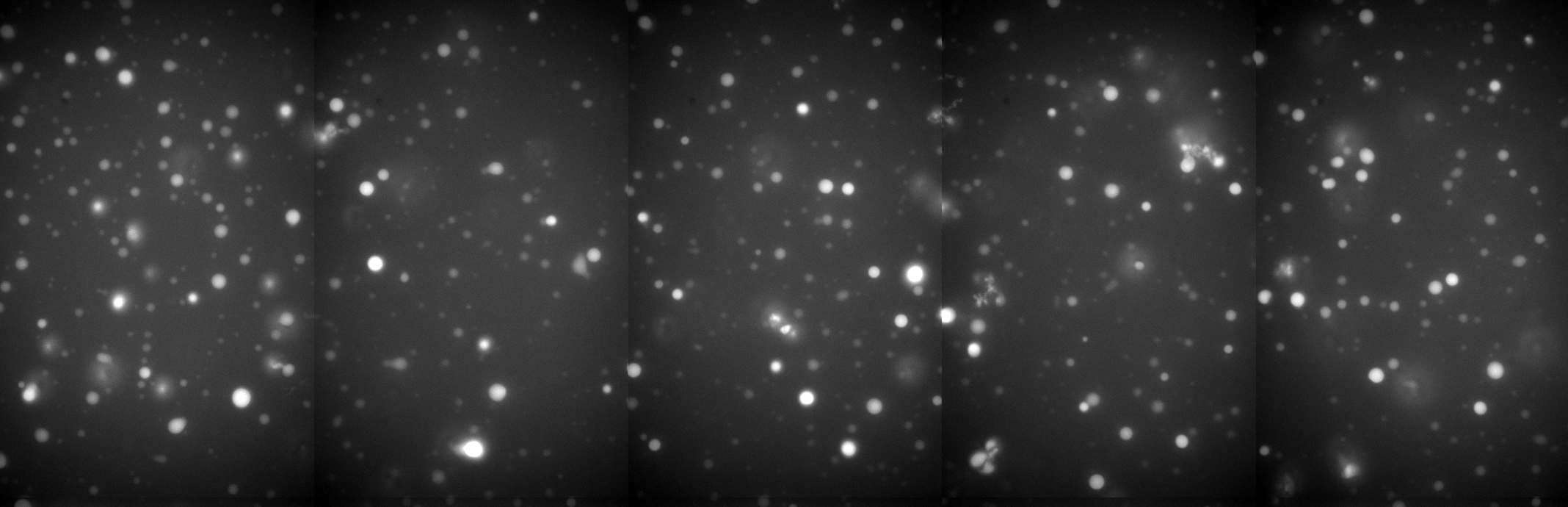

Supplement: Supplementary file 7 — Source data Fig. 5 [file 44318_2026_814_MOESM7_ESM.zip › Figure 5 Raw data/Figure 5 B/RF Scans/20uM-NSP2-RF-A488_20uM-NSP5-RF-scan-1.tif]

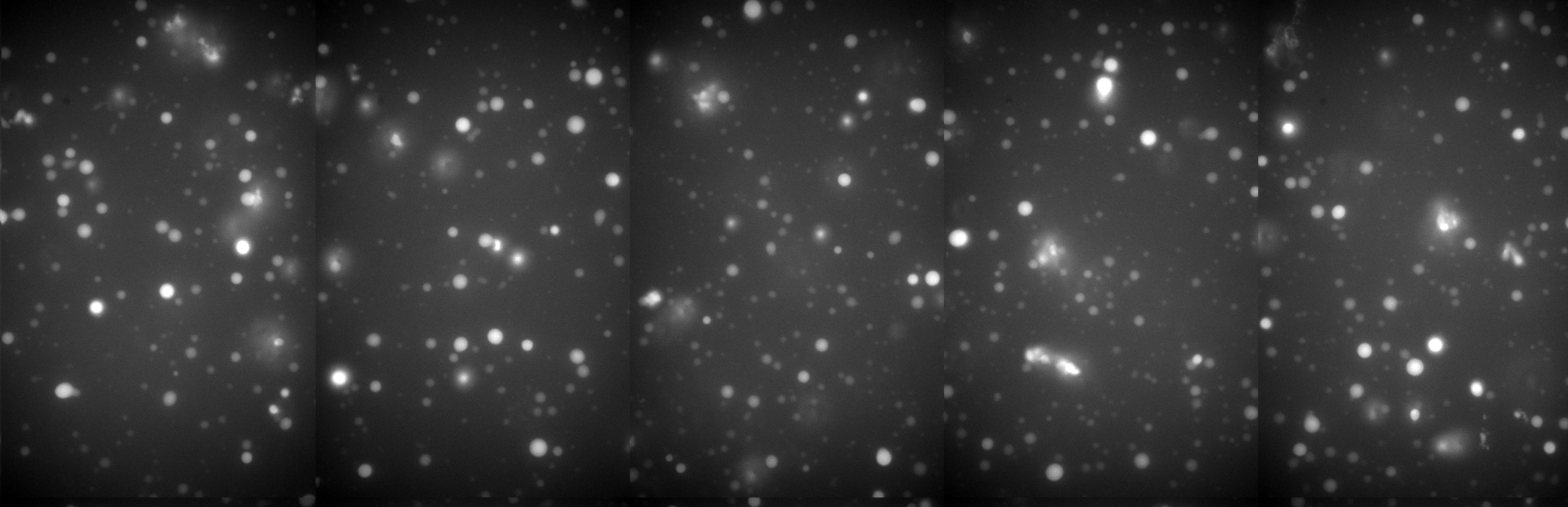

Supplement: Supplementary file 7 — Source data Fig. 5 [file 44318_2026_814_MOESM7_ESM.zip › Figure 5 Raw data/Figure 5 B/RF Scans/20uM-NSP2-RF-A488_20uM-NSP5-RF-scan-2.tif]

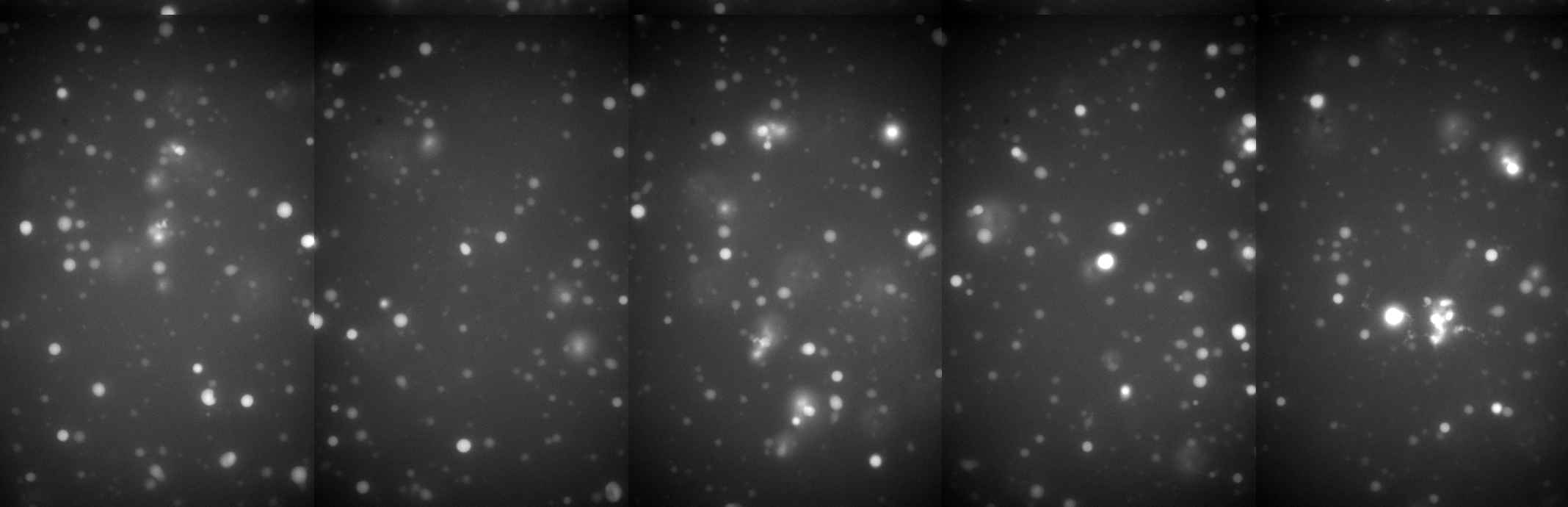

Supplement: Supplementary file 7 — Source data Fig. 5 [file 44318_2026_814_MOESM7_ESM.zip › Figure 5 Raw data/Figure 5 B/RF Scans/20uM-NSP2-RF-A488_20uM-NSP5-RF-scan-3.tif]

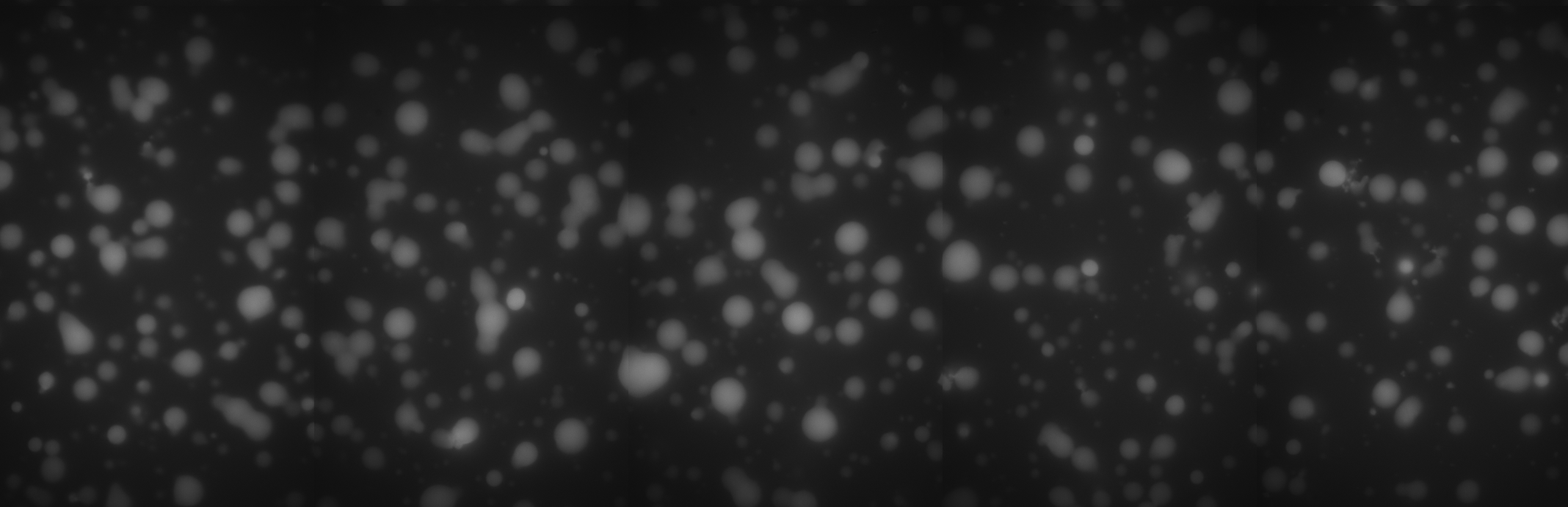

Supplement: Supplementary file 7 — Source data Fig. 5 [file 44318_2026_814_MOESM7_ESM.zip › Figure 5 Raw data/Figure 5 B/SA11 HP/2023_08_25_20uM-NSP2-A488_20uM_NSP5-SA11-HP-5min-3-1.tif]

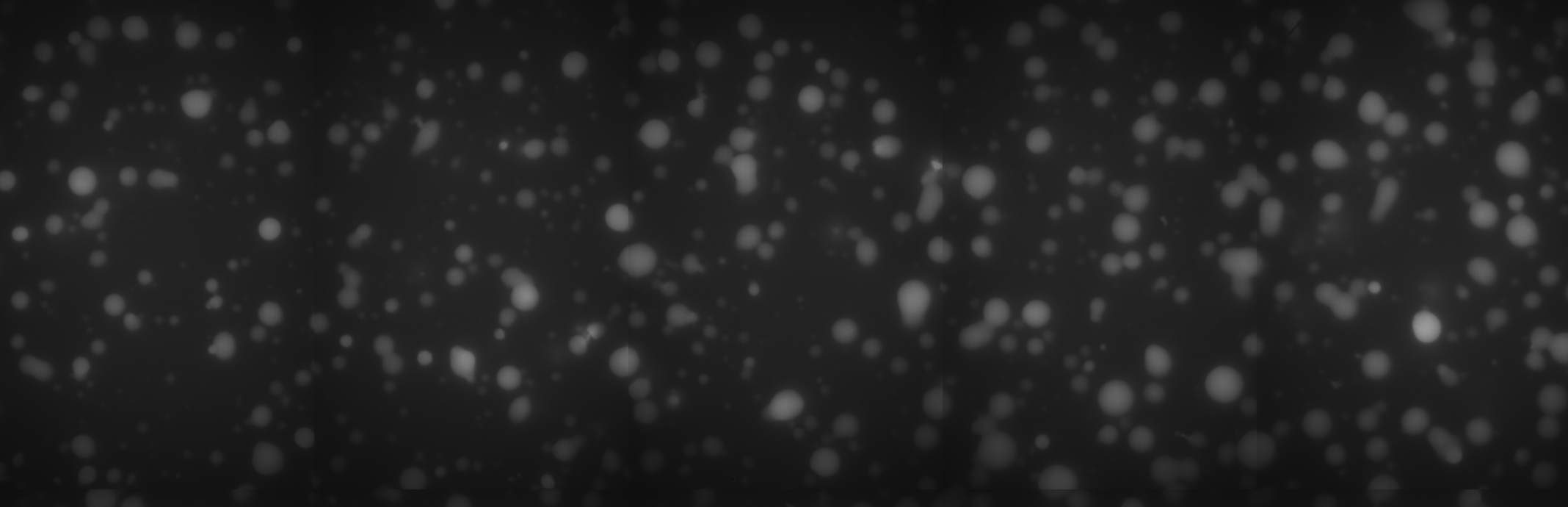

Supplement: Supplementary file 7 — Source data Fig. 5 [file 44318_2026_814_MOESM7_ESM.zip › Figure 5 Raw data/Figure 5 B/SA11 HP/2023_08_25_20uM-NSP2-A488_20uM_NSP5-SA11-HP-5min-3-3.tif]

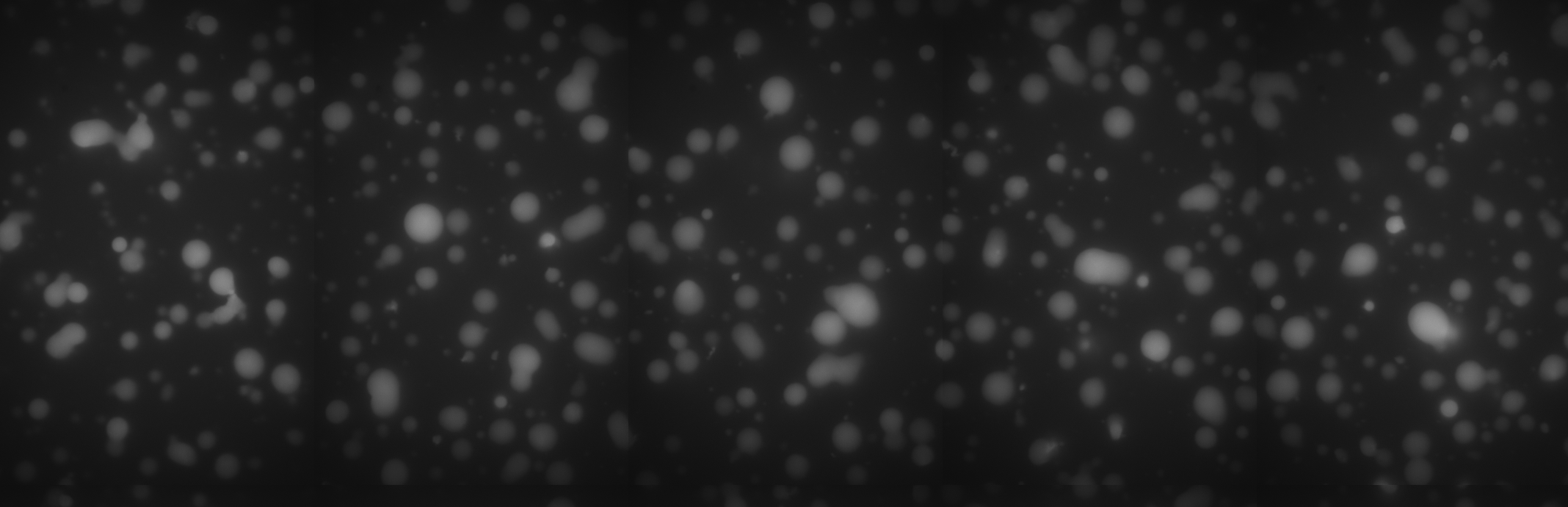

Supplement: Supplementary file 7 — Source data Fig. 5 [file 44318_2026_814_MOESM7_ESM.zip › Figure 5 Raw data/Figure 5 B/SA11 HP/2023_08_25_20uM-NSP2-A488_20uM_NSP5-SA11-HP-5min-3-2.tif]

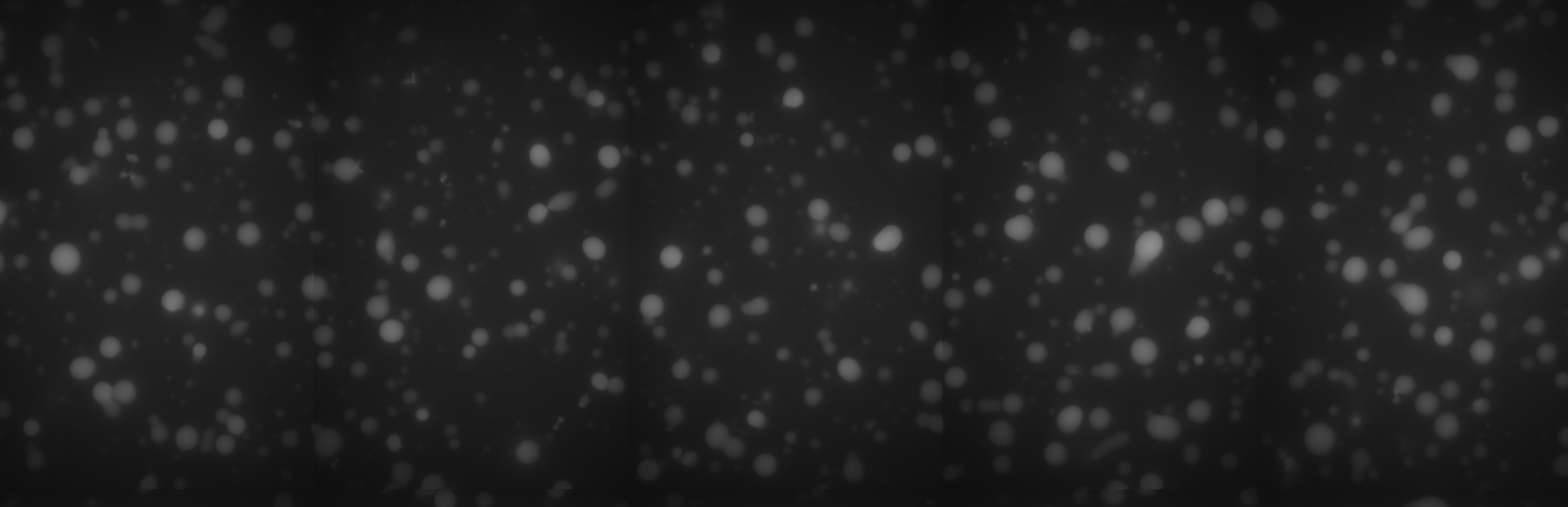

Supplement: Supplementary file 7 — Source data Fig. 5 [file 44318_2026_814_MOESM7_ESM.zip › Figure 5 Raw data/Figure 5 B/SA11 HP/2023_08_25_20uM-NSP2-A488_20uM_NSP5-SA11-HP-5min-3-4.tif]

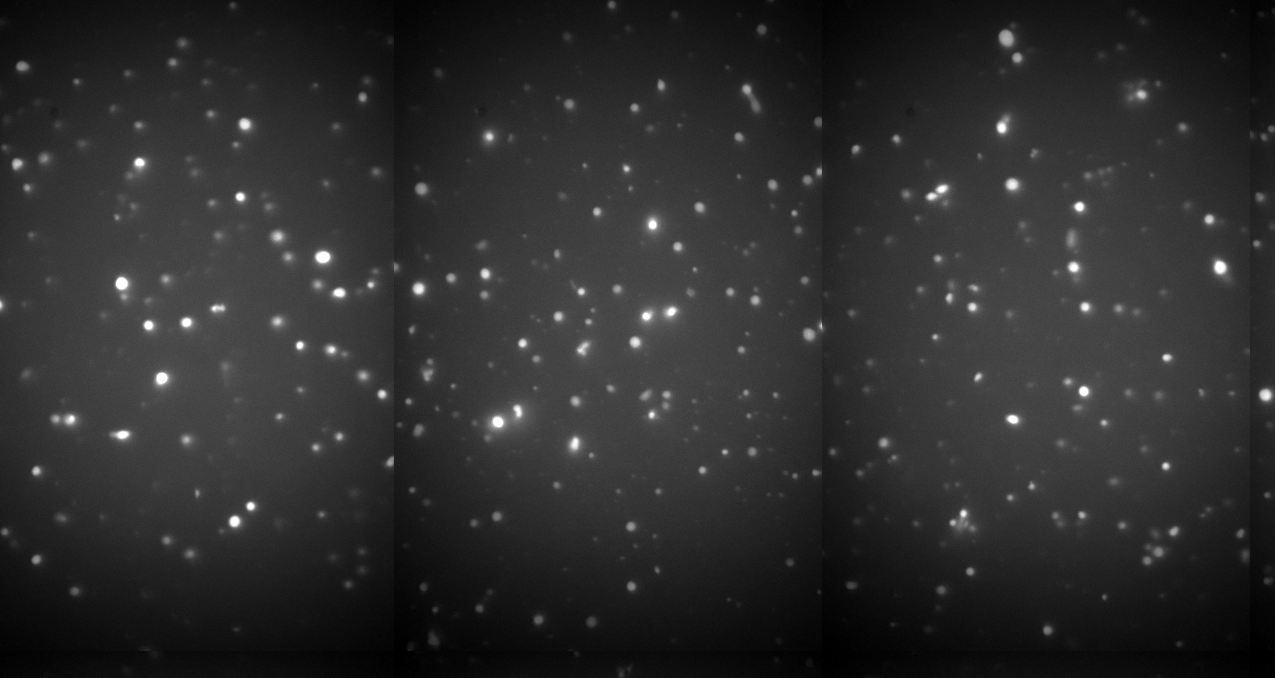

Supplement: Supplementary file 7 — Source data Fig. 5 [file 44318_2026_814_MOESM7_ESM.zip › Figure 5 Raw data/Figure 5 B/RF IDR HP scans/2024_10_01_25uM-NSP2-A488_25uM-NSP5-DeltaCHP_10min-scan-1.tif]

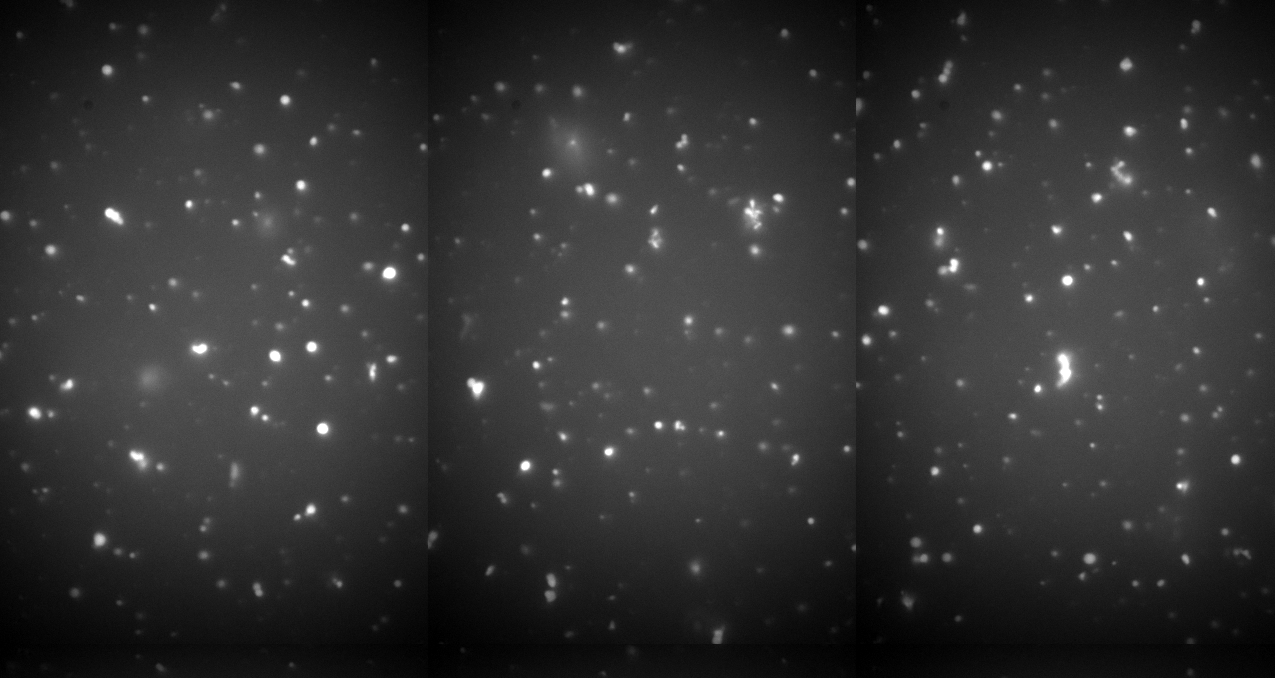

Supplement: Supplementary file 7 — Source data Fig. 5 [file 44318_2026_814_MOESM7_ESM.zip › Figure 5 Raw data/Figure 5 B/RF IDR HP scans/2024_10_01_25uM-NSP2-A488_25uM-NSP5-DeltaCHP_10min-scan-2.tif]

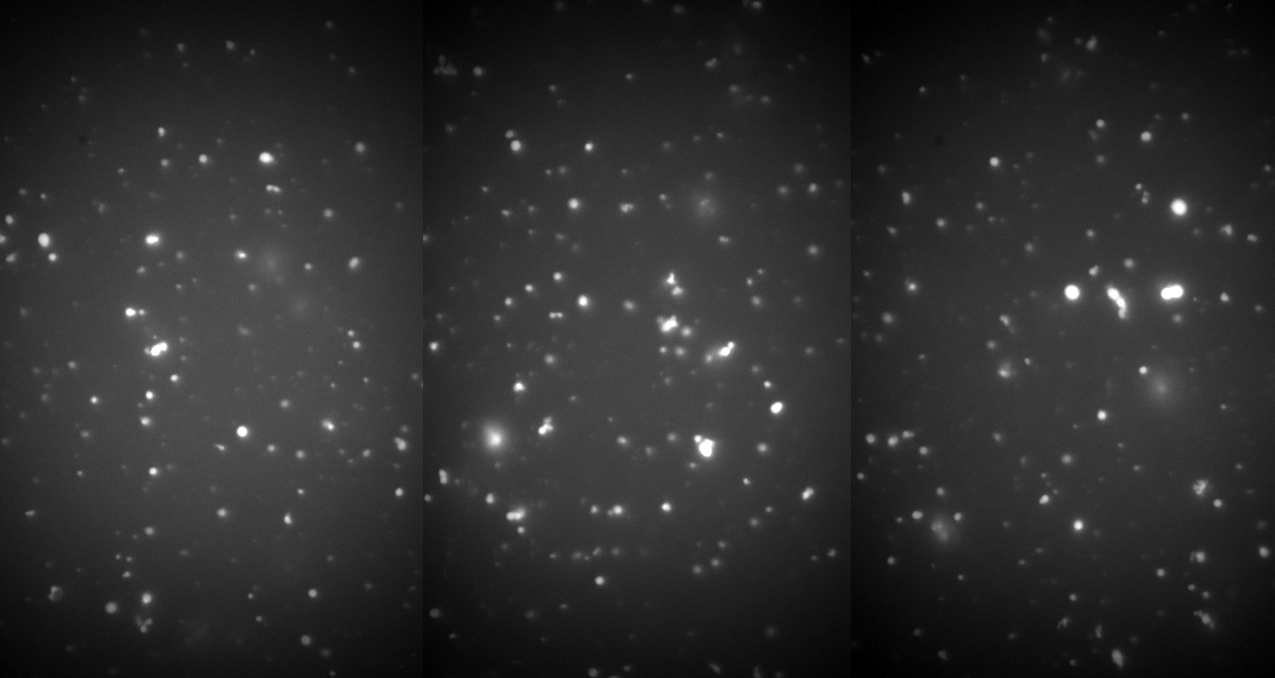

Supplement: Supplementary file 7 — Source data Fig. 5 [file 44318_2026_814_MOESM7_ESM.zip › Figure 5 Raw data/Figure 5 B/RF IDR HP scans/2024_10_01_25uM-NSP2-A488_25uM-NSP5-DeltaCHP_10min-scan-3.tif]

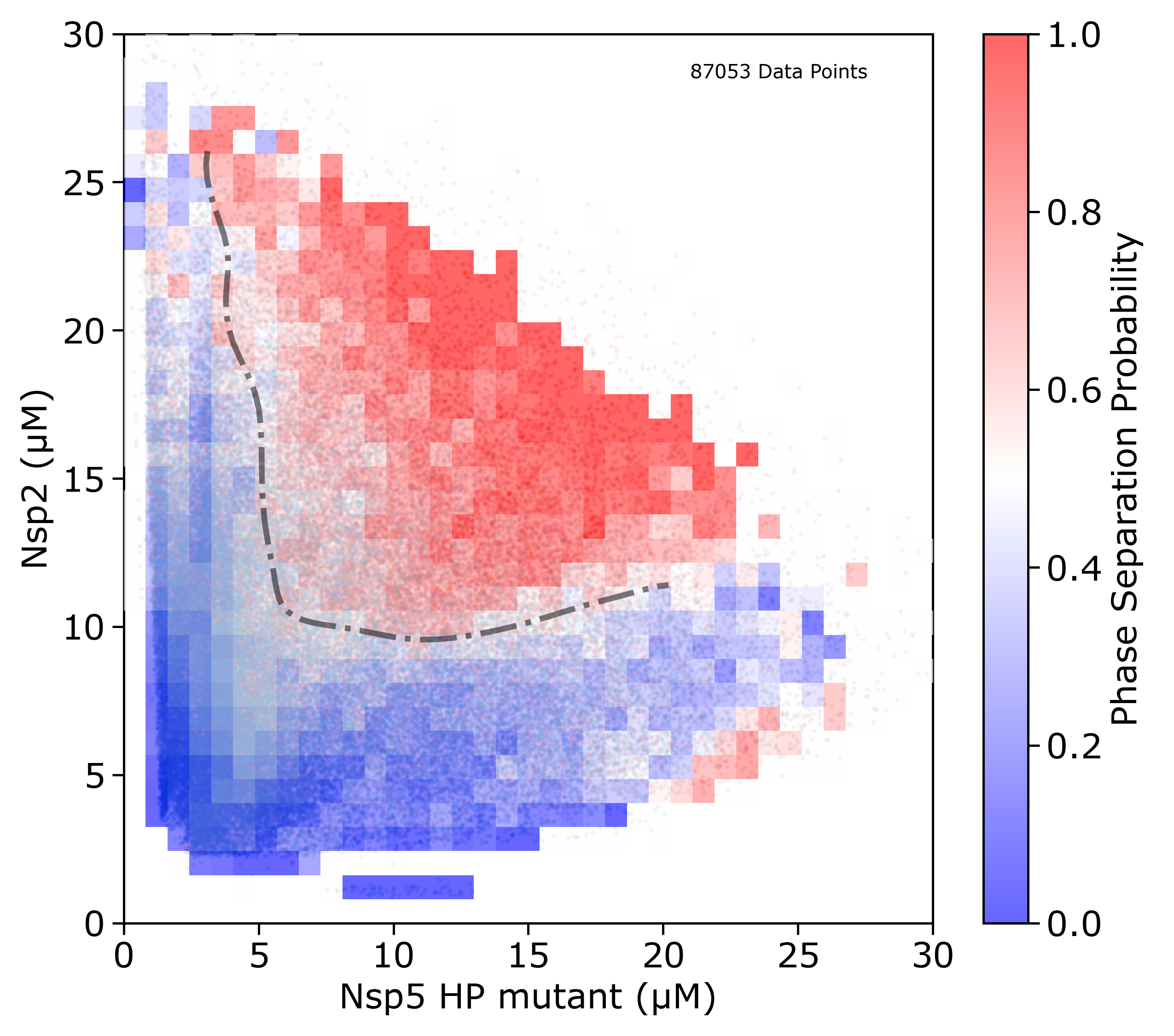

Supplement: Supplementary file 8 — Source data Fig. 6 [file 44318_2026_814_MOESM8_ESM.zip › Raw data/NSP5 SA11 HP/exp15_exp16.png]

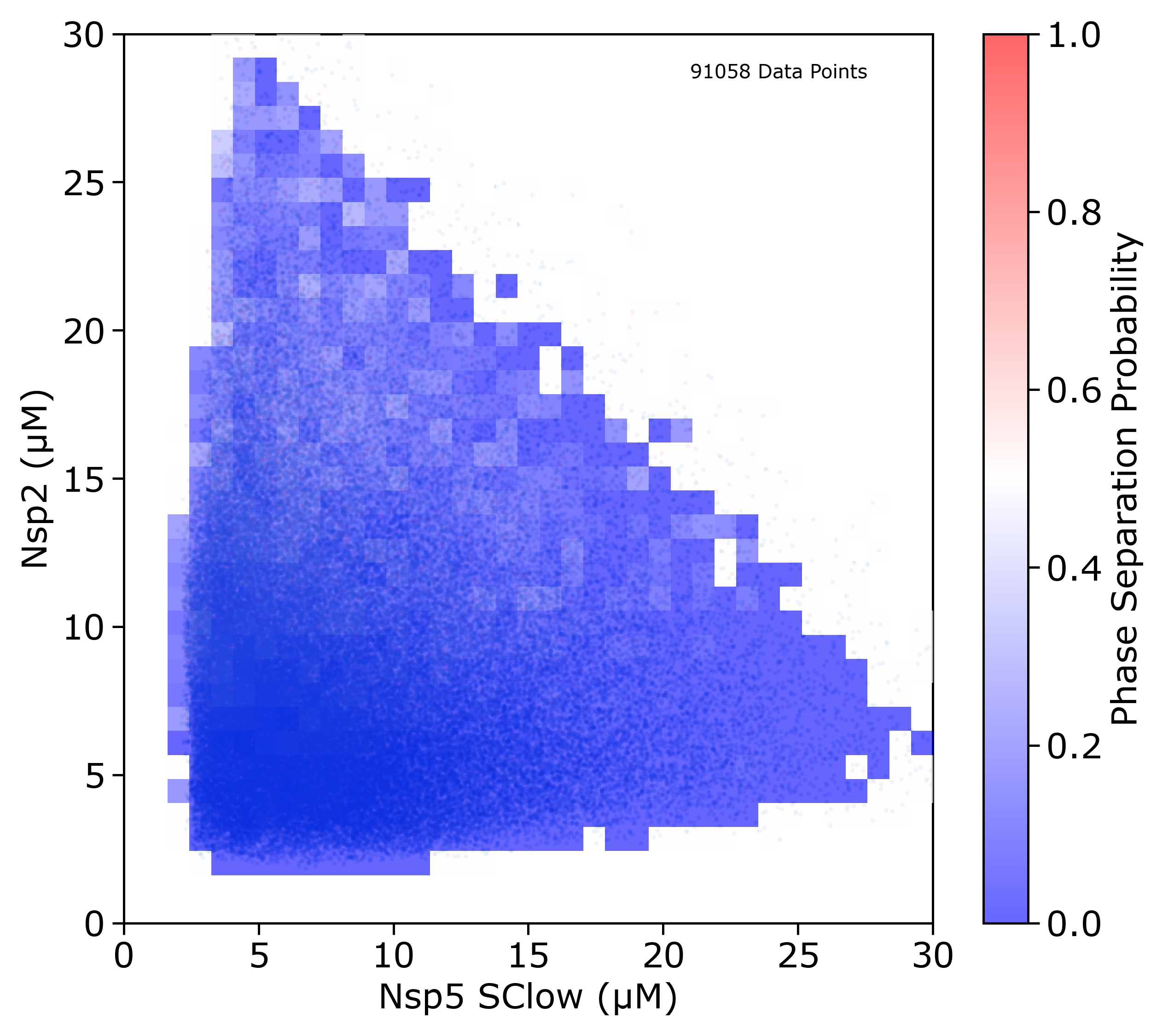

Supplement: Supplementary file 8 — Source data Fig. 6 [file 44318_2026_814_MOESM8_ESM.zip › Raw data/SClow/exp18.png]

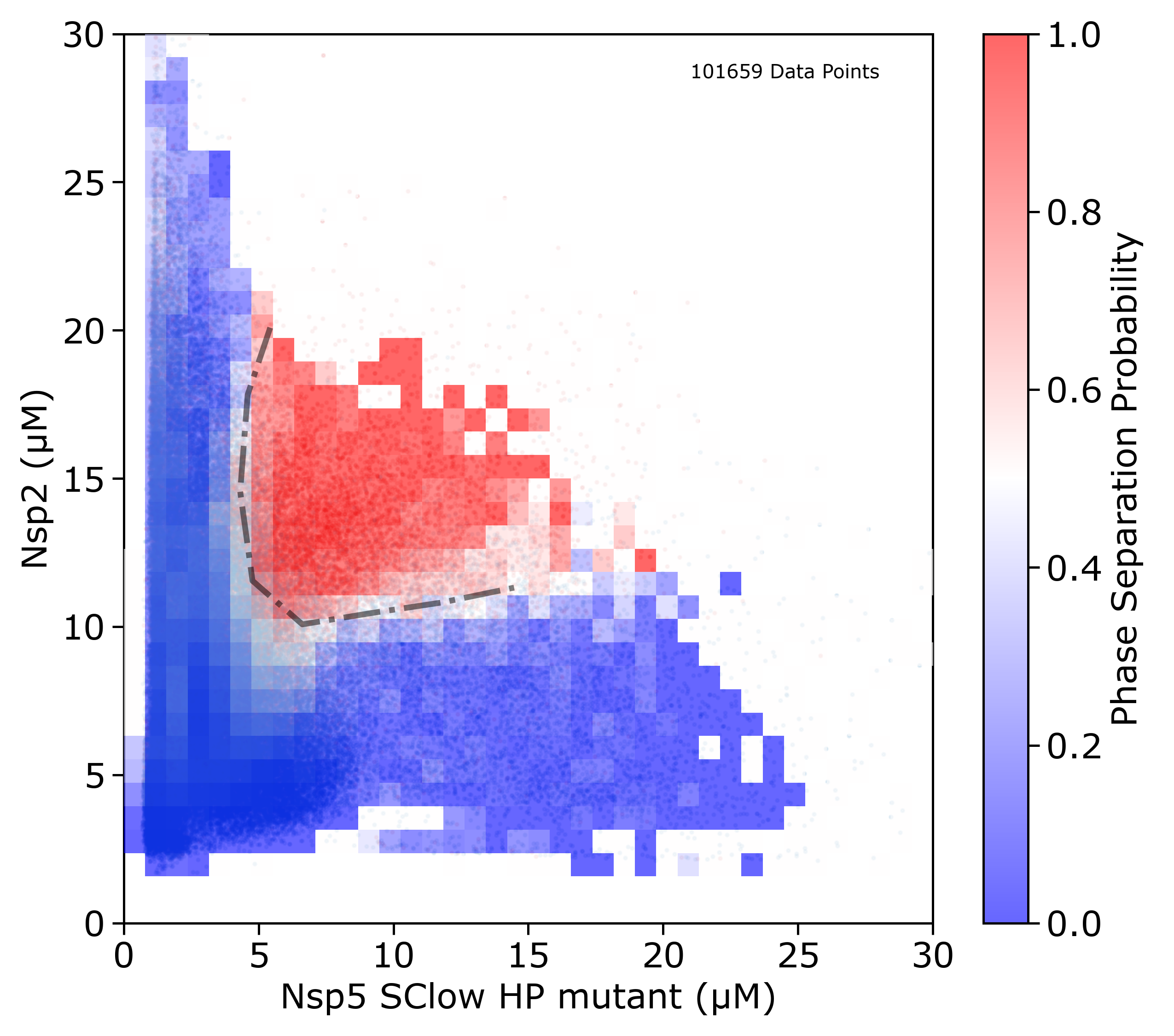

Supplement: Supplementary file 8 — Source data Fig. 6 [file 44318_2026_814_MOESM8_ESM.zip › Raw data/SClow HP/exp13_9exp.png]

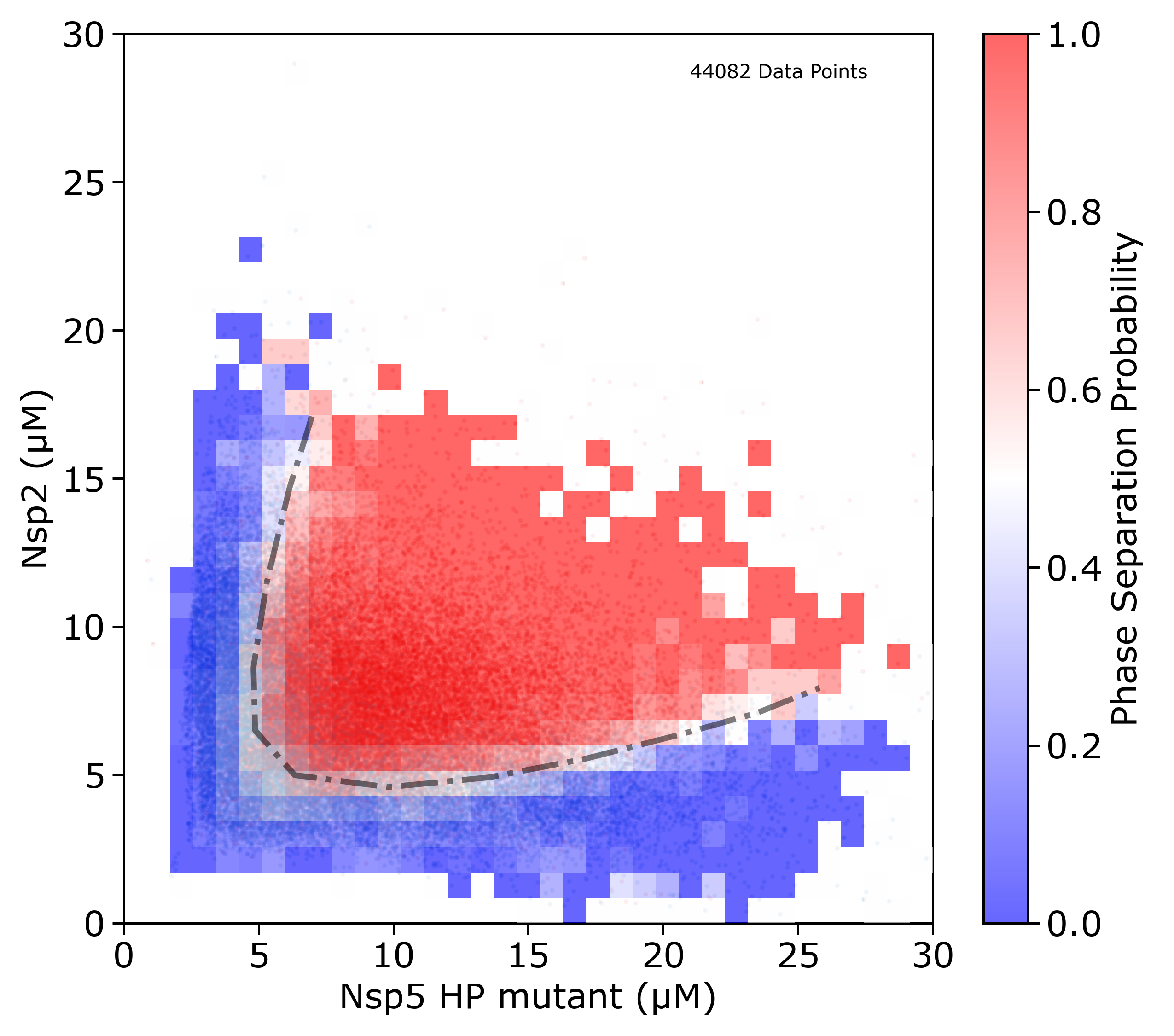

Supplement: Supplementary file 8 — Source data Fig. 6 [file 44318_2026_814_MOESM8_ESM.zip › Raw data/NSP5 RF HP/plot/7_.png]

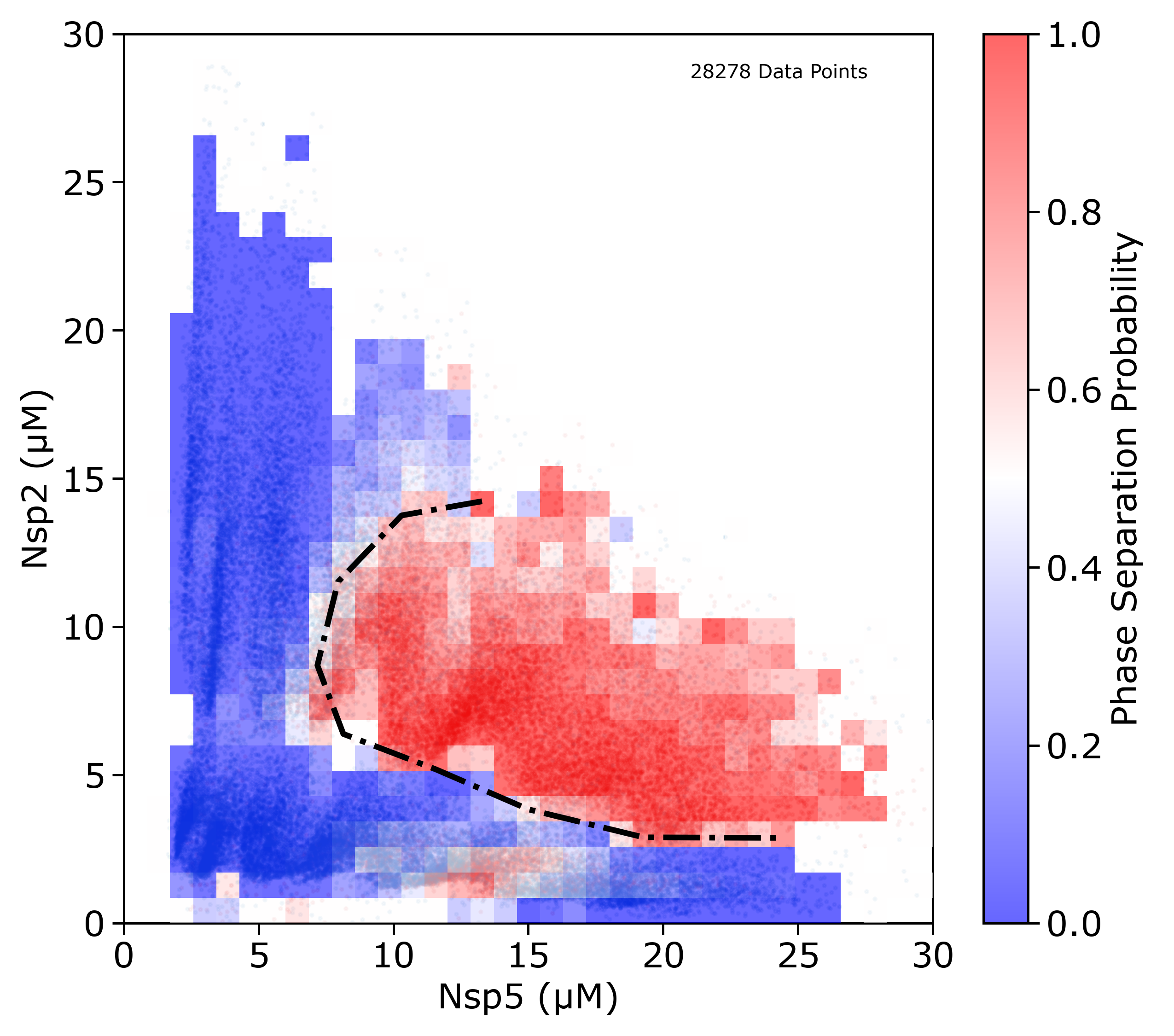

Supplement: Supplementary file 8 — Source data Fig. 6 [file 44318_2026_814_MOESM8_ESM.zip › Raw data/NSP5 SA11/plot/3_4.png]

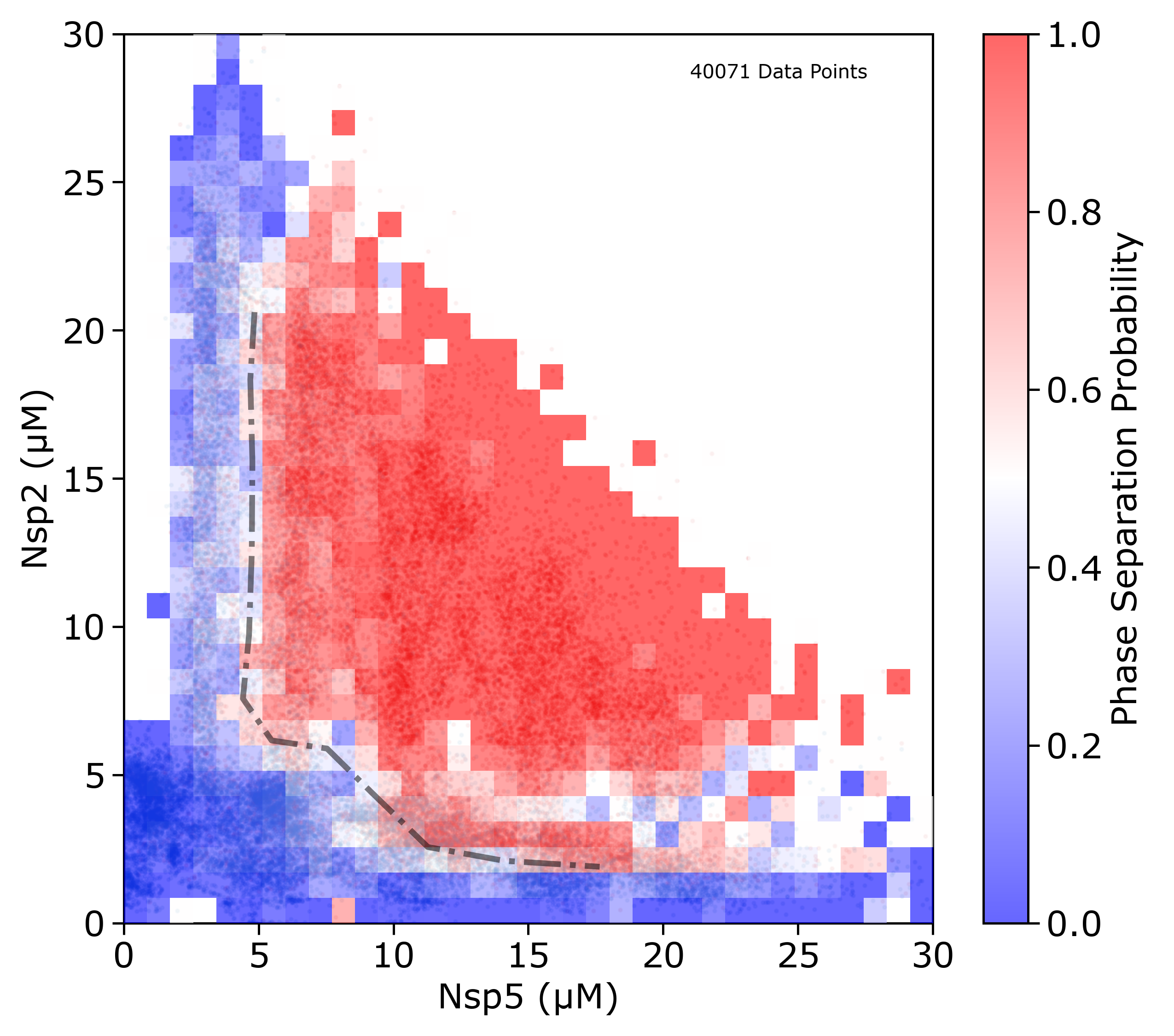

Supplement: Supplementary file 8 — Source data Fig. 6 [file 44318_2026_814_MOESM8_ESM.zip › Raw data/NSP5 RF/plot/1_2__.png]

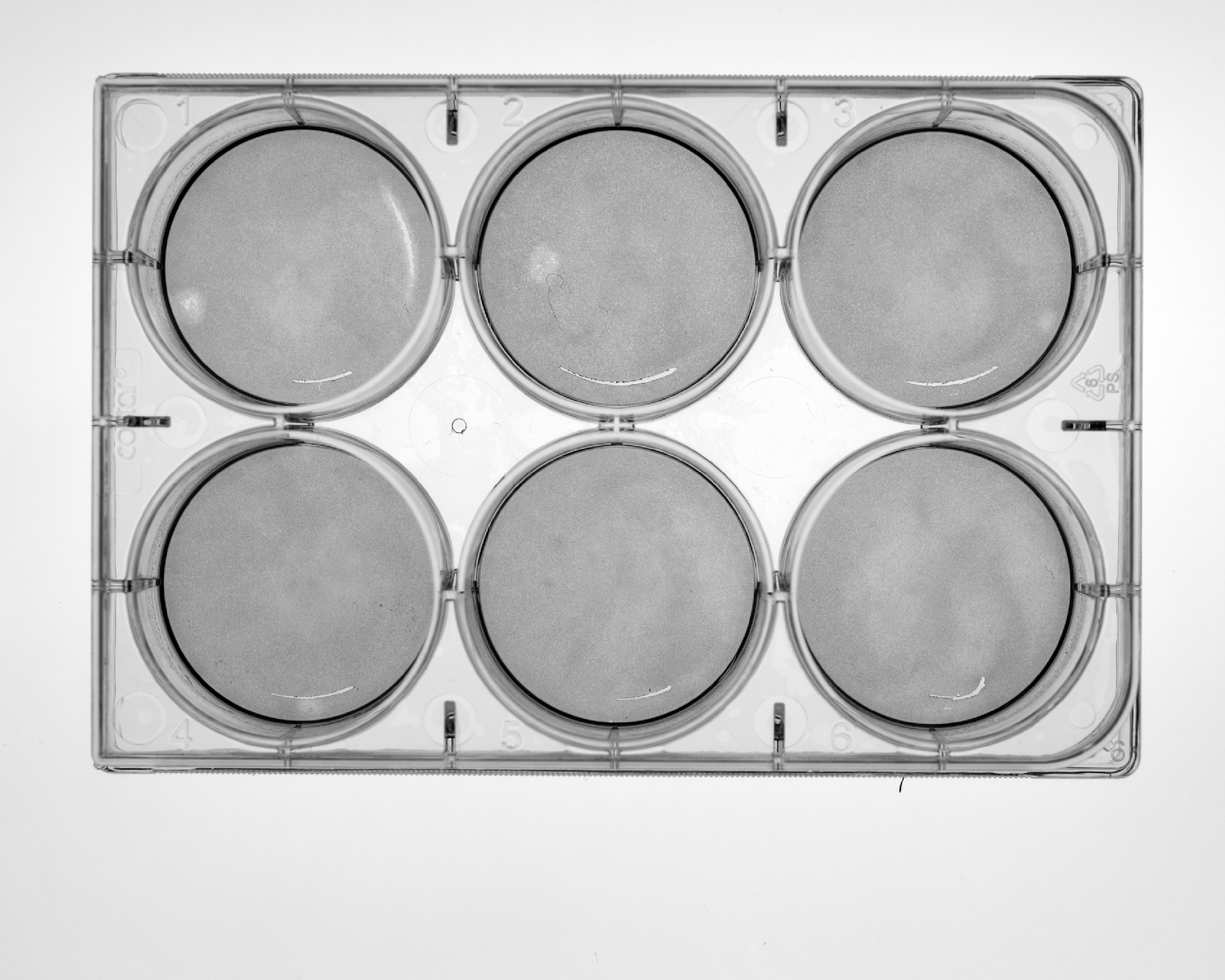

Supplement: Supplementary file 9 — Source data Fig. 7 [file 44318_2026_814_MOESM9_ESM.zip › Figure 8 raw/Figure 8 B/ma104 nsp5 sa11 s67a 10-1 to 10-5 Cyril 2025-02-06 7h38m53s(Coomassie Blue).tif]

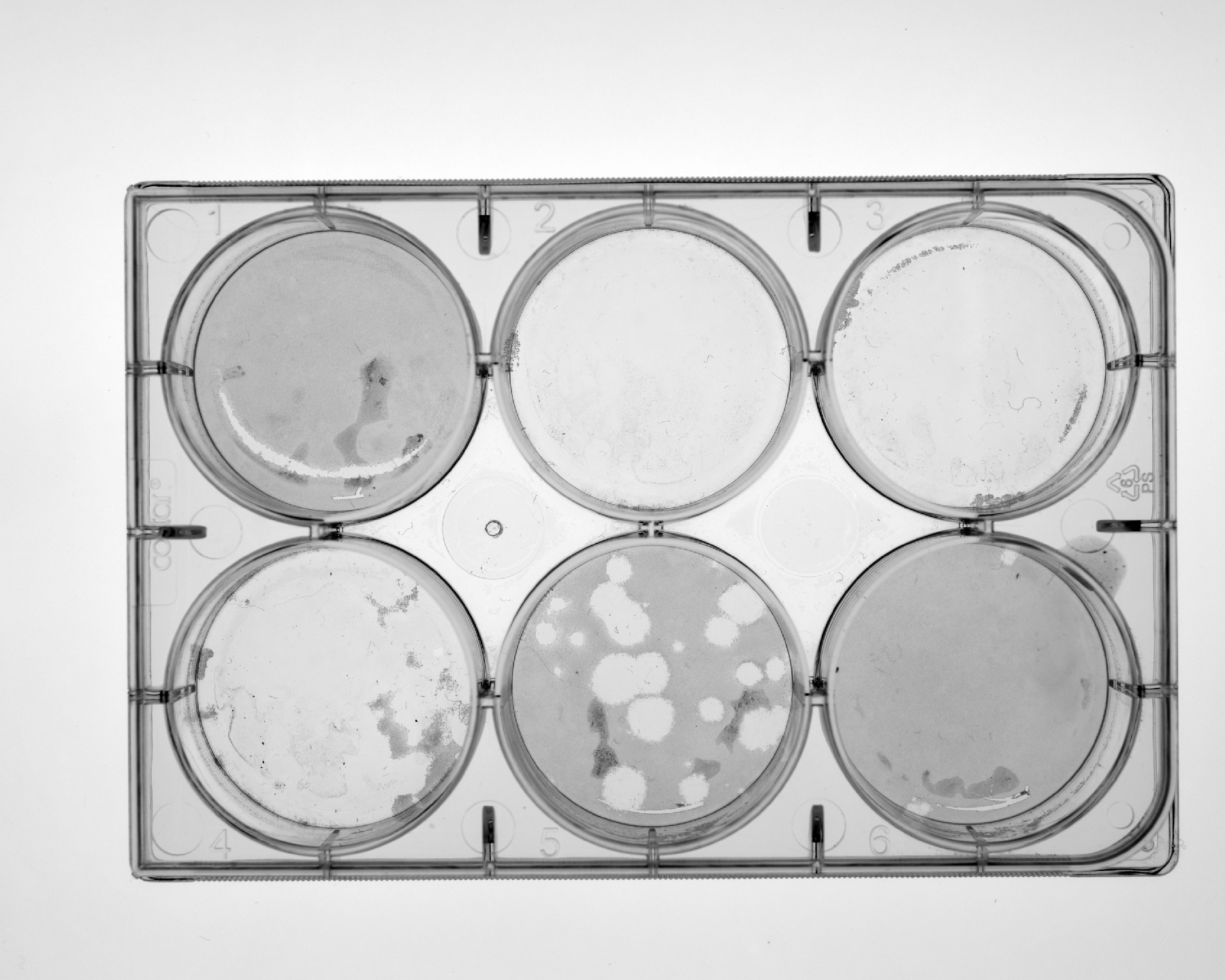

Supplement: Supplementary file 9 — Source data Fig. 7 [file 44318_2026_814_MOESM9_ESM.zip › Figure 8 raw/Figure 8 B/ma104nsp5 c2s 2025-02-03(Coomassie Blue).tif]

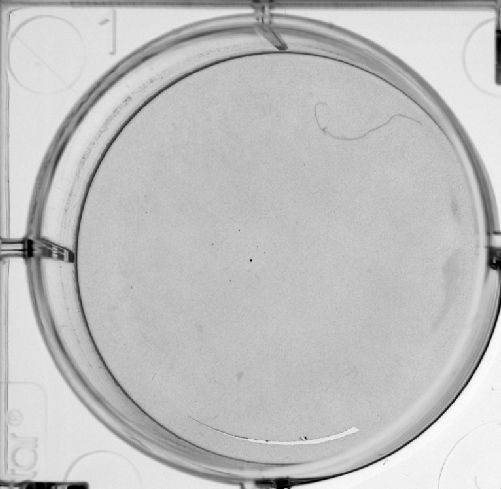

Supplement: Supplementary file 9 — Source data Fig. 7 [file 44318_2026_814_MOESM9_ESM.zip › Figure 8 raw/Figure 8 B/zoom 10-1 dilution ma104 nsp5 c2ss67a 2025-02-03(Coomassie Blue)-1.tif]

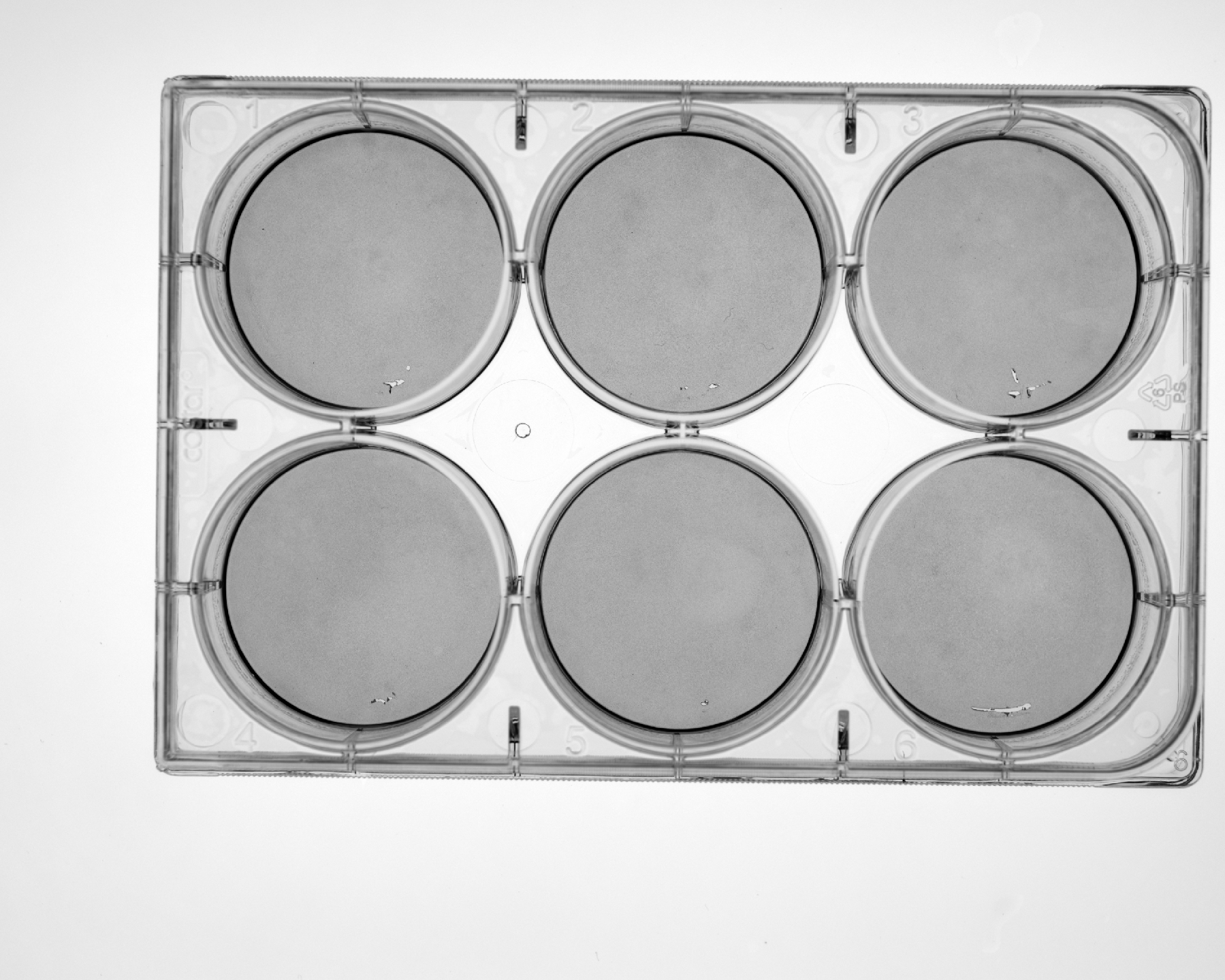

Supplement: Supplementary file 9 — Source data Fig. 7 [file 44318_2026_814_MOESM9_ESM.zip › Figure 8 raw/Figure 8 B/MA104 WT SA11 S67A 10-1 10-5 Cyril 2025-02-06 7h41m23s(Coomassie Blue).tif]

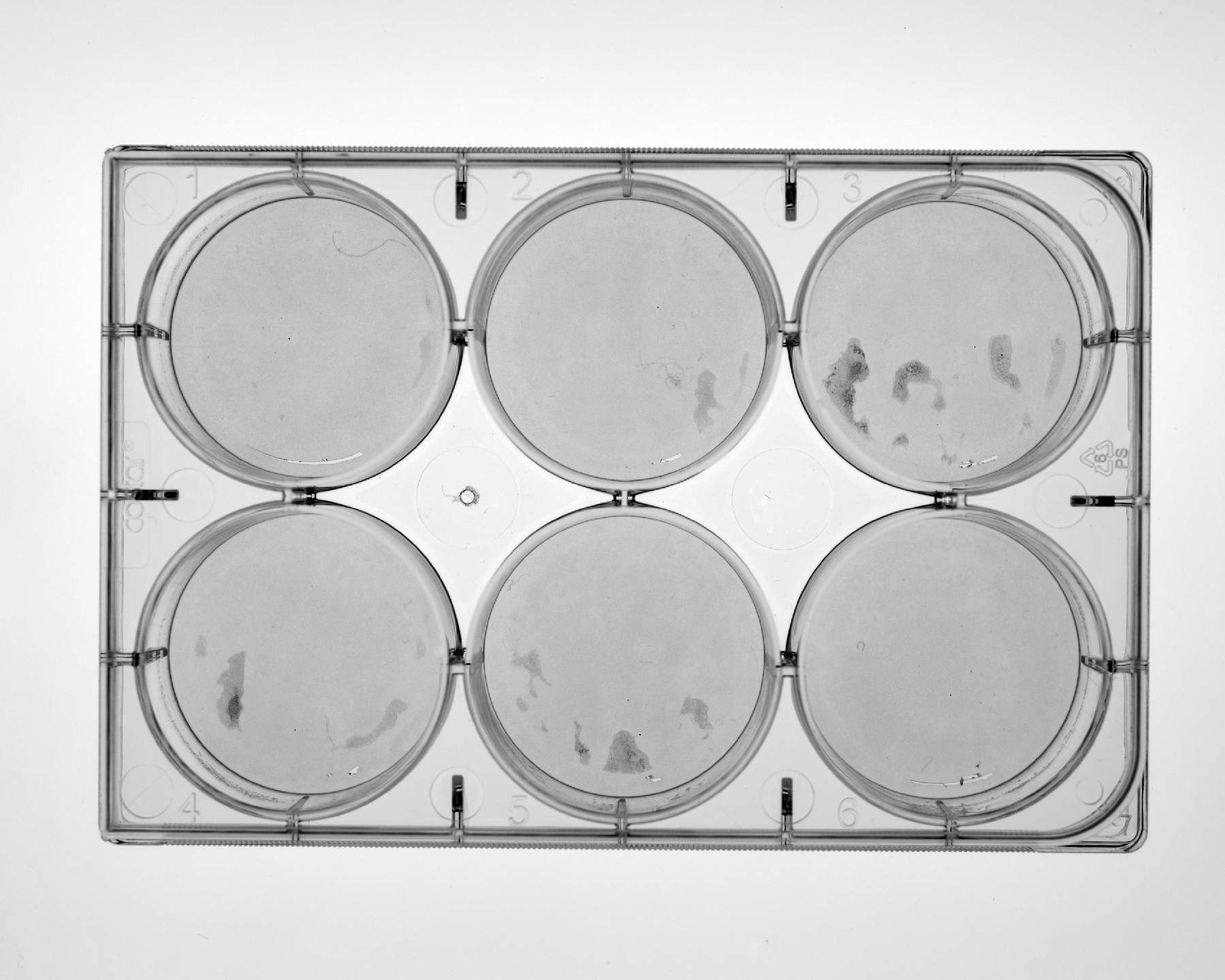

Supplement: Supplementary file 9 — Source data Fig. 7 [file 44318_2026_814_MOESM9_ESM.zip › Figure 8 raw/Figure 8 B/ma104 nsp5 c2ss67a 2025-02-03(Coomassie Blue).tif]

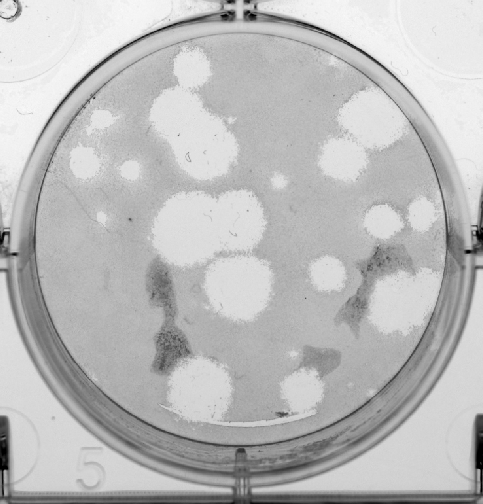

Supplement: Supplementary file 9 — Source data Fig. 7 [file 44318_2026_814_MOESM9_ESM.zip › Figure 8 raw/Figure 8 B/zoom 10-5 dilution ma104nsp5 c2s 2025-02-03(Coomassie Blue).tif]

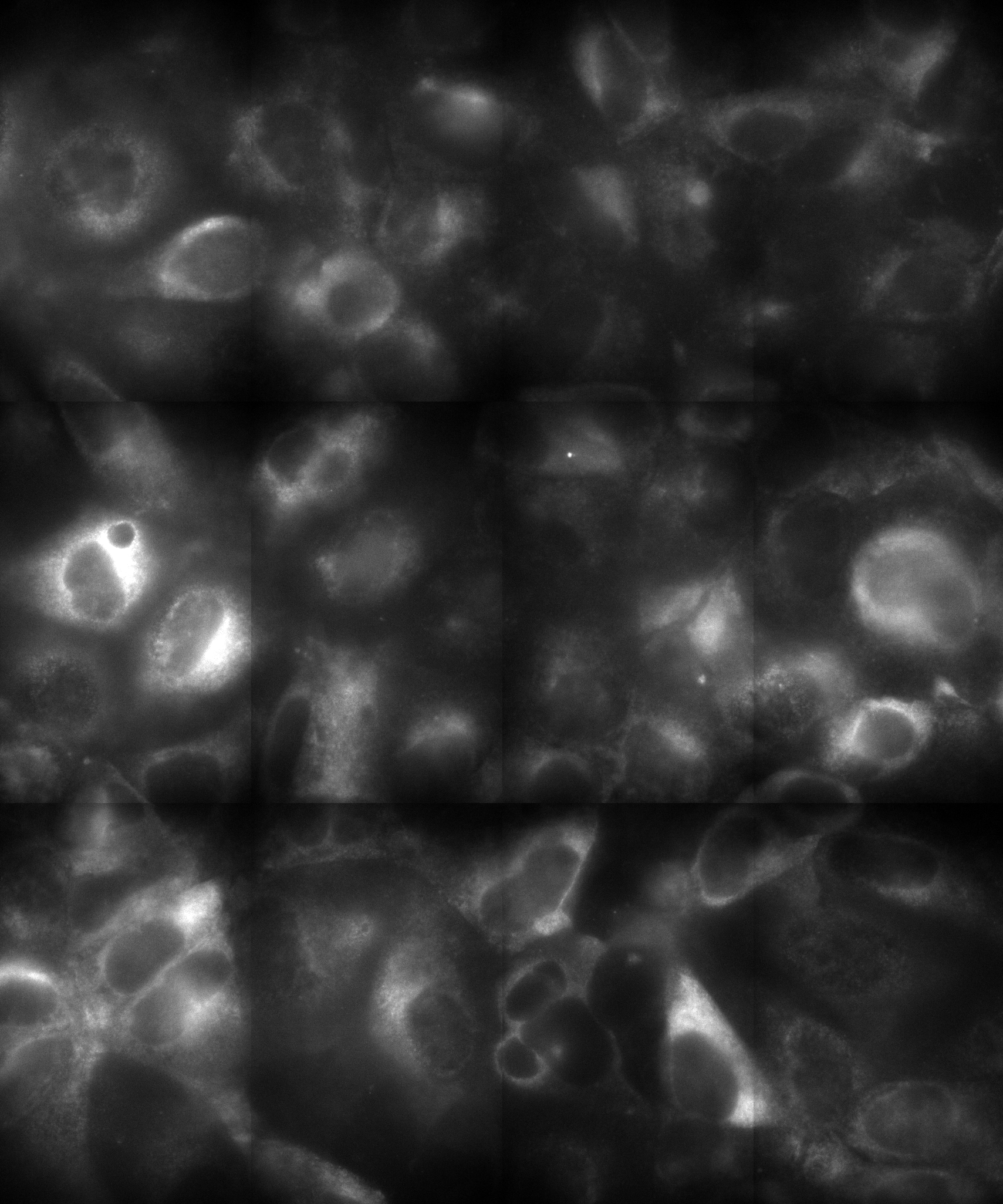

Supplement: Supplementary file 9 — Source data Fig. 7 [file 44318_2026_814_MOESM9_ESM.zip › Figure 8 raw/Figure 8C/SCLow-S67A-RV_1dpi_IF-antiNSP5-scan.tiff]

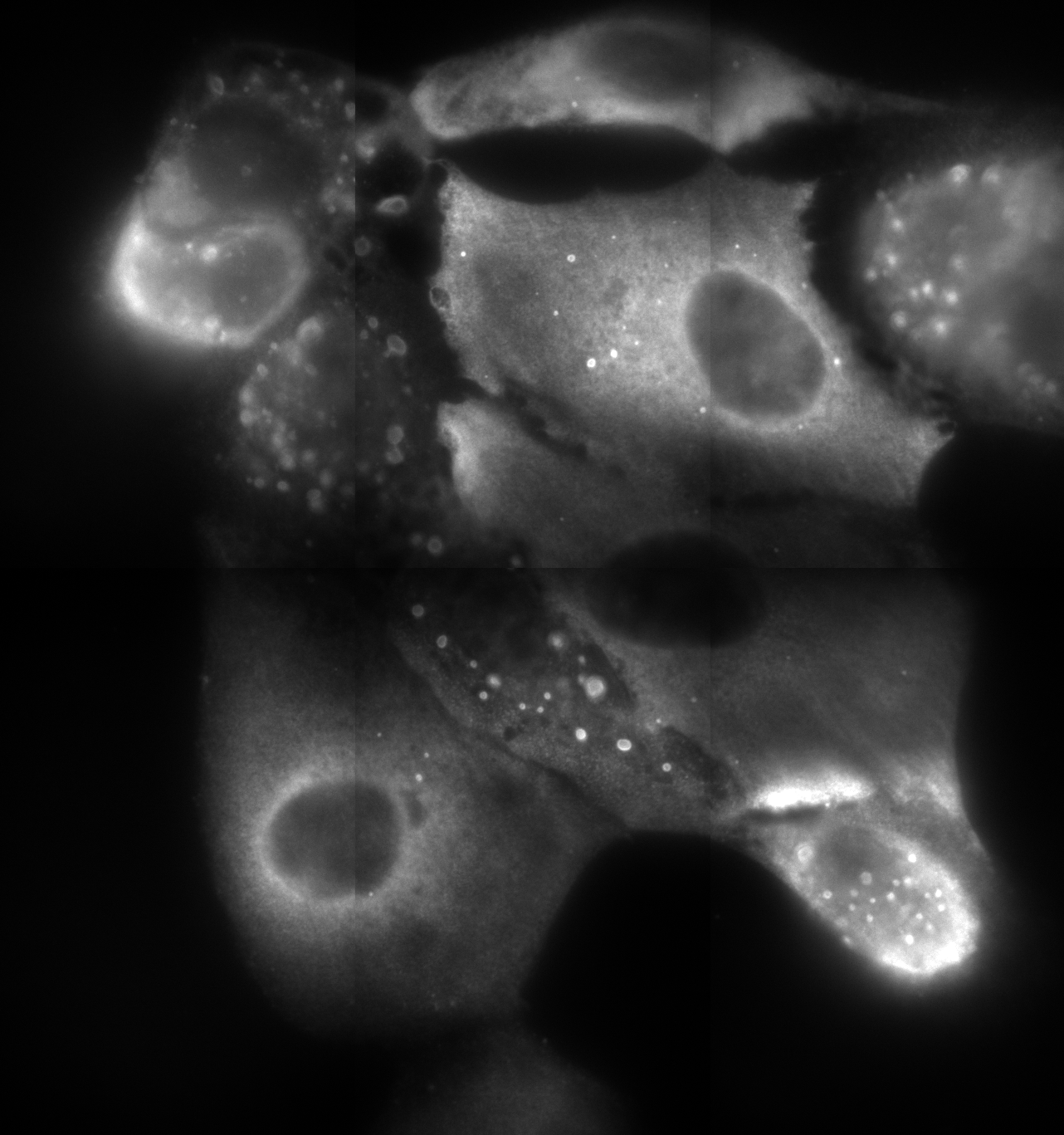

Supplement: Supplementary file 9 — Source data Fig. 7 [file 44318_2026_814_MOESM9_ESM.zip › Figure 8 raw/Figure 8C/SCLow-RV_1dpi_IF-antiNSP5-scan.tiff]

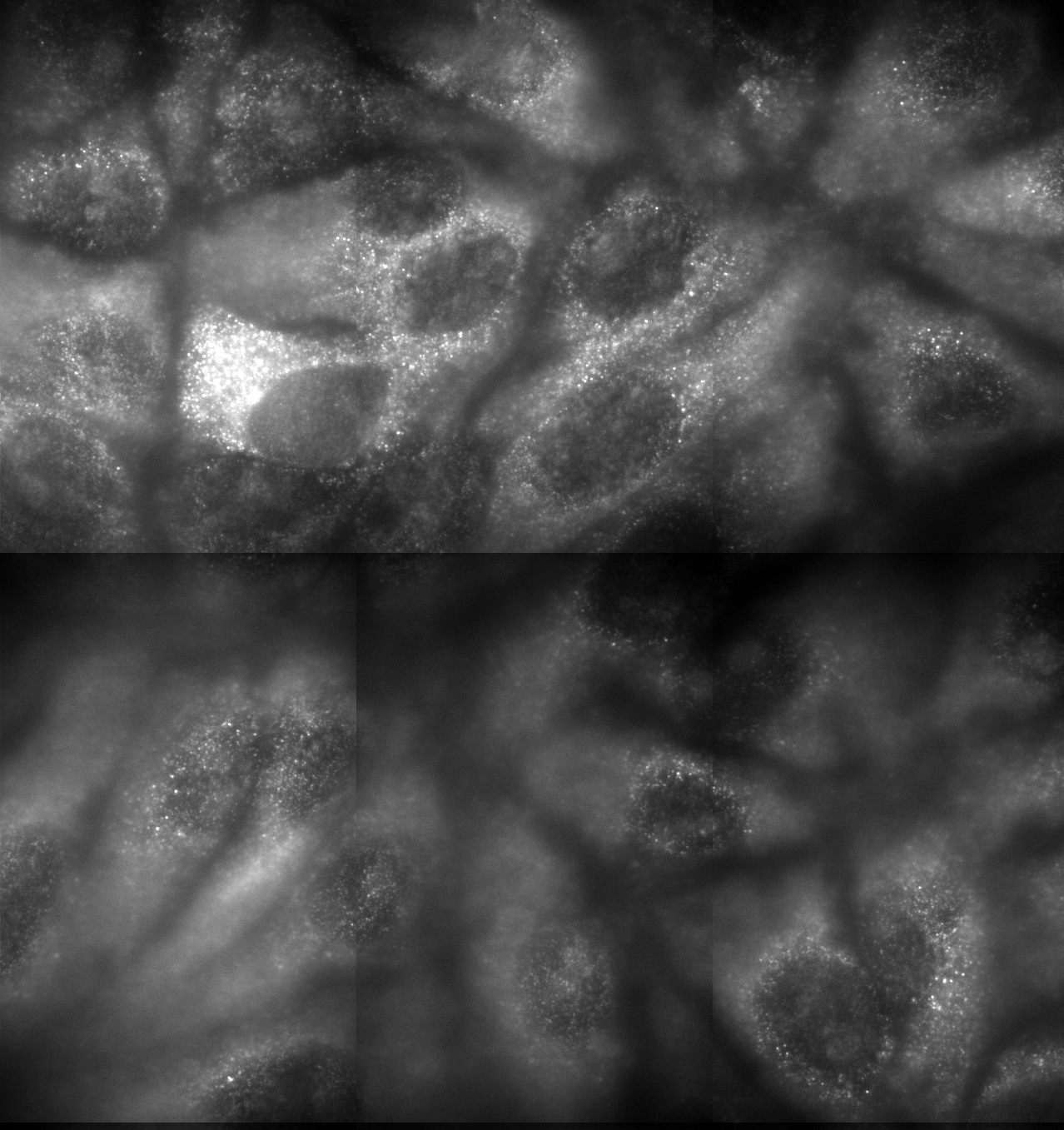

Supplement: Supplementary file 9 — Source data Fig. 7 [file 44318_2026_814_MOESM9_ESM.zip › Figure 8 raw/Figure 8C/uninfected-overview.tif]

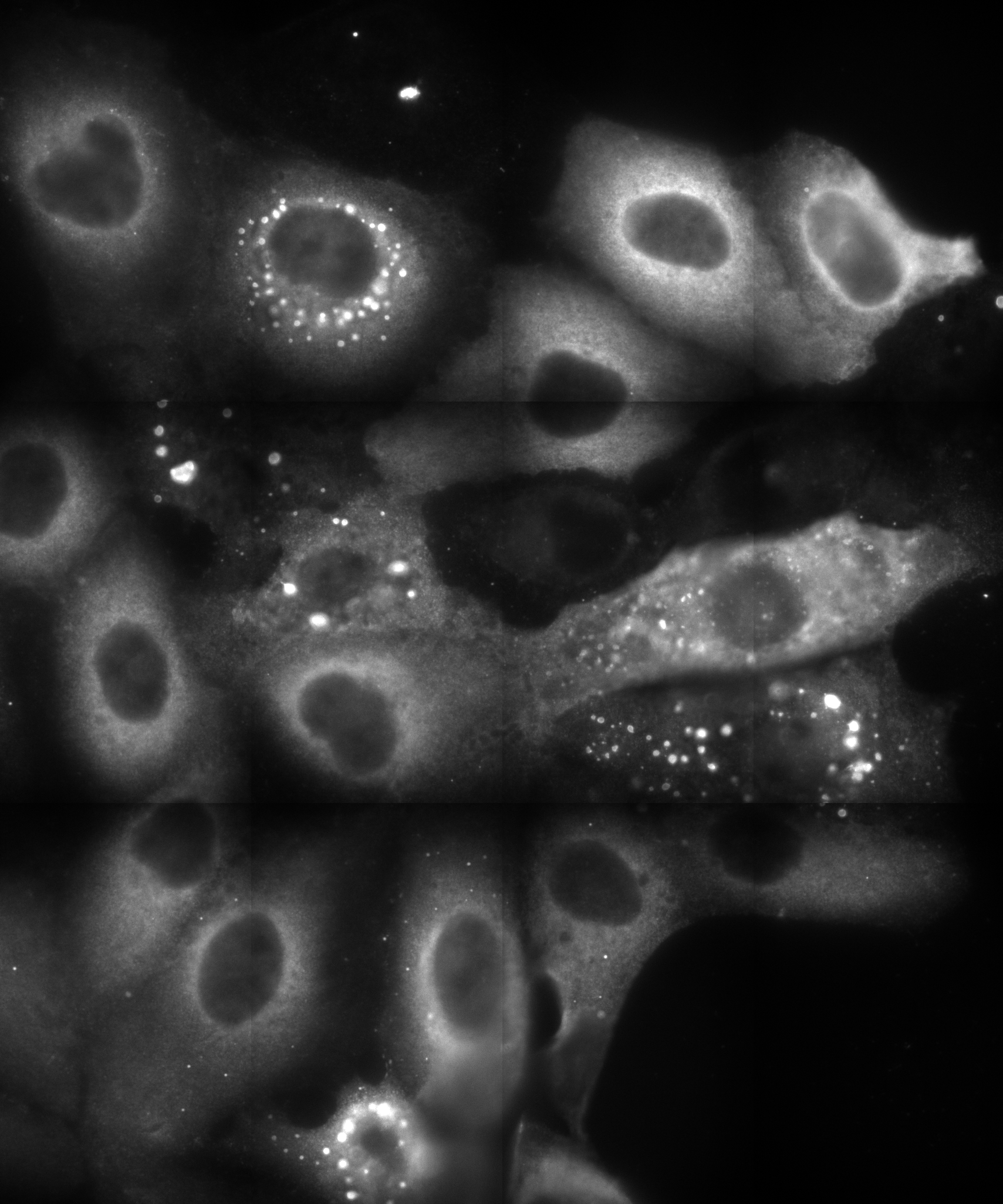

Supplement: Supplementary file 9 — Source data Fig. 7 [file 44318_2026_814_MOESM9_ESM.zip › Figure 8 raw/Figure 8C/2024_12_2_MA104-NSP5_SA11-S67D-RV_1dpi_IF-antiNSP5-scan4.tiff]

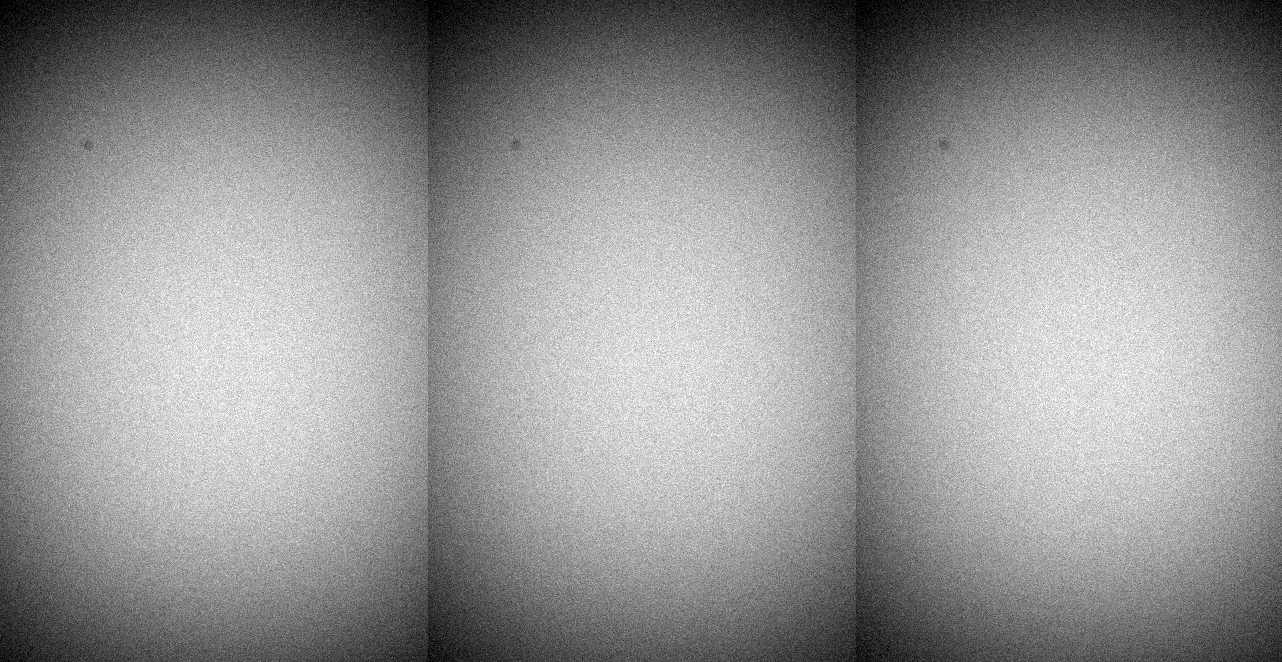

Supplement: Supplementary file 10 — Source data Fig. 8 [file 44318_2026_814_MOESM10_ESM.zip › Figure 9 A/NSP5 WT DeltaC/25uM-NSP2-RF-WT-A488_0uM-NSP5-RF-WT_50uM-NSP5-RF-DeltaC-10min-scan-1.tif]

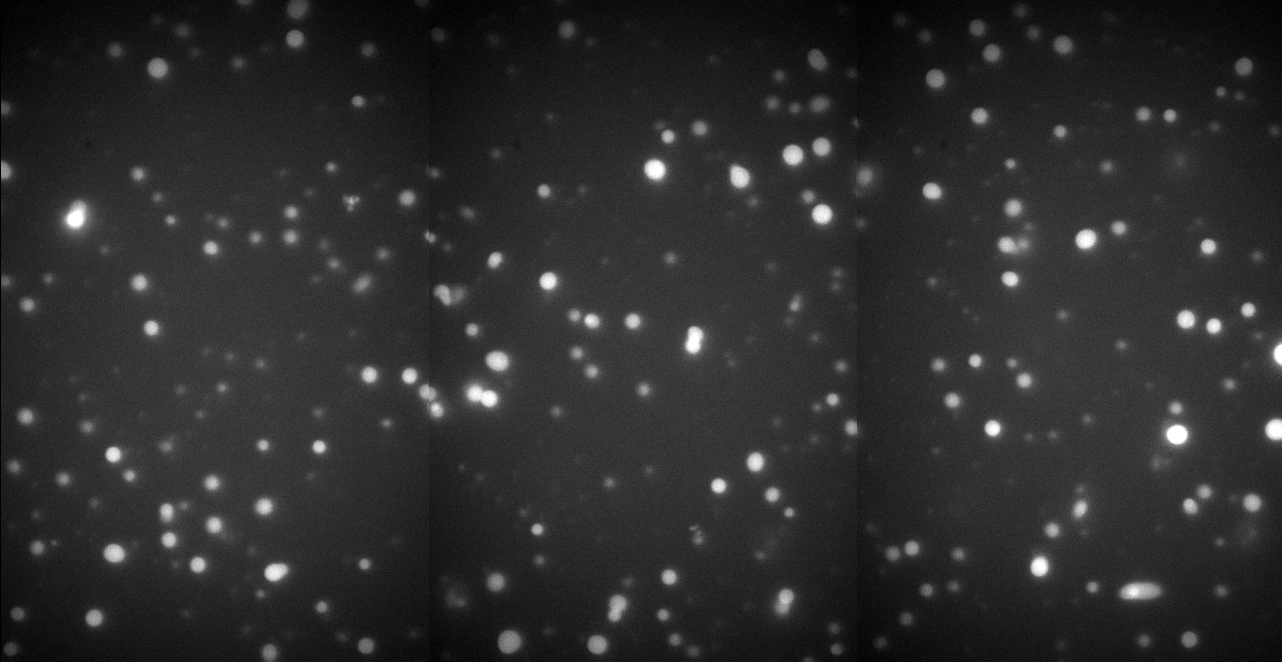

Supplement: Supplementary file 10 — Source data Fig. 8 [file 44318_2026_814_MOESM10_ESM.zip › Figure 9 A/NSP5 WT DeltaC/25uM-NSP2-RF-WT-A488_73-5uM-NSP5-RF-WT_12-5uM-NSP5-RF-DeltaC-10min-1.tif]

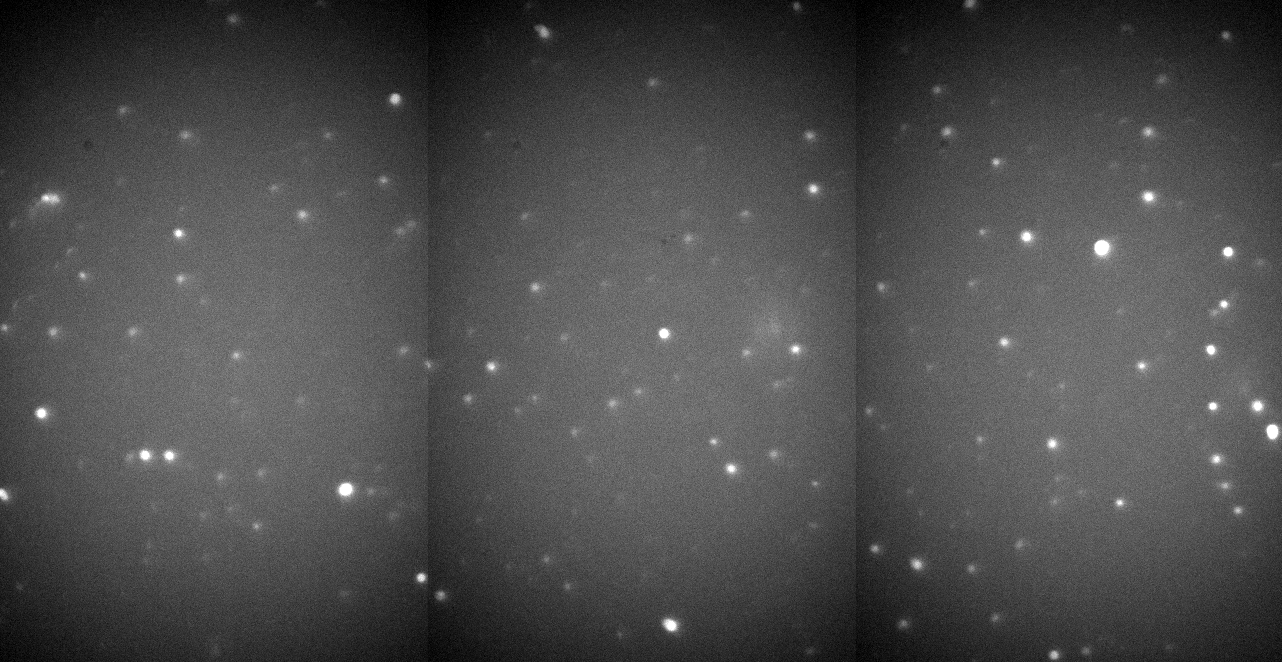

Supplement: Supplementary file 10 — Source data Fig. 8 [file 44318_2026_814_MOESM10_ESM.zip › Figure 9 A/NSP5 WT DeltaC/25uM-NSP2-RF-WT-A488_12-5uM-NSP5-RF-WT_37-5uM-NSP5-RF-DeltaC-10min-1.tif]

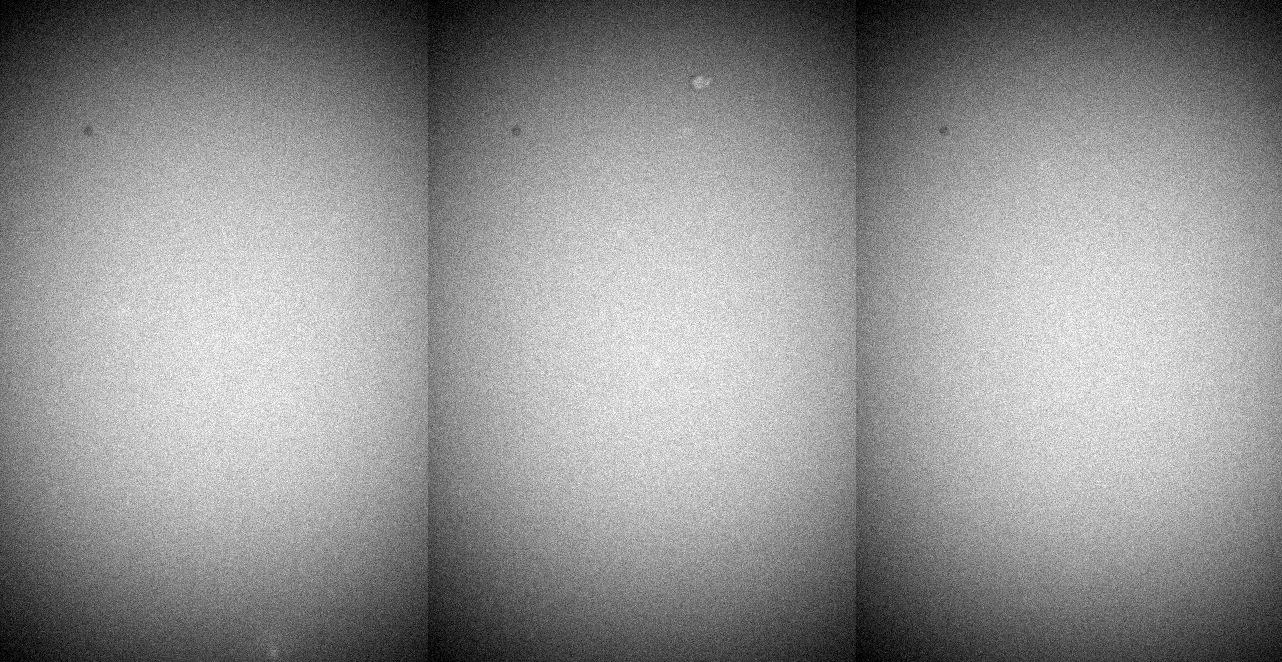

Supplement: Supplementary file 10 — Source data Fig. 8 [file 44318_2026_814_MOESM10_ESM.zip › Figure 9 A/NSP5 WT DeltaC/25uM-NSP2-RF-WT-A488_0uM-NSP5-RF-WT_50uM-NSP5-RF-DeltaC-10min-scan-2.tif]

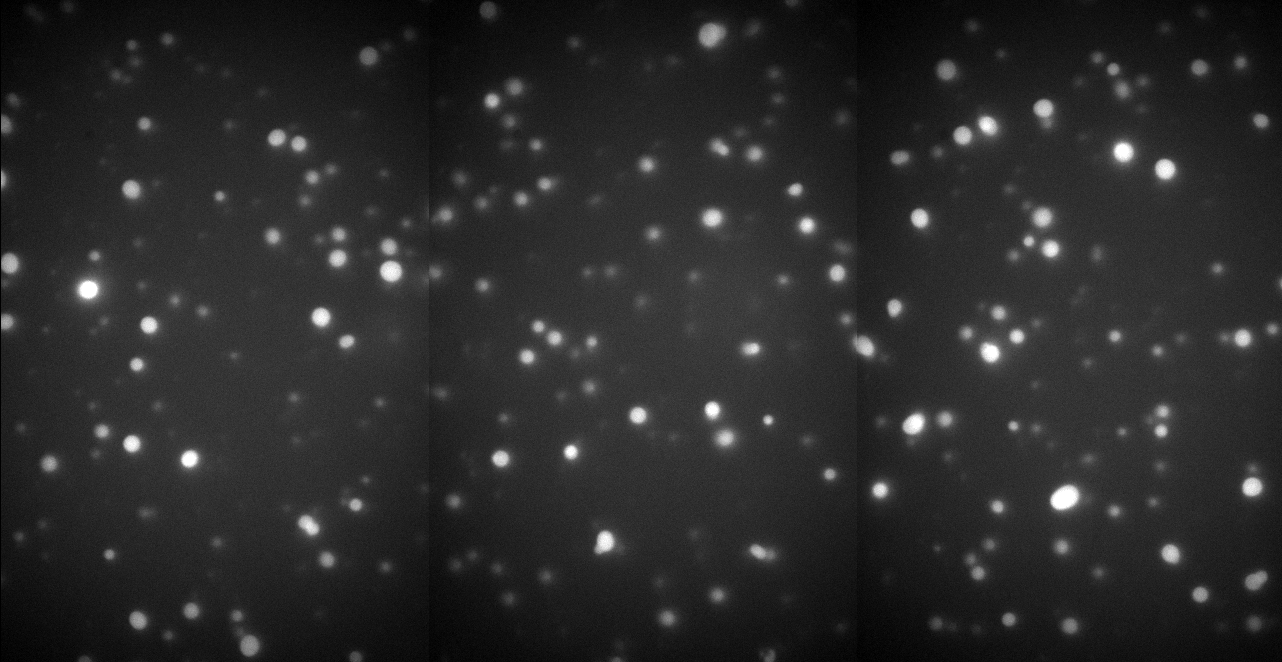

Supplement: Supplementary file 10 — Source data Fig. 8 [file 44318_2026_814_MOESM10_ESM.zip › Figure 9 A/NSP5 WT DeltaC/25uM-NSP2-RF-WT-A488_73-5uM-NSP5-RF-WT_12-5uM-NSP5-RF-DeltaC-10min-2.tif]

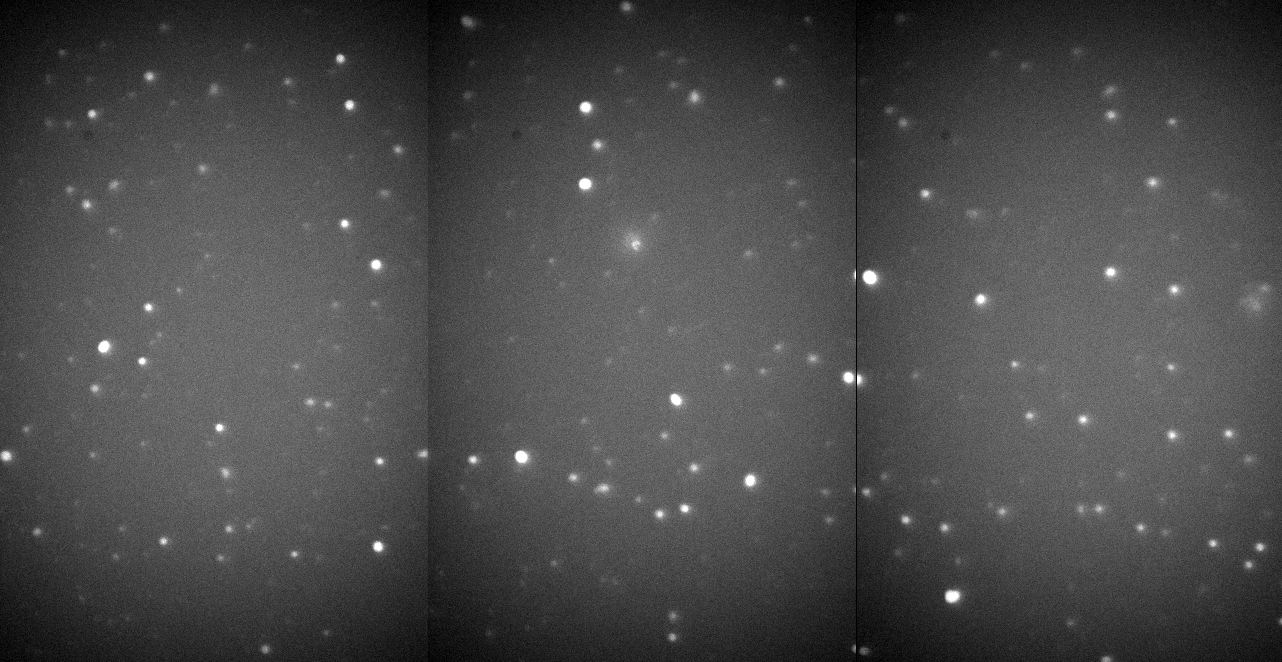

Supplement: Supplementary file 10 — Source data Fig. 8 [file 44318_2026_814_MOESM10_ESM.zip › Figure 9 A/NSP5 WT DeltaC/25uM-NSP2-RF-WT-A488_12-5uM-NSP5-RF-WT_37-5uM-NSP5-RF-DeltaC-10min-2.tif]

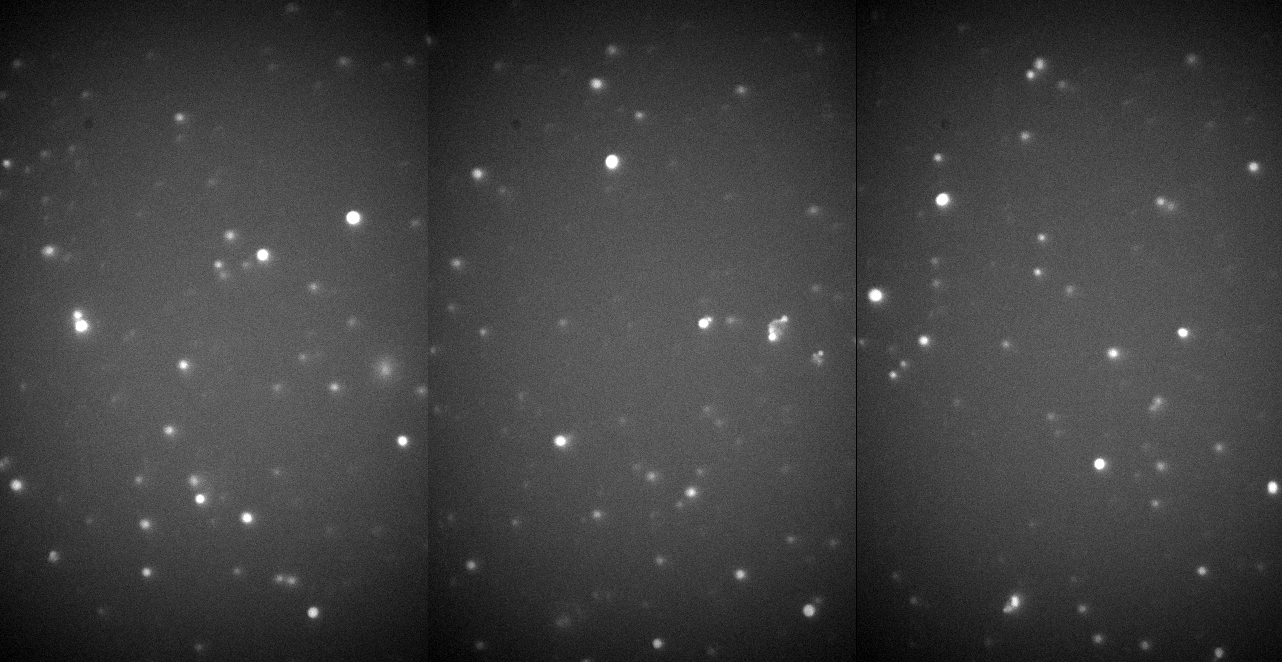

Supplement: Supplementary file 10 — Source data Fig. 8 [file 44318_2026_814_MOESM10_ESM.zip › Figure 9 A/NSP5 WT DeltaC/25uM-NSP2-RF-WT-A488_12-5uM-NSP5-RF-WT_37-5uM-NSP5-RF-DeltaC-10min-3.tif]

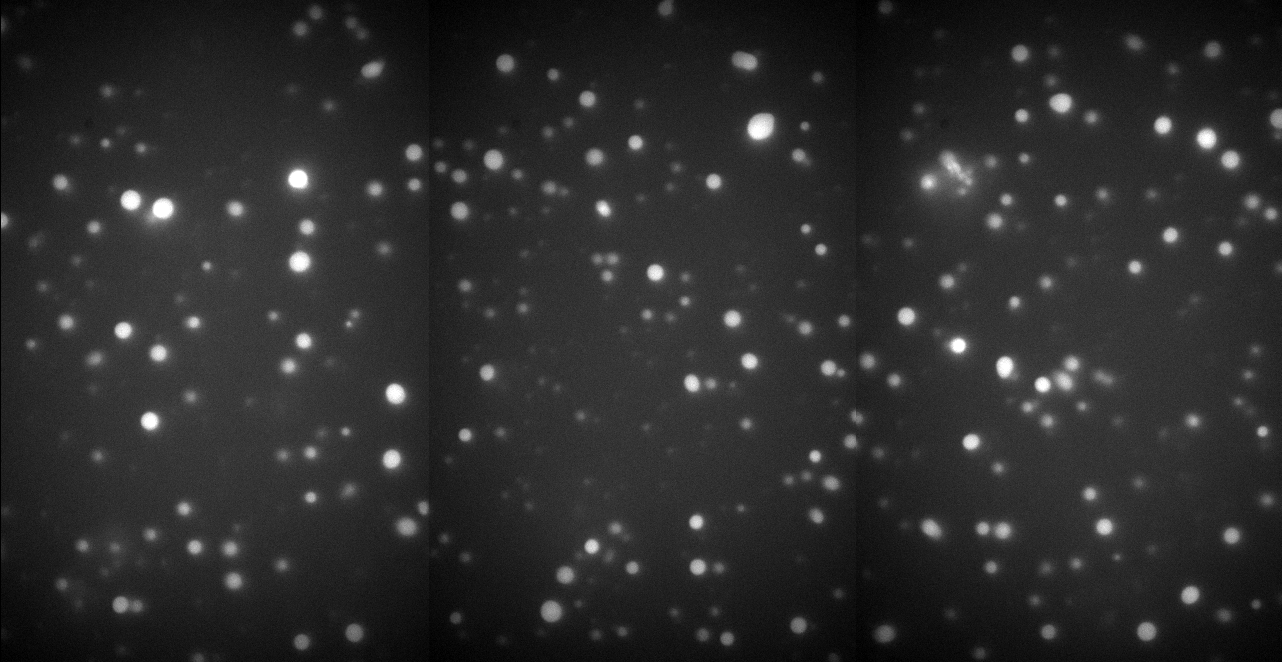

Supplement: Supplementary file 10 — Source data Fig. 8 [file 44318_2026_814_MOESM10_ESM.zip › Figure 9 A/NSP5 WT DeltaC/25uM-NSP2-RF-WT-A488_73-5uM-NSP5-RF-WT_12-5uM-NSP5-RF-DeltaC-10min-3.tif]

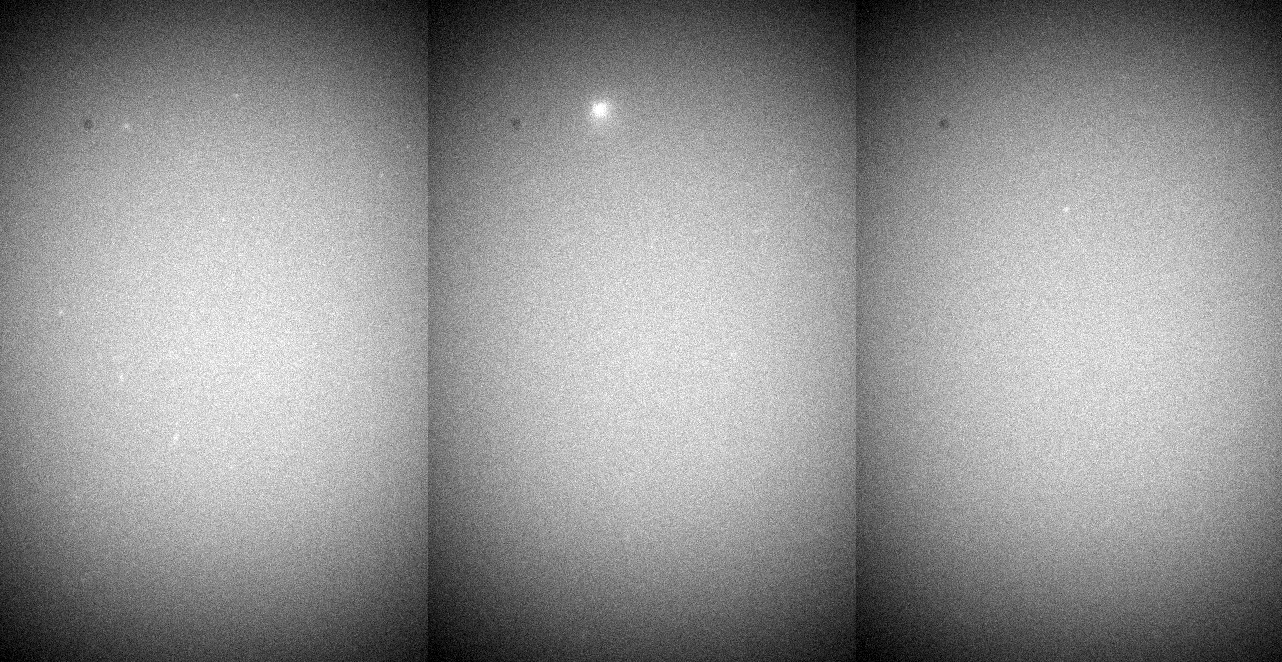

Supplement: Supplementary file 10 — Source data Fig. 8 [file 44318_2026_814_MOESM10_ESM.zip › Figure 9 A/NSP5 WT DeltaC/25uM-NSP2-RF-WT-A488_0uM-NSP5-RF-WT_50uM-NSP5-RF-DeltaC-10min-scan-3.tif]

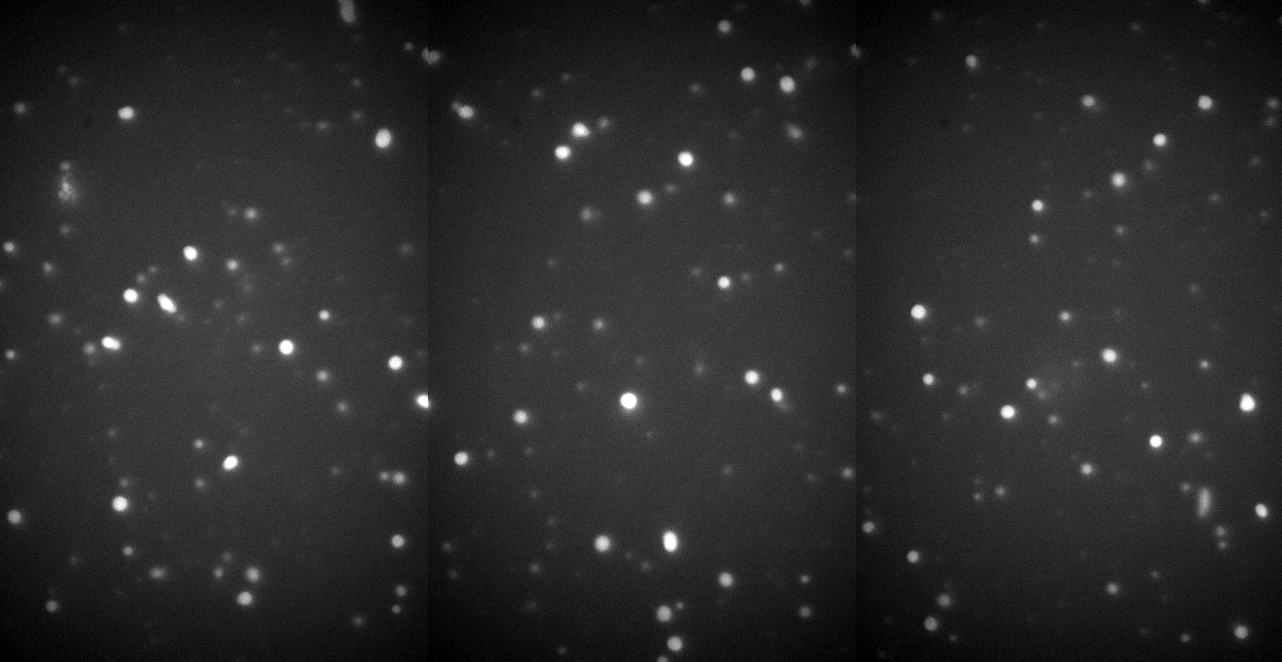

Supplement: Supplementary file 10 — Source data Fig. 8 [file 44318_2026_814_MOESM10_ESM.zip › Figure 9 A/NSP5 WT DeltaC/25uM-NSP2-RF-WT-A488_25uM-NSP5-RF-WT_25uM-NSP5-RF-DeltaC-10min-3.tif]

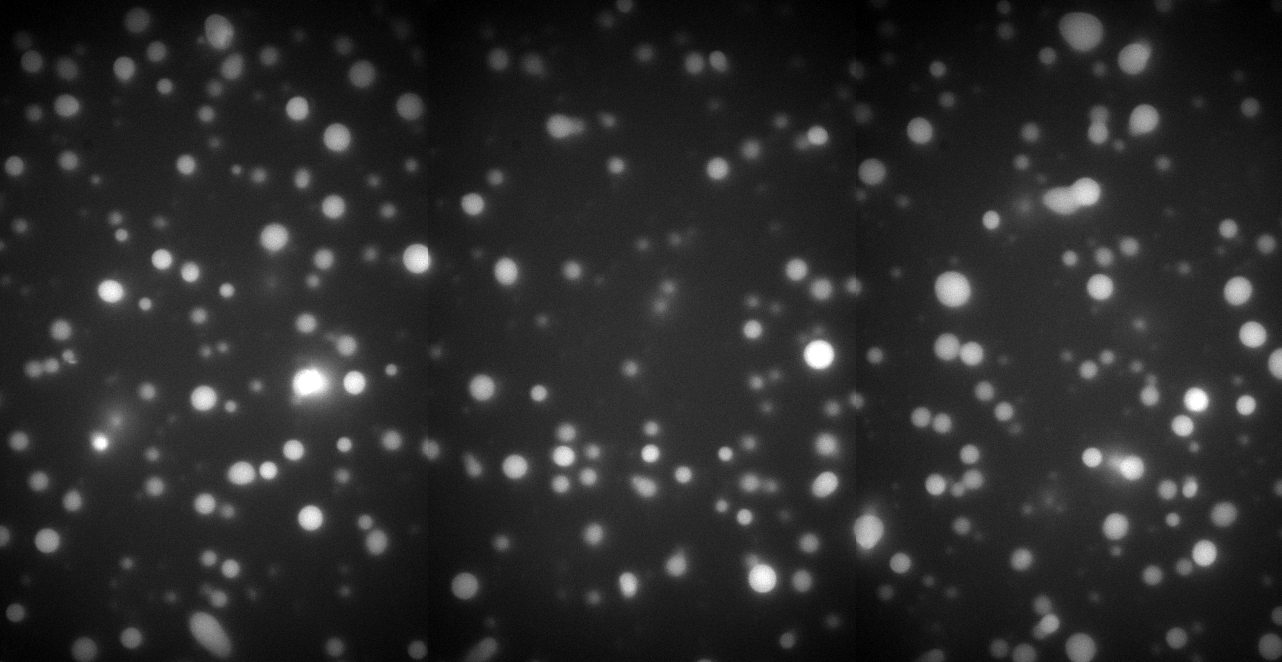

Supplement: Supplementary file 10 — Source data Fig. 8 [file 44318_2026_814_MOESM10_ESM.zip › Figure 9 A/NSP5 WT DeltaC/25uM-NSP2-RF-WT-A488_50uM-NSP5-RF-WT_0uM-NSP5-RF-DeltaC-10min-1.tif]

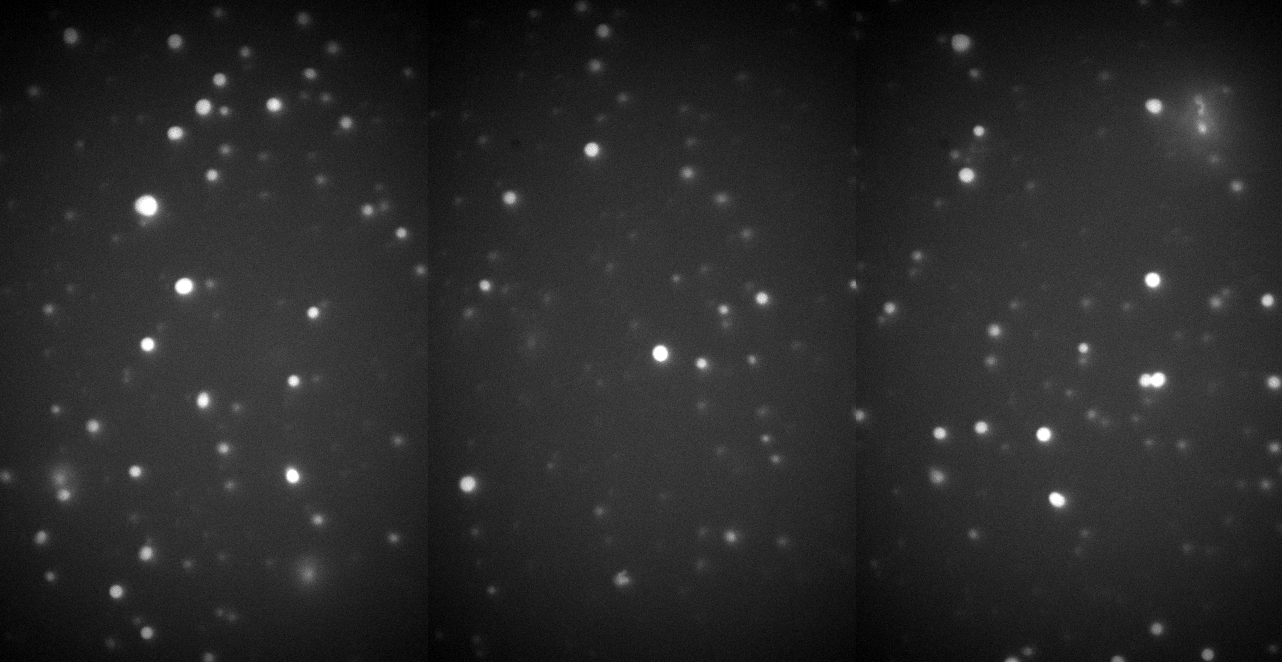

Supplement: Supplementary file 10 — Source data Fig. 8 [file 44318_2026_814_MOESM10_ESM.zip › Figure 9 A/NSP5 WT DeltaC/25uM-NSP2-RF-WT-A488_25uM-NSP5-RF-WT_25uM-NSP5-RF-DeltaC-10min-2.tif]

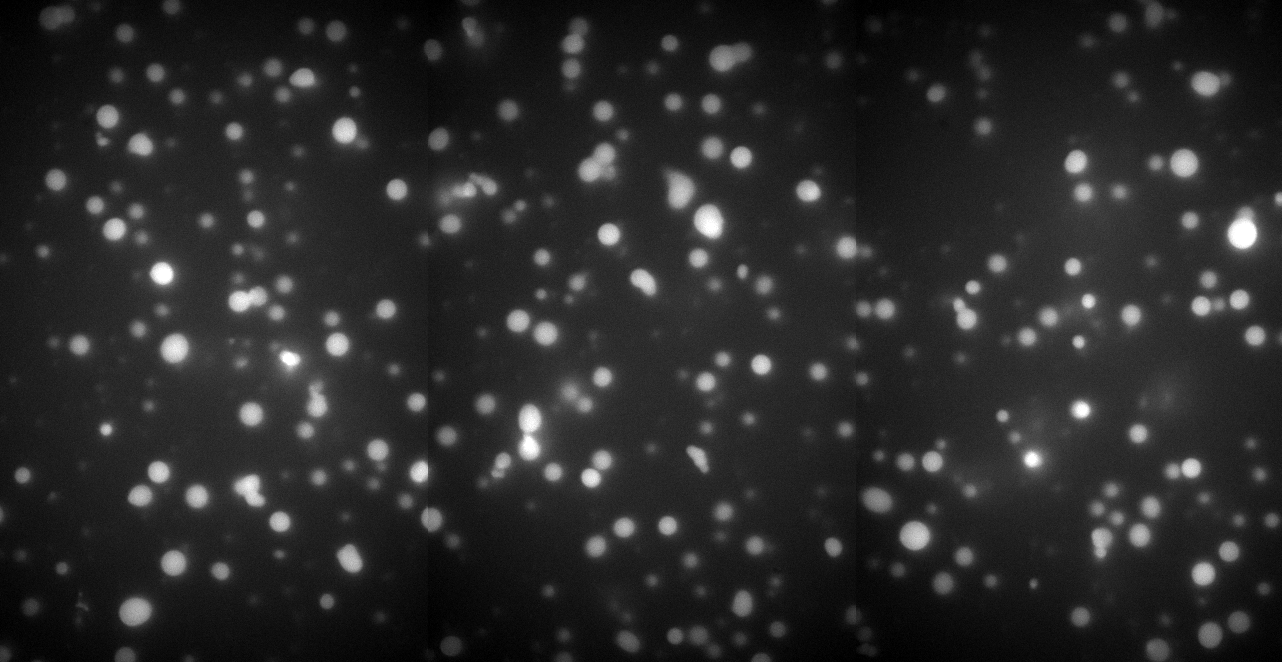

Supplement: Supplementary file 10 — Source data Fig. 8 [file 44318_2026_814_MOESM10_ESM.zip › Figure 9 A/NSP5 WT DeltaC/25uM-NSP2-RF-WT-A488_50uM-NSP5-RF-WT_0uM-NSP5-RF-DeltaC-10min-3.tif]

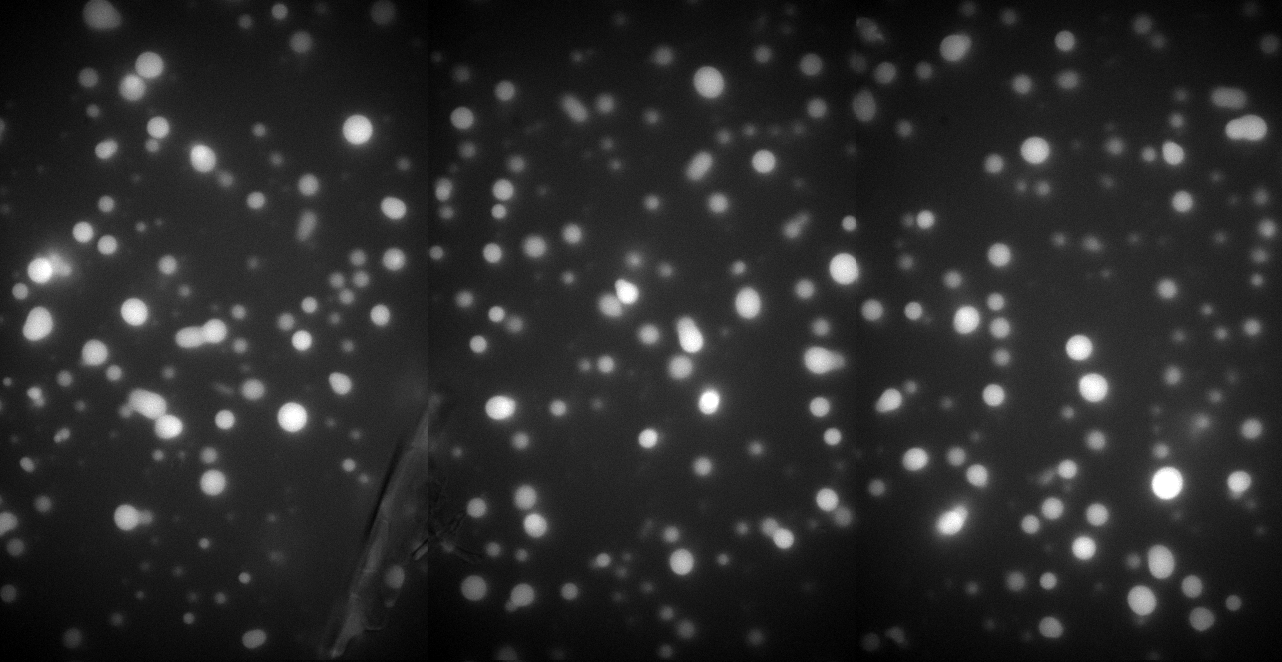

Supplement: Supplementary file 10 — Source data Fig. 8 [file 44318_2026_814_MOESM10_ESM.zip › Figure 9 A/NSP5 WT DeltaC/25uM-NSP2-RF-WT-A488_50uM-NSP5-RF-WT_0uM-NSP5-RF-DeltaC-10min-2.tif]

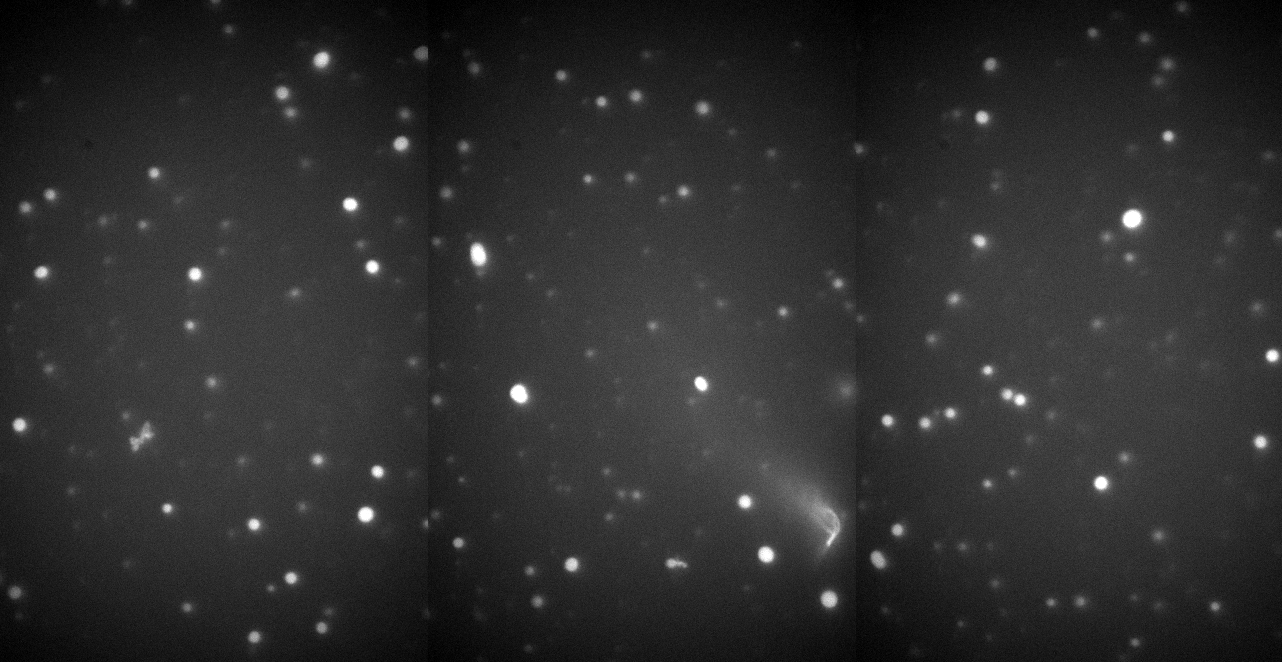

Supplement: Supplementary file 10 — Source data Fig. 8 [file 44318_2026_814_MOESM10_ESM.zip › Figure 9 A/NSP5 WT DeltaC/25uM-NSP2-RF-WT-A488_25uM-NSP5-RF-WT_25uM-NSP5-RF-DeltaC-10min-1.tif]

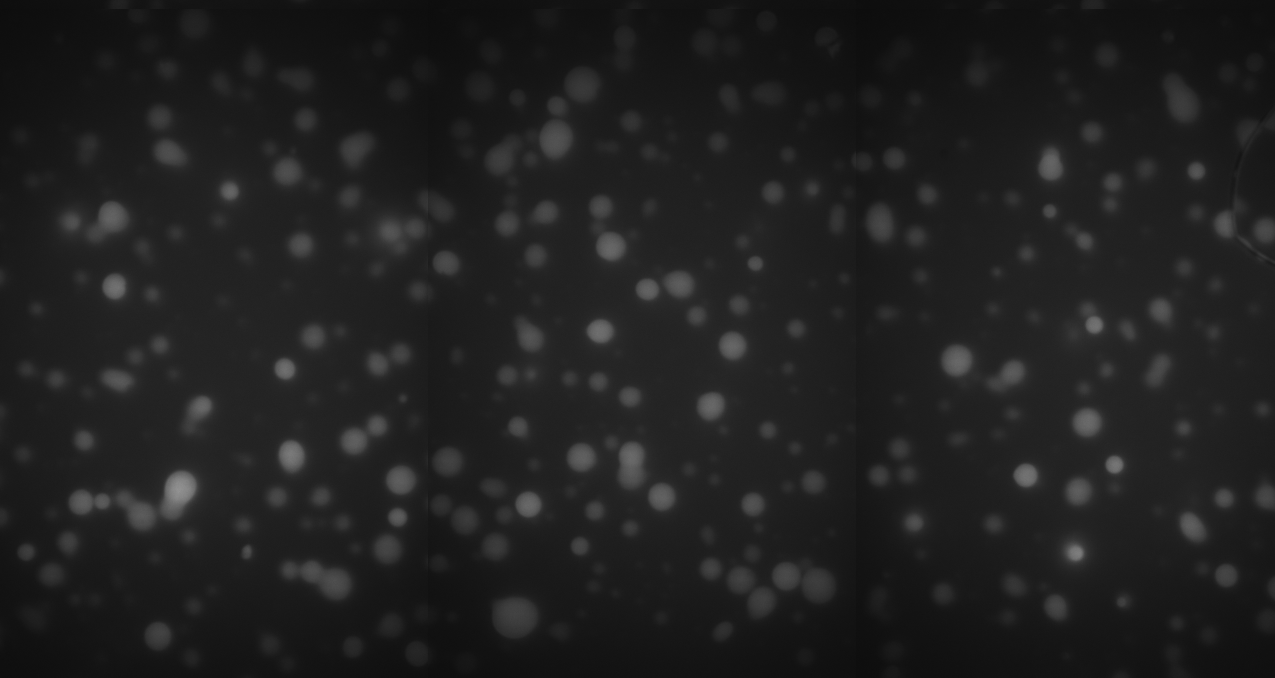

Supplement: Supplementary file 10 — Source data Fig. 8 [file 44318_2026_814_MOESM10_ESM.zip › Figure 9 A/NSP5 HP CTD/2024_10_01_25uM-NSP2-A488_25uM-NSP5-RF-HP_10min-scan1-2.tif]

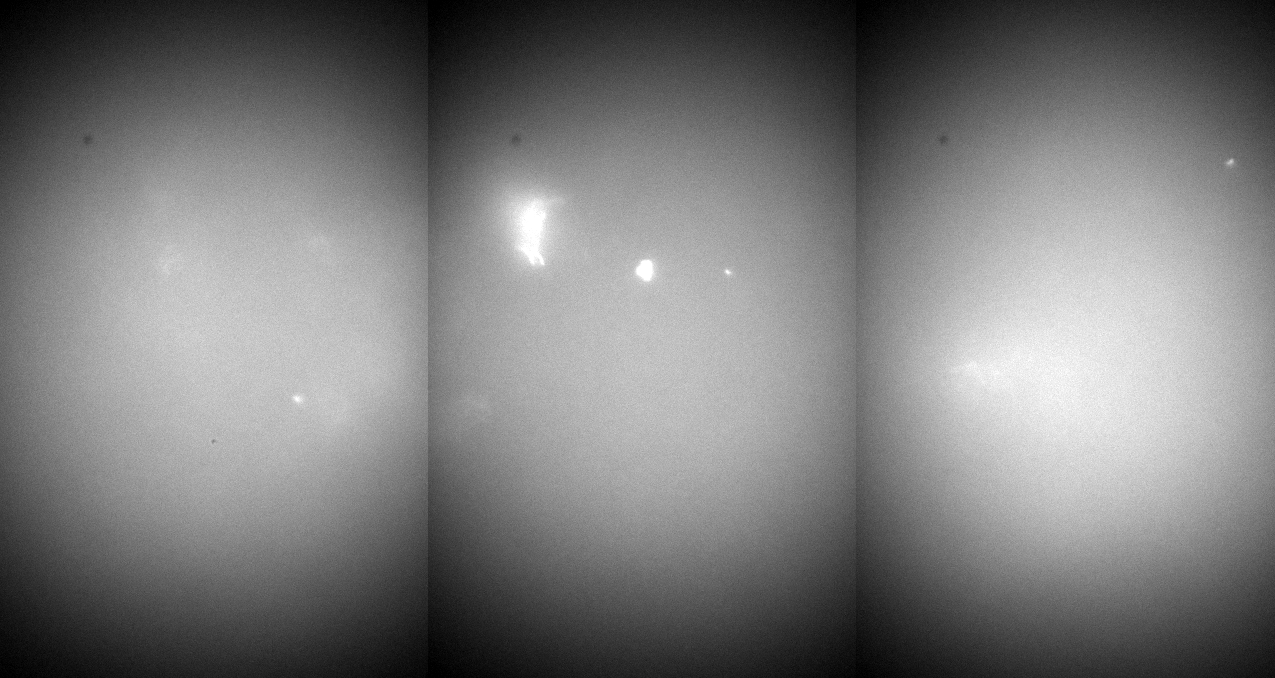

Supplement: Supplementary file 10 — Source data Fig. 8 [file 44318_2026_814_MOESM10_ESM.zip › Figure 9 A/NSP5 HP CTD/2024_10_01_25uM-NSP2-A488_25uM-NSP5-RF-CTD_10min-scan-2.tif]

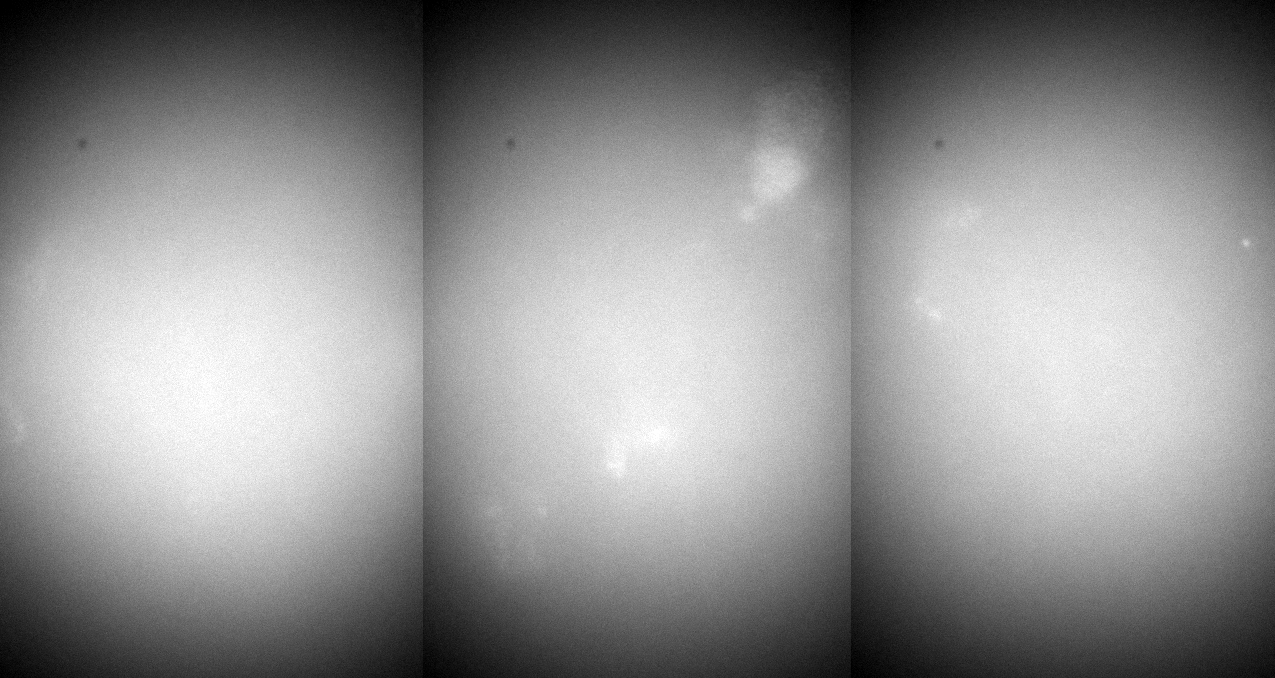

Supplement: Supplementary file 10 — Source data Fig. 8 [file 44318_2026_814_MOESM10_ESM.zip › Figure 9 A/NSP5 HP CTD/2024_10_01_25uM-NSP2-A488_25uM-NSP5-RF-CTD_10min-scan-3.tif]

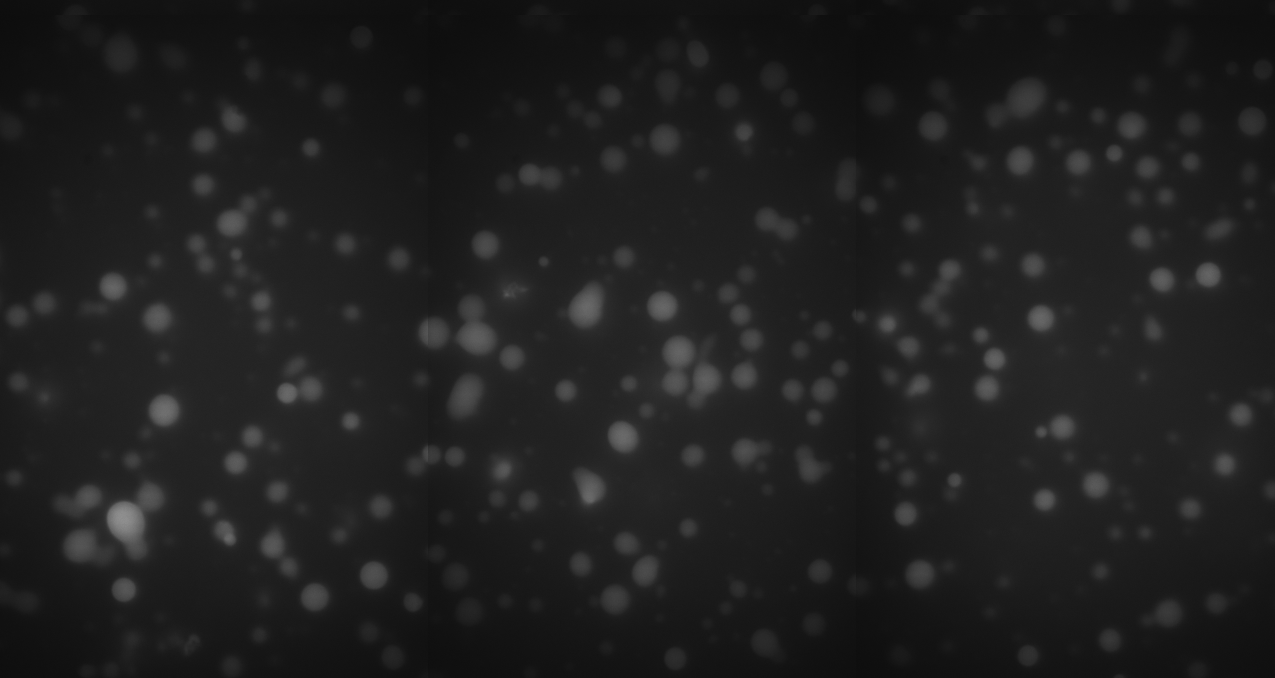

Supplement: Supplementary file 10 — Source data Fig. 8 [file 44318_2026_814_MOESM10_ESM.zip › Figure 9 A/NSP5 HP CTD/2024_10_01_25uM-NSP2-A488_25uM-NSP5-RF-HP_10min-scan1-3.tif]

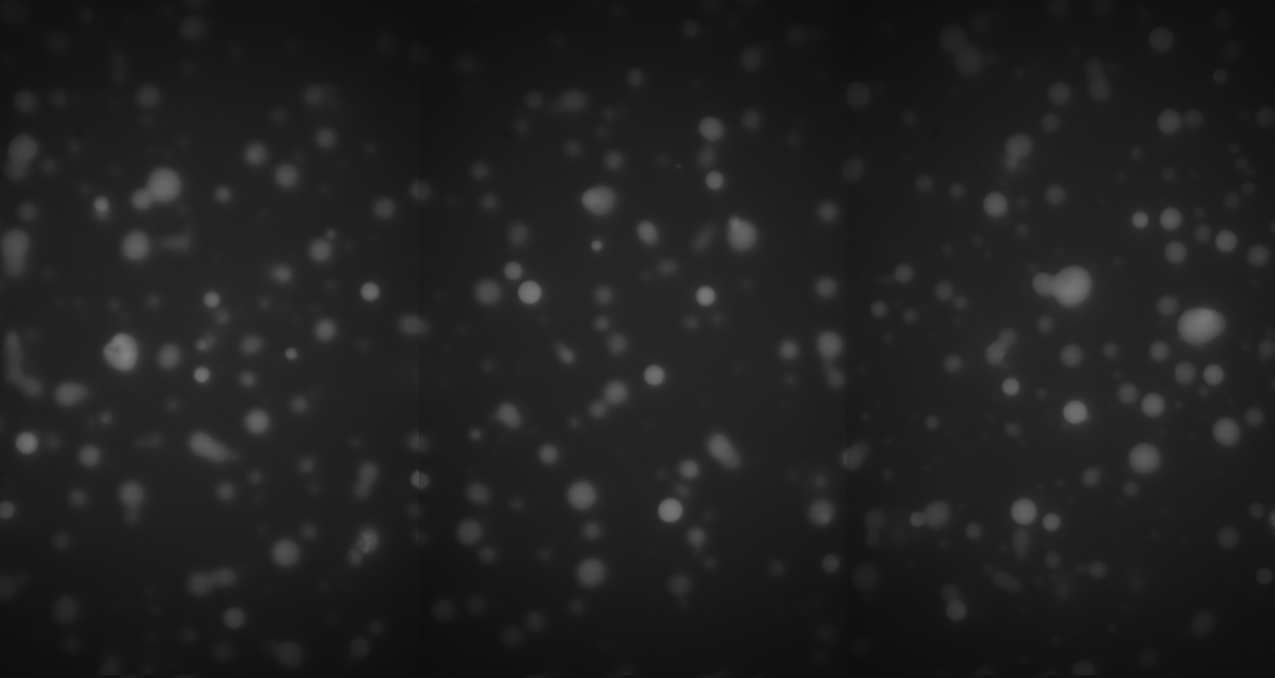

Supplement: Supplementary file 10 — Source data Fig. 8 [file 44318_2026_814_MOESM10_ESM.zip › Figure 9 A/NSP5 HP CTD/2024_10_01_25uM-NSP2-A488_25uM-NSP5-RF-HP_10min-scan1-1.tif]

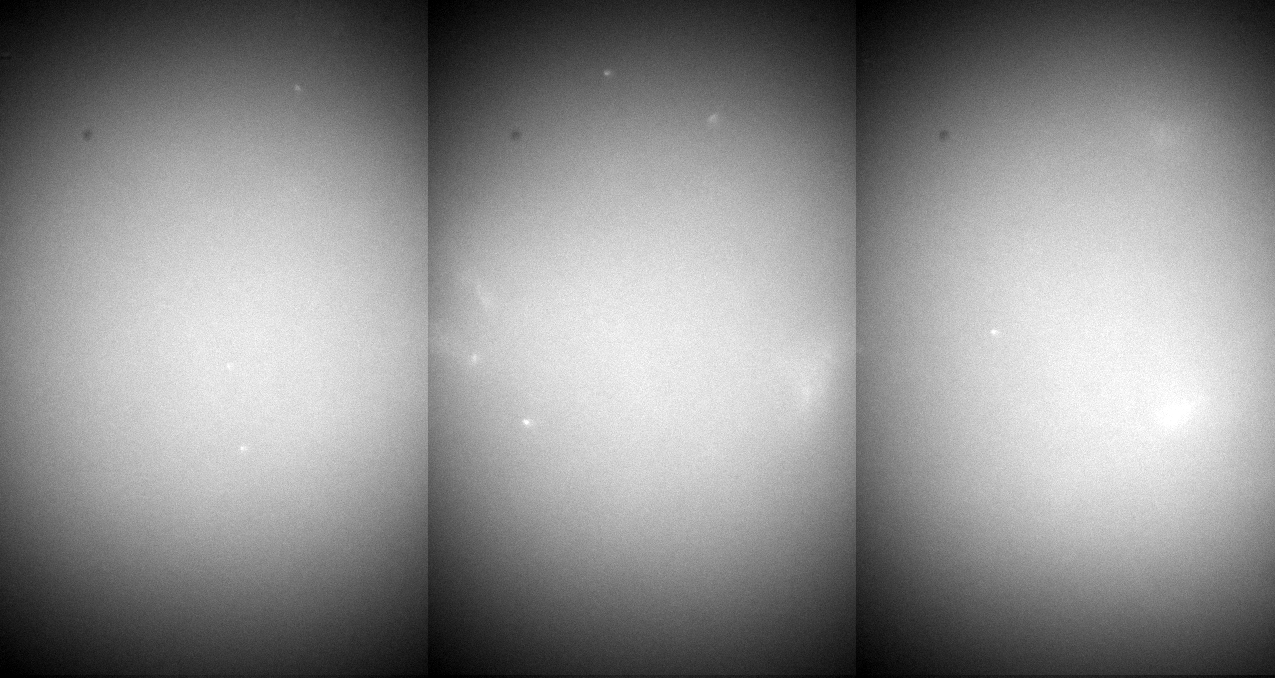

Supplement: Supplementary file 10 — Source data Fig. 8 [file 44318_2026_814_MOESM10_ESM.zip › Figure 9 A/NSP5 HP CTD/2024_10_01_25uM-NSP2-A488_25uM-NSP5-RF-CTD_10min-scan-1.tif]

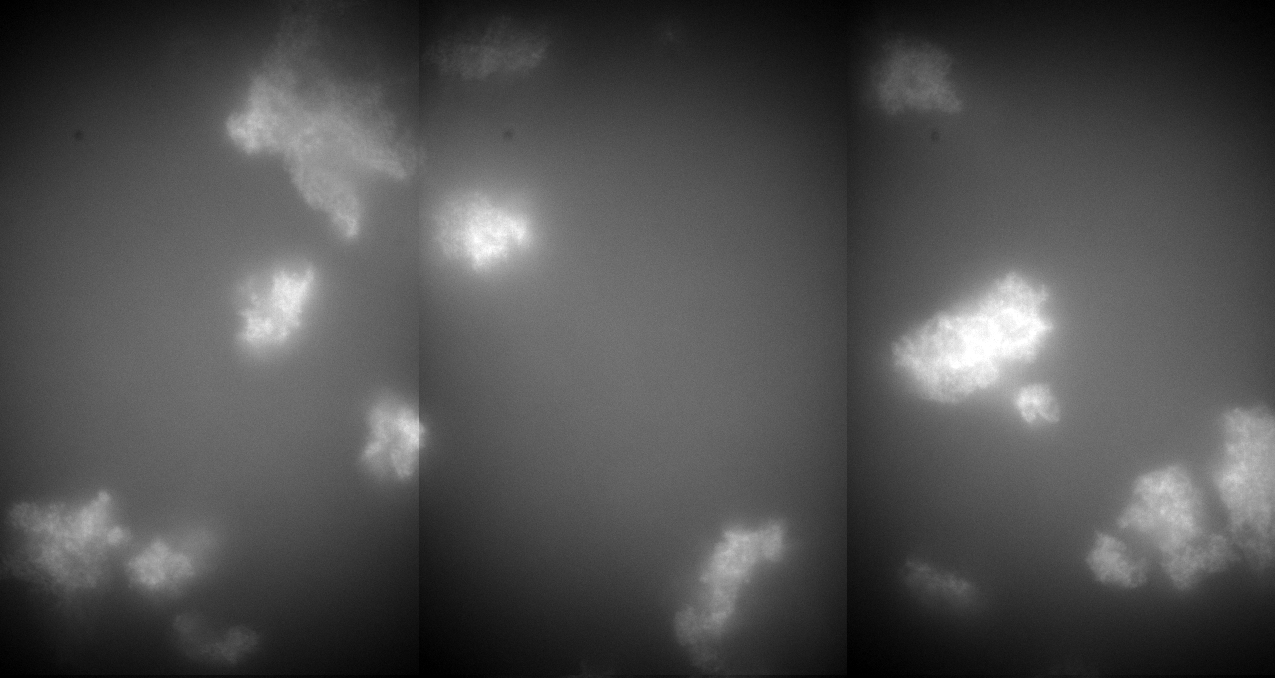

Supplement: Supplementary file 10 — Source data Fig. 8 [file 44318_2026_814_MOESM10_ESM.zip › Figure 9 A/NSP5 HP CTD/2024_10_01_25uM-NSP2-A488_6uM-NSP5-RF-HP_18uM-NSP5-RF-CTD_10min-scan-1.tif]

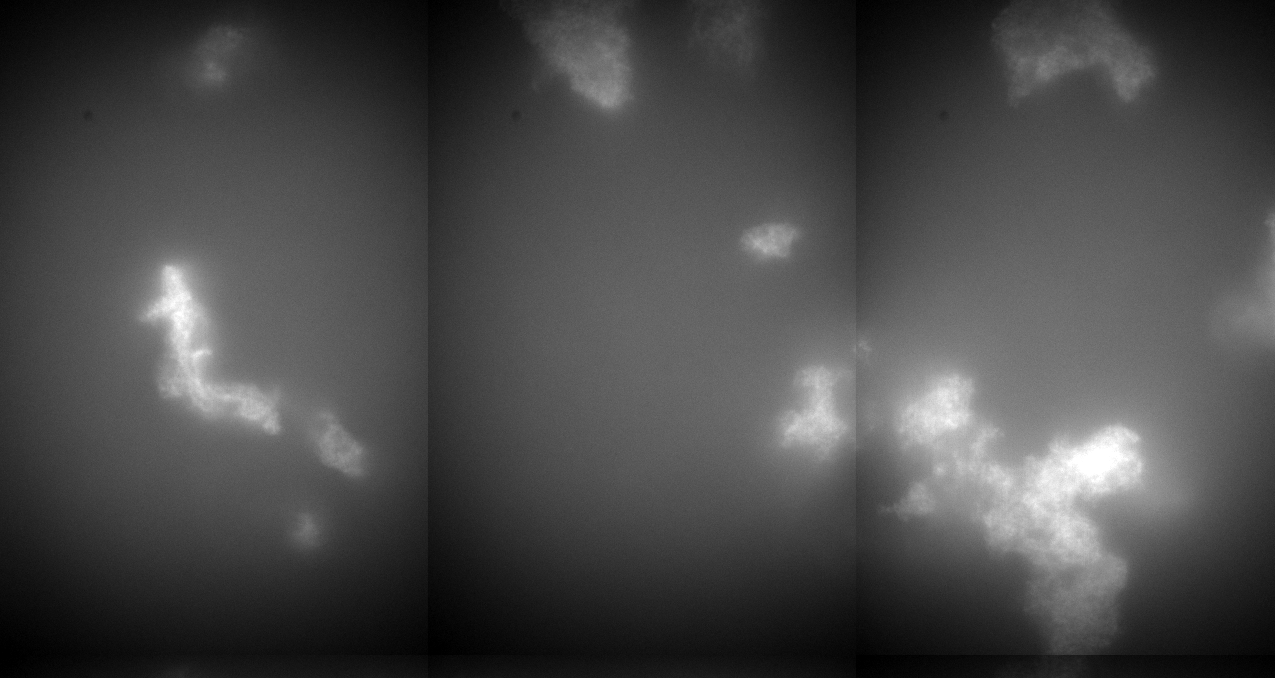

Supplement: Supplementary file 10 — Source data Fig. 8 [file 44318_2026_814_MOESM10_ESM.zip › Figure 9 A/NSP5 HP CTD/2024_10_01_25uM-NSP2-A488_6uM-NSP5-RF-HP_18uM-NSP5-RF-CTD_10min-scan-2.tif]

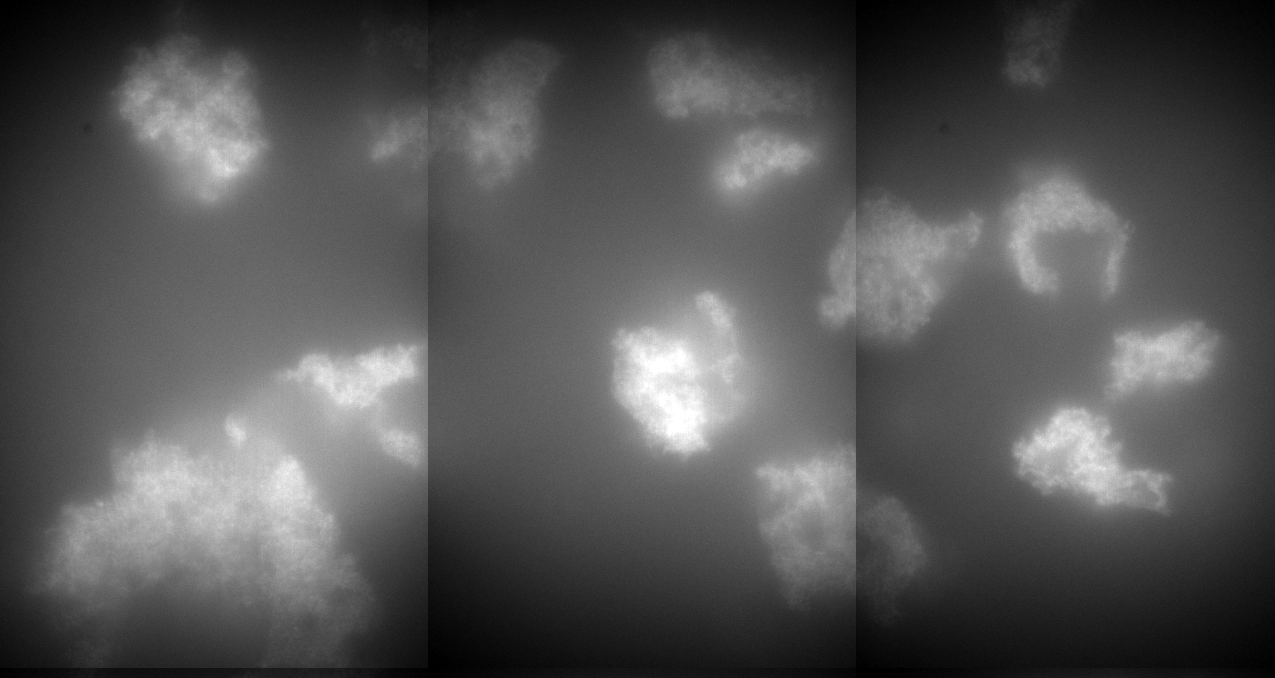

Supplement: Supplementary file 10 — Source data Fig. 8 [file 44318_2026_814_MOESM10_ESM.zip › Figure 9 A/NSP5 HP CTD/2024_10_01_25uM-NSP2-A488_6uM-NSP5-RF-HP_18uM-NSP5-RF-CTD_10min-scan-3.tif]

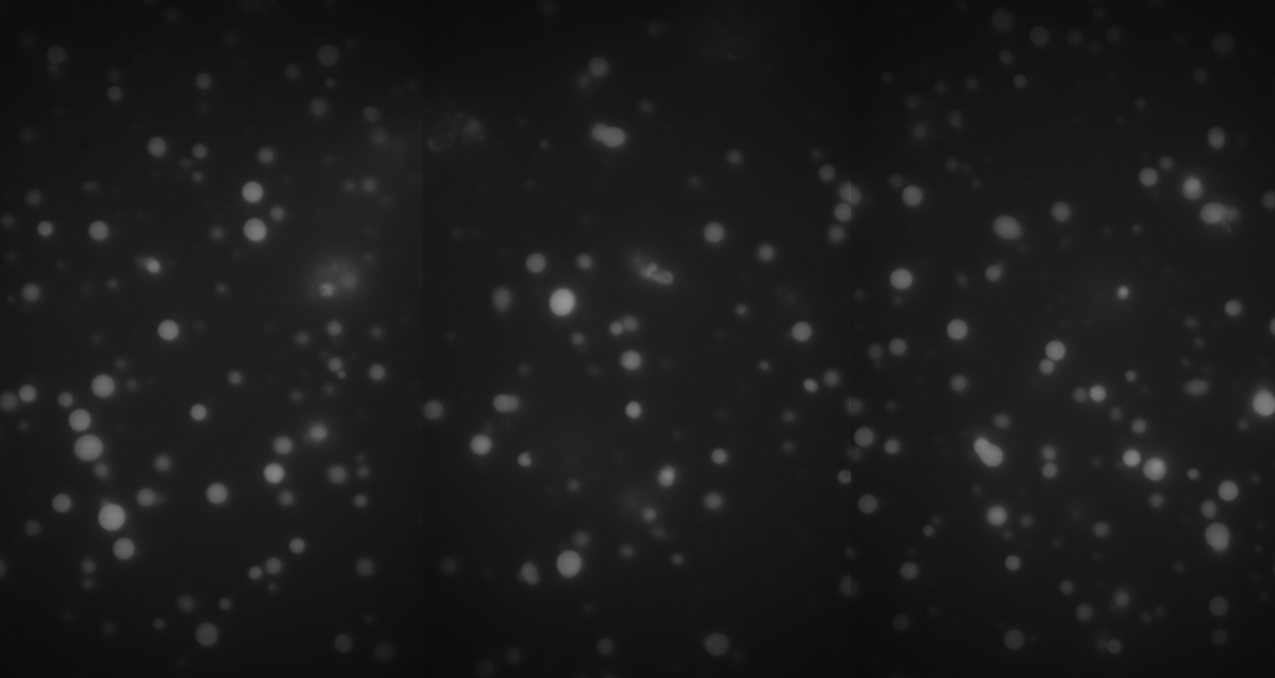

Supplement: Supplementary file 10 — Source data Fig. 8 [file 44318_2026_814_MOESM10_ESM.zip › Figure 9 A/NSP5 HP CTD/2024_10_01_25uM-NSP2-A488_18uM-NSP5-RF-HP_6uM-NSP5-RF-CTD_10min-scan-1.tif]

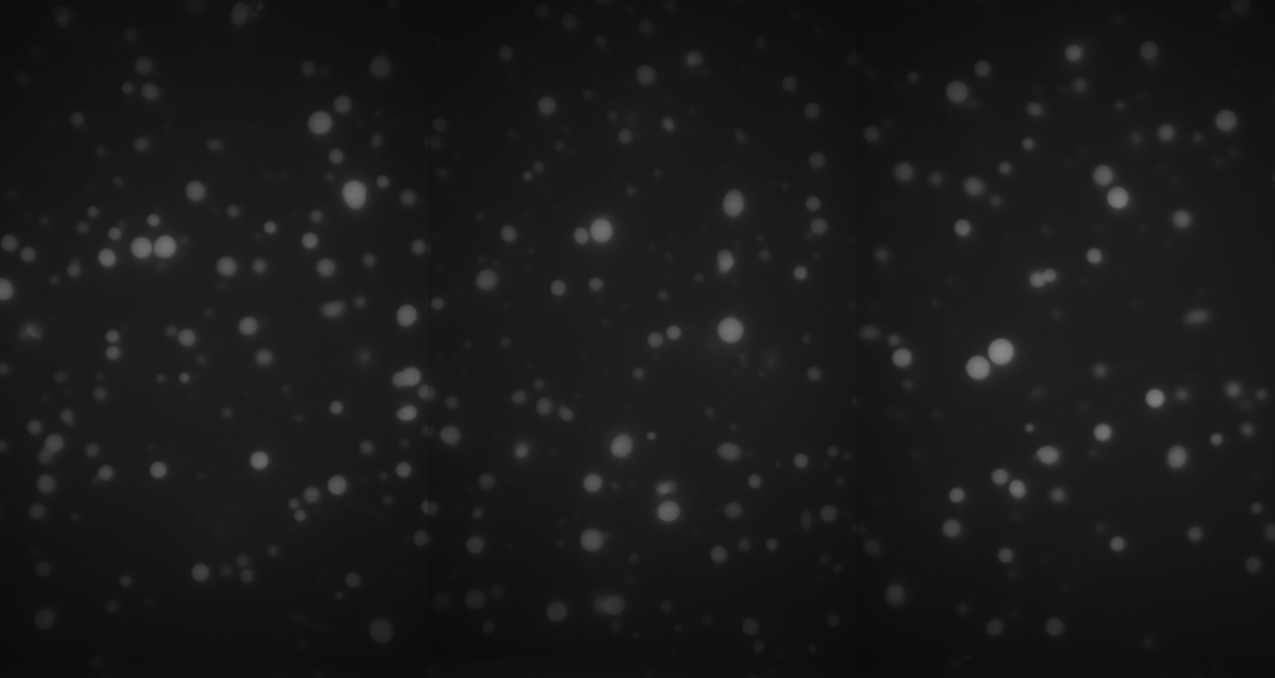

Supplement: Supplementary file 10 — Source data Fig. 8 [file 44318_2026_814_MOESM10_ESM.zip › Figure 9 A/NSP5 HP CTD/2024_10_01_25uM-NSP2-A488_18uM-NSP5-RF-HP_6uM-NSP5-RF-CTD_10min-scan-3.tif]

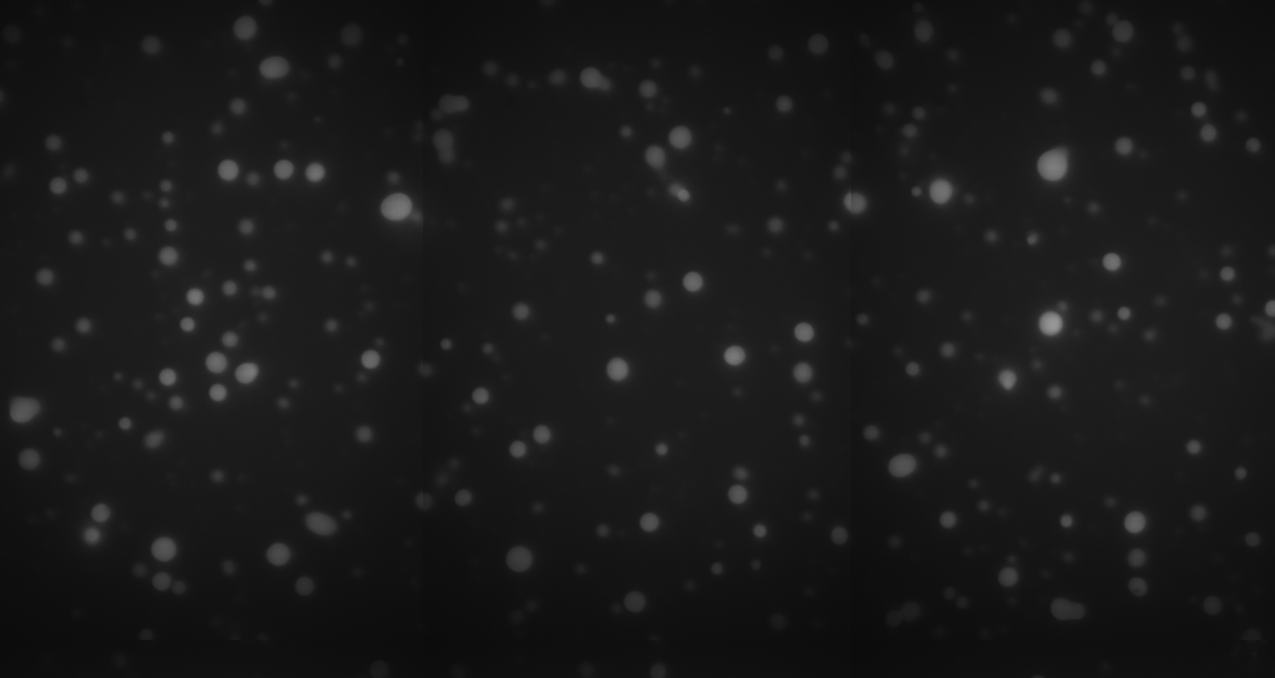

Supplement: Supplementary file 10 — Source data Fig. 8 [file 44318_2026_814_MOESM10_ESM.zip › Figure 9 A/NSP5 HP CTD/2024_10_01_25uM-NSP2-A488_18uM-NSP5-RF-HP_6uM-NSP5-RF-CTD_10min-scan-2.tif]

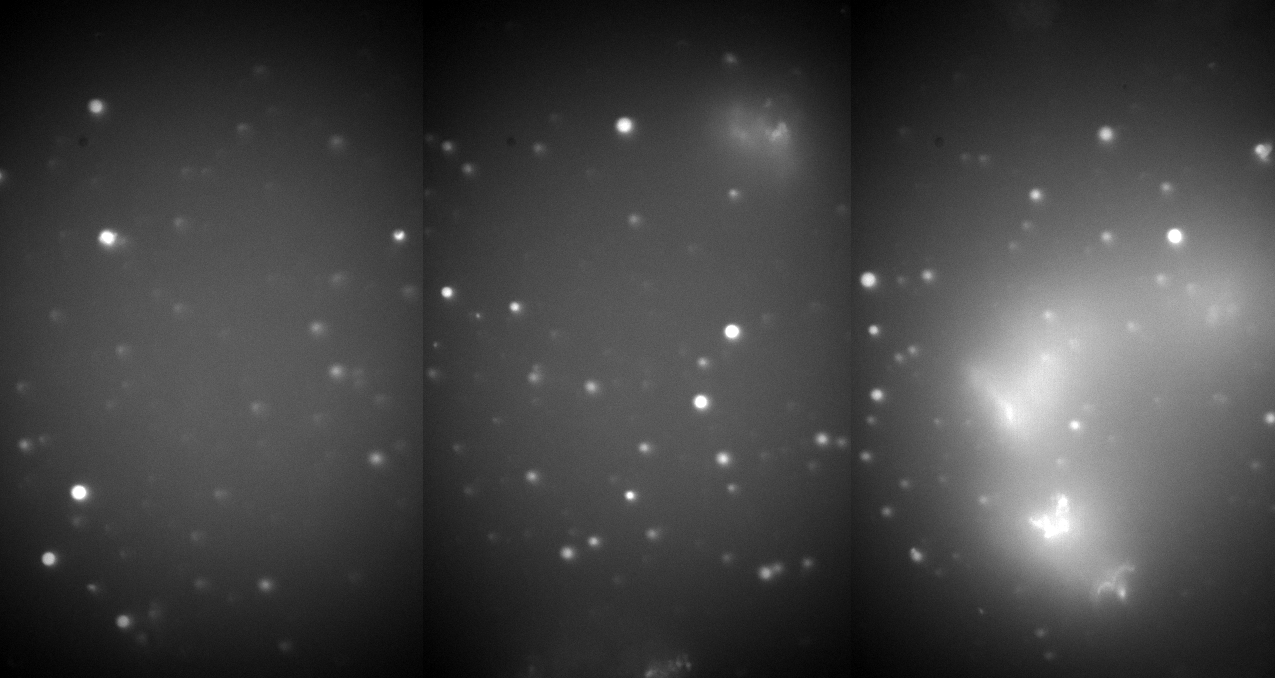

Supplement: Supplementary file 10 — Source data Fig. 8 [file 44318_2026_814_MOESM10_ESM.zip › Figure 9 A/NSP5 HP CTD/2024_10_01_25uM-NSP2-A488_12uM-NSP5-RF-HP_12uM-NSP5-RF-CTD_10min-scan1-1.tif]

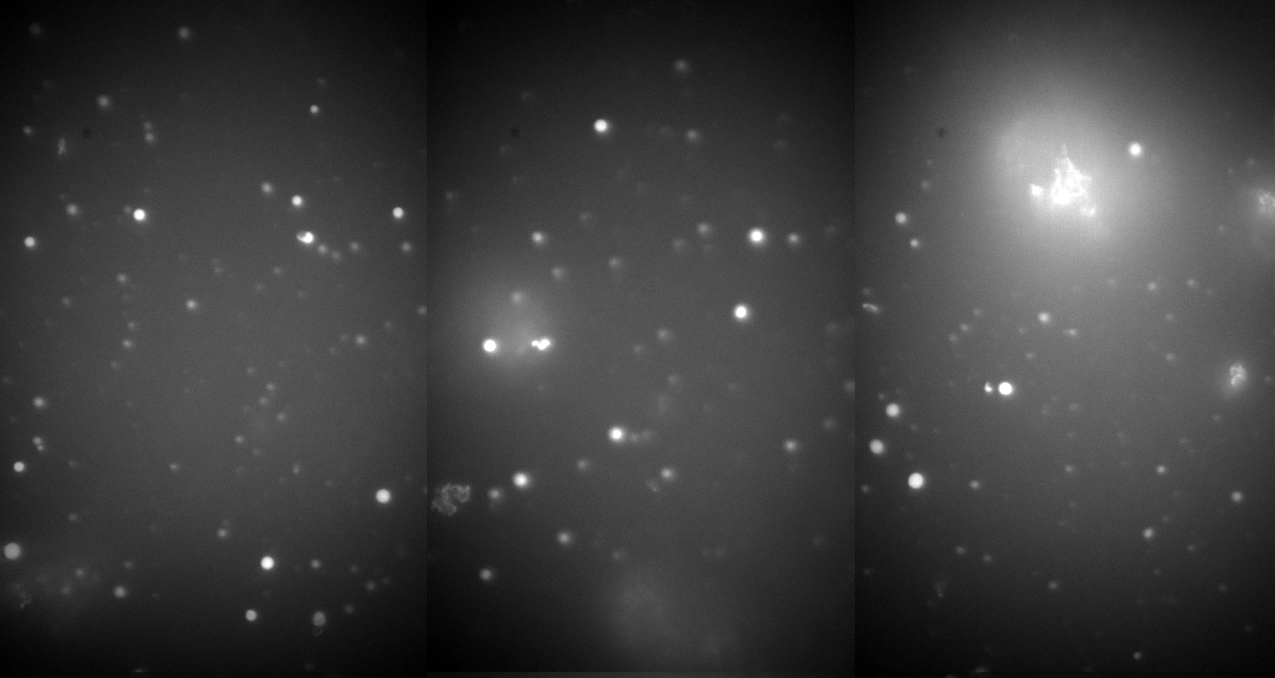

Supplement: Supplementary file 10 — Source data Fig. 8 [file 44318_2026_814_MOESM10_ESM.zip › Figure 9 A/NSP5 HP CTD/2024_10_01_25uM-NSP2-A488_12uM-NSP5-RF-HP_12uM-NSP5-RF-CTD_10min-scan1-3.tif]

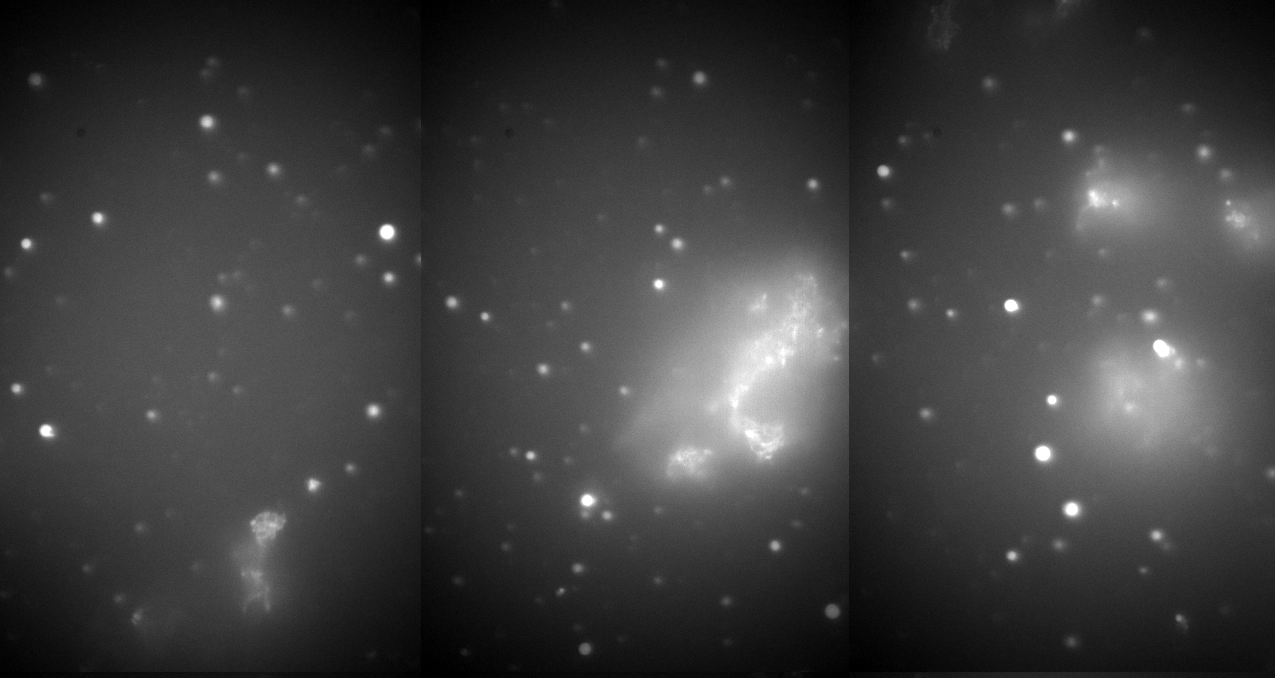

Supplement: Supplementary file 10 — Source data Fig. 8 [file 44318_2026_814_MOESM10_ESM.zip › Figure 9 A/NSP5 HP CTD/2024_10_01_25uM-NSP2-A488_12uM-NSP5-RF-HP_12uM-NSP5-RF-CTD_10min-scan1-2.tif]
