# Supplementary figures and images for: Phosphorylation tunes strain-specific protein condensation during rotavirus replication organelle assembly (part 3 of 3)
Source: EMBO J. 2026 May 26;45(13):4733–65. doi: 10.1038/s44318-026-00814-z (PMC13324165; doi:10.1038/s44318-026-00814-z)

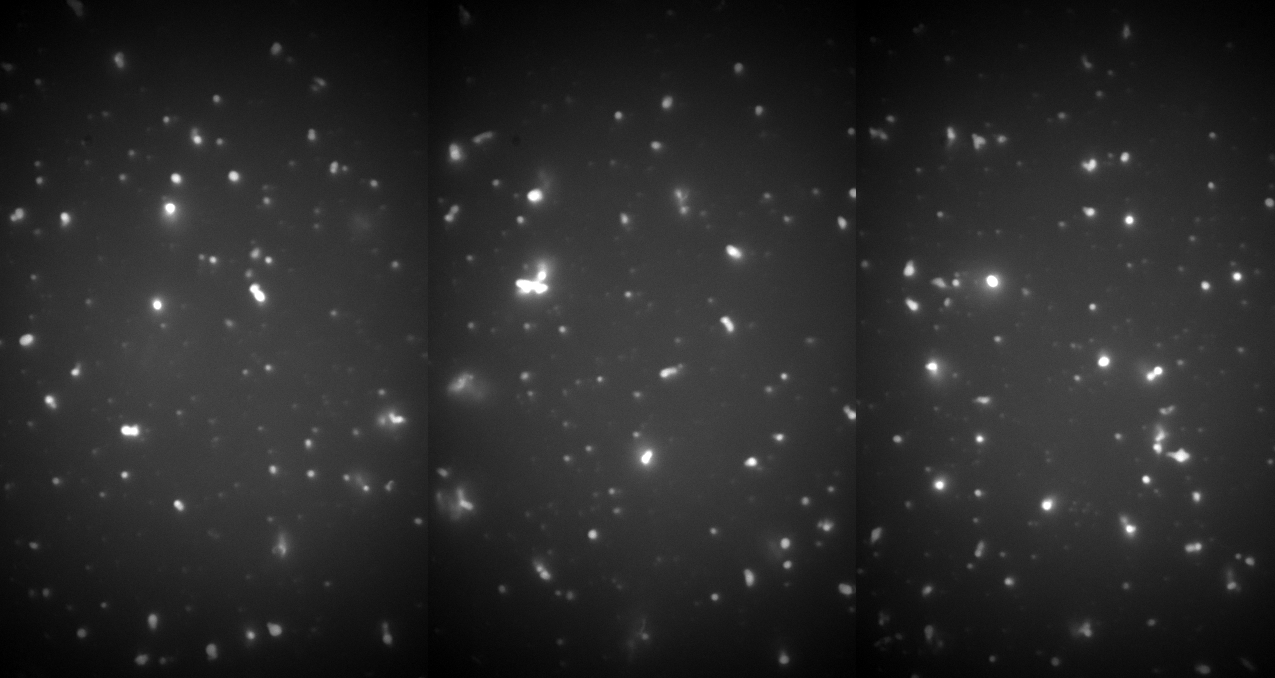

Supplement: Supplementary file 10 — Source data Fig. 8 [file 44318_2026_814_MOESM10_ESM.zip › Figure 9 A/NSP5 HP DeltaC HP/2024_10_01_25uM-NSP2-A488_6uM-NSP5-RF-HP_18uM-NSP5-RF-HP-DeltaCHP_10min-scan-2.tif]

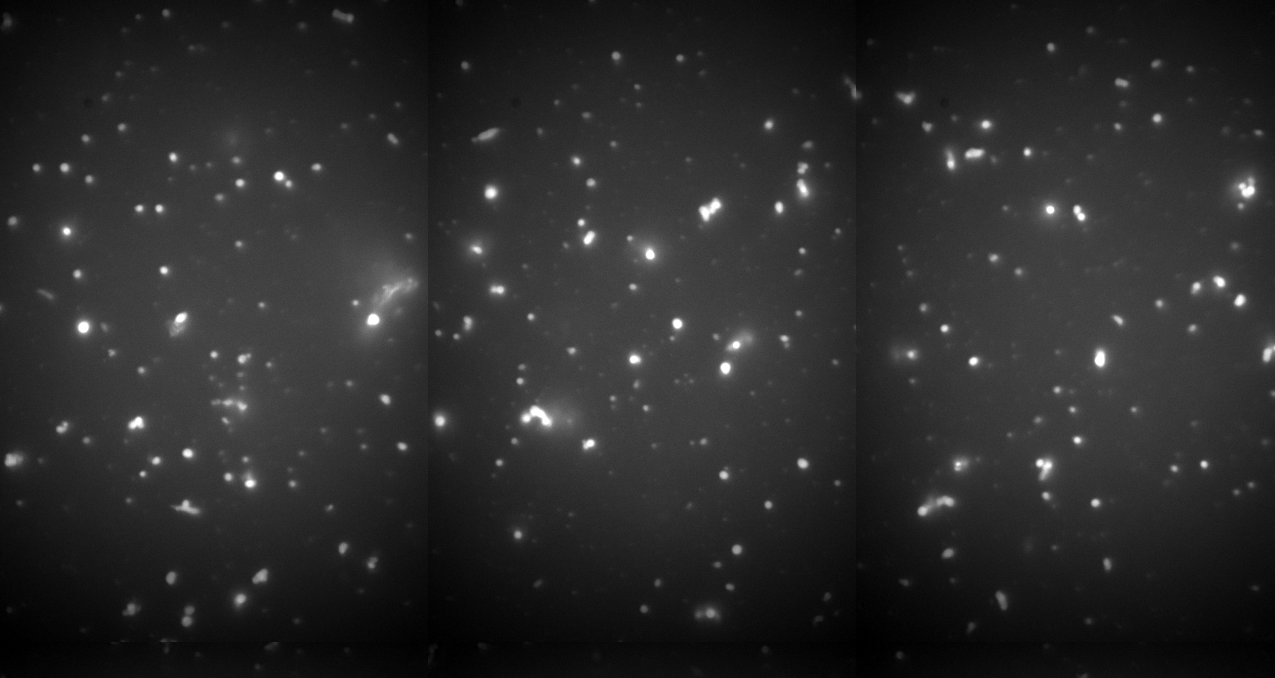

Supplement: Supplementary file 10 — Source data Fig. 8 [file 44318_2026_814_MOESM10_ESM.zip › Figure 9 A/NSP5 HP DeltaC HP/2024_10_01_25uM-NSP2-A488_6uM-NSP5-RF-HP_18uM-NSP5-RF-HP-DeltaCHP_10min-scan-3.tif]

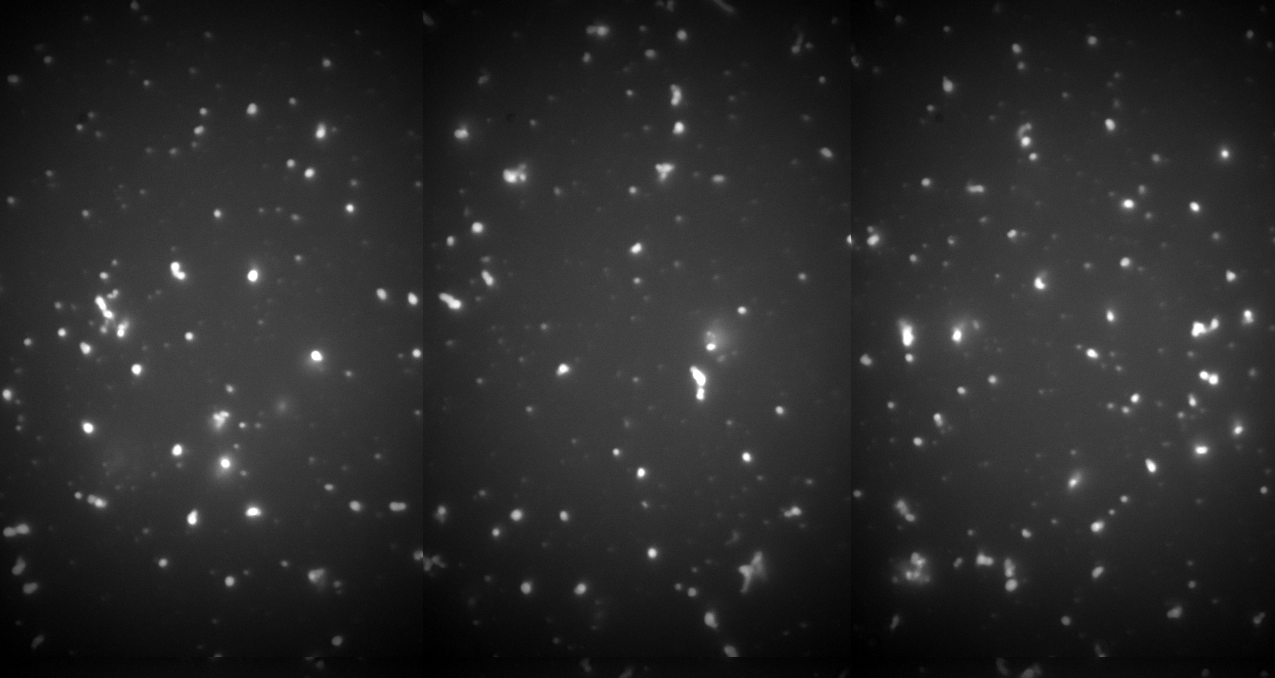

Supplement: Supplementary file 10 — Source data Fig. 8 [file 44318_2026_814_MOESM10_ESM.zip › Figure 9 A/NSP5 HP DeltaC HP/2024_10_01_25uM-NSP2-A488_6uM-NSP5-RF-HP_18uM-NSP5-RF-HP-DeltaCHP_10min-scan-1.tif]

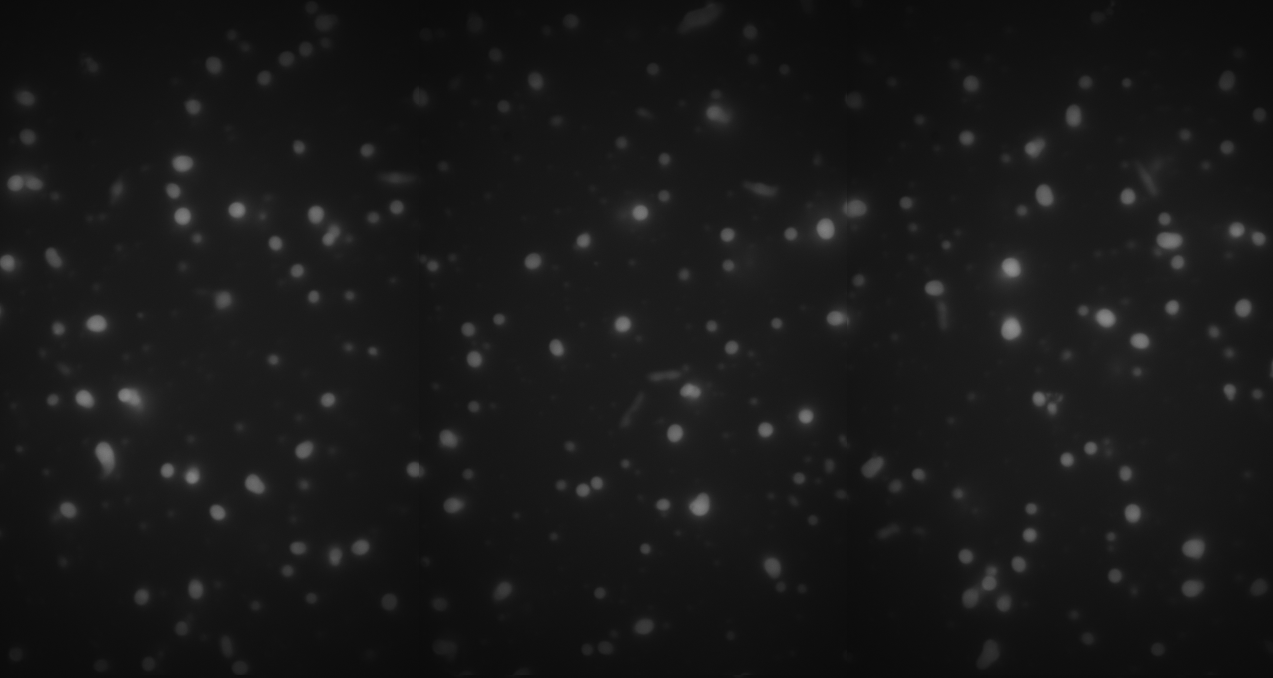

Supplement: Supplementary file 10 — Source data Fig. 8 [file 44318_2026_814_MOESM10_ESM.zip › Figure 9 A/NSP5 HP DeltaC HP/2024_10_01_25uM-NSP2-A488_18uM-NSP5-RF-HP_6uM-NSP5-RF-HP-DeltaCHP_10min-scan-1.tif]

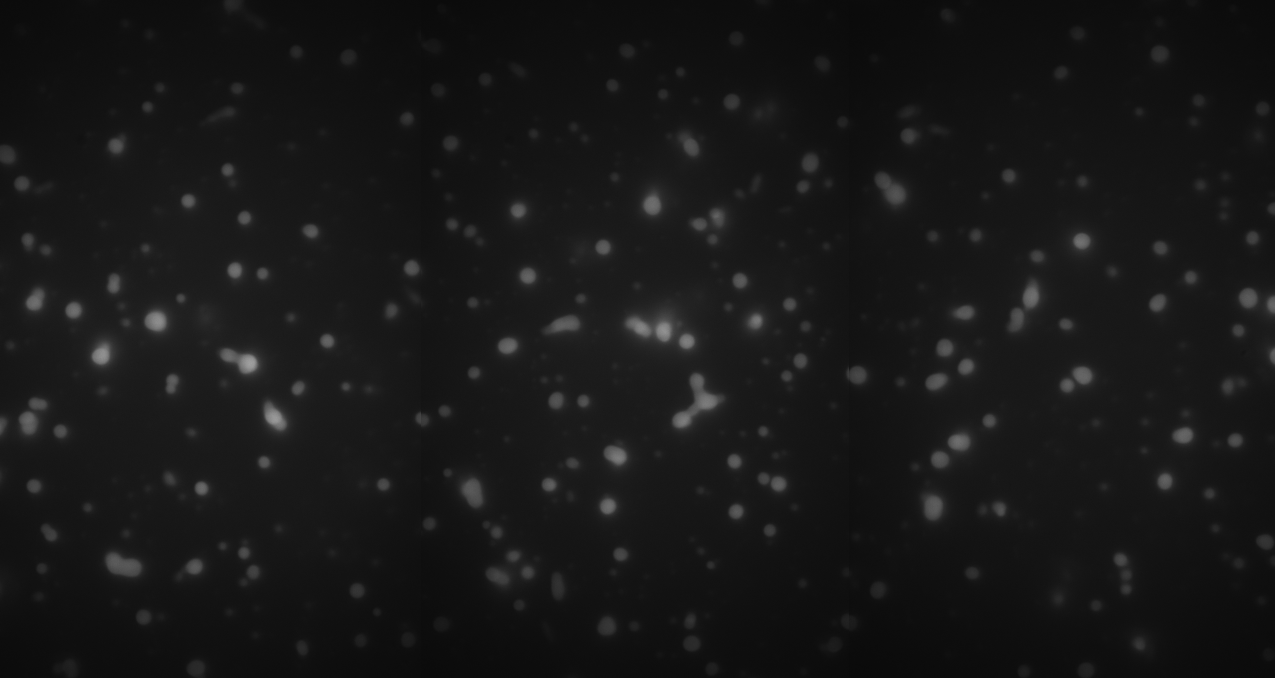

Supplement: Supplementary file 10 — Source data Fig. 8 [file 44318_2026_814_MOESM10_ESM.zip › Figure 9 A/NSP5 HP DeltaC HP/2024_10_01_25uM-NSP2-A488_18uM-NSP5-RF-HP_6uM-NSP5-RF-HP-DeltaCHP_10min-scan-3.tif]

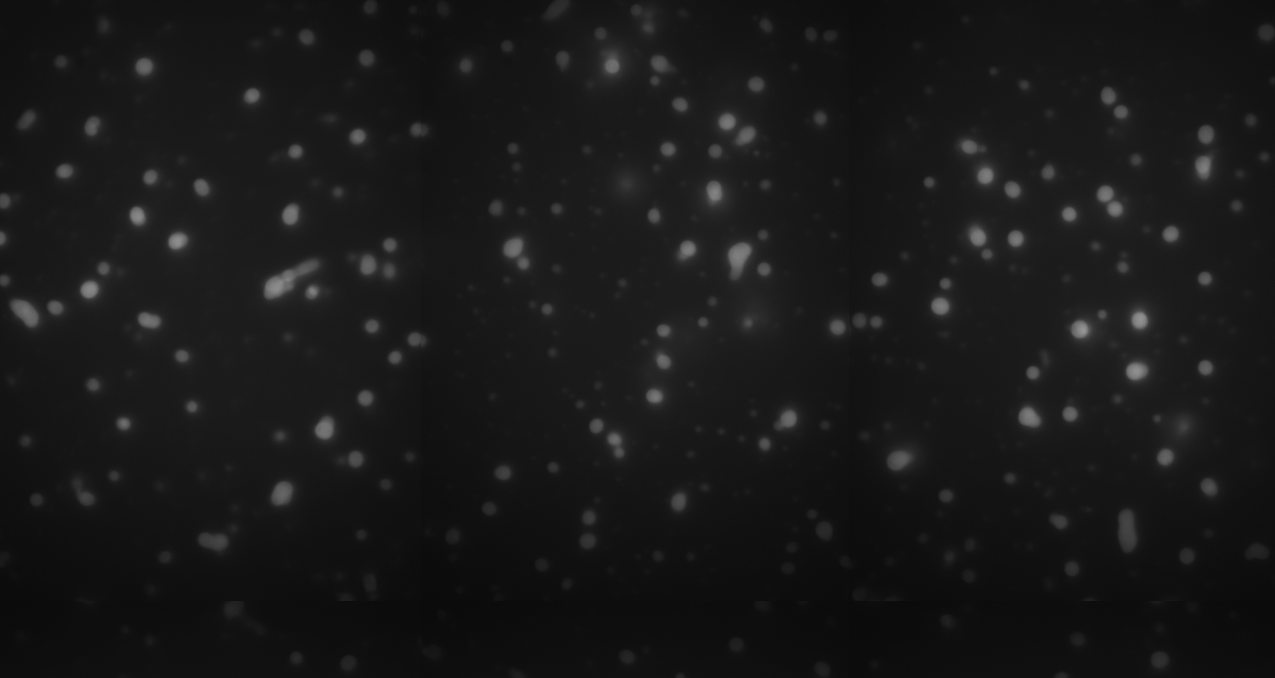

Supplement: Supplementary file 10 — Source data Fig. 8 [file 44318_2026_814_MOESM10_ESM.zip › Figure 9 A/NSP5 HP DeltaC HP/2024_10_01_25uM-NSP2-A488_18uM-NSP5-RF-HP_6uM-NSP5-RF-HP-DeltaCHP_10min-scan-2.tif]

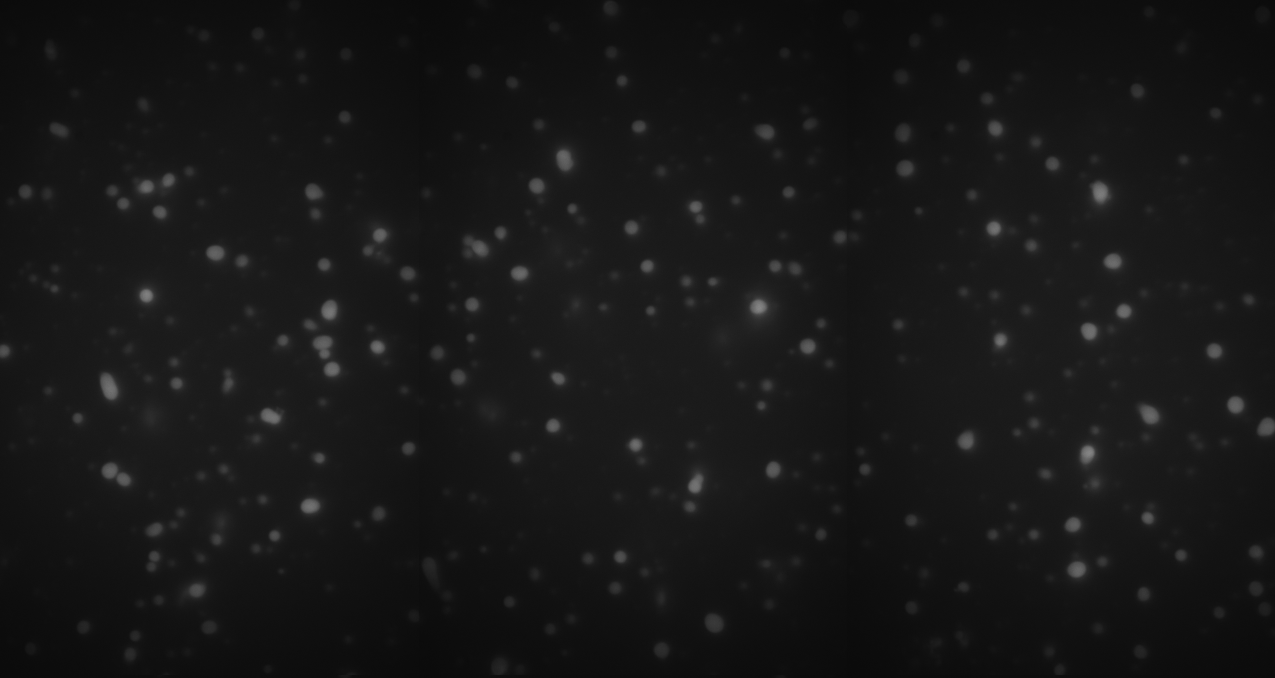

Supplement: Supplementary file 10 — Source data Fig. 8 [file 44318_2026_814_MOESM10_ESM.zip › Figure 9 A/NSP5 HP DeltaC HP/2024_10_01_25uM-NSP2-A488_12uM-NSP5-RF-HP_12uM-NSP5-RF-HP-DeltaCHP_10min-scan-1.tif]

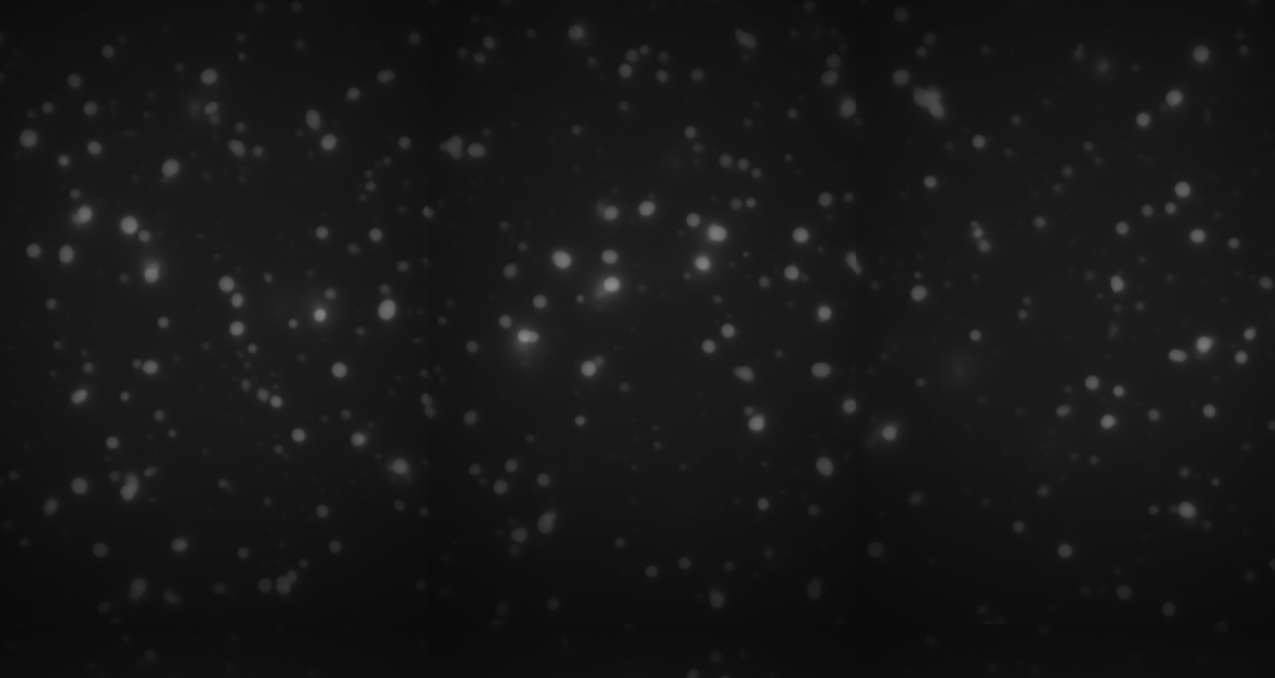

Supplement: Supplementary file 10 — Source data Fig. 8 [file 44318_2026_814_MOESM10_ESM.zip › Figure 9 A/NSP5 HP DeltaC HP/2024_10_01_25uM-NSP2-A488_12uM-NSP5-RF-HP_12uM-NSP5-RF-HP-DeltaCHP_10min-scan-2.tif]

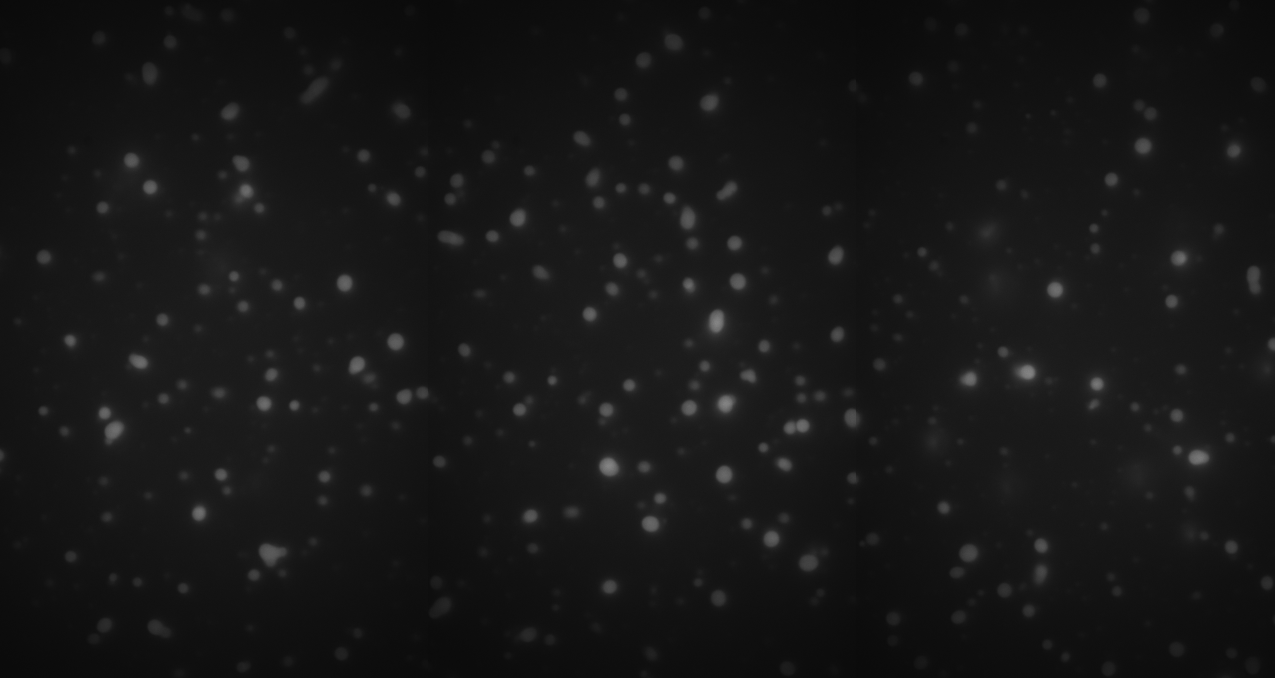

Supplement: Supplementary file 10 — Source data Fig. 8 [file 44318_2026_814_MOESM10_ESM.zip › Figure 9 A/NSP5 HP DeltaC HP/2024_10_01_25uM-NSP2-A488_12uM-NSP5-RF-HP_12uM-NSP5-RF-HP-DeltaCHP_10min-scan-3.tif]

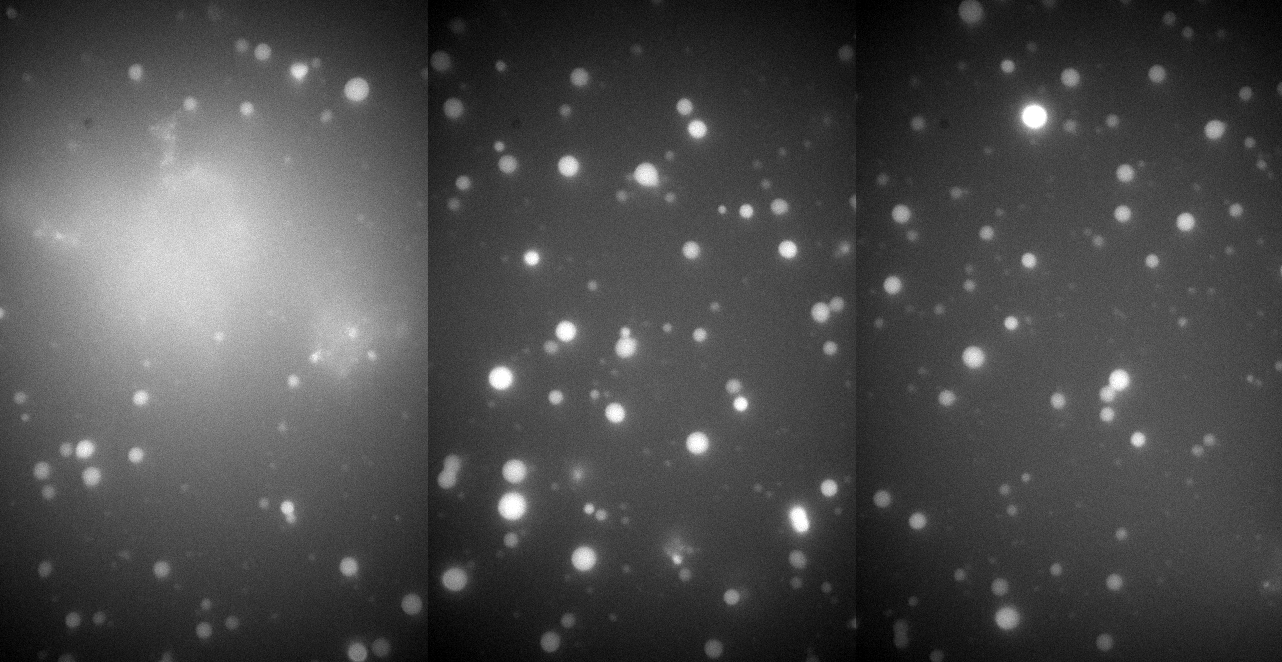

Supplement: Supplementary file 10 — Source data Fig. 8 [file 44318_2026_814_MOESM10_ESM.zip › Figure 9 A/NSP5 WT CTD/50uM-NSP2-RF-WT-A488_37-5uM-NSP5-RF-WT_12-5uM-NSP5-RF-CTD40-10min-scan-3.tif]

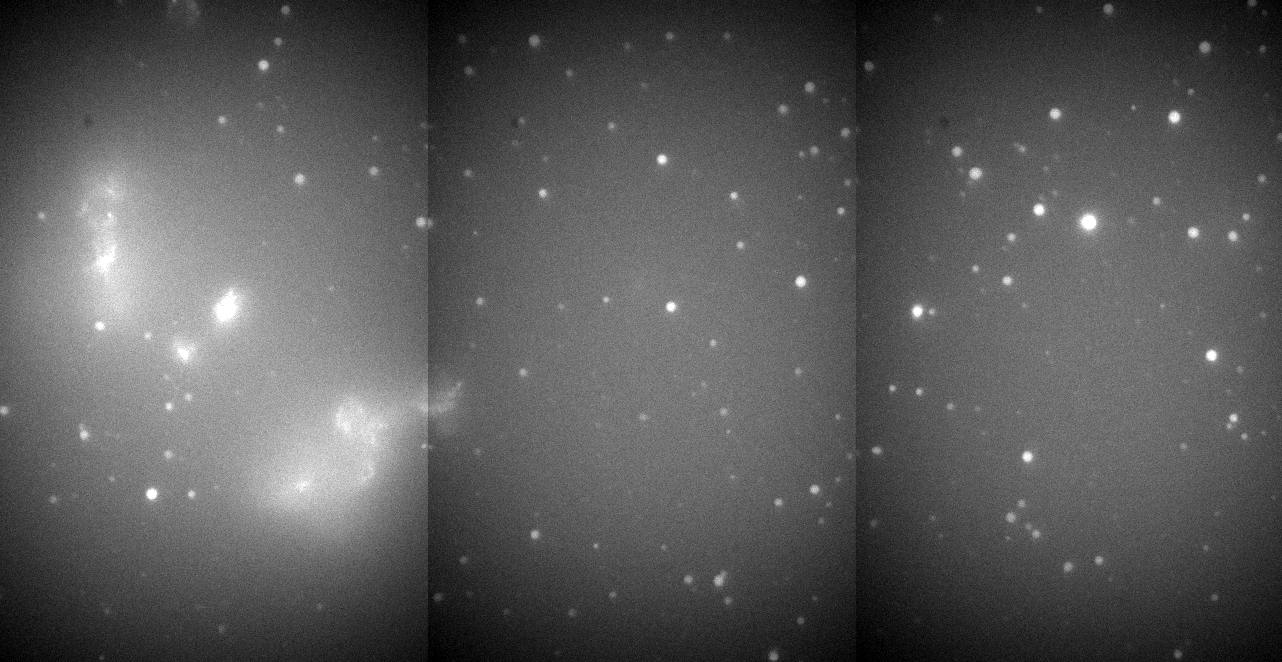

Supplement: Supplementary file 10 — Source data Fig. 8 [file 44318_2026_814_MOESM10_ESM.zip › Figure 9 A/NSP5 WT CTD/50uM-NSP2-RF-WT-A488_25uM-NSP5-RF-WT_25uM-NSP5-RF-CTD40-10min-scan-3.tif]

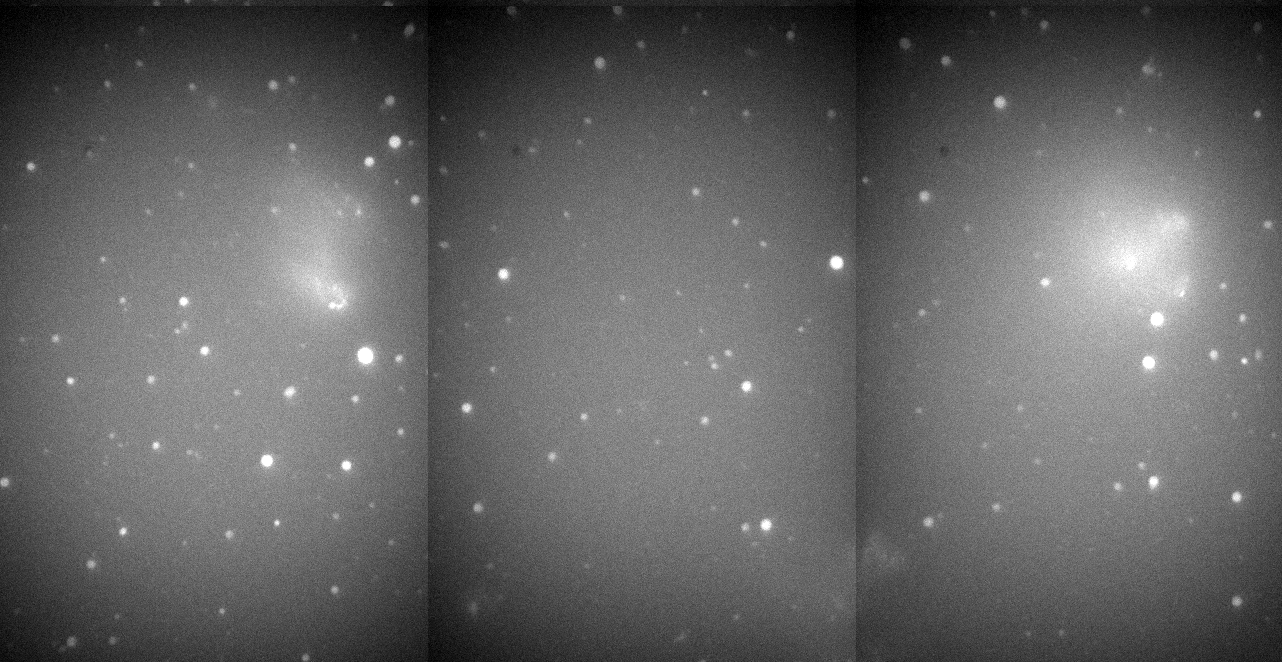

Supplement: Supplementary file 10 — Source data Fig. 8 [file 44318_2026_814_MOESM10_ESM.zip › Figure 9 A/NSP5 WT CTD/50uM-NSP2-RF-WT-A488_25uM-NSP5-RF-WT_25uM-NSP5-RF-CTD40-10min-scan-2.tif]

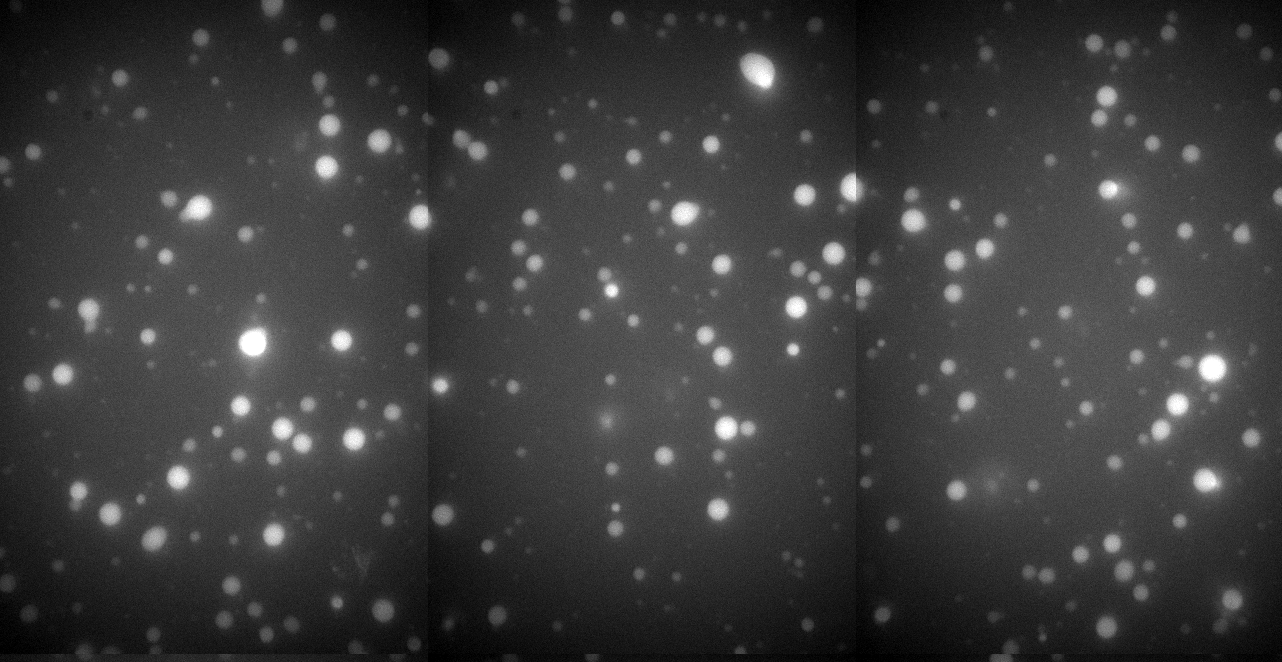

Supplement: Supplementary file 10 — Source data Fig. 8 [file 44318_2026_814_MOESM10_ESM.zip › Figure 9 A/NSP5 WT CTD/50uM-NSP2-RF-WT-A488_37-5uM-NSP5-RF-WT_12-5uM-NSP5-RF-CTD40-10min-scan-2.tif]

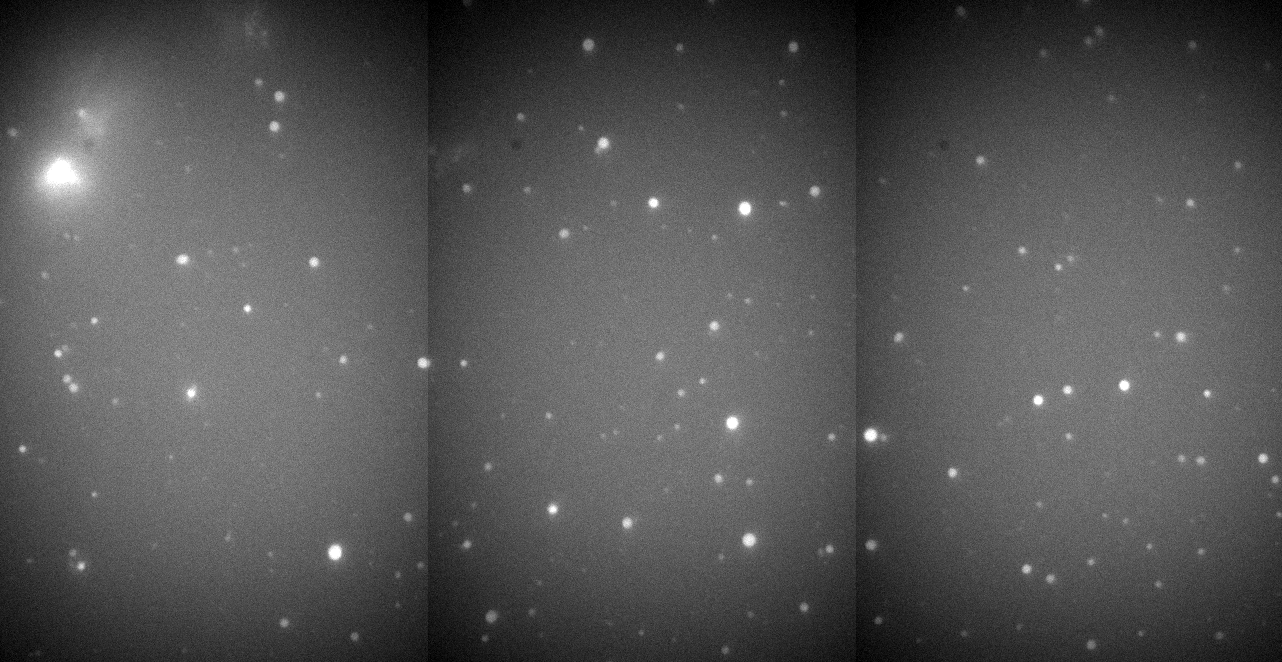

Supplement: Supplementary file 10 — Source data Fig. 8 [file 44318_2026_814_MOESM10_ESM.zip › Figure 9 A/NSP5 WT CTD/50uM-NSP2-RF-WT-A488_25uM-NSP5-RF-WT_25uM-NSP5-RF-CTD40-10min-scan-1.tif]

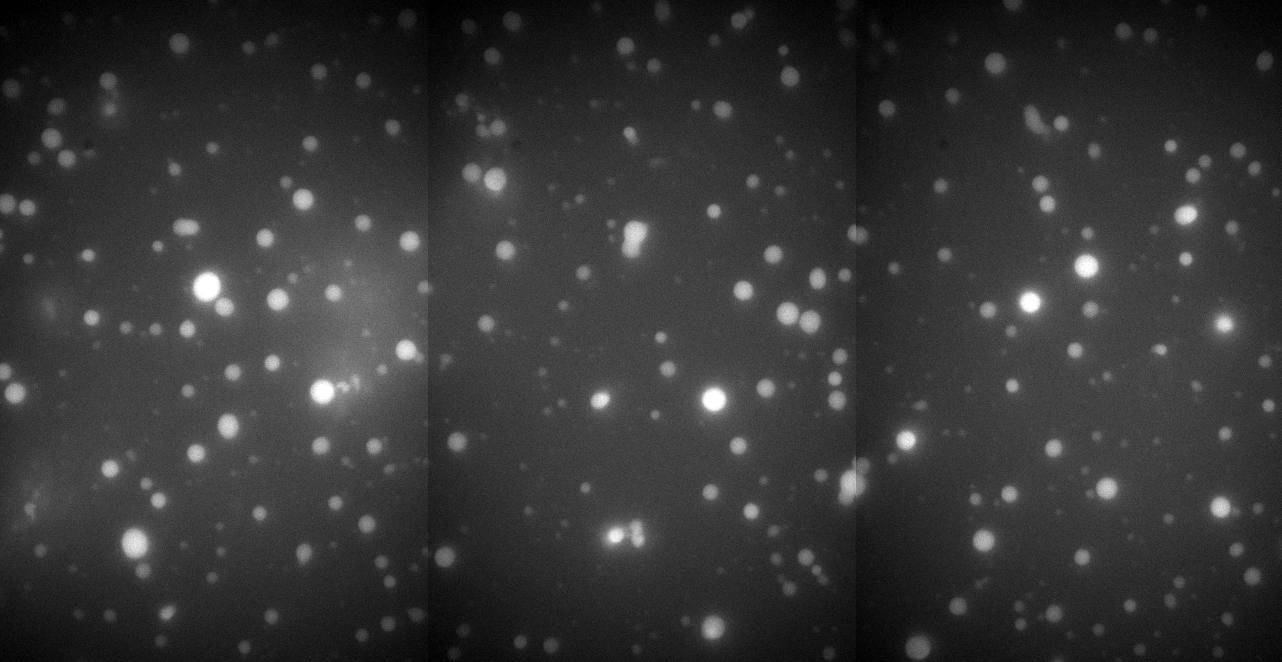

Supplement: Supplementary file 10 — Source data Fig. 8 [file 44318_2026_814_MOESM10_ESM.zip › Figure 9 A/NSP5 WT CTD/50uM-NSP2-RF-WT-A488_37-5uM-NSP5-RF-WT_12-5uM-NSP5-RF-CTD40-10min-scan-1.tif]

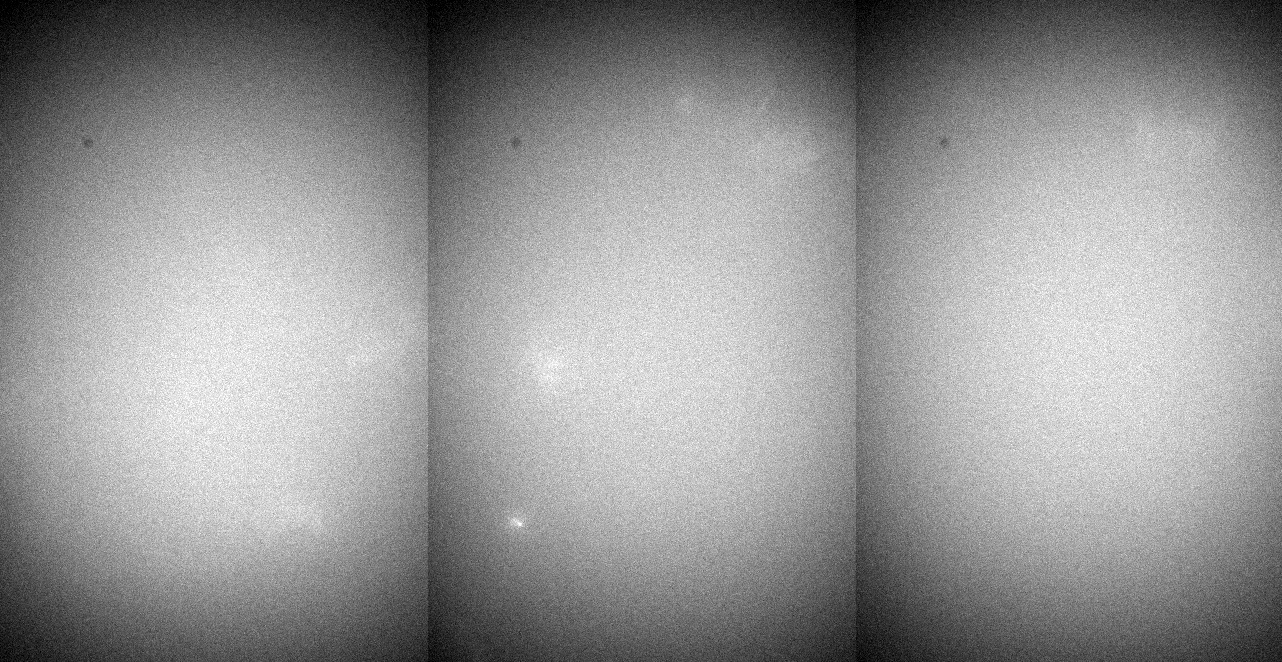

Supplement: Supplementary file 10 — Source data Fig. 8 [file 44318_2026_814_MOESM10_ESM.zip › Figure 9 A/NSP5 WT CTD/50uM-NSP2-RF-WT-A488_12-5uM-NSP5-RF-WT_37-5uM-NSP5-RF-CTD40-10min-scan-2.tif]

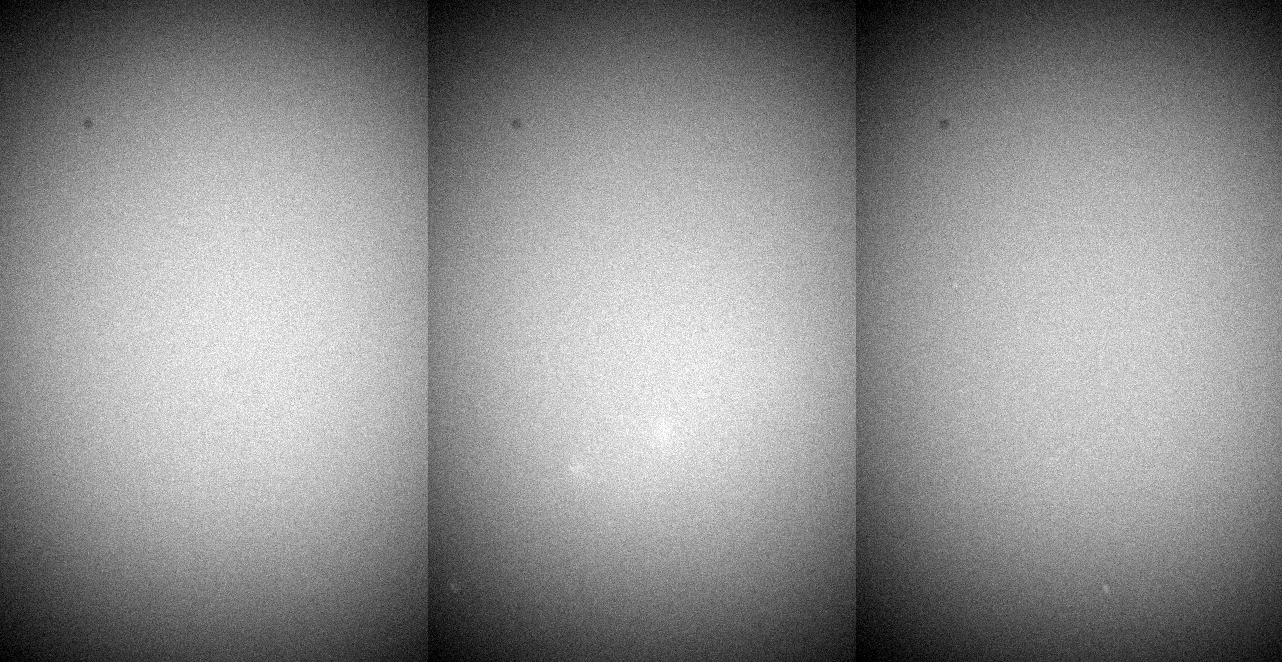

Supplement: Supplementary file 10 — Source data Fig. 8 [file 44318_2026_814_MOESM10_ESM.zip › Figure 9 A/NSP5 WT CTD/50uM-NSP2-RF-WT-A488_12-5uM-NSP5-RF-WT_37-5uM-NSP5-RF-CTD40-10min-scan-3.tif]

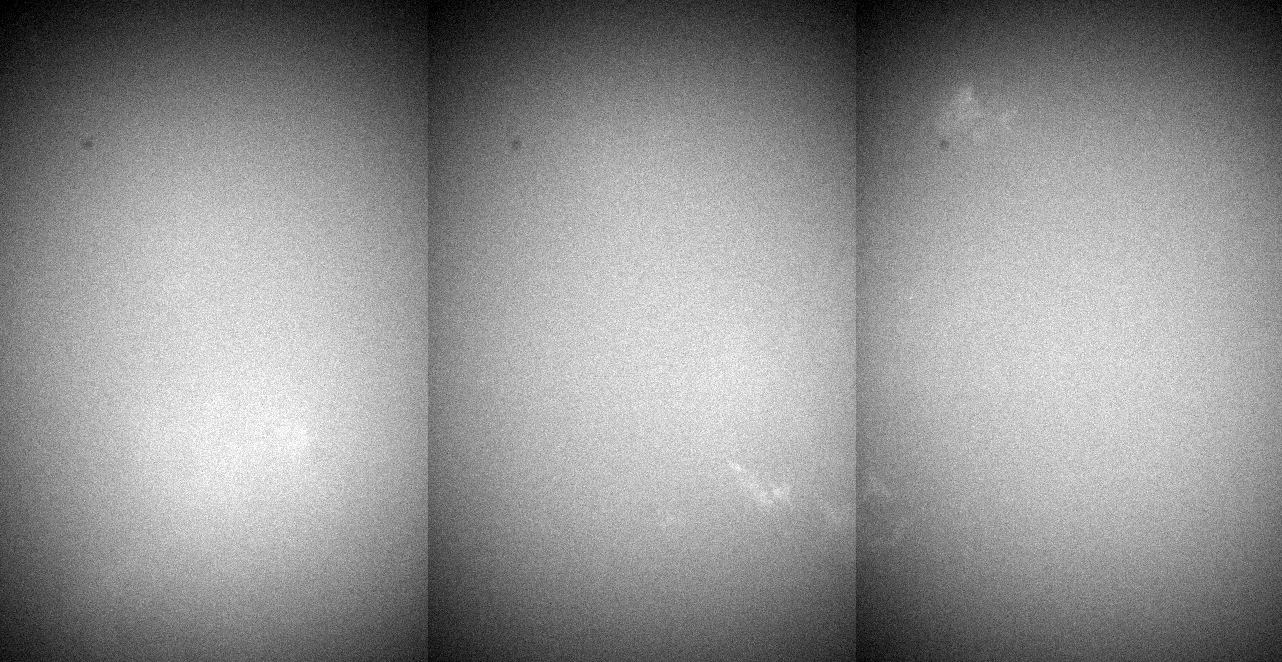

Supplement: Supplementary file 10 — Source data Fig. 8 [file 44318_2026_814_MOESM10_ESM.zip › Figure 9 A/NSP5 WT CTD/50uM-NSP2-RF-WT-A488_12-5uM-NSP5-RF-WT_37-5uM-NSP5-RF-CTD40-10min-scan-1.tif]

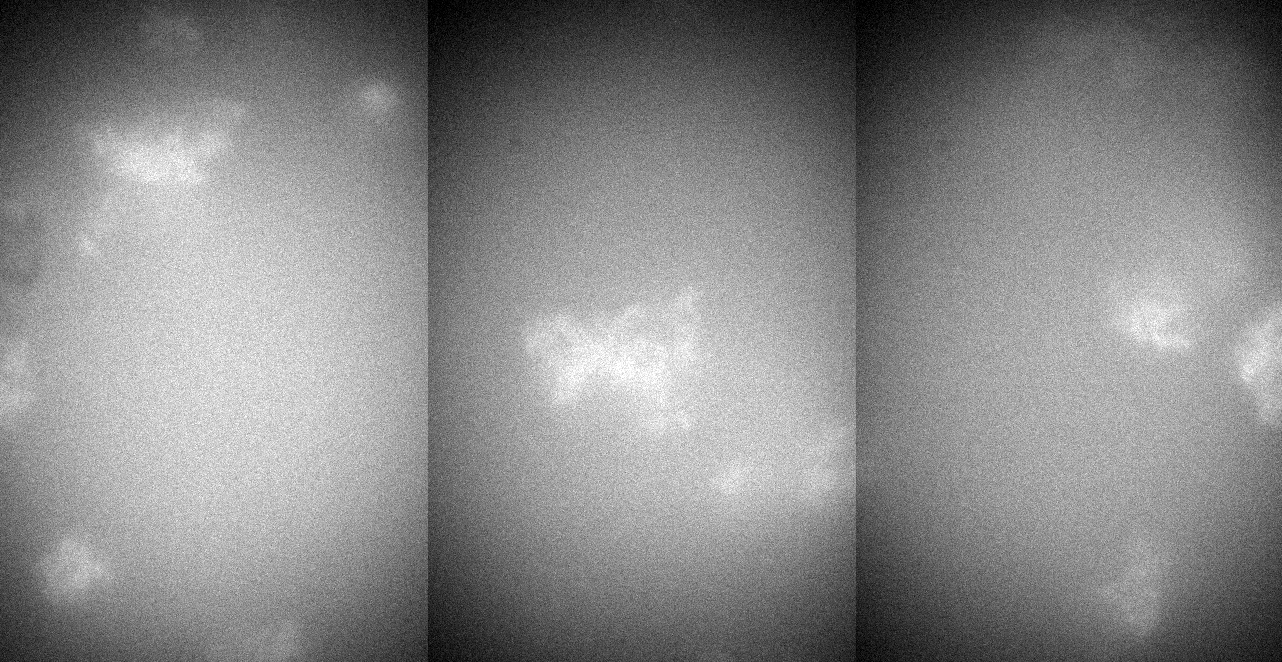

Supplement: Supplementary file 10 — Source data Fig. 8 [file 44318_2026_814_MOESM10_ESM.zip › Figure 9 A/NSP5 WT CTD/50uM-NSP2-RF-WT-A488_0uM-NSP5-RF-WT_50uM-NSP5-RF-CTD40-10min-scan-1.tif]

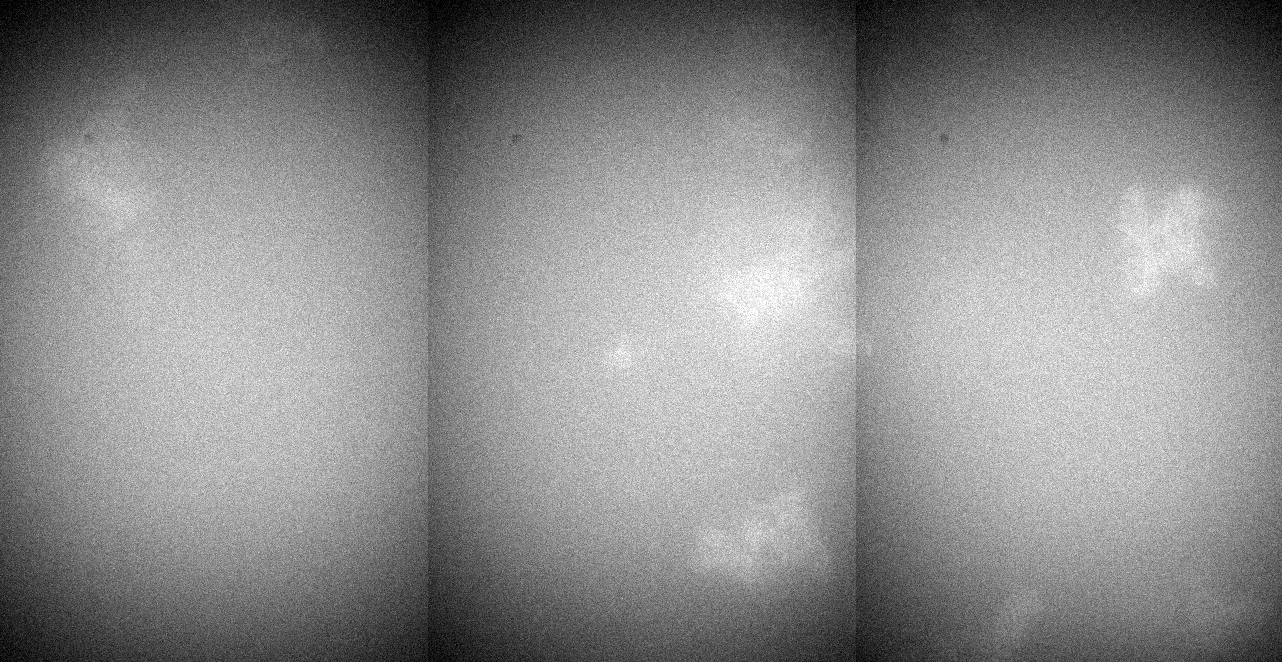

Supplement: Supplementary file 10 — Source data Fig. 8 [file 44318_2026_814_MOESM10_ESM.zip › Figure 9 A/NSP5 WT CTD/50uM-NSP2-RF-WT-A488_0uM-NSP5-RF-WT_50uM-NSP5-RF-CTD40-10min-scan-2.tif]

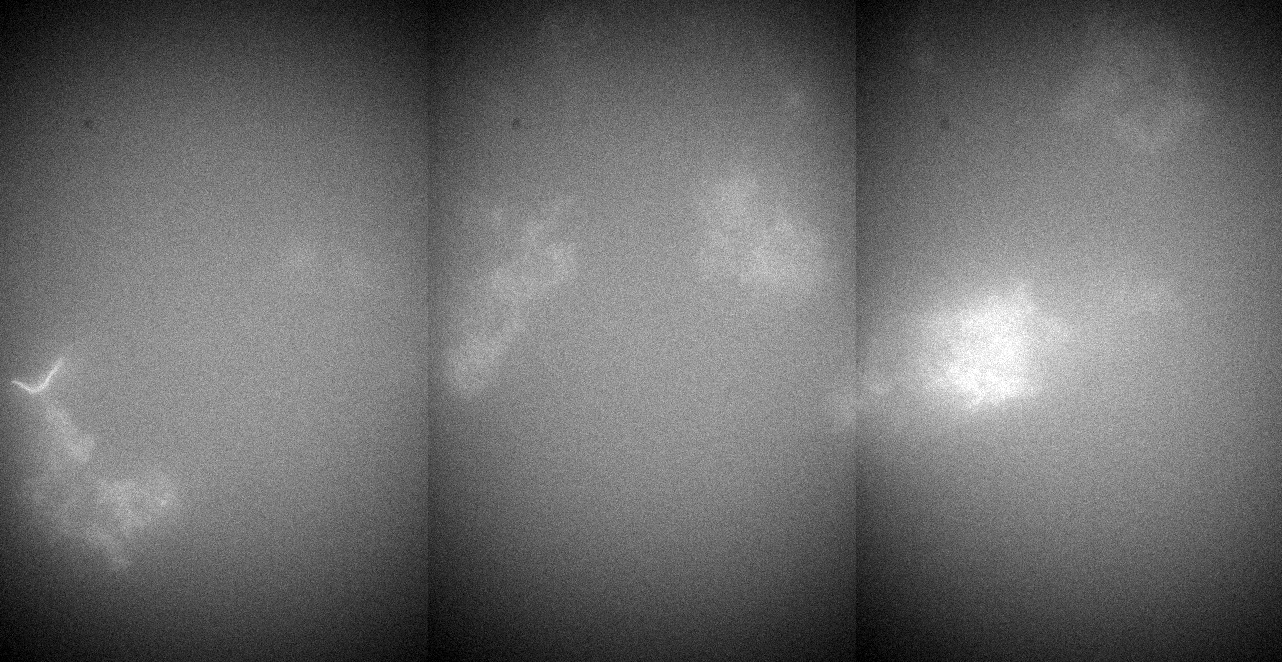

Supplement: Supplementary file 10 — Source data Fig. 8 [file 44318_2026_814_MOESM10_ESM.zip › Figure 9 A/NSP5 WT CTD/50uM-NSP2-RF-WT-A488_0uM-NSP5-RF-WT_50uM-NSP5-RF-CTD40-10min-scan-3.tif]

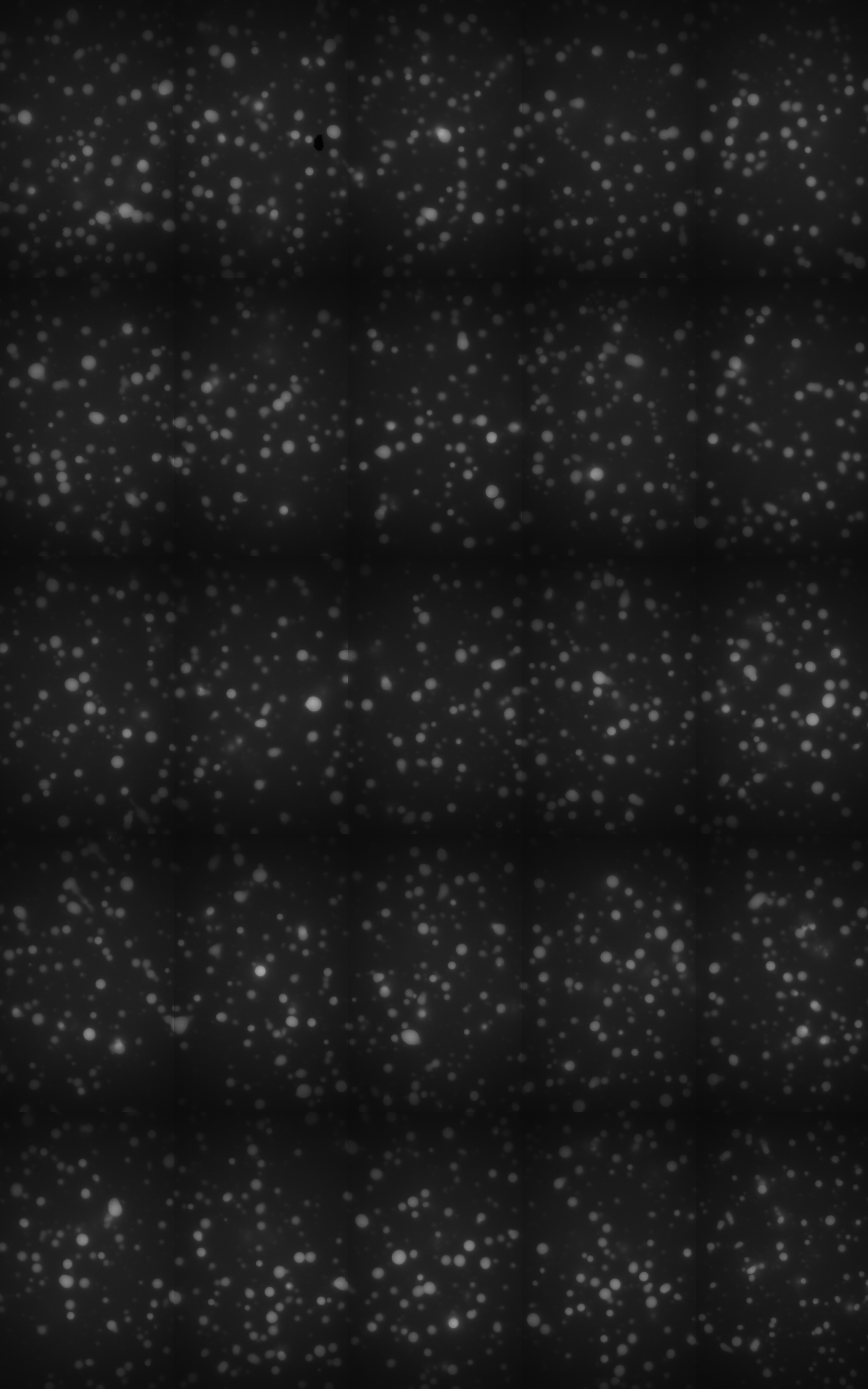

Supplement: Supplementary file 10 — Source data Fig. 8 [file 44318_2026_814_MOESM10_ESM.zip › Figure 9 A/NSP5 HP CTD/20241001_NSP5-RF-HP_CTD/2024_10_01_25uM-NSP2-A488_25uM-NSP5-RF-HP_10min-scan.tiff]

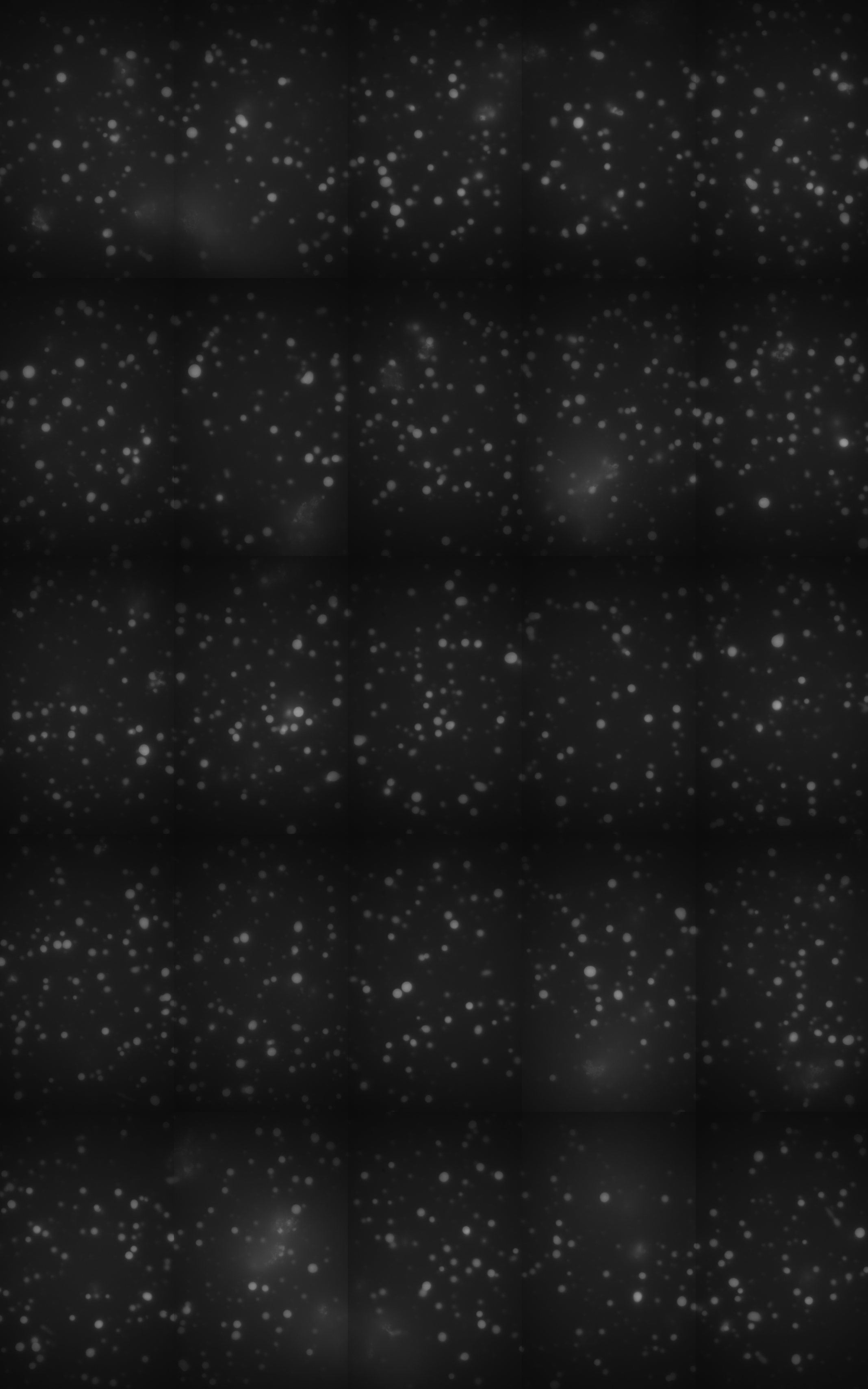

Supplement: Supplementary file 10 — Source data Fig. 8 [file 44318_2026_814_MOESM10_ESM.zip › Figure 9 A/NSP5 HP CTD/20241001_NSP5-RF-HP_CTD/2024_10_01_25uM-NSP2-A488_18uM-NSP5-RF-HP_6uM-NSP5-RF-CTD_10min-scan.tiff]

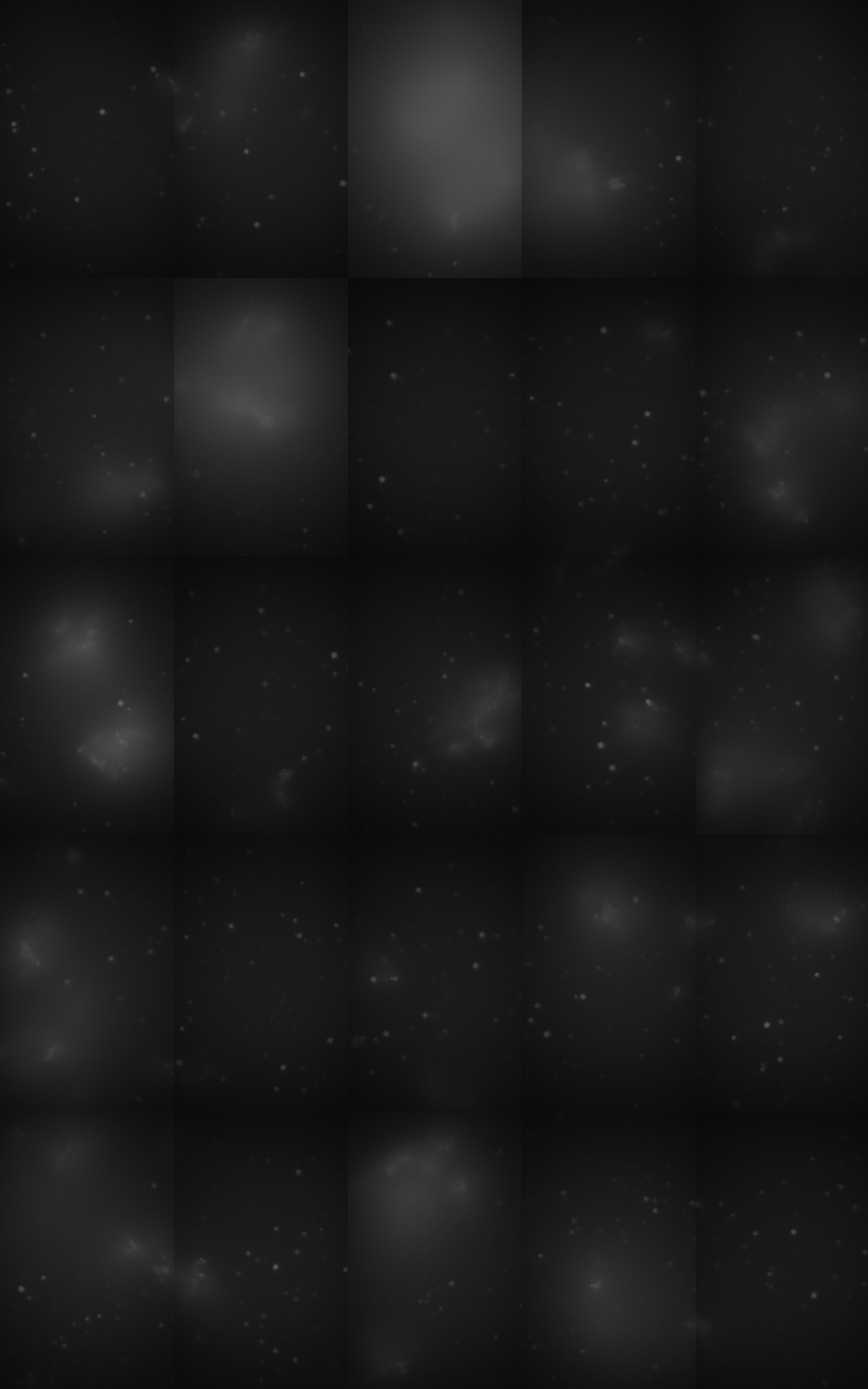

Supplement: Supplementary file 10 — Source data Fig. 8 [file 44318_2026_814_MOESM10_ESM.zip › Figure 9 A/NSP5 HP CTD/20241001_NSP5-RF-HP_CTD/2024_10_01_25uM-NSP2-A488_12uM-NSP5-RF-HP_12uM-NSP5-RF-CTD_10min-scan1.tiff]

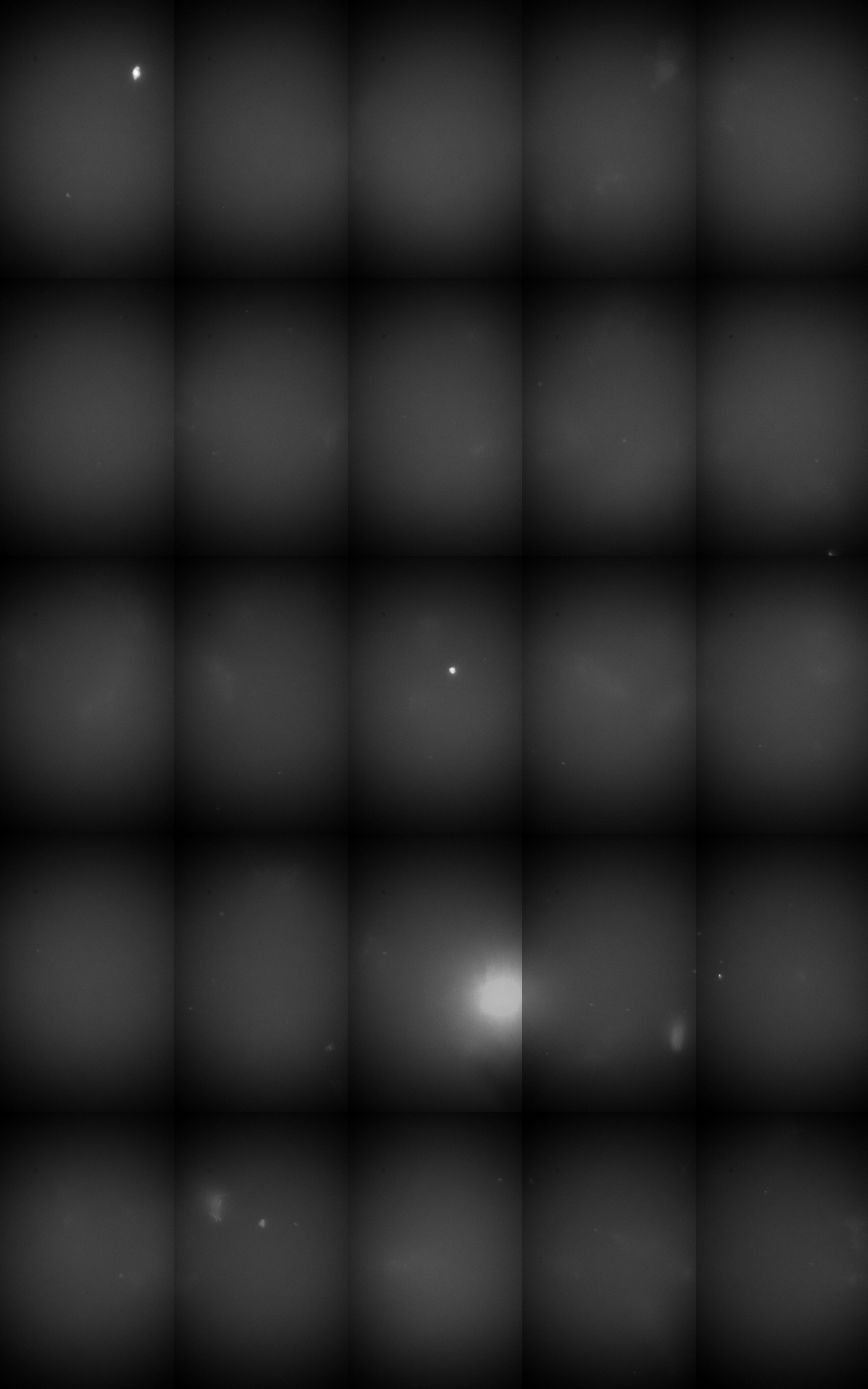

Supplement: Supplementary file 10 — Source data Fig. 8 [file 44318_2026_814_MOESM10_ESM.zip › Figure 9 A/NSP5 HP CTD/20241001_NSP5-RF-HP_CTD/2024_10_01_25uM-NSP2-A488_25uM-NSP5-RF-CTD_10min-scan.tiff]

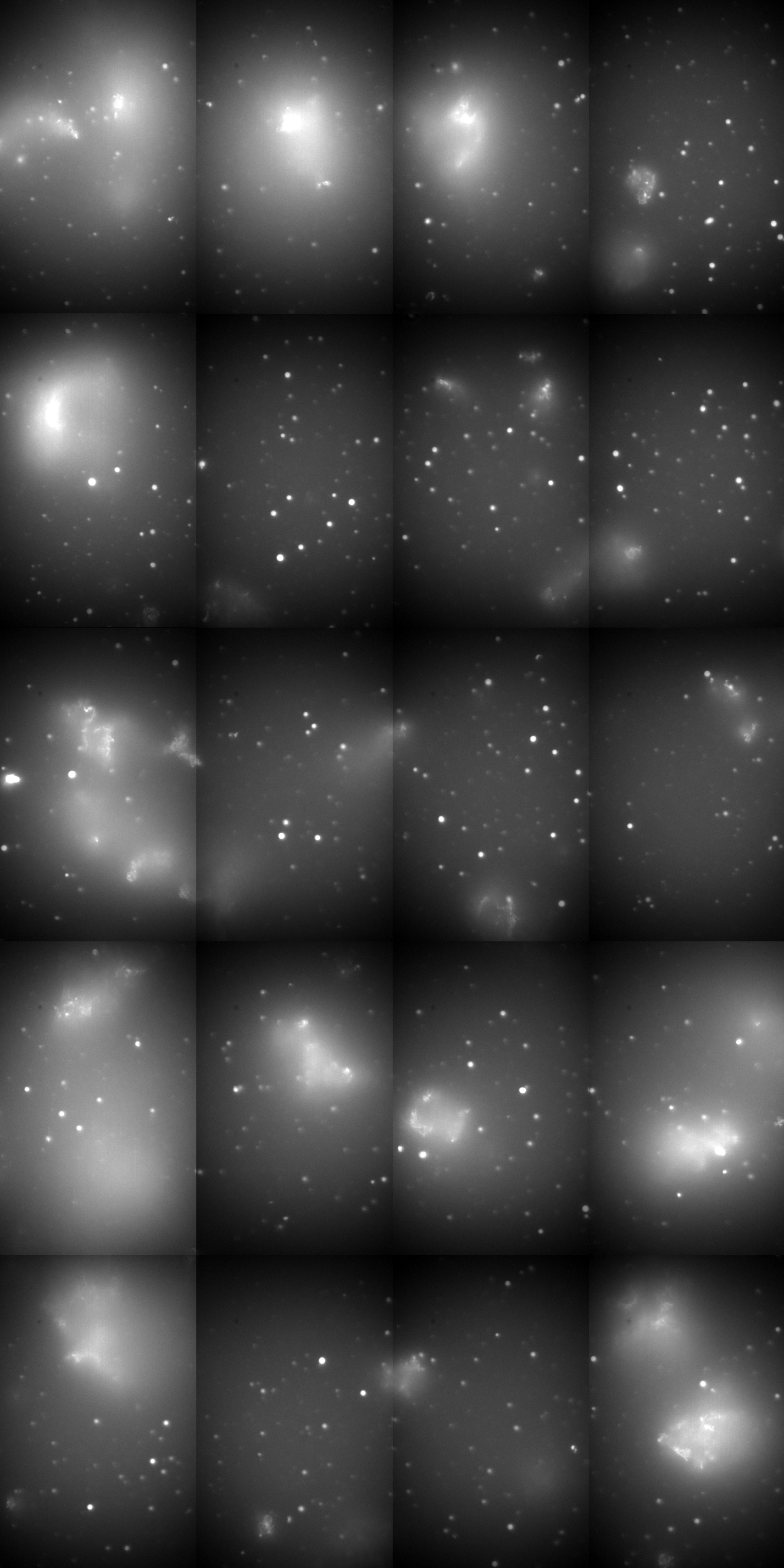

Supplement: Supplementary file 10 — Source data Fig. 8 [file 44318_2026_814_MOESM10_ESM.zip › Figure 9 A/NSP5 HP CTD/20241001_NSP5-RF-HP_CTD/2024_10_01_25uM-NSP2-A488_12uM-NSP5-RF-HP_12uM-NSP5-RF-CTD_10min-scan.tiff]

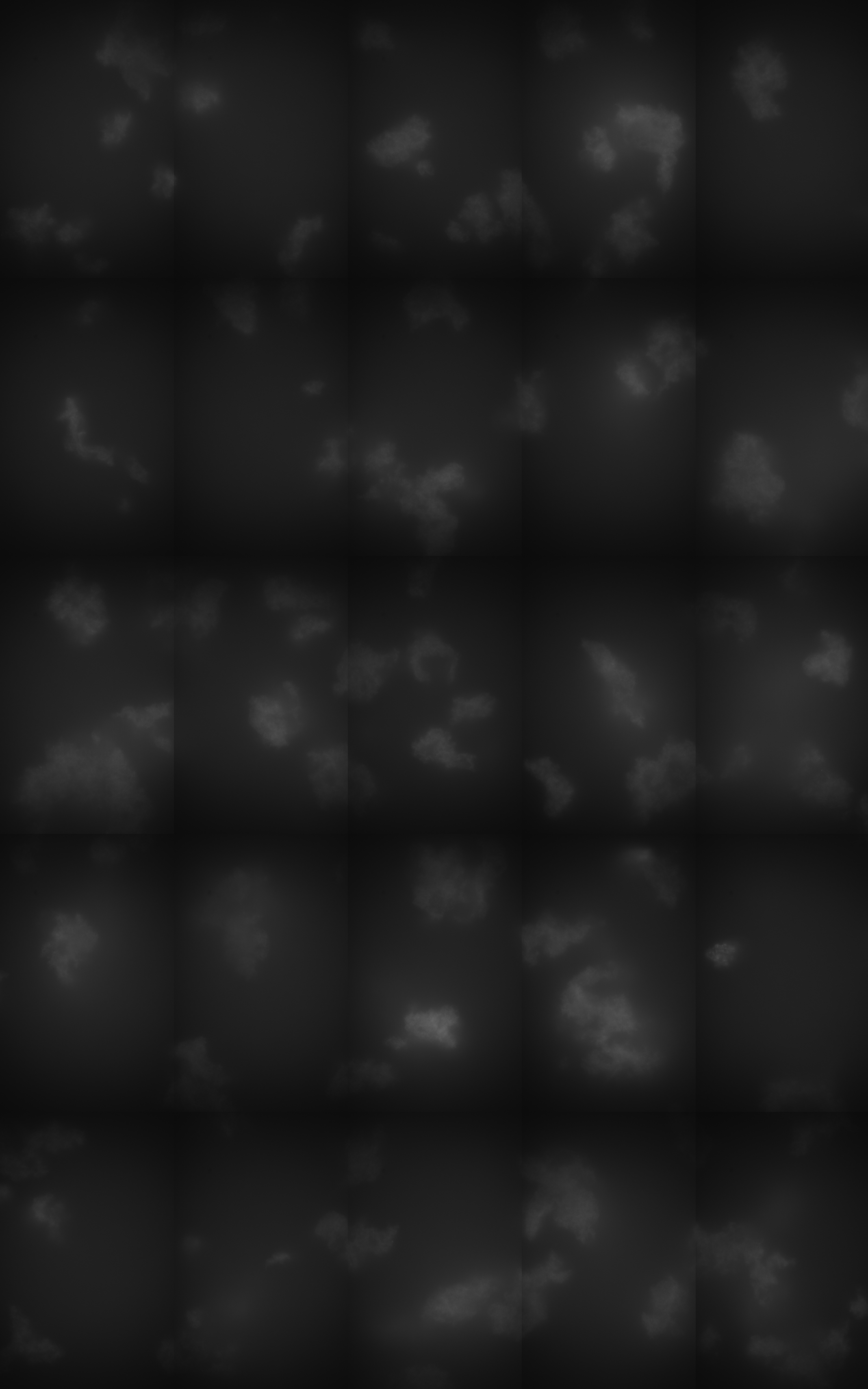

Supplement: Supplementary file 10 — Source data Fig. 8 [file 44318_2026_814_MOESM10_ESM.zip › Figure 9 A/NSP5 HP CTD/20241001_NSP5-RF-HP_CTD/2024_10_01_25uM-NSP2-A488_6uM-NSP5-RF-HP_18uM-NSP5-RF-CTD_10min-scan.tiff]

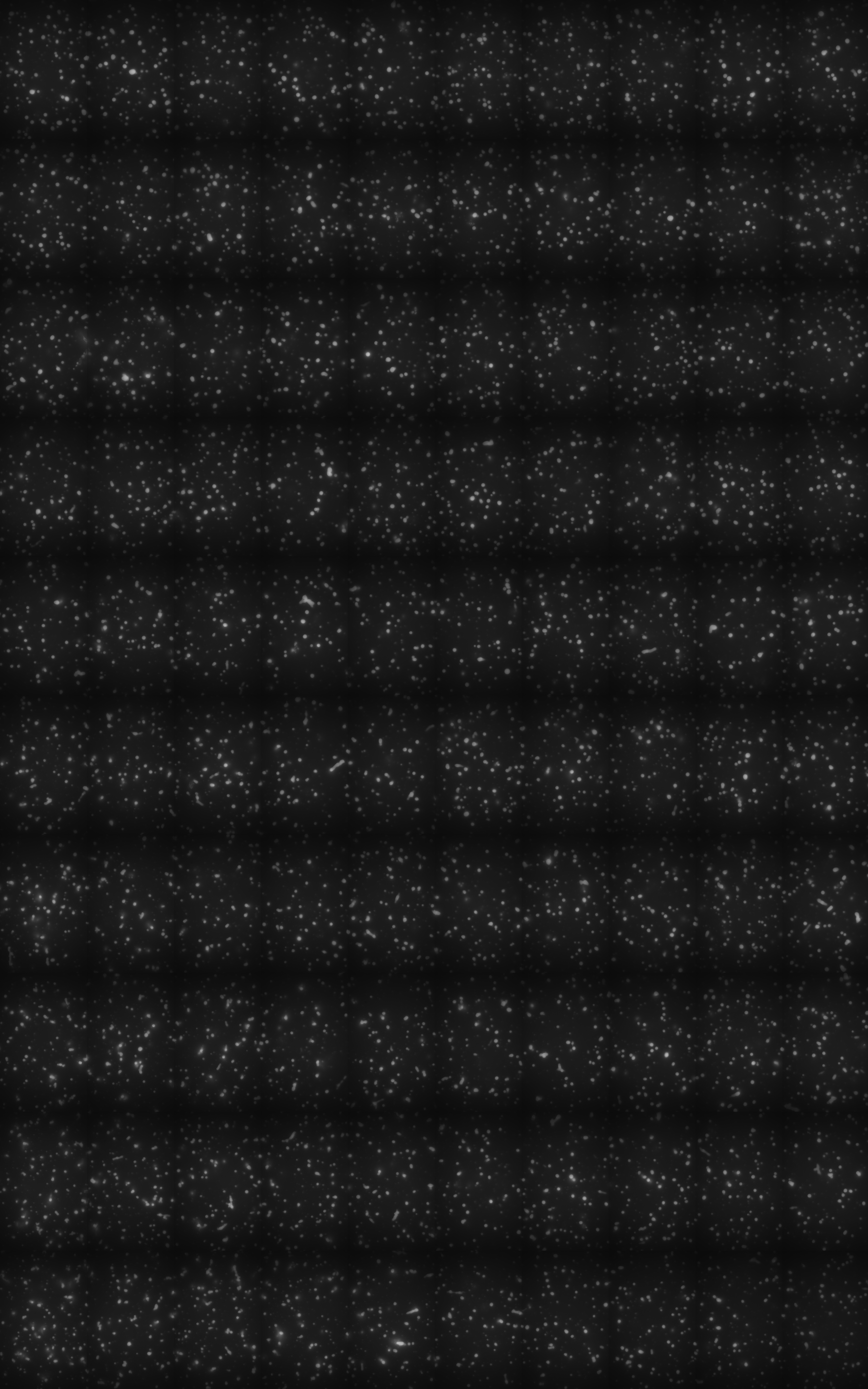

Supplement: Supplementary file 10 — Source data Fig. 8 [file 44318_2026_814_MOESM10_ESM.zip › Figure 9 A/NSP5 HP DeltaC HP/20241001_NSP5-RF-HP_DeltaC-HP/2024_10_01_25uM-NSP2-A488_18uM-NSP5-RF-HP_6uM-NSP5-RF-HP-DeltaCHP_10min-scan1.tiff]

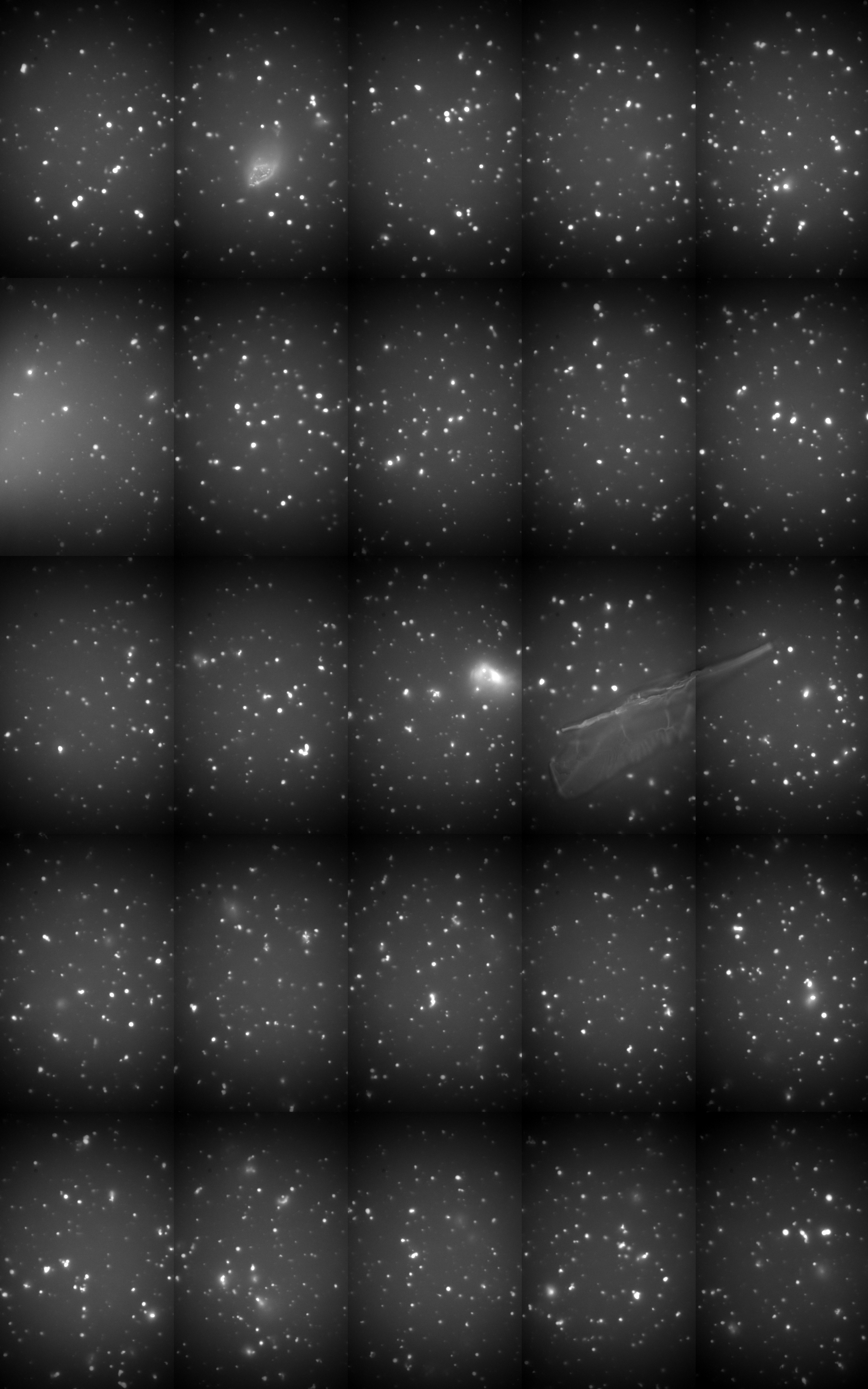

Supplement: Supplementary file 10 — Source data Fig. 8 [file 44318_2026_814_MOESM10_ESM.zip › Figure 9 A/NSP5 HP DeltaC HP/20241001_NSP5-RF-HP_DeltaC-HP/2024_10_01_25uM-NSP2-A488_25uM-NSP5-RF-HP-DeltaCHP_10min-scan.tiff]

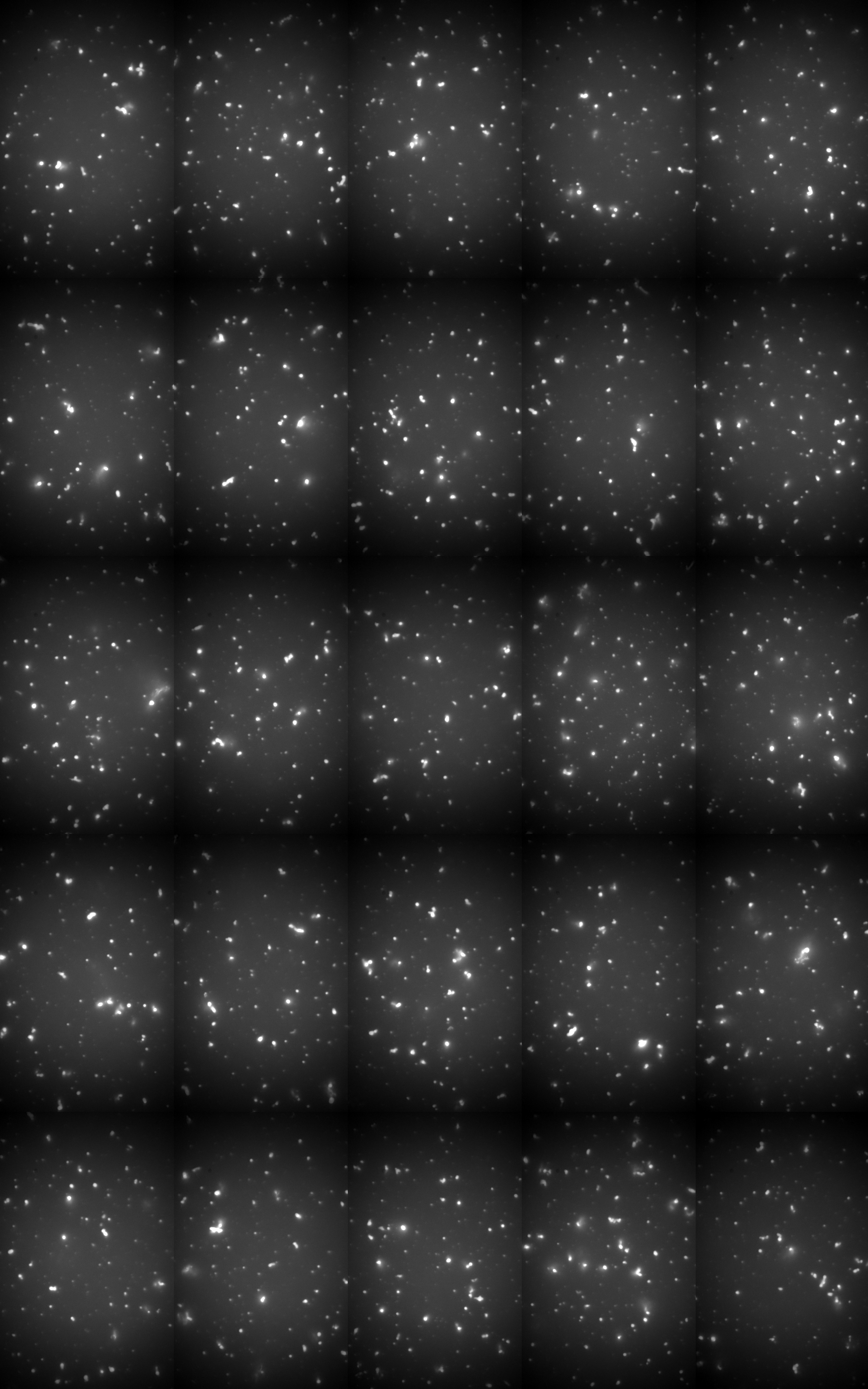

Supplement: Supplementary file 10 — Source data Fig. 8 [file 44318_2026_814_MOESM10_ESM.zip › Figure 9 A/NSP5 HP DeltaC HP/20241001_NSP5-RF-HP_DeltaC-HP/2024_10_01_25uM-NSP2-A488_6uM-NSP5-RF-HP_18uM-NSP5-RF-HP-DeltaCHP_10min-scan.tiff]

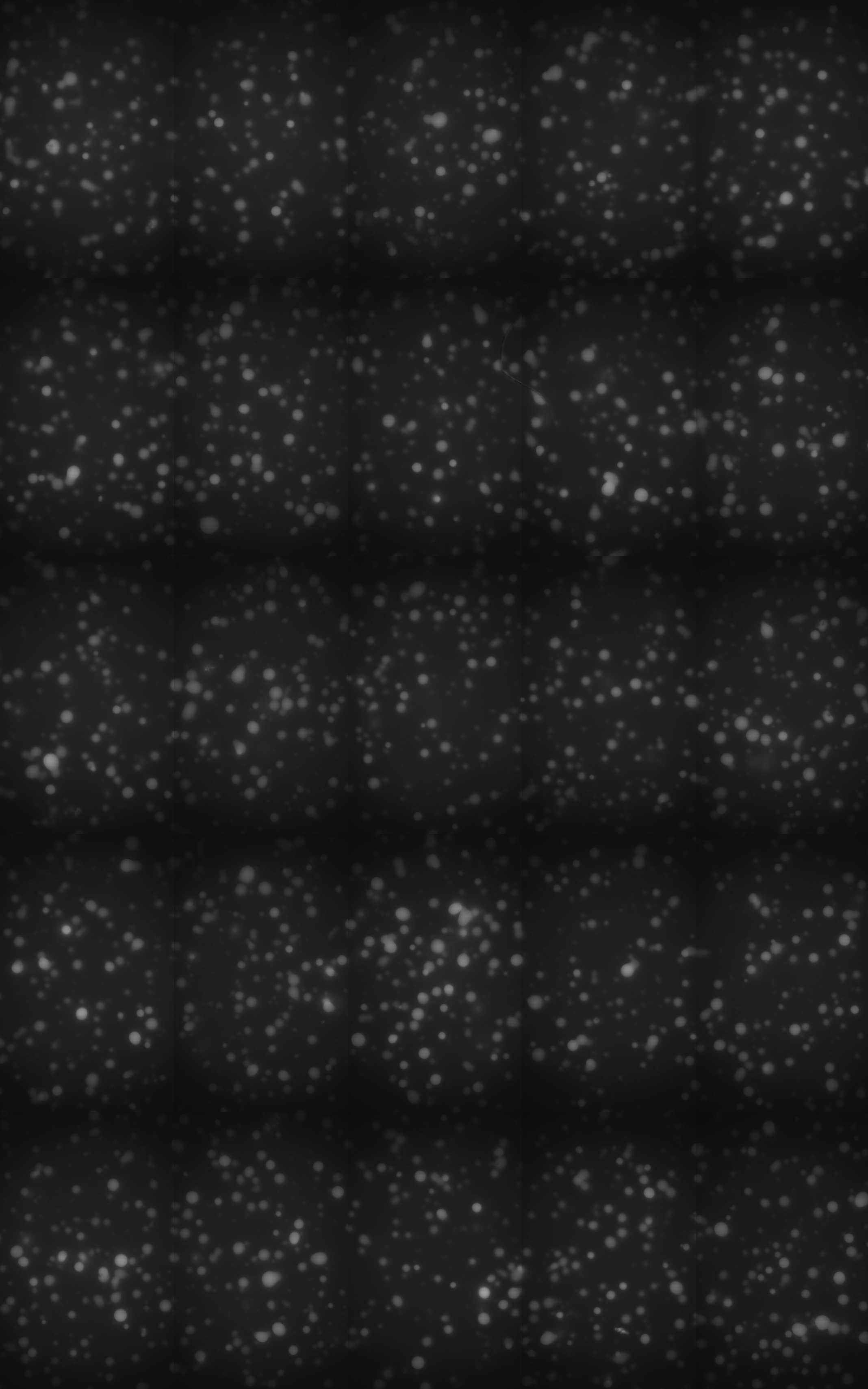

Supplement: Supplementary file 10 — Source data Fig. 8 [file 44318_2026_814_MOESM10_ESM.zip › Figure 9 A/NSP5 HP DeltaC HP/20241001_NSP5-RF-HP_DeltaC-HP/2024_10_01_25uM-NSP2-A488_25uM-NSP5-RF-HP_10min-scan1.tiff]

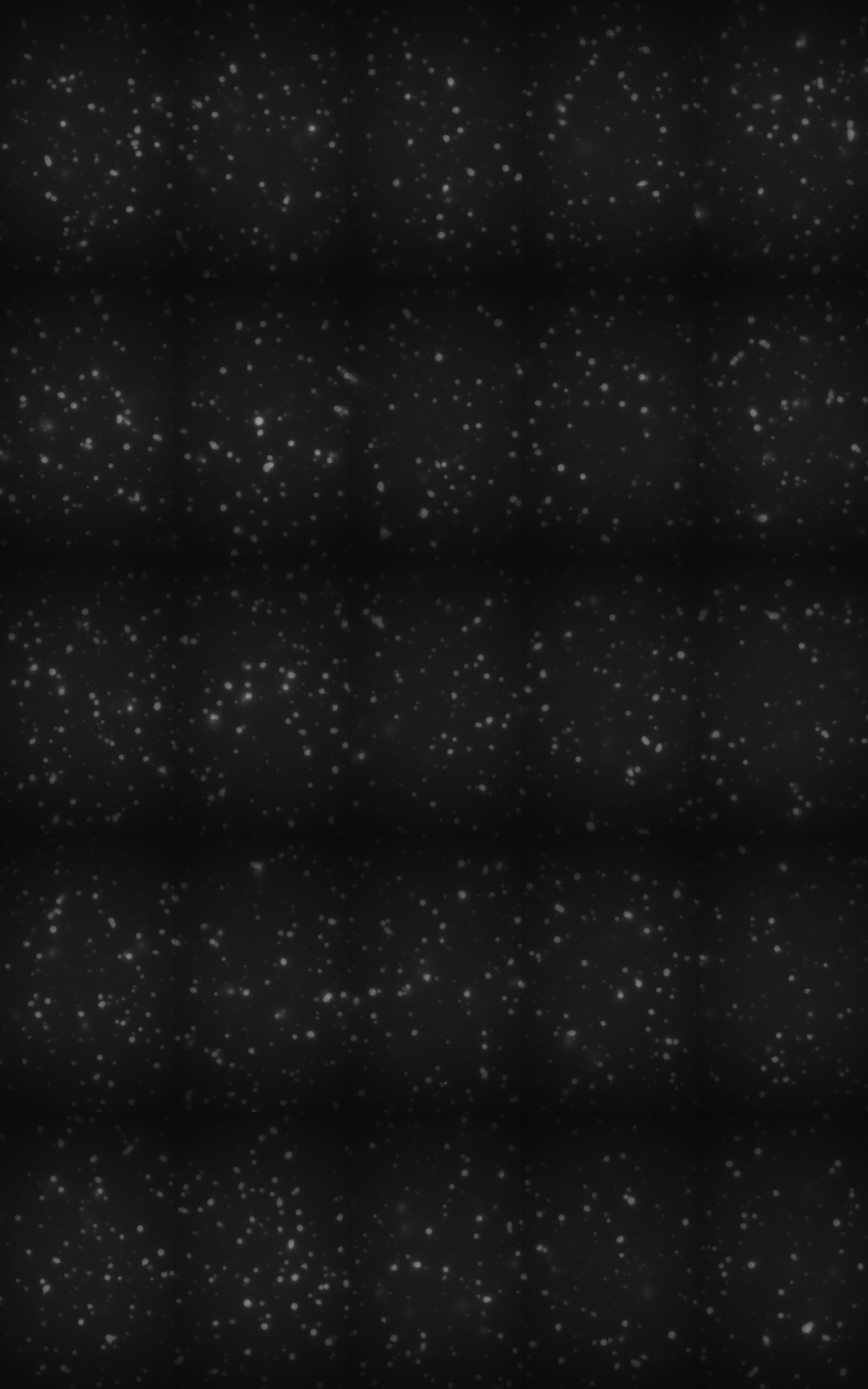

Supplement: Supplementary file 10 — Source data Fig. 8 [file 44318_2026_814_MOESM10_ESM.zip › Figure 9 A/NSP5 HP DeltaC HP/20241001_NSP5-RF-HP_DeltaC-HP/2024_10_01_25uM-NSP2-A488_12uM-NSP5-RF-HP_12uM-NSP5-RF-HP-DeltaCHP_10min-scan.tiff]

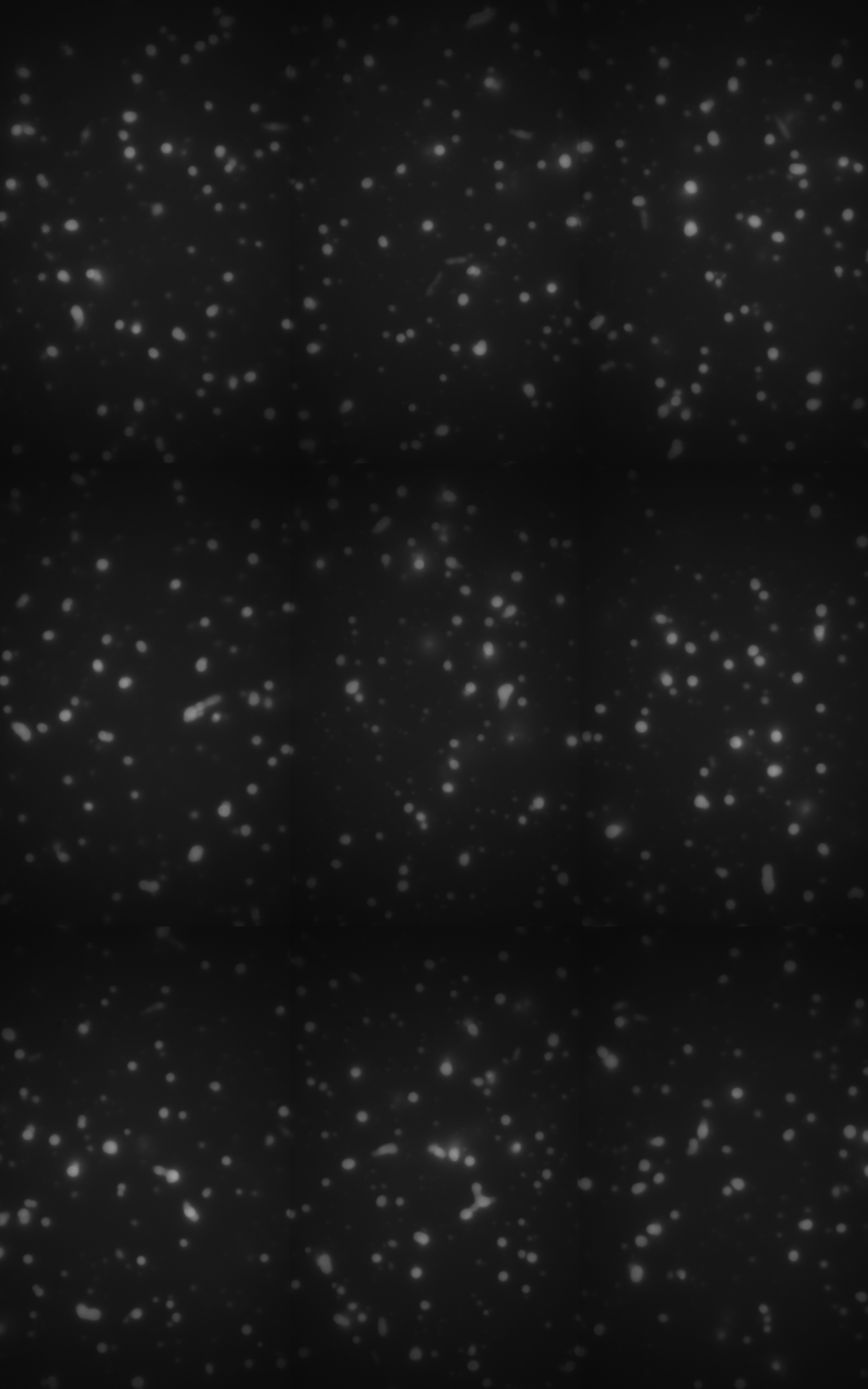

Supplement: Supplementary file 10 — Source data Fig. 8 [file 44318_2026_814_MOESM10_ESM.zip › Figure 9 A/NSP5 HP DeltaC HP/20241001_NSP5-RF-HP_DeltaC-HP/2024_10_01_25uM-NSP2-A488_18uM-NSP5-RF-HP_6uM-NSP5-RF-HP-DeltaCHP_10min-scan.tiff]

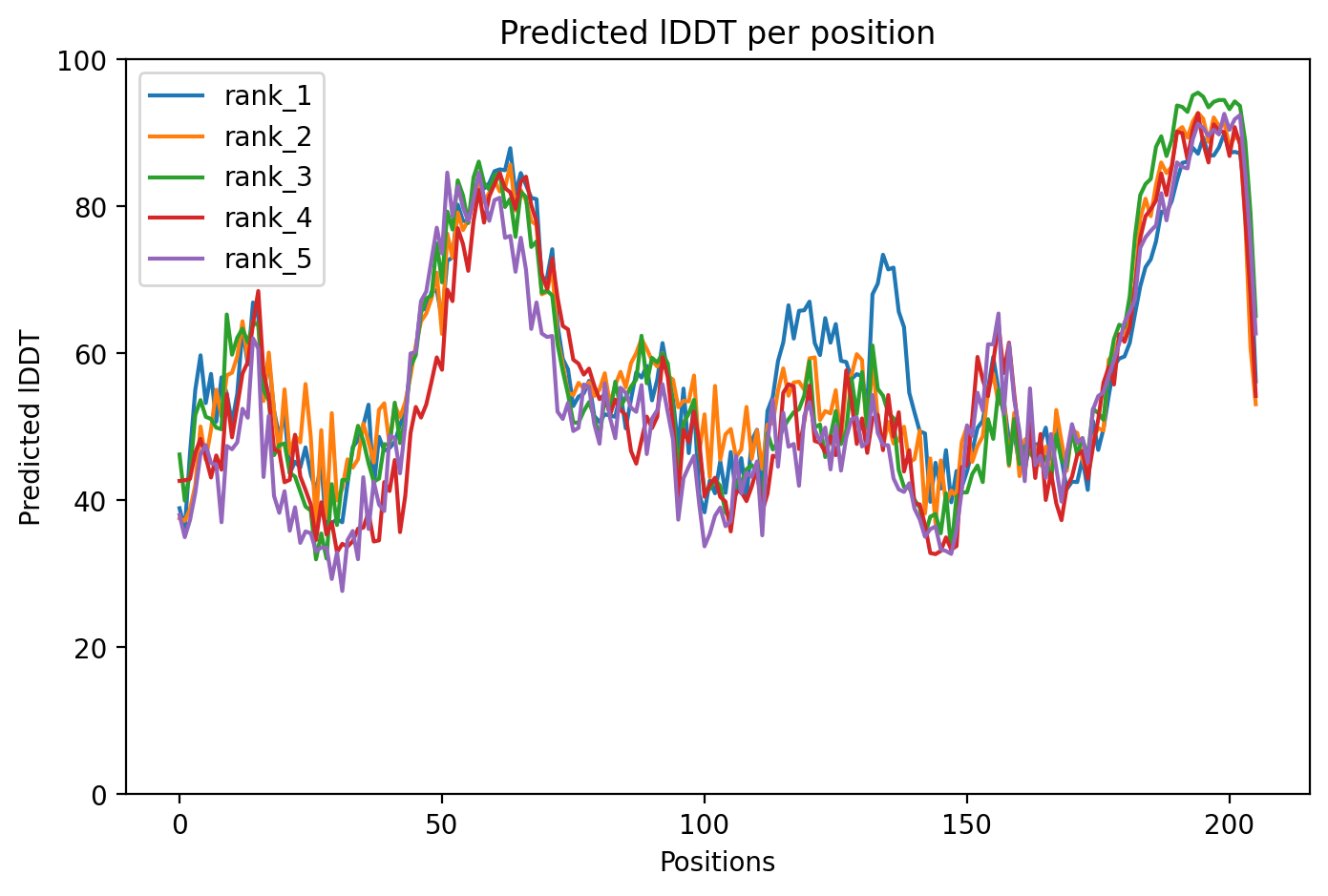

Supplement: Supplementary file 11 — Source data Fig. 9 [file 44318_2026_814_MOESM11_ESM.zip › Figure_7/Figure 7A/NSP5 SA11 HP/NSP5SA11HP_6fef1_plddt.png]

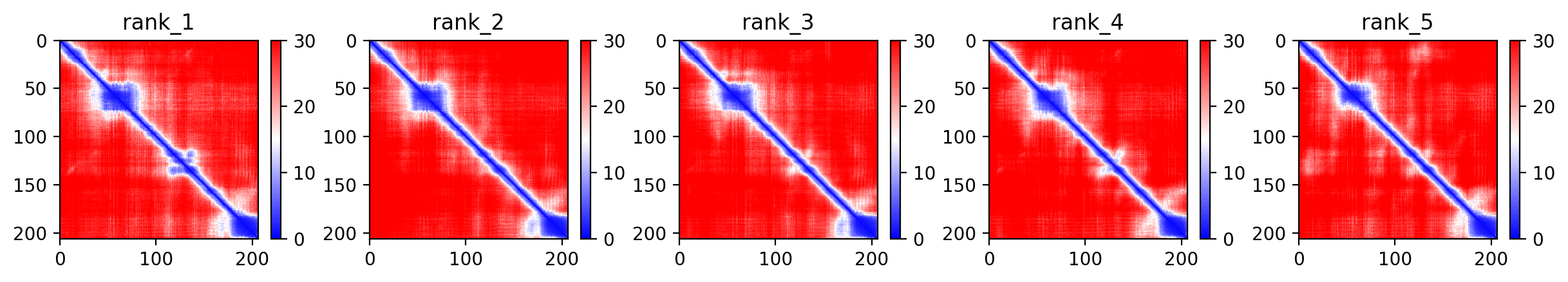

Supplement: Supplementary file 11 — Source data Fig. 9 [file 44318_2026_814_MOESM11_ESM.zip › Figure_7/Figure 7A/NSP5 SA11 HP/NSP5SA11HP_6fef1_pae.png]

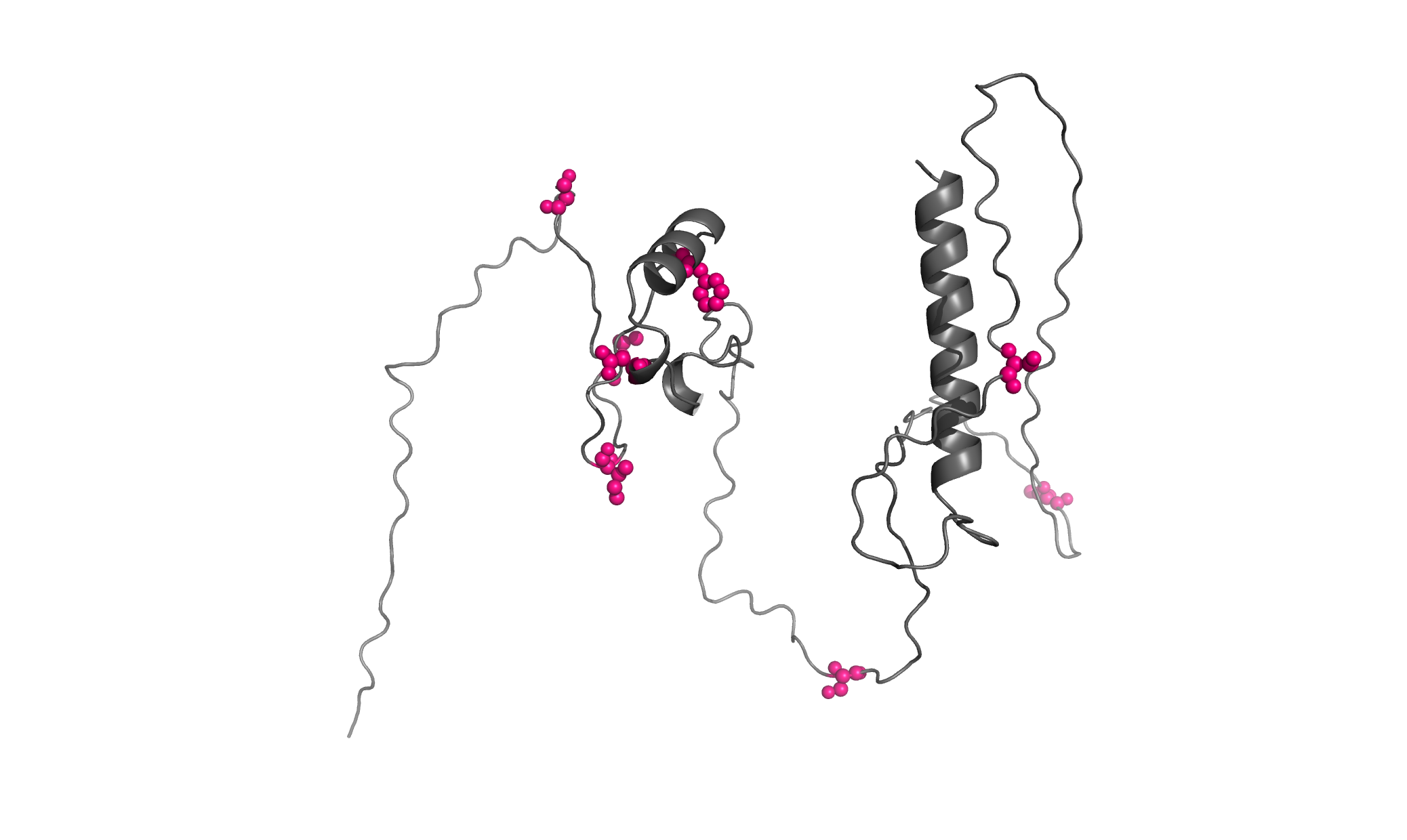

Supplement: Supplementary file 11 — Source data Fig. 9 [file 44318_2026_814_MOESM11_ESM.zip › Figure_7/Figure 7A/NSP5 SA11 HP/NSP5HP_dpi300.png]

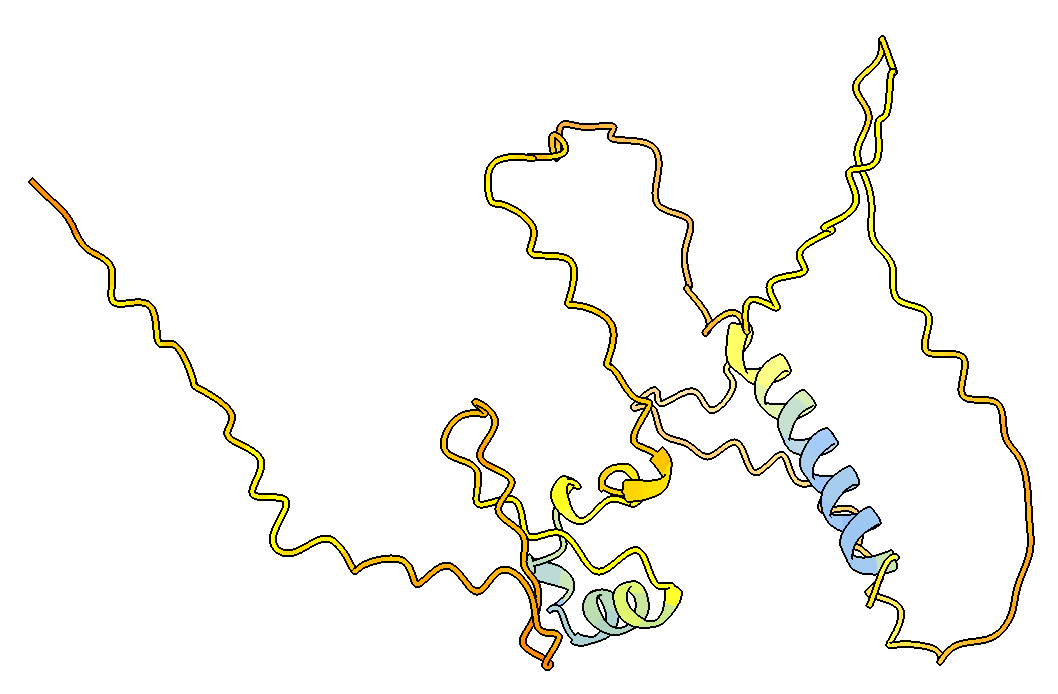

Supplement: Supplementary file 11 — Source data Fig. 9 [file 44318_2026_814_MOESM11_ESM.zip › Figure_7/Figure 7A/NSP5 SA11 HP/Screenshot 2025-04-20 at 11.22.39.png]

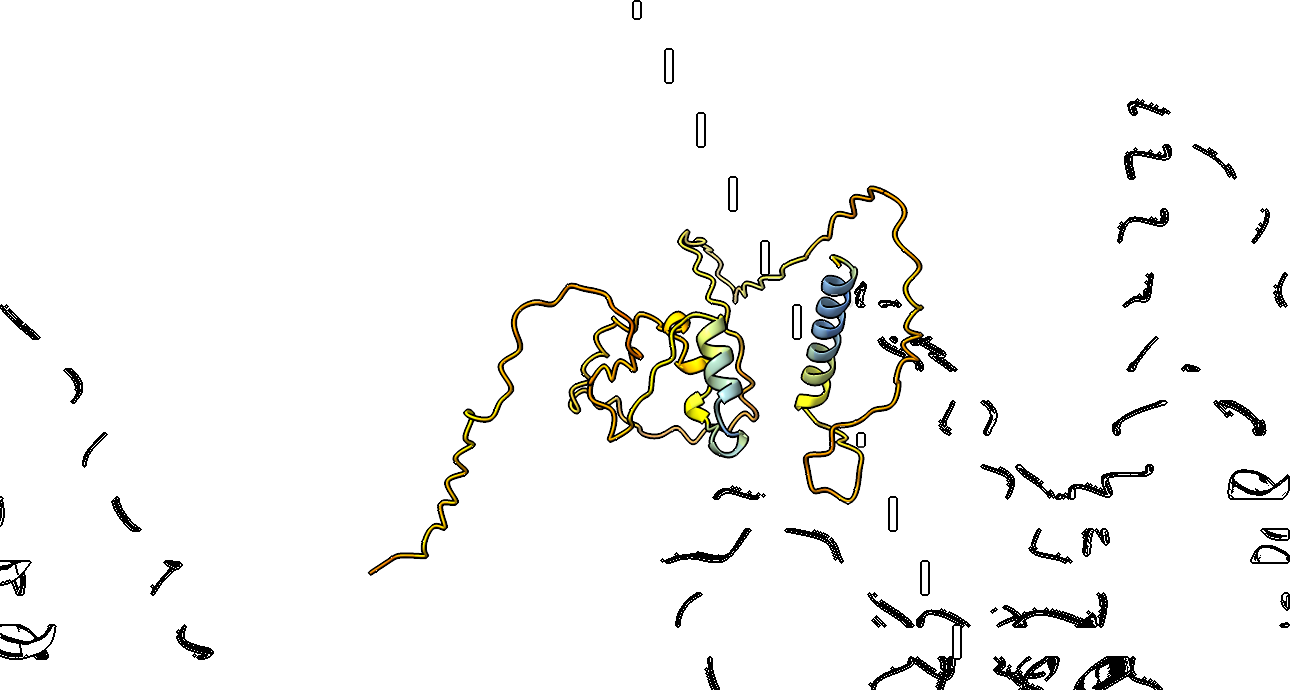

Supplement: Supplementary file 11 — Source data Fig. 9 [file 44318_2026_814_MOESM11_ESM.zip › Figure_7/Figure 7A/NSP5 SA11 HP/NSP5SA11HP_plddt.tif]

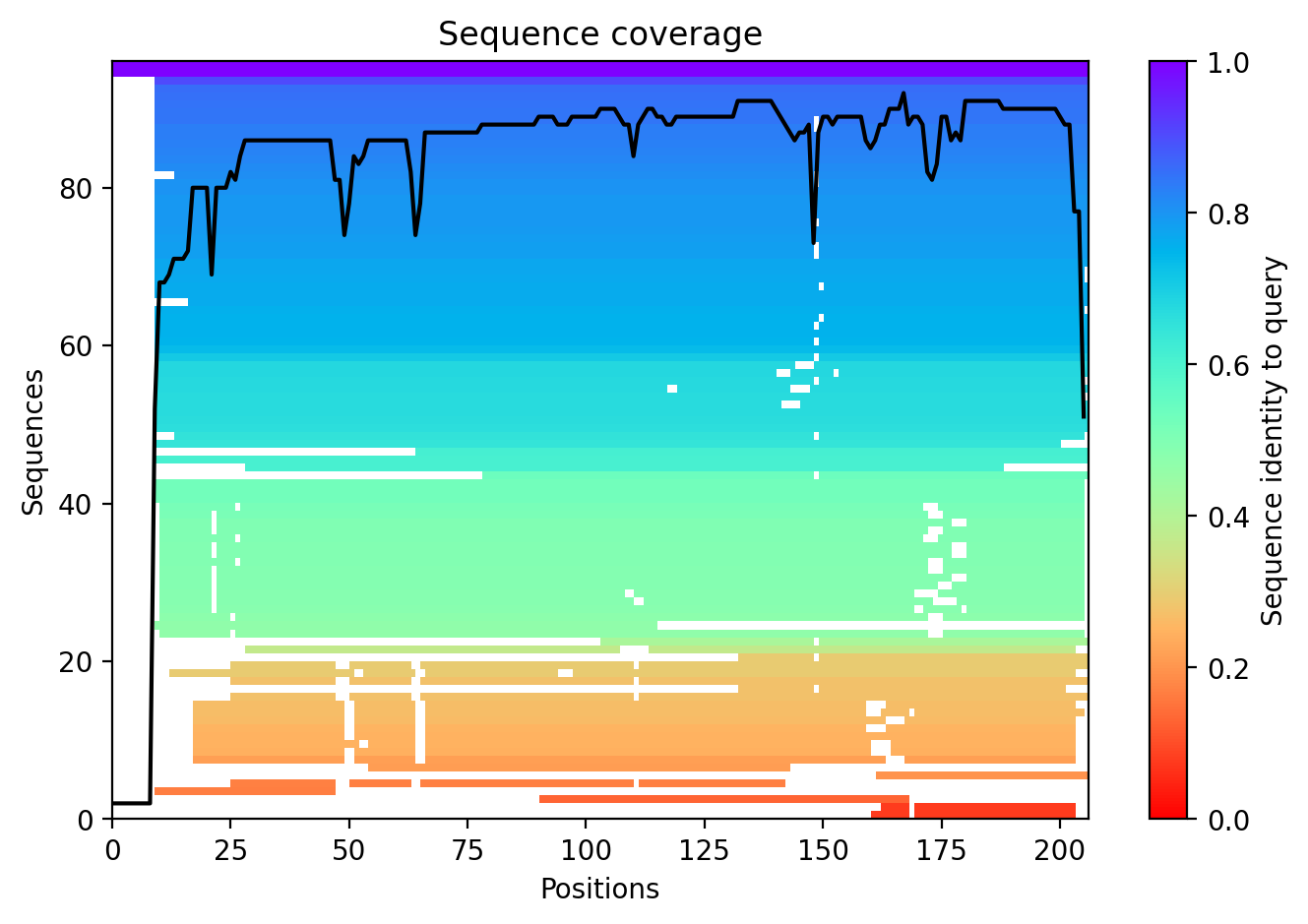

Supplement: Supplementary file 11 — Source data Fig. 9 [file 44318_2026_814_MOESM11_ESM.zip › Figure_7/Figure 7A/NSP5 SA11 HP/NSP5SA11HP_6fef1_coverage.png]

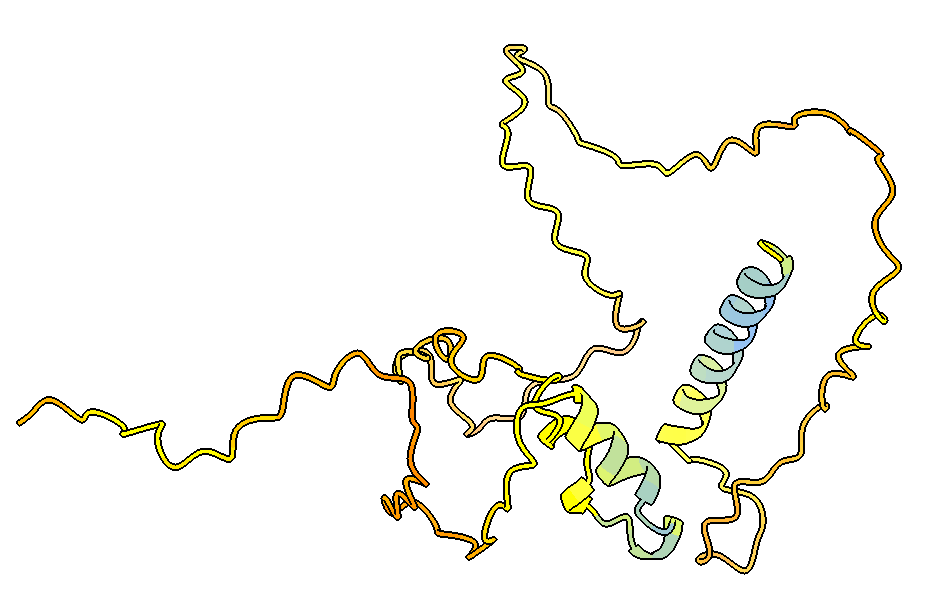

Supplement: Supplementary file 11 — Source data Fig. 9 [file 44318_2026_814_MOESM11_ESM.zip › Figure_7/Figure 7A/NSP5 SA11/Screenshot 2025-04-20 at 11.21.13.png]

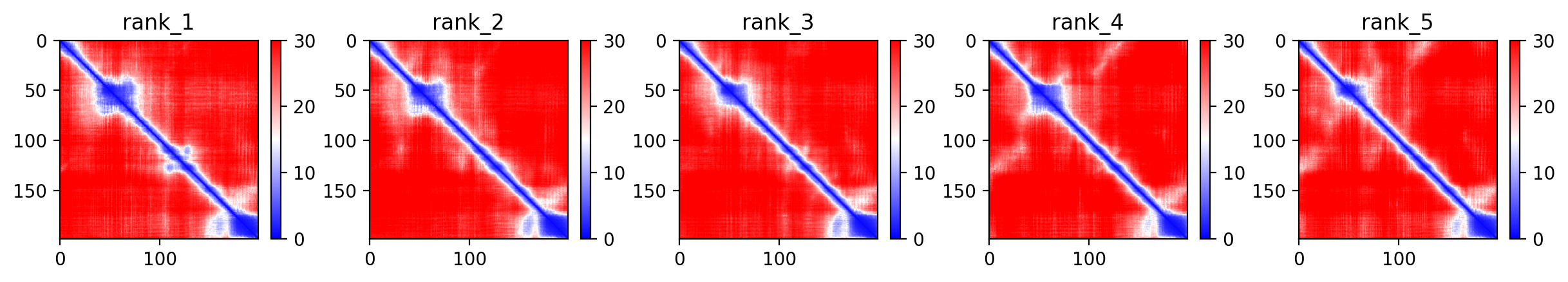

Supplement: Supplementary file 11 — Source data Fig. 9 [file 44318_2026_814_MOESM11_ESM.zip › Figure_7/Figure 7A/NSP5 SA11/NSP5SA11_d9b1a_pae.png]

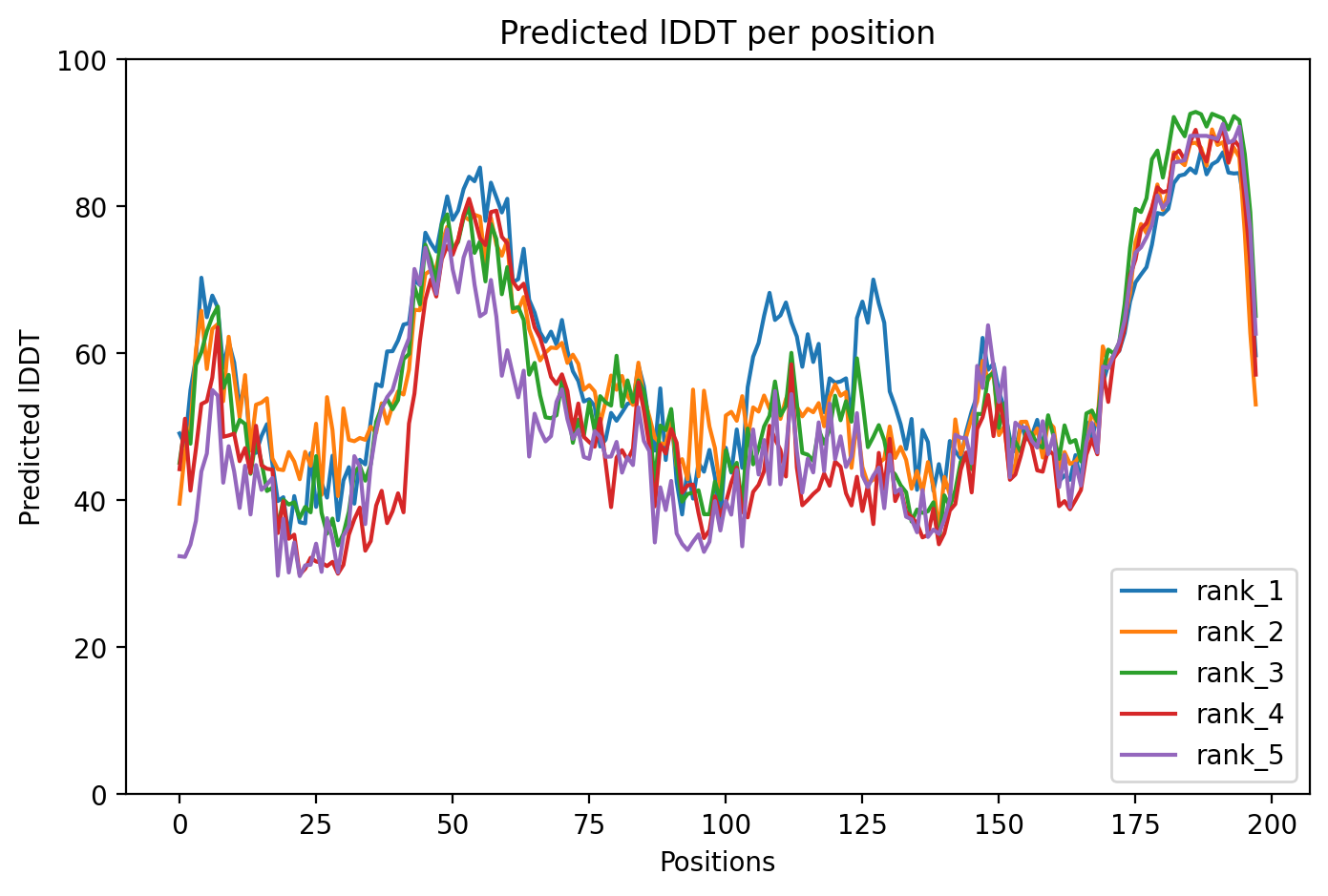

Supplement: Supplementary file 11 — Source data Fig. 9 [file 44318_2026_814_MOESM11_ESM.zip › Figure_7/Figure 7A/NSP5 SA11/NSP5SA11_d9b1a_plddt.png]

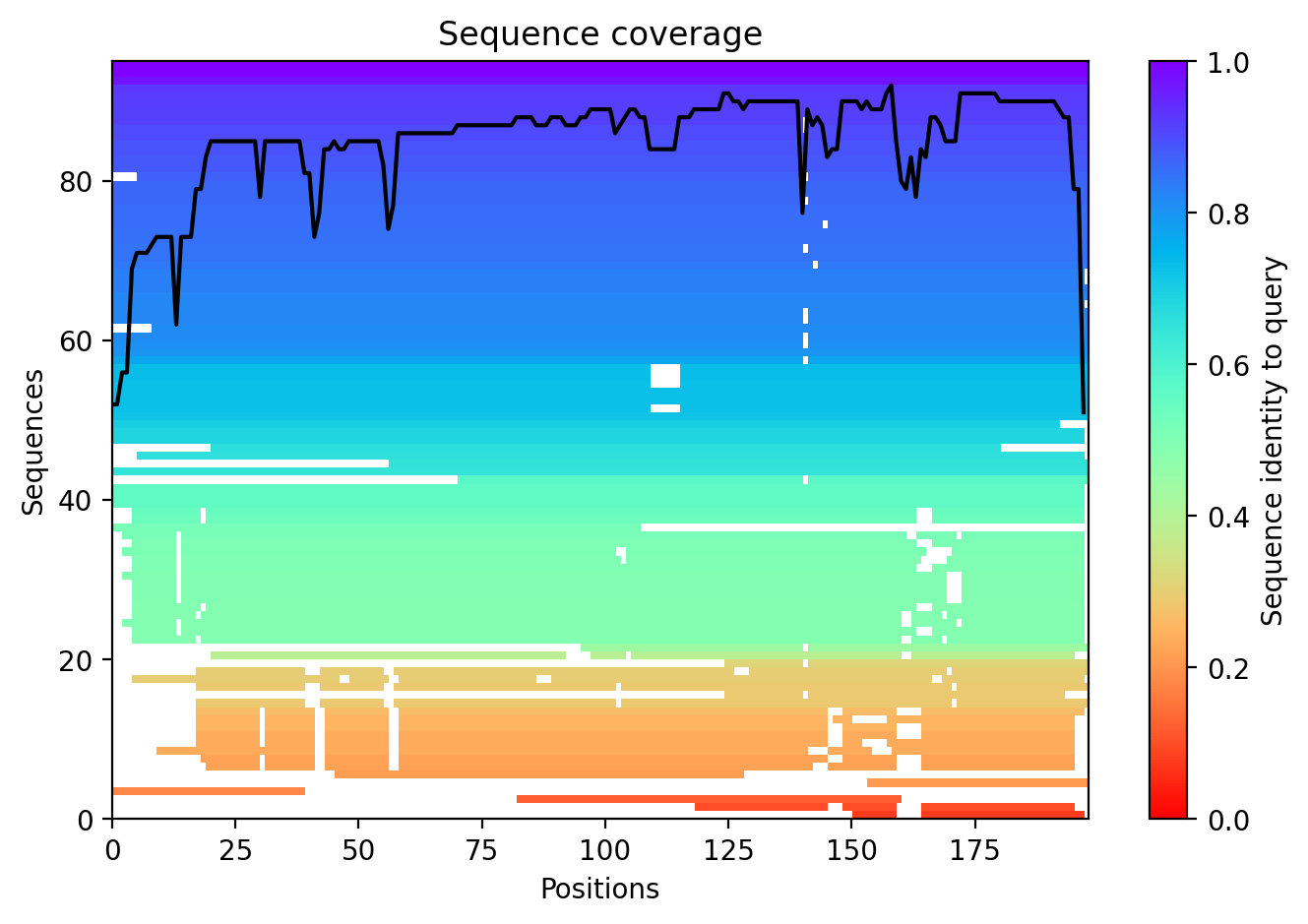

Supplement: Supplementary file 11 — Source data Fig. 9 [file 44318_2026_814_MOESM11_ESM.zip › Figure_7/Figure 7A/NSP5 SA11/NSP5SA11_d9b1a_coverage.png]

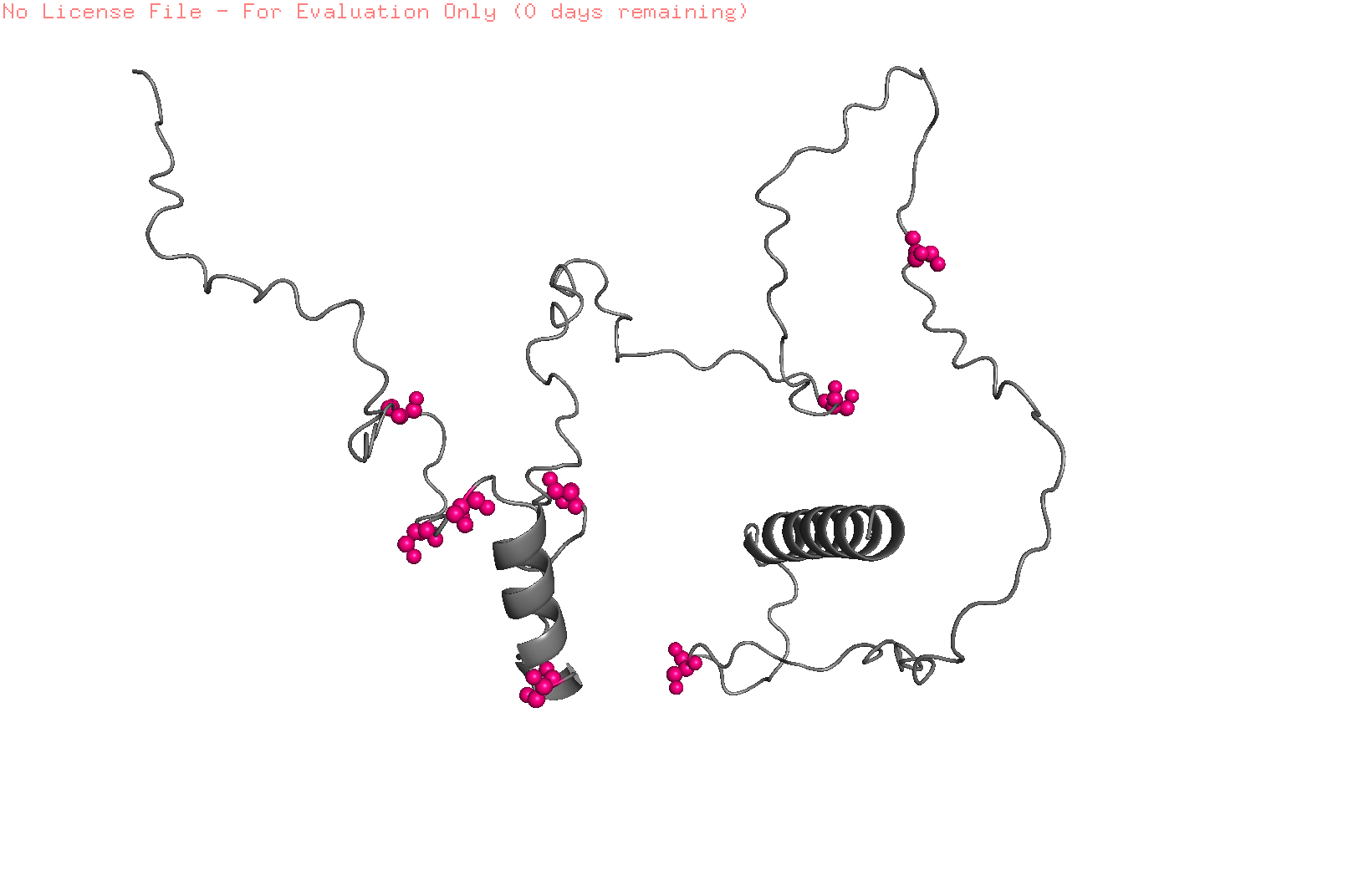

Supplement: Supplementary file 11 — Source data Fig. 9 [file 44318_2026_814_MOESM11_ESM.zip › Figure_7/Figure 7A/NSP5 SA11/NSP5SA11_hotpink_new.png]

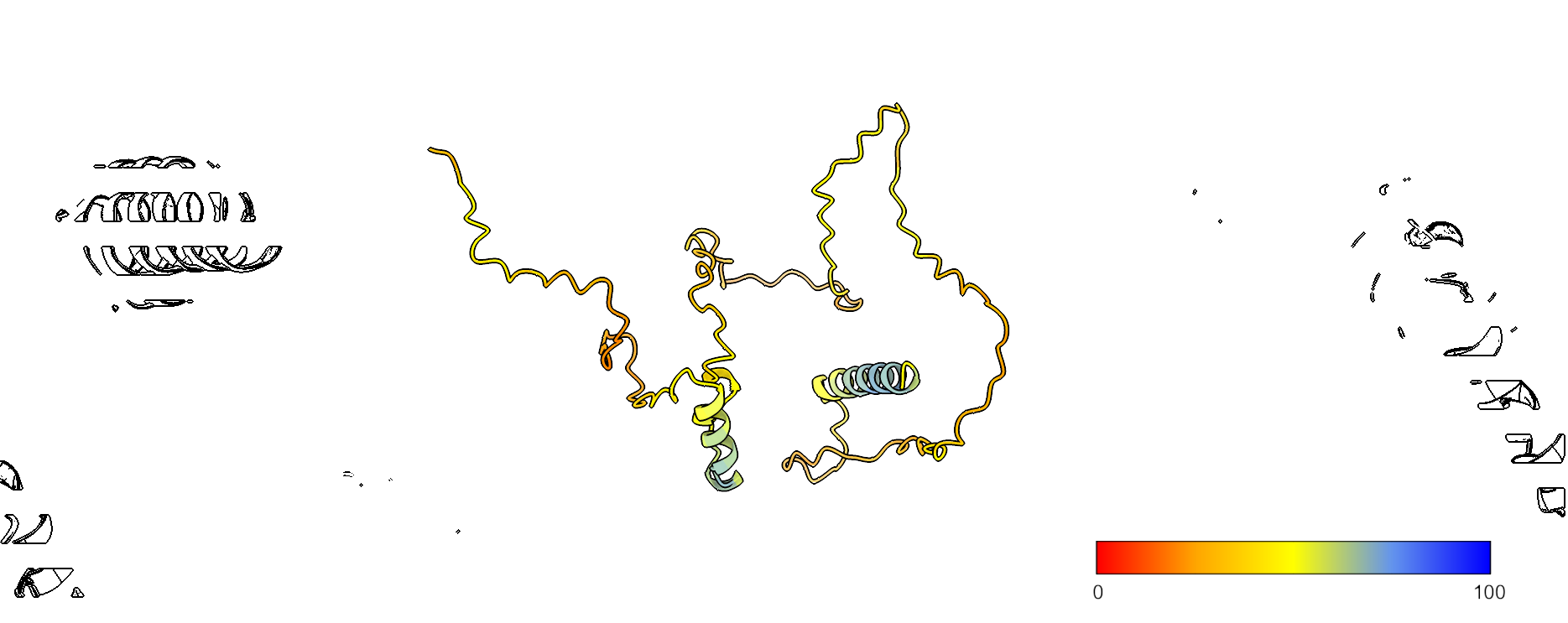

Supplement: Supplementary file 11 — Source data Fig. 9 [file 44318_2026_814_MOESM11_ESM.zip › Figure_7/Figure 7A/NSP5 SA11/NSP5SA11_plDDT.tif]

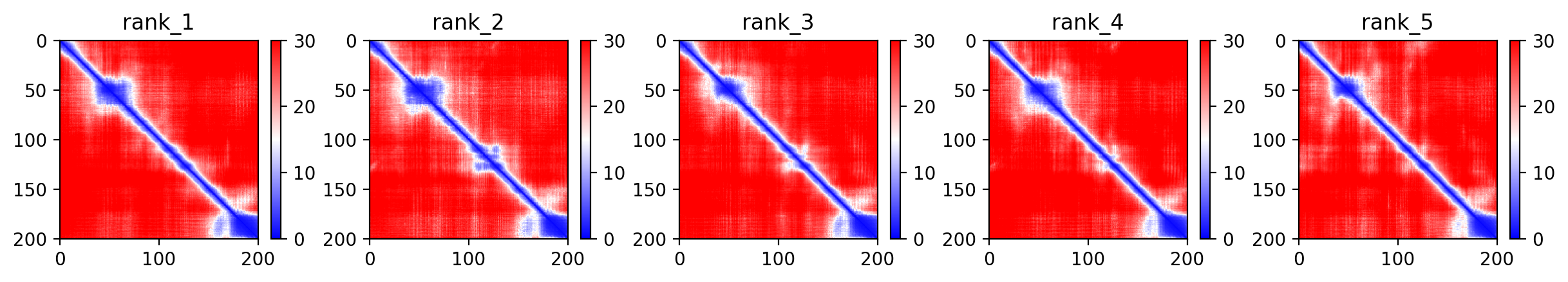

Supplement: Supplementary file 11 — Source data Fig. 9 [file 44318_2026_814_MOESM11_ESM.zip › Figure_7/Figure 7A/SClow/C2S_c9c96_pae.png]

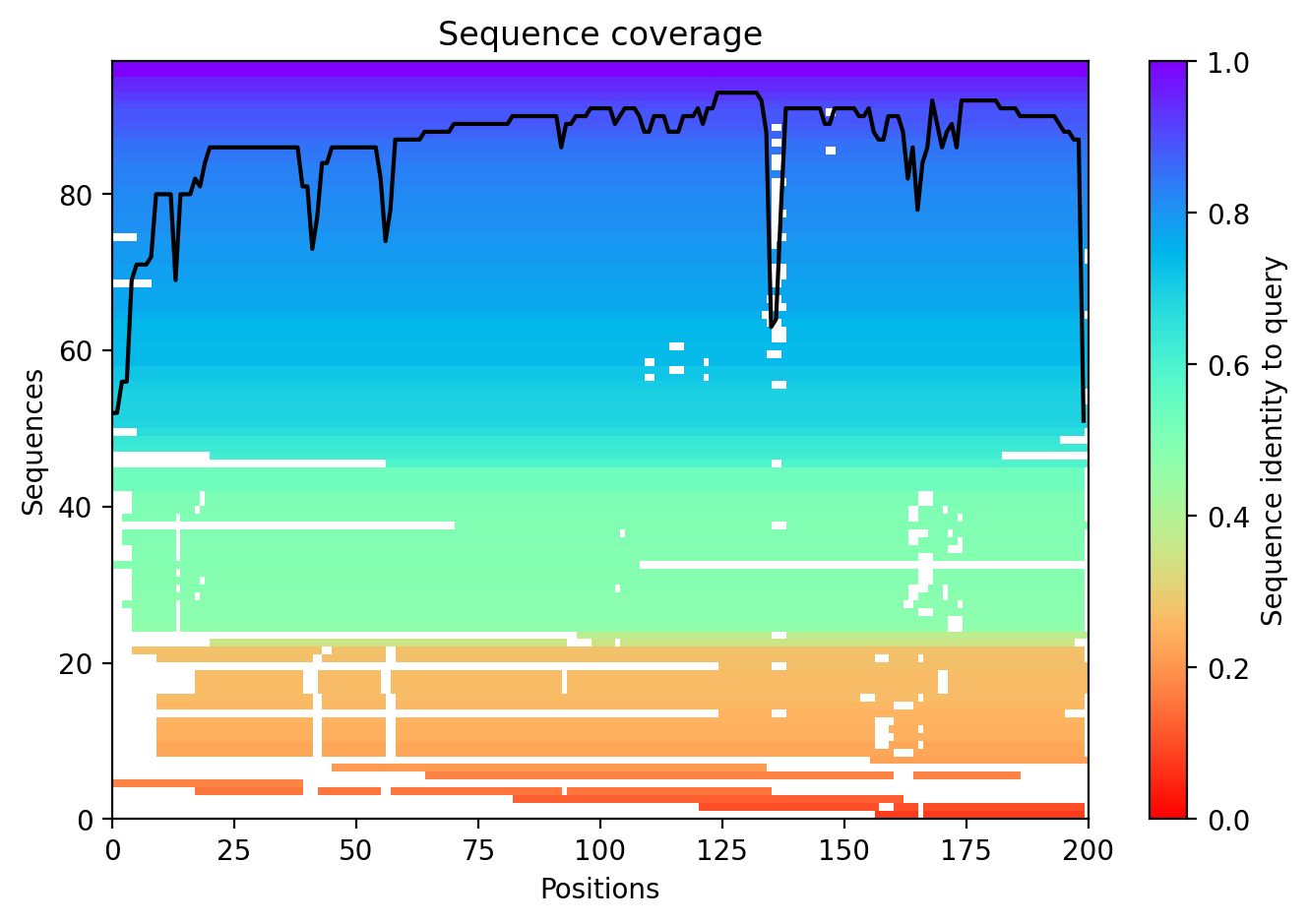

Supplement: Supplementary file 11 — Source data Fig. 9 [file 44318_2026_814_MOESM11_ESM.zip › Figure_7/Figure 7A/SClow/C2S_c9c96_coverage.png]

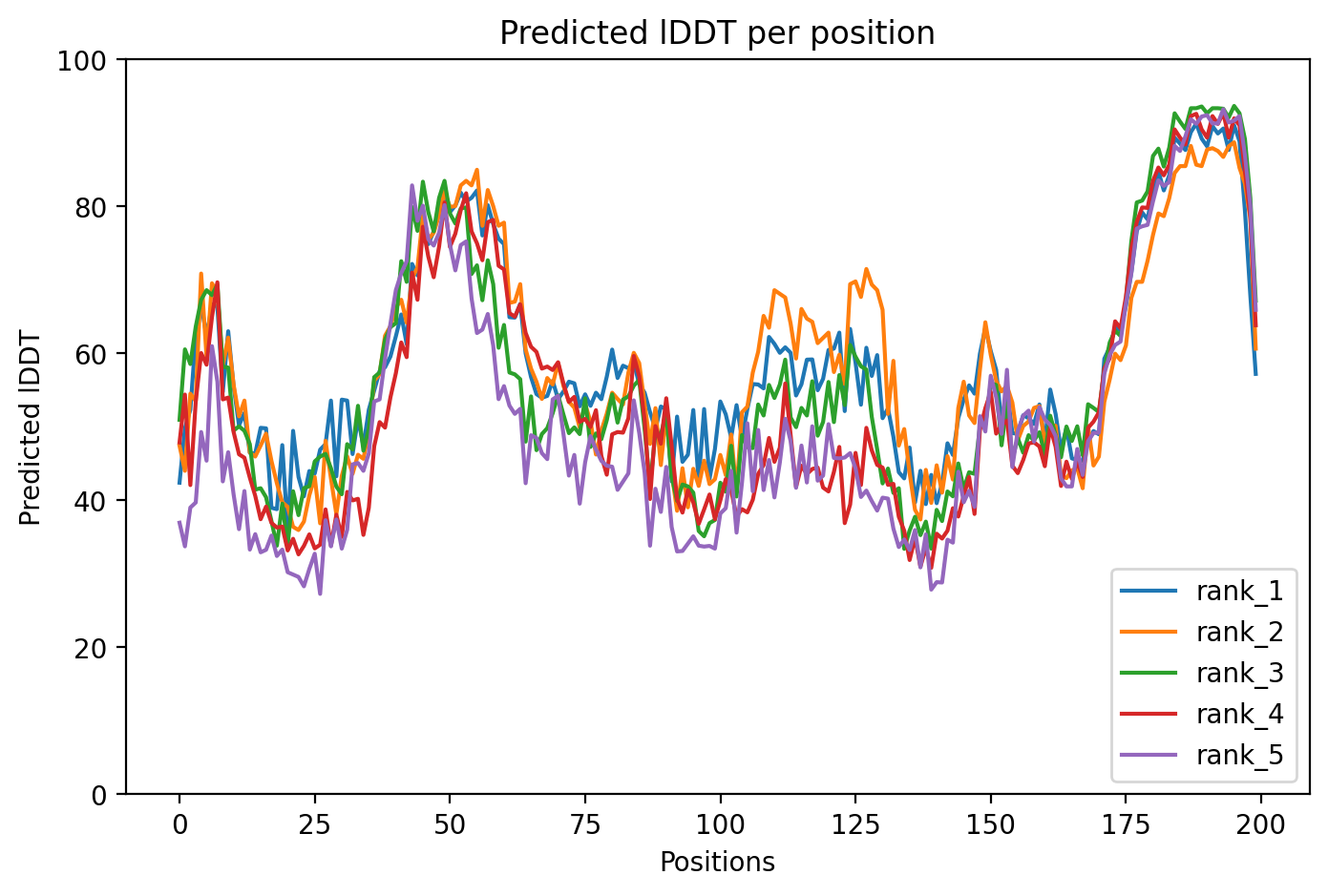

Supplement: Supplementary file 11 — Source data Fig. 9 [file 44318_2026_814_MOESM11_ESM.zip › Figure_7/Figure 7A/SClow/C2S_c9c96_plddt.png]

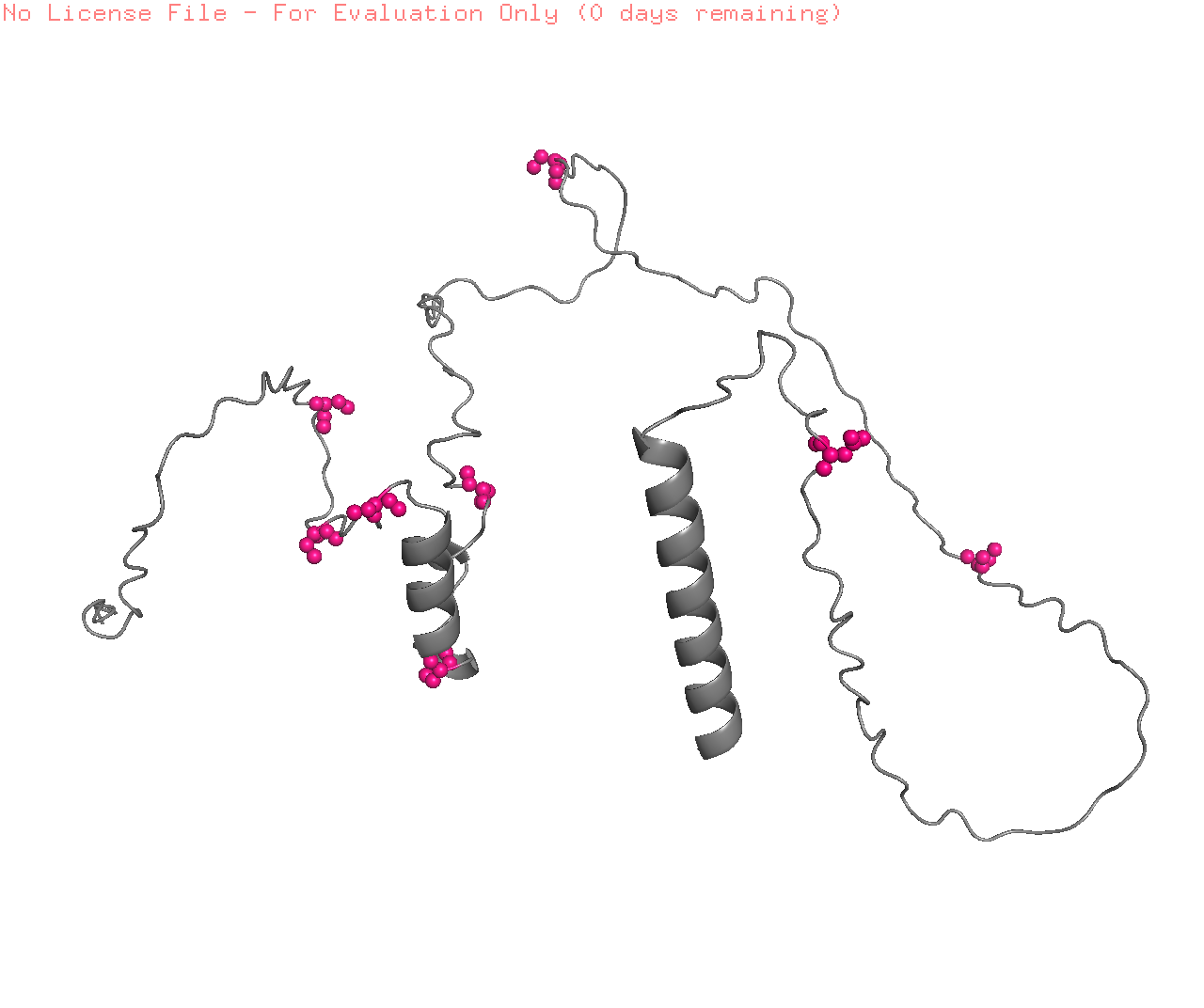

Supplement: Supplementary file 11 — Source data Fig. 9 [file 44318_2026_814_MOESM11_ESM.zip › Figure_7/Figure 7A/SClow/C2S-hotpink-new.png]

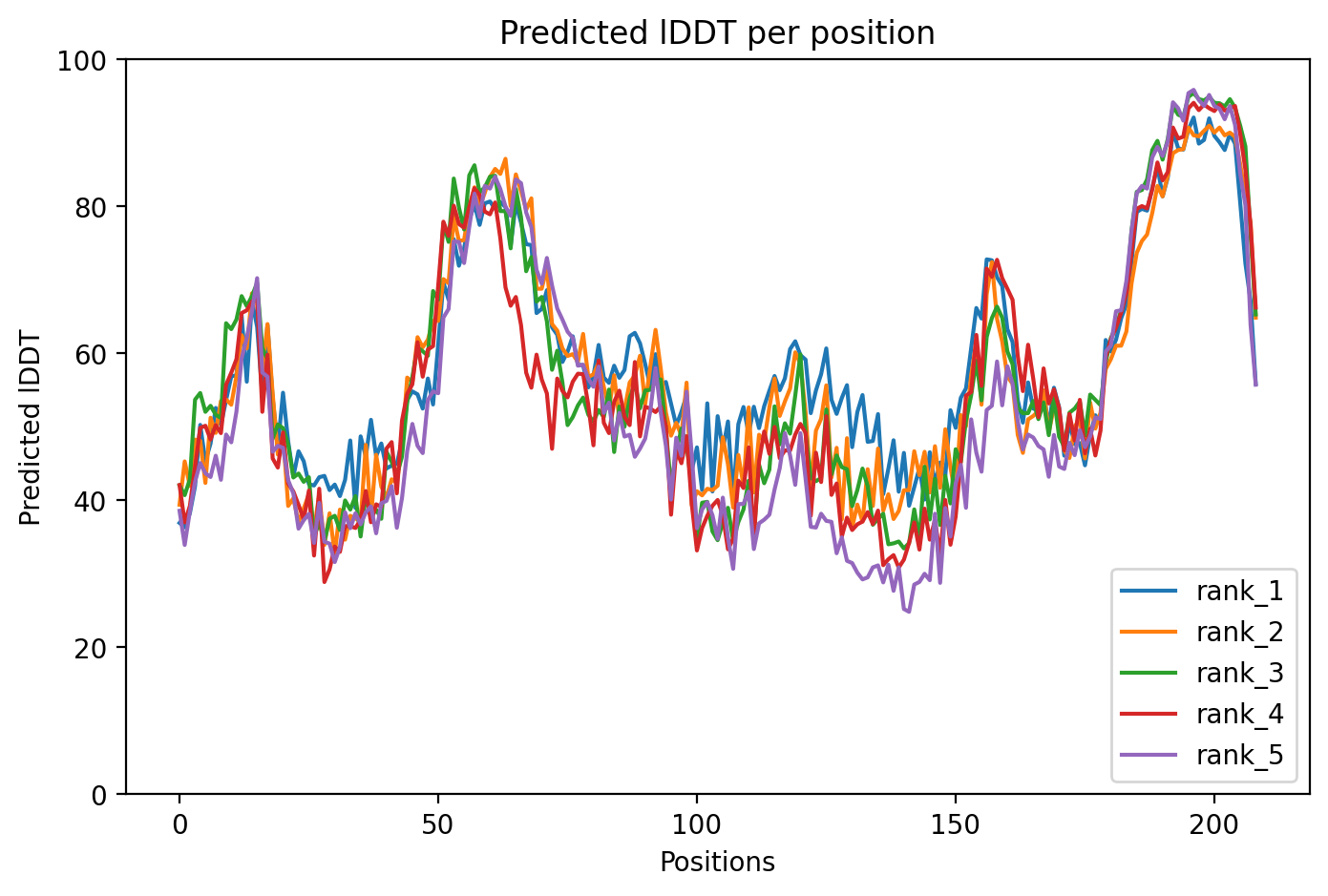

Supplement: Supplementary file 11 — Source data Fig. 9 [file 44318_2026_814_MOESM11_ESM.zip › Figure_7/Figure 7A/SClow HP/SClowHP_9a3a3_plddt.png]

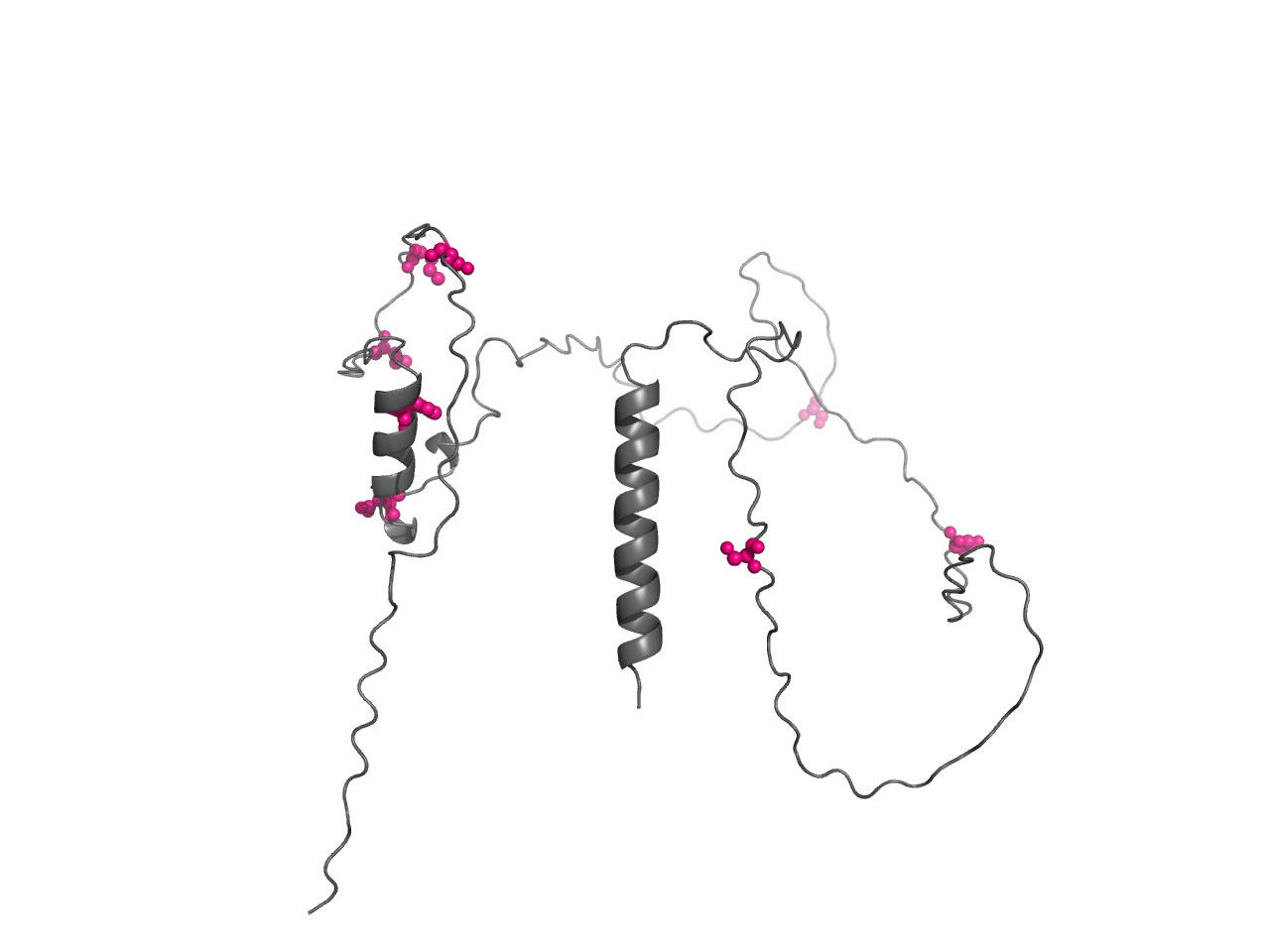

Supplement: Supplementary file 11 — Source data Fig. 9 [file 44318_2026_814_MOESM11_ESM.zip › Figure_7/Figure 7A/SClow HP/SClowHP_dpi300.png]

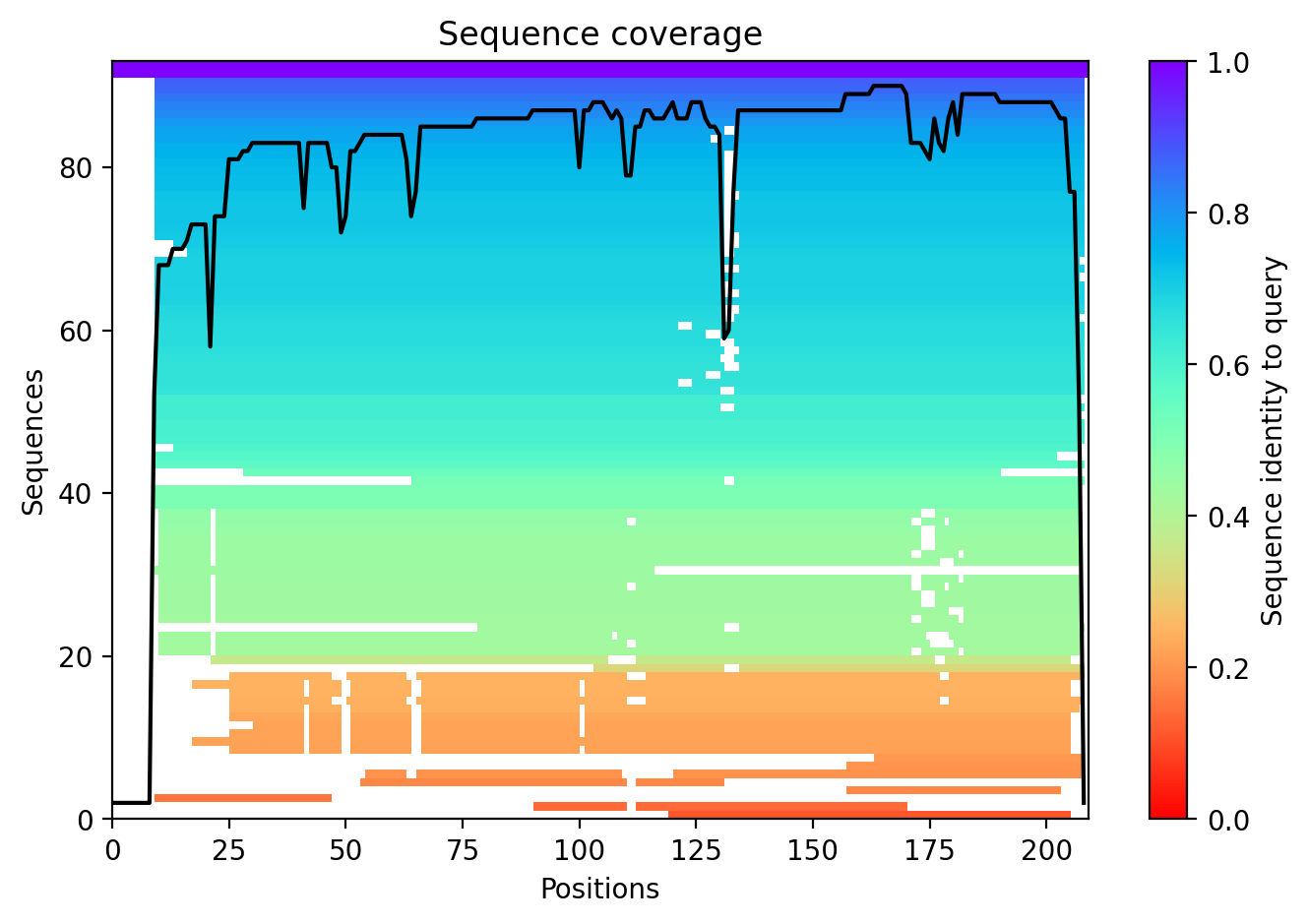

Supplement: Supplementary file 11 — Source data Fig. 9 [file 44318_2026_814_MOESM11_ESM.zip › Figure_7/Figure 7A/SClow HP/SClowHP_9a3a3_coverage.png]

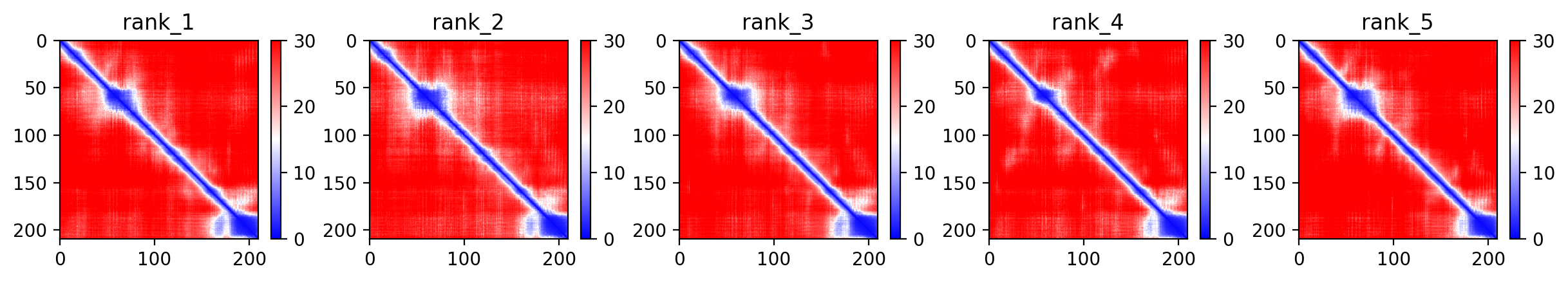

Supplement: Supplementary file 11 — Source data Fig. 9 [file 44318_2026_814_MOESM11_ESM.zip › Figure_7/Figure 7A/SClow HP/SClowHP_9a3a3_pae.png]

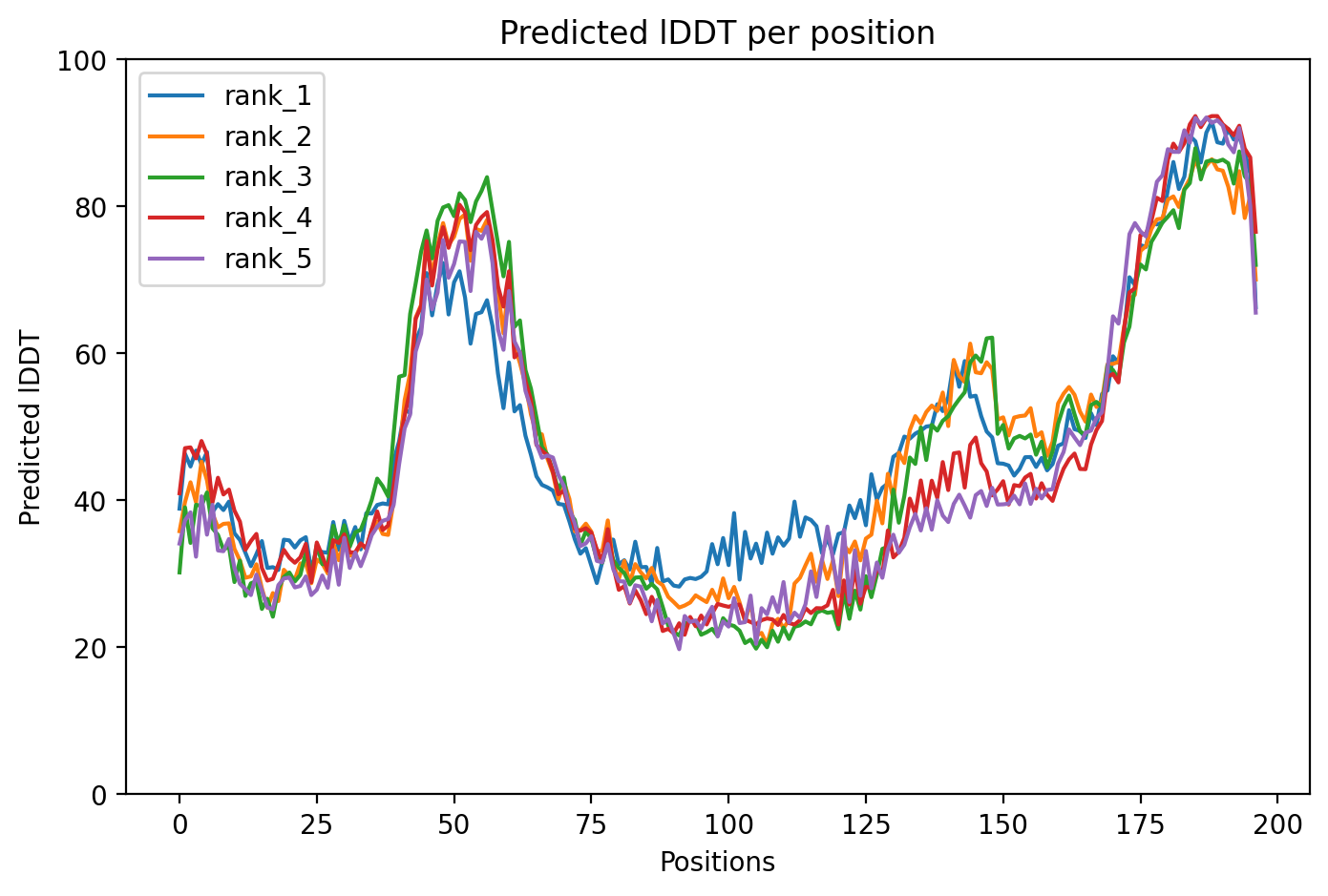

Supplement: Supplementary file 11 — Source data Fig. 9 [file 44318_2026_814_MOESM11_ESM.zip › Figure_7/Figure 7A/NSP5 RF HP/NSP5RFHPmonomer_54e43/NSP5RFHPmonomer_54e43_plddt.png]

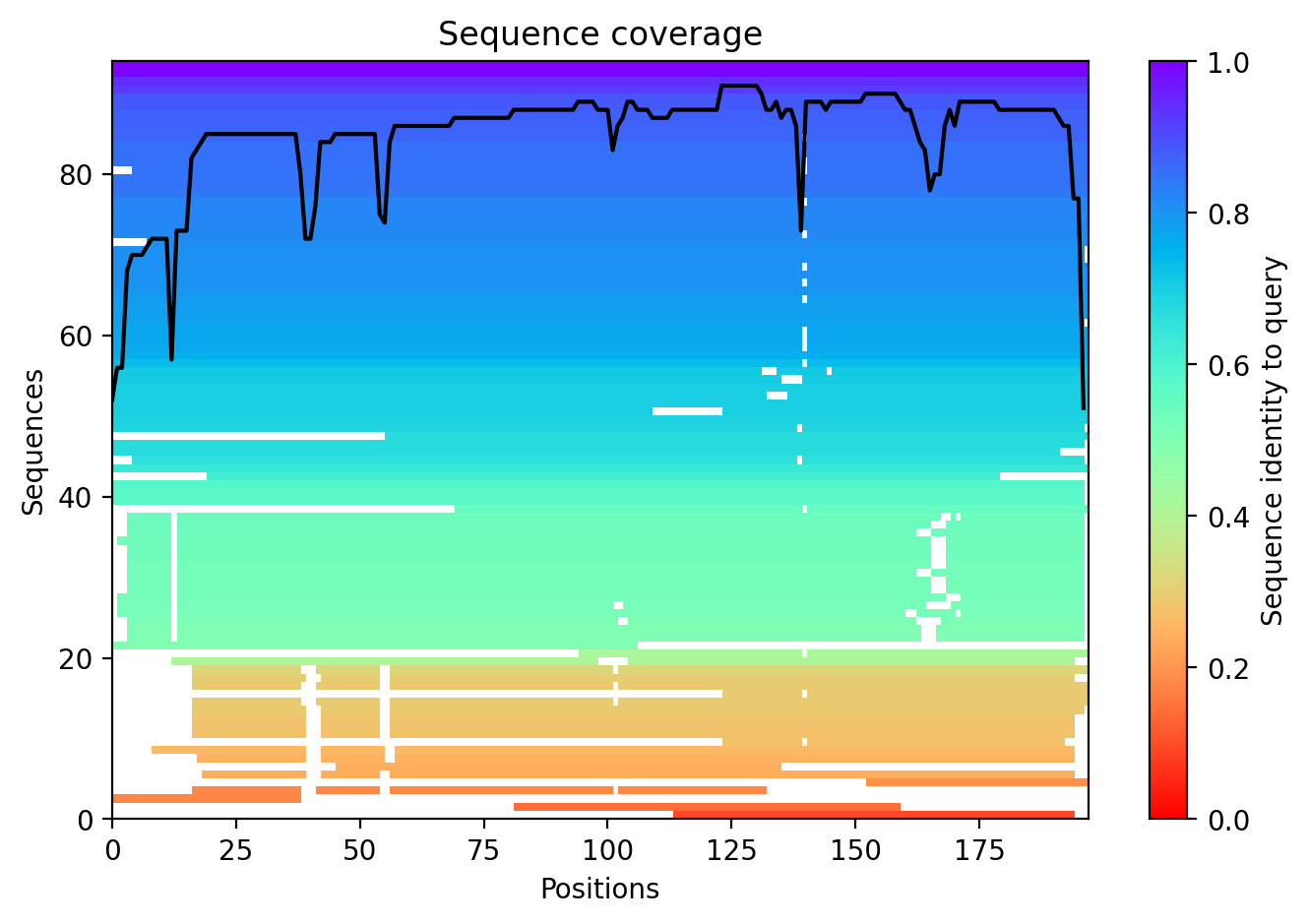

Supplement: Supplementary file 11 — Source data Fig. 9 [file 44318_2026_814_MOESM11_ESM.zip › Figure_7/Figure 7A/NSP5 RF HP/NSP5RFHPmonomer_54e43/NSP5RFHPmonomer_54e43_coverage.png]

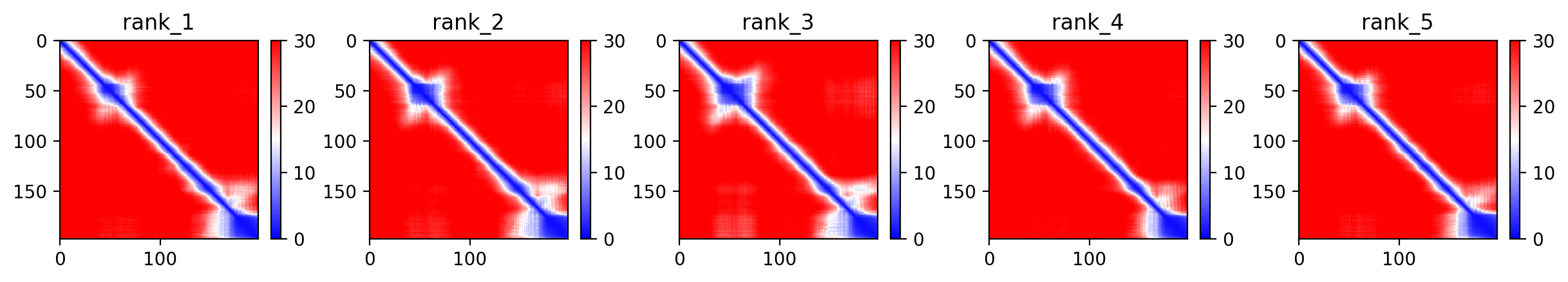

Supplement: Supplementary file 11 — Source data Fig. 9 [file 44318_2026_814_MOESM11_ESM.zip › Figure_7/Figure 7A/NSP5 RF HP/NSP5RFHPmonomer_54e43/NSP5RFHPmonomer_54e43_pae.png]
