# Supplementary material for: Phosphorylation tunes strain-specific protein condensation during rotavirus replication organelle assembly
Source: EMBO J. 2026 May 26;45(13):4733–65. doi: 10.1038/s44318-026-00814-z (PMC13324165; doi:10.1038/s44318-026-00814-z)
Supplement: Supplementary file 12 — Expanded View Figures [file 44318_2026_814_MOESM12_ESM.pdf]

Expanded View Figures

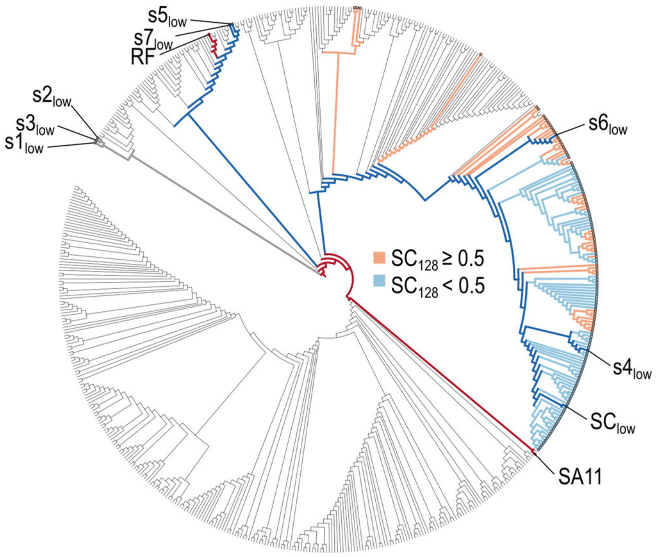

Figure EV1. Phylogenetic analysis of NSP5 variants.

Phylogenetic analysis of 451 unique NSP5 sequences alongside 128 computationally designed permutation variants derived from the SA11-like cluster (SC). Naturally occurring low-scoring variants ($S1_{Low}$-$S7_{Low}$; DeePhase <0.5) are indicated at their respective tips and represent a minority of NSP5 sequences with reduced predicted LLPS propensity that were selected for further analysis. Cell-culture-adapted reference strains SA11 and RF are highlighted in dark red. The SA11-like cluster (SC) consensus and the engineered SC_{Low} variant are marked in dark blue. Among the 128 permutation variants (SC_{128}), tips are coloured orange for sequences with DeePhase scores > 0.5 (high LLPS propensity), and light blue for sequences with scores <0.5 (low-LLPS propensity). Light blue variants therefore denote low-propensity sequences within the SC used to examine how reduced intrinsic LLPS propensity affects condensate formation.

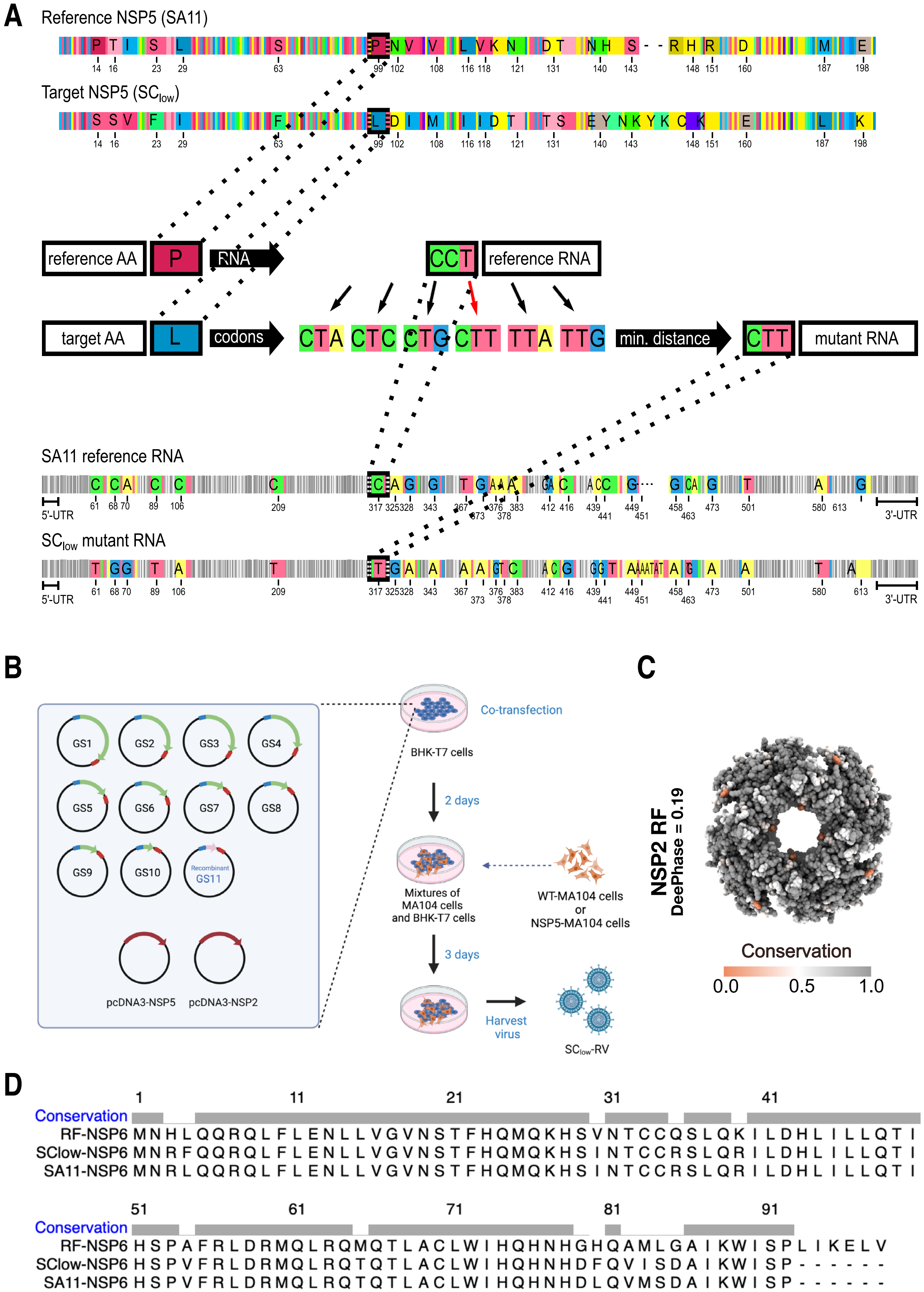
A
Reference NSP5 (SA11)
Target NSP5 (SC$_{low}$)
reference AA
P
RNA
CCT
reference RNA
target AA
L
codons
CTA CTC CTG CTT TTA TTG
min. distance
CTT
mutant RNA
SA11 reference RNA
SC$_{low}$ mutant RNA
5'-UTR
3'-UTR
B
GS1
GS2
GS3
GS4
GS5
GS6
GS7
GS8
GS9
GS10
Recombinant GS11
pcDNA3-NSP5
pcDNA3-NSP2
Co-transfection
BHK-T7 cells
2 days
Mixtures of MA104 cells and BHK-T7 cells
WT-MA104 cells or NSP5-MA104 cells
3 days
Harvest virus
SC$_{low}$-RV
C
NSP2 RF
DeePhase = 0.19
Conservation
0.0
0.5
1.0
D
Conservation
RF-NSP6 MNHLQQRQLFLENLLVGVNSTFHQMQKHSVNTCCQSLQKILDHLILLQTI
SClow-NSP6 MNRFQQRQLFLENLLVGVNSTFHQMQKHSINTCCRSLQRILDHLILLQTI
SA11-NSP6 MNRLQQRQLFLENLLVGVNSTFHQMQKHSINTCCRSLQRILDHLILLQTI
RF-NSP6 HSPAFRLDRMQLRQMQTLACLWIHQHNHGHQAMLGAIKWISPLIKELV
SClow-NSP6 HSPVFRLDRMQLRQTQTLACLWIHQHNHDFQVISDAIKWISP------
SA11-NSP6 HSPVFRLDRMQLRQTQTLACLWIHQHNHDLQVMSDAIKWISP------

Figure EV2. Design, rescue, and genetic stability of the SC$_{Low}$-RV.

(**A**) RNA sequence design for gene segment 11 SC$_{Low}$ using the reference RNA sequence of NSP5 SA11. The coding sequence of NSP5 from SA11 was used as a reference, and synonymous codons were selected to minimise nucleotide divergence while preserving amino acid identity wherever possible. In cases of amino acid substitution (e.g., Proline to Leucine at position 99), the target codon employed required the smallest possible number of nucleotide changes. A total of 35 nucleotide changes were introduced in the SC$_{Low}$ sequence, while untranslated regions (UTRs) were left unchanged. Positions of nucleotide substitutions are indicated. (**B**) Schematic overview of the reverse genetics strategy used to generate SC$_{Low}$-RV. Ten pT7 plasmids encoding the SA11 gene segments (GS1–GS10) and one recombinant plasmid encoding SC$_{Low}$, were co-transfected into BHK-T7 cells along with expression plasmids for NSP2 and NSP5 (pcDNA3-NSP2 and pcDNA3-NSP5). At 48 h post-transfection, either WT-MA104 or NSP5-expressing MA104 (NSP5-MA104) cells were overlaid. Recombinant virus was harvested after three freeze–thaw cycles upon observation of full cytopathic effect (CPE), as described in Methods (Papa et al, 2019). (**C**) Conservation analysis of NSP2 across rotavirus A strains. Full-length NSP2 sequences were aligned, and residue conservation was mapped onto the NSP2 octamer crystal structure (PDB: 1L9V; SA11 strain). Key NSP5-binding regions are conserved across strains (Jiang et al, 2006). (**D**) Multiple sequence alignment of the NSP6 open reading frame encoded by gene segment 11. The NSP6 sequences of SA11, RF, and SC$_{Low}$ sequence were compared, confirming preservation of NSP6 in the engineered SC$_{Low}$-RV genome.

© The Author(s)

A

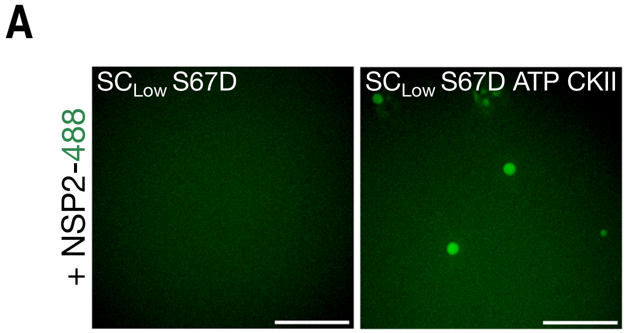

B

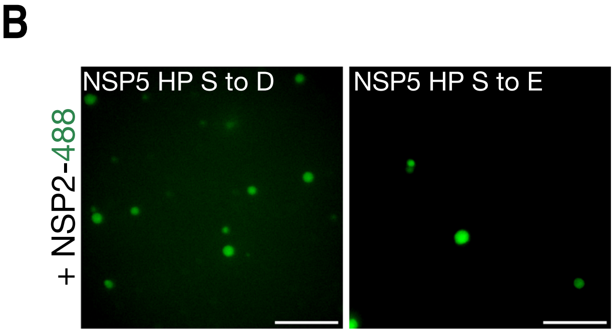

C NSP5 WT phosphorylated

Phosphorylation sites: 4, 8, 9, 22, 24, 25, 26, 30, 42, 63, 65, 72

1 MSLSIDVTSL PSISSSIYKN ESSSTTSTLS GKSIGRSEQY ISPDAEAFSK YMLSKSPEDI GPSDSASNDP LTSFSIRSNA

Phosphorylation sites: 90, 93, 124, 129, 130

81 VKTNADAGVS MDSSTQSRPS SNVGCDQVDF SFNKGIKVNA NLDSSISIST SSKKEKSKSD HKSRKHYPKI EAESDSDDYI

Phosphorylation sites: 164

161 LDDSDSDDGK CKNCKYKRKY FALRMRMKQV AMQLIEDL

D SC_{low} phosphorylated

Phosphorylation sites: 4, 8, 24, 42, 65, 72

1 MSLSIDVTSL PSISSSVYKN ESFSTTSTIS GKSIGRSEQY ISPDAEAFNK YMLSKSPEDI GPFDSASNDP LTSFSIRSNA

Phosphorylation sites: 90, 93, 101, 124, 125, 127

81 VKTNADAGVS MDSSAQSRLS SDIGCDQMDF SLNKGIKIDA TLDSSISIST TSKKEKSKQE YKNKYKKCY PKIEAESDSDE

Phosphorylation sites: 166

161 YVLDDSDSDD GKCKNCKYKK KYFALRLRMK QVAMQLIKDL

E NSP2 WT phosphorylated

1 MAELACFCYP HLESDSYRFI PFNSLAIKCM LTAKVDKKDQ DKFYNSIIYG IAPPPQFKKR YNTNDNSRGM NYETPMFNKV

81 AVLICEALNS IKVTQSDVAS VLSKVISVRH LENLVLRREN HQDVLFHSKE LLLRSVLIAI GHSKEIETTA TAEGGEVVFQ

161 NAAFTMWKLT YLEHRLMPIL DQNFIEYKIT VNEDKPISES HVRELIAELR WQYNKFAVIT HGKGHYRVVK YSSVANHADR

241 VYATFKSNNK NGNVIEFNLL DQRIIWQNWY AFTSSMKQGN TLEICKKLLF QKMKRESNPF KGLSTDRKMD EVSQIGIPLE

321 HHHHHH

P Phosphorylation

© The Author(s)

◀ **Figure EV3. Analysis of phosphorylation of NSP5 variants in vitro.**

(**A**) Comparison of NSP5 HP variants S-to-D *vs* S-to-E. In vitro phase-separation assays of NSP5 in the presence of NSP2. Atto-488-labelled NSP2 (25 µM) was mixed with equimolar amounts of unlabelled NSP5 variants and imaged as described in Methods. Scale bar, 10 µm. (**B**) In vitro phase-separation assays of phosphomimetic S67D (strain SA11) in the presence of NSP2, without CKII + ATP incubation (left) and upon phosphorylation by CKII (right). Scale bar, 10 µm. (**C**–**E**) Sequence coverage maps showing phosphorylation sites of NSP5 variants and NSP2 after in vitro phosphorylation. Sequence coverage is shown for NSP5 RF (**C**) and SC_{Low} (**D**) and NSP2 (**E**). Confidently identified phosphorylation sites are indicated by pink boxes. No phosphorylation sites were confidently identified in the untreated samples.

A

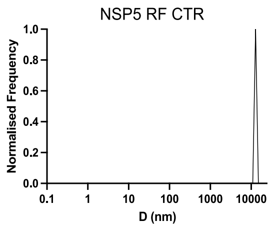

B

	Oligomerisation NSP5 variant only	LLPS NSP5 variant + NSP2
NSP5 IDR CTR	Yes	Yes
* NSP5 HP IDR CTR	Yes	Yes
NSP5 IDR	No	No
* NSP5 HP IDR	No	Yes
CTR	Aggregation	Aggregation
SCLow IDR CTR	No	No
* SCLow HP IDR CTR	Yes	Yes

Figure EV4. The C-terminal region (CTR) of NSP5 alone forms large aggregates.

(**A**) Hydrodynamic diameters (D, in nm) of 1 µM NSP5-RF CTR peptide measured in PBS pH 7.4, as described in Methods, using dynamic light scattering (DLS). (**B**) Summary of the oligomeric states and LLPS capacities of examined NSP5 constructs. Schematic representations of the protein constructs are shown; phosphomimic variants are indicated with a red asterisk.

© The Author(s)

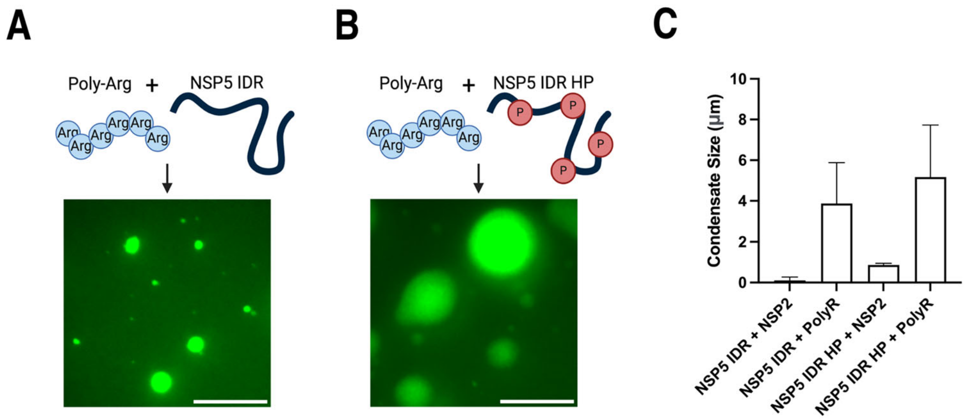

Figure EV5. Both phosphomimetic and non-phosphomimetic NSP5 IDRs undergo phase separation with poly-arginine.

(**A**, **B**). Phase separation of the NSP5 IDR (**A**) and its phosphomimetic variant IDR HP (**B**) in the presence of poly-L-arginine (poly-Arg, average molecular weight ~40 kDa). DyLight-488-labelled IDR or IDR HP (25 µM; schematically shown, with phosphomimetic sites indicated by red 'P') was mixed with poly-Arg (5 µM), and condensates were imaged by wide-field fluorescence microscopy. Scale bar, 10 µm. (**C**) Quantification of condensate area (µm²) formed by NSP5 IDR or IDR HP with poly-L-arginine (PolyR). Nine regions of interest (50 µm × 80 µm each) were analysed as described in "Methods". Error bars represent standard deviation from three independent technical repeats. For comparison, the condensate area formed by NSP2 with NSP5 IDR or IDR HP (Fig. 9) are shown.

© The Author(s)
