# Supplementary material for: Phosphorylation tunes strain-specific protein condensation during rotavirus replication organelle assembly
Source: EMBO J. 2026 May 26;45(13):4733–65. doi: 10.1038/s44318-026-00814-z (PMC13324165; doi:10.1038/s44318-026-00814-z)
Supplement: Supplementary file 2 — Peer Review File [file 44318_2026_814_MOESM2_ESM.pdf]

THE EMBO JOURNAL

Phosphorylation tunes strain-specific protein condensation during rotavirus replication organelle assembly

Julia Acker, Xinyu Wang, Alonso Pardal, Daniel Desirò, Tanushree Agarwal, Alice Colyer, Aidan Tollervey, Rob Scrutton, Cyril Haller, Lee Sherry, Kadi Saar, Ksenia Fominykh, Margaret Johncock, Sai Hou Chong, Rosie Murray, Jamie Terry, Jeremy Schmit, Antonio Calabrese, Tuomas Knowles, and Alexander Borodavka

Corresponding author(s): Alexander Borodavka (alexander.borodavka@path.ox.ac.uk)

Review Timeline:

Submission Date:	15th Jun 25
Editorial Decision:	28th Jul 25
Revision Received:	8th Jan 26
Editorial Decision:	12th Mar 26
Revision Received:	27th Apr 26
Accepted:	7th May 26

Editor: Ieva Gailite

Transaction Report:

(Note: With the exception of the correction of typographical or spelling errors that could be a source of ambiguity, letters and reports are not edited. Depending on transfer agreements, referee reports obtained elsewhere may or may not be included in this compilation. Referee reports are anonymous unless the Referee chooses to sign their reports.)

Dear Alex,

Thank you for submitting your manuscript for consideration by the EMBO Journal. We have now received comments from a full set of reviewers, which are included below for your information.

As you will see, all reviewers are generally positive in their evaluation, and especially reviewers #2 and #3 appreciate the contribution of the study to the research field. However, they also indicate a number of concerns that would need to be addressed before they can support publication here. In particular, reviewer #1 finds that more direct evidence for the relevance of phosphorylation levels for Nsp5 variant phase separation properties would need to be provided. In a similar vein, reviewer #3 requests additional in-depth analysis of the material properties of the investigated Nsp5-eGFP viroplasms, while reviewer #2 asks for an analysis of the condensation properties of natural rotavirus Nsp5 variants. From my side, I find these requests generally reasonable and constructive. Therefore, based on these positive assessments, I invite you to submit a revised manuscript in response to the comments by all reviewers.

I would be happy to discuss the revision in more detail via email or phone/videoconferencing. However, please note that I will be away from the office between July 31 - August 18 and will be able to arrange a direct Zoom call only after my return.

We generally allow three months as standard revision time, which can be extended to six months in the case of major revisions. Should you foresee a problem in meeting this deadline, please let us know in advance to discuss an extension.

As a matter of policy, competing manuscripts published during this period will not negatively impact on our assessment of the conceptual advance presented by your study. However, please contact me as soon as possible upon publication of any related work to discuss the appropriate course of action.

When preparing your letter of response to the referees' comments, please bear in mind that this will form part of the Review Process File and will therefore be available online to the community. For more details on our Transparent Editorial Process, please visit our website: https://www.embopress.org/page/journal/14602075/authorguide#transparentprocess. Please also see the attached instructions for further guidelines on preparation of the revised manuscript.

Please feel free to contact me if you have any further questions regarding the revision. Thank you for the opportunity to consider your work for publication. I look forward to discussing your revision.

With best wishes,

Ieva

Ieva Gailite, PhD
Senior Scientific Editor
The EMBO Journal
Meyerhofstrasse 1
D-69117 Heidelberg
Tel: +4962218891309
i.gailite@embojournal.org

Instructions for preparing your revised manuscript:

Please make sure you upload a letter of response to the referees' comments together with the revised manuscript.

Please also check that the title and abstract of the manuscript are brief, yet explicit, even to non-specialists.

When assembling figures, please refer to our figure preparation guideline in order to ensure proper formatting and readability in print as well as on screen:
https://bit.ly/EMBOPressFigurePreparationGuideline
See also guidelines for figure legends: https://www.embopress.org/page/journal/14602075/authorguide#figureformat

At EMBO Press we ask authors to provide source data for the main manuscript figures. You will receive a separate email with instructions for providing source data with your revised manuscript, including how to upload and organize the files.

IMPORTANT: When you send the revision we will require

- a point-by-point response to the referees' comments, with a detailed description of the changes made (as a word file).
- a word file of the manuscript text.
- individual production quality figure files (one file per figure)
- a complete author checklist, which you can download from our author guidelines (https://www.embopress.org/page/journal/14602075/authorguide).
- Expanded View files (replacing Supplementary Information)
Please see out instructions to authors
https://www.embopress.org/page/journal/14602075/authorguide#expandedview
- a Reagents and Tools Table as part of the Methods section, which can be downloaded from our author guidelines (https://www.embopress.org/page/journal/14602075/authorguide#structuredmethods)

Please remember: Digital image enhancement is acceptable practice, as long as it accurately represents the original data and conforms to community standards. If a figure has been subjected to significant electronic manipulation, this must be noted in the figure legend or in the 'Materials and Methods' section. The editors reserve the right to request original versions of figures and the original images that were used to assemble the figure.

Further information is available in our Guide For Authors: https://www.embopress.org/page/journal/14602075/authorguide

We realize that it is difficult to revise to a specific deadline. In the interest of protecting the conceptual advance provided by the work, we recommend a revision within 3 months (26th Oct 2025). Please discuss the revision progress ahead of this time with the editor if you require more time to complete the revisions. Use the link below to submit your revision:

https://emboj.msubmit.net/cgi-bin/main.plex

Referee #1:

The concern with this manuscript falls on one major point. The authors claim that phase separation/LLPS potential is dependent on phosphorylation status (Figure 5). However, the changes introduced into the low mutant are amino acid changes and not phosphorylation mutants (sequence changes only). Therefore, while the data provide important leads, the claim that phosphorylation influences the potential of LLPS or phase separation of SClow are not supported by the data presented.

Likewise, in Figure 6, the data presented only suggest that sequence determinants, and not phosphorylation-dependent events, that influence the phase separation behaviors.

In Figure 7, the authors again claim shifts in oligomerization based on phosphorylation. I agree that the phospho blots and viral replication kinetics shown in the earlier figures 3 & 4 provide compelling points of discussion, until this point in the manuscript there is no real evidence that phosphorylation determines the condensation/coacervation/LLPS potential.

In the Discussion the authors do highlight "sequence-dependent" phase separation behavior. That claim is justified.

The authors provide quite compelling data with state-of-the-art techniques and approaches. However, the manuscript suffers from direct evidence that phosphorylation events dictate phase separation behavior. Other than the data generated by algorithms, more direct evidence should be provided for publication in EMBO J.

Referee #2:

In this manuscript, Acker et al. investigate how phosphorylation of rotavirus NSP5 modulates NSP5-NSP2 interactions to promote phase-separation. By employing a bioinformatic based approach, they identify NSP5 variants from different rotavirus strains that vary in their predicted propensity for phase-separation. They then used sequence information from these variants to engineer an NSP5 variant with a low propensity for phase-separation (SClow). The authors characterize and compare the molecular biology of SClow and NSP5 from a lab adapted rotavirus strain as a means to explore how the amino acid sequence of NSP5 and its phosphorylation interplay as key determinants of phase-separation and formation of viral factories. They demonstrate that SClow is highly dependent on phosphorylation for phase-separation in vitro, and for viral factory formation, and ultimately for efficient viral replication in infected cells. Employing a range of techniques including in vitro oligomerization, phase-separation assays, and hydrogen-deuterium exchange, they characterize NSP5 interactions, and how these interactions are altered by phosphorylation. The data presented in this manuscript support a model in which phosphorylation of NSP5 alters NSP5 binding modes, and in the case of SClow promotes the formation of NSP5-NSP2 condensates in the context of NSP5 variants with low basal phase-separation propensity. The authors conclude a context-dependent role for NSP5 phosphorylation, and suggest that there is mechanistic diversity in the regulation of viral condensate formation by phosphorylation between viral

strains.

This is a well put together study, that is clearly communicated. A major strength of this study is the in-depth characterization of NSP5 interactions, which combines several different powerful approaches. This combination of approaches provides valuable mechanistic insight into how the modulation of specific protein-protein interactions may alter emergent behavior, insight that is often lacking in other studies. While this study will of course be of relevance to others interested in viral replication, the mechanistic insight gained will likely be of interest to a much wider audience. Understanding how protein-protein interactions translate to differences in emergent behaviors such as condensate formation/properties is an important fundamental question relevant to many cellular processes. For this reason, the manuscript reports an important and valuable study. However, additional context may be required to establish fully wider claims suggested by the authors. Suggested improvements are listed here under major comments.

Major comments:
1. An important part of the takeaway message from this manuscript is that NSP5 variants from different rotavirus strains vary in their phase separation propensity. However, this is only explored as an in silico prediction. The engineered SClow mutant is proposed to represent the generalized behavior of the naturally occurring low LLPS NSP5 variants upon which its engineering was based. The reasoning behind this is sound but requires extrapolation. To remedy this situation, can the authors test the phase-separation of naturally occurring NSP5 variants (or select representative predicted low LLPS variants) to validate the DeePhase predictions? Although some experiments may be difficult to do with certain viral strains, a test of the basic principle should be possible using reductionist in vitro approaches, such as LLPS assays with recombinant protein.
2. The hydrogen-deuterium data presented in Figure 10 is compelling. However, the comparison between NSP5 WT and NSP5 phosphomimic only partially interrogates the proposed model and claims. If a comparison of SClow could be performed (perhaps including phospho-mutants of SClow also) this could significantly strengthen the link between alternate interaction interfaces and alternate emergent behavior as it pertains to the central question of how this biology differs between high and low LLPS variants.
3. The order in which figures are called out in the text is confusing and may not be in line with journal standards. For example, the description of the results seems to flow from Figure 1 to Figure 3 without really addressing the content in Figure 2.

Minor comments:
1. In several figures, condensate "size" is measured and given in the units µm. Would this be better described as condensate diameter? This may be more useful to the reader in terms of gauging the impact of observed differences, as small changes in diameter obviously equate to changes of greater magnitude in terms of condensate volume for example, and the terminology "size" is more ambiguous.
2. The discussion of the HDX results described in figure 10 speak of deprotection. Could the authors explain to the non-expert in HDX how protection and deprotection can be interpreted in terms of interaction interface?

Referee #3:

This manuscript addresses how sequence diversity and phosphorylation can modulate the phase separation properties of the non-structural rotavirus protein NSP5 and its interaction with NSP2. By combining machine learning-based sequence analysis, rational protein engineering, biophysical and cell-based assays, and structural mass spectrometry, the authors demonstrate that. In contrast, many natural NSP5 variants are LLPS-competent, and some identified low-LLPS variants (like SClow) repair their condensation ability through phosphorylation. The phosphorylation acts as an allosteric switch, controlling the function of NSP5 and the physical nature of viroplasm formation-a core process in the replication cycle of rotavirus. The work provides both mechanistic insight and evolutionary context, with implications for viral replication and condensate regulation.

Comments-

1) Please consider adding line numbers to the manuscript. It would be easier to refere to specific line numbers when commenting.
2) Please consider revising the Levenshtein distance definition for accuracy: "insertions, deletions, or substitutions," not just "changes," to align with the standard biological interpretation. Perhaps the sentence "The Levenshtein distance quantifies the minimum number of single-residue changes needed to transform one sequence into another..." can be revised to "...minimum number of single-residue insertions, deletions, or substitutions needed to..."
3) The text mentions that NSP5 is highly sequence-divergent but does not clarify how divergent (e.g., percent identity) or whether this variation maps to known regulatory motifs or regions. A short elaboration would enrich the rationale for the sequence-based LLPS prediction.
4) Was S67D alone sufficient to restore LLPS in earlier reports, or only phosphorylation patterning?
5) Have the authors tried quantifying condensate material properties using FRAP (Fluorescence Recovery After Photobleaching) on NSP5-eGFP viroplasms formed by: Wild-type NSP5, SClow, SClow HP, and S67A mutants? The rationale behind it is to test whether condensates formed under different phosphorylation states vary in material properties (e.g., dynamics, fluidity). This experiment can reveal whether phosphorylation modulates not only formation but also maturation and dynamics of viral condensates.

6) The finding that some NSP5 variants have low in vitro LLPS propensity but still form condensates in vivo should be discussed in the context of possible cellular co-factors or crowding effects, as this bridges the gap between the biophysical and cell biological data. More importantly, concerning the variations in the phosphorylation status of NSP2 and the enzymatic activities of NSP2 during infection
7) In the abstract, "failed to phase separate in vitro yet still supported condensate formation and viral replication in cells" might be misread as contradictory. Consider revising for clarity (e.g., "Despite lacking in vitro phase separation capability, the variant supported condensate formation and replication in a cellular context").
Minor points:
1. Be consistent in figure citation formatting - "Fig 5" should be written as "Fig. 5" or the other way around.
2. "...through a process known as dynamic allostery (Berlow et al., 2018; Ferreon et al., 2013; Hilser and Thompson, 2007; Kern and Zuiderweg, 2003). However, identification of such allosteric events..."- You are missing a period after the closing parenthesis before the new sentence.
3. Try rephrasing- "The onset of viroplasm formation depends on the expression levels of both proteins, and on the multiplicity of infection and its duration..." to "The onset of viroplasm formation depends on both the protein expression levels and the multiplicity and duration of infection..."- avoids repetition of "on the".
4. The phrase "membrane-less cellular compartments" should be more precisely stated as "membrane-less organelles" or "biomolecular condensates", which are the widely accepted scientific terms used in the field.

Overall, this is a significant and well-executed study that will be of broad interest in the fields of condensates and virology. The manuscript is well-written, the figures are appropriately designed and informative.

Dear Ieva,

We thank you and the reviewers for your time and for the careful evaluation of our manuscript titled *Phosphorylation enables allosteric control of a viral condensate* (EMBOJ-2025-121475) and for the constructive suggestions that have substantially improved the work. We have revised the manuscript extensively to:

(i) strengthen the experimental basis for phosphorylation-dependent regulation;
(ii) validate the *in silico* predictions with a naturally occurring low-LLPS NSP5 variant;
(iii) extend the mechanistic interface analysis to the low-propensity background, and
(iv) improve clarity and presentation throughout.

Below we respond point-by-point to each referee comment (*in italics*), where we state our response and what we changed (**in bold**) and where (page/line numbers refer to the revised manuscript), and what the new/updated data show. The changes are also shown in red throughout the attached Word document to help the reviewers to navigate the revised manuscript. Due to additional experimental work (new virus rescue experiments, new production and purification of recombinant proteins, development of new *in vitro* phosphorylation assays suitable for condensate imaging experiments, and new HDX data, we have updated the list of co-authors accordingly.

Referee #1

Comment: The concern with this manuscript falls on one major point. The authors claim that phase separation/LLPS potential is dependent on phosphorylation status (Figure 5). However, the changes introduced into the low mutant are amino acid changes and not phosphorylation mutants (sequence changes only). Therefore, while the data provide important leads, the claim that phosphorylation influences the potential of LLPS or phase separation of SClow are not supported by the data presented.

We agree that, in the original submission, some wording could be read as attributing causality to phosphorylation based largely on phosphomimetic or sequence-engineering comparisons. To address this directly, we added an experiment that changes phosphorylation state without altering sequence: recombinant SC_{Low} phosphorylated by casein kinase II (CKII) known to mediate hyperphosphorylation of NSP5 shows increased condensate formation compared with an ATP-only control without casein kinase II (CKII) (Fig. 5B), and phosphorylation of SC_{Low} was confirmed by LC–MS (Expanded View Fig. EV3). These additions are described in the Results on pp. 14-15, lines 406–433, and they provide the direct biochemical evidence requested (same sequence, different phosphorylation states, different condensation outcome).

Comment: Likewise, in Fig. 6, the data presented only suggest sequence determinants, and not phosphorylation-dependent events, influence the phase separation behaviors.

We clarified the logic and framing so that Fig. 6 is presented as a quantitative mapping of phase behavior across variants and phosphomimetic counterparts, while the conclusion that phosphorylation itself can enable LLPS in low-propensity backgrounds is now anchored to the new CKII phosphorylation experiment (Fig. 5B; p.15). In addition, we explicitly validate the "low basal propensity" class using a naturally occurring low-scoring variant ($S4_{Low}$) and show that it only forms condensates upon introduction of HP substitutions or phosphorylation using CKII (Fig. 5A; p.15), supporting the context-dependent model that sequence sets baseline propensity and phosphorylation can enable nucleation in low-propensity backgrounds.

Comment: In Fig. 7, the authors again claim shifts in oligomerization based on phosphorylation; until this point there is no real evidence that phosphorylation determines condensation/coacervation/LLPS potential.

In the revision, we retained the oligomerisation measurements because they are important mechanistic context, but we adjusted the narrative so that oligomerisation changes are not presented as the sole "evidence" for phosphorylation control. Instead, the direct causal support is now provided by kinase-driven phosphorylation of SC_{Low} (Fig. 5B; p.15, lines 411–433) together with LC–MS confirmation (Expanded View Fig. EV3; p.15, line 411–433), and Fig. 7 is used to interpret how phosphorylation/phosphomimetic charge patterning correlates with altered assembly states that accompany condensation in the low-propensity background. We have changed the figure title (Figure 7. *Hyperphosphorylation enables oligomerisation of low LLPS propensity NSP5 variants*) to more accurately reflect the data.

Comment: I agree that the phospho blots and viral replication kinetics shown in the earlier figures 3 & 4 provide compelling points of discussion, until this point in the manuscript there is no real evidence that phosphorylation determines the condensation/coacervation/LLPS potential.

In response, we strengthened the manuscript's central claim by adding direct phosphorylation rescue (CKII/ATP + LC–MS verification) and by broadening beyond engineered SC_{Low} to test a naturally occurring low-propensity NSP5 variant ($S4_{Low}$). We also refined the language throughout to clearly distinguish phosphomimetic sequence changes from enzymatic phosphorylation, and to present phosphorylation-dependent regulation as context-dependent rather than universal (supported by the abstract framing on p.1, lines 32–35). In addition, we show that the S67A point mutation, which abrogates NSP5 hyperphosphorylation in the SA11 strain yet still permits viral replication, completely abolishes viroplasm formation and viral replication when introduced into the SC_{Low} background (Fig. 8). This finding further supports our conclusion that phosphorylation plays a context-dependent role in condensate formation that depends on the underlying NSP5 sequence and its basal LLPS propensity. We note that the S67A mutant (Papa et al., 2019; J Virol 94:e01110-19) provides the cleanest means of eliminating NSP5 hyperphosphorylation without using casein kinase inhibitors, which are often inefficient and can have off-target effects that complicate interpretation.

Referee #2

In this manuscript, Acker et al. investigate how phosphorylation of rotavirus NSP5 modulates NSP5-NSP2 interactions to promote phase-separation. By employing a bioinformatic based approach, they identify NSP5 variants from different rotavirus strains that vary in their predicted propensity for phase-separation. They then used sequence information from these variants to engineer an NSP5 variant with a low propensity for phase-separation (SClow). The authors characterize and compare the molecular biology of SClow and NSP5 from a lab adapted rotavirus strain as a means to explore how the amino acid sequence of NSP5 and its phosphorylation interplay as key determinants of phase-separation and formation of viral factories. They demonstrate that SClow is highly dependent on phosphorylation for phase-separation in vitro, and for viral factory formation, and ultimately for efficient viral replication in infected cells. Employing a range of techniques including in vitro oligomerization, phase-separation assays, and hydrogen-deuterium exchange, they characterize NSP5 interactions, and how these interactions are altered by phosphorylation. The data presented in this manuscript support a model in which phosphorylation of NSP5 alters NSP5 binding modes, and in the case of SClow promotes the formation of NSP5-NSP2 condensates in the context of NSP5 variants with low basal phase-separation propensity. The authors conclude a context-dependent role for NSP5 phosphorylation, and suggest that there is mechanistic diversity in the regulation of viral condensate formation by phosphorylation between viral strains.

This is a well put together study, that is clearly communicated. A major strength of this study is the in-depth characterization of NSP5 interactions, which combines several different powerful approaches. This combination of approaches provides valuable mechanistic insight into how the modulation of specific protein-protein interactions may alter emergent behavior, insight that is often lacking in other studies. While this study will of course be of relevance to others interested in viral replication, the mechanistic insight gained will likely be of interest to a much wider audience. Understanding how protein-protein interactions translate to differences in emergent behaviors such as condensate formation/properties is an important fundamental question relevant to many cellular processes. For this reason, the manuscript reports an important and valuable study. However, additional context may be required to establish fully wider claims suggested by the authors. Suggested improvements are listed here under major comments.

Major comment 1: *An important part of the takeaway message from this manuscript is that NSP5 variants from different rotavirus strains vary in their phase separation propensity. However, this is only explored as an in silico prediction. The engineered SClow mutant is proposed to represent the generalized behavior of the naturally occurring low LLPS NSP5 variants upon which its engineering was based. The reasoning behind this is sound but requires extrapolation. To remedy this situation, can the authors test the phase-separation of naturally occurring NSP5 variants (or select representative predicted low LLPS variants) to validate the DeePhase predictions? Although some experiments may be difficult to do with certain viral strains, a test of the basic principle should be possible using reductionist in vitro approaches, such as LLPS assays with recombinant protein.*

We thank the reviewer for this comment, and we have added experimental validation using a naturally occurring low-scoring NSP5 variant from the naturally occurring variant with the GenBank ID BBB18699 (designated $S4_{Low}$). We now report that $S4_{Low}$ shows the lowest DeePhase score among the tested RV variants and, critically, that $S4_{Low}$ only forms condensates upon introduction of eight S→D substitutions ($S4_{Low}$ HP) (Fig. 5A), mirroring the engineered SC_{Low} behavior and experimentally validating the prediction (Results p.15, lines 411–433). Moreover, $S4_{Low}$ closely phenocopies SC_{Low} in its oligomeric state, and in our *in vitro* assay fails to form condensates unless phosphorylated by casein kinase (Fig.5).

Major comment 2: The hydrogen-deuterium data presented in Figure 10 is compelling. However, the comparison between NSP5 WT and NSP5 phosphomimic only partially interrogates the proposed model and claims. If a comparison of SClow could be performed (perhaps including phospho-mutants of SClow also) this could significantly strengthen the link between alternate interaction interfaces and alternate emergent behavior as it pertains to the central question of how this biology differs between high and low LLPS variants.

We expanded the HDX–MS analysis and its interpretation to include the low-propensity background (SC_{Low}) and its phosphomimetic counterpart (SC_{Low} HP). In the revised Results, we report that similar to WT NSP5, SC_{Low} exhibits protection within the IDR upon addition of NSP2 (HDX Results p. 28, lines 659–674). These new data are presented in the revised Fig. 10. Notably, despite showing IDR protection (Fig. 10B & C), SC_{Low} does not form condensates under these conditions. In contrast, the condensate-forming SC_{Low} HP displays protection in the CTR region, consistent with a shift toward CTR-mediated NSP2 binding in the hyperphosphorylated context, closely mirroring the WT NSP5–NSP2 interaction mode (Fig. 10E). We thank the Reviewer for this excellent suggestion, which enabled us to refine the mechanistic model presented in Fig. 11.

Major comment 3: The order in which figures are called out is confusing (e.g., Results flow from Fig. 1 to Fig. 3 without addressing Fig. 2).

We thank the Reviewer for pointing this discrepancy, we revised the Results narrative to improve continuity and figure order, ensuring that the computational survey and sequence-divergence rationale (Fig. 2) are introduced and discussed before transitioning to the cell-based and mechanistic sections; the revised text now presents the DeePhase survey and sequence divergence context early (e.g., p.4, lines 144-147), which aligns the narrative with the figure sequence and addresses this issue.

Minor comment 1: Condensate "size" is measured in μm; would diameter be clearer?

We agree and improved terminology so the metric is explicit. Where a linear measure is discussed we now use "condensate diameter," for example in the section discussing effects on condensate growth (p.24, line 583), we quantify condensate area ($μm^2$) rather than an ambiguous "size."

Minor comment 2: Please explain how HDX protection/deprotection relates to interaction interfaces for non-experts.

We added a brief, reader-facing explanation directly in the HDX Results text describing "protected" versus "deprotected" amide hydrogens in the context of solvent exposure and binding/structural stabilization (p.25, lines 609–611), and then link those changes to the interpretation of NSP2-dependent interface usage (p.25, lines 620–626).

Referee #3

This manuscript addresses how sequence diversity and phosphorylation can modulate the phase separation properties of the non-structural rotavirus protein NSP5 and its interaction with NSP2. By combining machine learning-based sequence analysis, rational protein engineering, biophysical and cell-based assays, and structural mass spectrometry, the authors demonstrate that. In contrast, many natural NSP5 variants are LLPS-competent, and some identified low-LLPS variants (like SClow) repair their condensation ability through phosphorylation. The phosphorylation acts as an allosteric switch, controlling the function of NSP5 and the physical nature of viroplasm formation-a core process in the replication cycle of rotavirus. The work provides both mechanistic insight and evolutionary context, with implications for viral replication and condensate regulation.

Comment 1: Please consider adding line numbers to the manuscript. It would be easier to refer to specific line numbers when commenting.

Thank you for pointing it out, the line numbers are now included throughout the revised manuscript.

Comment 2: Please consider revising the Levenshtein distance definition for accuracy: "insertions, deletions, or substitutions," not just "changes," to align with the standard biological interpretation. Perhaps the sentence "The Levenshtein distance quantifies the minimum number of single-residue changes needed to transform one sequence into another..." can be revised to "...minimum number of single-residue insertions, deletions, or substitutions needed to...

We corrected the definition to the standard formulation - single-residue substitutions, insertions, or deletions - at the point where we introduce this analysis (p.4, lines 155–158).

Comment 3: The text mentions NSP5 is highly sequence-divergent but does not clarify how divergent or whether variation maps to known regions; please elaborate (e.g., percent identity).

We added quantitative context and mapping, stating that RVA NSP5 sequences are ~78% identical overall and that variability is concentrated primarily in residues 102–156 (p.4, lines 144–146), strengthening the rationale for using sequence-based LLPS prediction and engineering.

Comment 4: Was S67D alone sufficient to restore LLPS in earlier reports, or only phosphorylation patterning?

Thank you for this comment. We now explicitly address this in the Results: while S67D was previously implicated in restoring hyperphosphorylation, a single S67D substitution does not restore SC_{Low} droplet formation under our assay conditions, whereas enzymatic phosphorylation by CKII does (Results p.15, lines 412–416 and immediately following in the same paragraph, and Discussion p.33, lines 797-801).

Comment 5: Have the authors tried quantifying condensate material properties using FRAP (Fluorescence Recovery After Photobleaching) on NSP5-eGFP viroplasms formed by: Wild-type NSP5, SClow, SClow HP, and S67A mutants? The rationale behind it is to test whether condensates formed under different phosphorylation states vary in material properties (e.g., dynamics, fluidity). This experiment can reveal whether phosphorylation modulates not only formation but also maturation and dynamics of viral condensates.

We agree that condensate material properties are an important dimension; however, we did not include FRAP in this revision because the principal revision need as highlighted particularly by Reviewer #1 was to provide direct causal evidence that phosphorylation can enable condensation in low-propensity variants, which we addressed by adding kinase-driven phosphorylation with LC–MS confirmation and by expanding mechanistic interface mapping (CKII/ATP + LC–MS: p.15; HDX expansion: pp.25-26). In addition, FRAP in infected-cell viroplasms is particularly sensitive to infection stage, ongoing protein synthesis/recruitment, multiplicity of infection, expression variability, and condensate maturation as the infection progresses even within the same cell, as we have previoosly shown for rotavirus viroplasms (Geiger, Acker *et al.*, EMBO J, 2021). Given the observed differences in replication kinetics of these variants, these factors make fluorescence recovery kinetics very difficult to interpret without a substantial additional experimental and analytical framework, while not addressing the key questions of the mechanistic role of phosphorylation in condensate formation. We therefore prioritised experiments that directly resolve the key causality concern while viewing FRAP as a valuable future extension that would be used for *in vitro* reconstituted condensates.

Comment 6: Low in vitro LLPS propensity but condensate formation in vivo should be discussed in the context of cofactors/crowding; also consider NSP2 phosphorylation/enzymatic activities.

We strengthened the context-dependent framing in the Abstract and early Results to reduce perceived contradiction and to emphasize that cellular conditions can support condensate formation even when reductionist in vitro systems reveal low basal propensity; the Abstract now states that the engineered low-propensity variant lacks in vitro phase separation unless phosphorylated yet supports condensate formation and replication in cells, and we now provide direct experimental anchoring using a naturally occurring low-propensity NSP5 variant ($S4_{Low}$; p.15) to support the broader biological point.

We have also considered the Reviewer's suggestion to include NSP2 phosphorylation activities, and we used LC/MS to analyse its phosphorylation status in our in vitro droplet/phosphorylation assays. These results have now been included in the Expanded view Figure EV3).

Comment 7: In the abstract, "failed to phase separate in vitro yet still supported condensate formation and viral replication in cells" might be misread as contradictory. Consider revising for clarity (e.g., "Despite lacking in vitro phase separation capability, the variant supported condensate formation and replication in a cellular context").

We revised the Abstract to explicitly state the conditional nature of the *in vitro* phenotype ("unless phosphorylated"), removing the apparent contradiction while preserving the key conclusion that condensates and replication occur in cells (p.1, lines 30–31).

Minor points (formatting consistency, punctuation, repetition, terminology such as "membrane-less organelles").
We standardised terminology and formatting throughout (including consistent "Fig." formatting), corrected punctuation, and adopted field-standard wording in the Introduction (e.g., "membrane-less organelles"), improving readability and consistency. We thank the Reviewer for their valuable time and help.

Overall, this is a significant and well-executed study that will be of broad interest in the fields of condensates and virology. The manuscript is well-written, the figures are appropriately designed and informative.

We thank the Reviewer for their generous and kind assessment of our work, and we hope that these revisions and new experiments address the reviewers' concerns and strengthen the manuscript's mechanistic conclusions. We thank the referees again for their helpful input and would be pleased to address any further questions.

Sincerely,
Dr Alexander Borodavka on behalf of all authors

1st Revision - Editorial Decision **12th Mar 2026**

Dear Alex,

Thank you for submitting a revised version of your manuscript. I sincerely apologise for the unusually prolonged assessment process due to delays in reviewer report submission. We have now received input from two of the original reviewers, which I have included below.

As you can see, reviewer #2 is now satisfied with the revisions, while reviewer #1 requests further clarifications for general readability and clarity that should be incorporated in the final version. There are also a few editorial and formatting points that need to be addressed before I can extend official acceptance of the manuscript:

1. Please note that it is The EMBO Journal policy for the transcript of the editorial process (containing referee reports and your response letters) to be published as an online supplement to each paper. If you should prefer removal of any referee-only figures included in the point-by-point response(s), e.g. because they may still be used for future publication or because they have been reproduced from published work by others, please do let us know immediately via response email.
More information is available here: https://www.embopress.org/transparent-process#Review_Process
2. Please check the correct name for the one of the authors: Antonio N. Calabrese is mentioned in the manuscript, while Anton Calabrese is listed in our online system.
3. Please include the corresponding author's email on the title page of the manuscript.
4. The manuscript sections should be in the following order: Title page - Abstract - Introduction - Results - Discussion - Methods - Data Availability - Acknowledgments - Disclosure and Competing Interests Statement - References - Figure Legends - (Main Tables with legends if applicable) - Expanded View Figure Legends.
5. Please remove figures from the manuscript text file, while leaving their legends in, after References.
6. CRediT has replaced the traditional author contributions section because it offers a systematic, machine-readable author contributions format that allows for more effective research assessment. Please remove the Authors Contributions from the manuscript and use the free text boxes beneath each contributing author's name in our online submission system to add specific details on the author's contribution. More information is available in our guide to authors.
7. Please include the "Funding" section in "Acknowledgements".
8. Please check that the funding information is correct and identical both in the manuscript and our online system. Currently, EPSRC SBS DTP [2597129], Funding from the BBSRC (BB/M012573/1) and Wellcome (208385/Z/17/Z), Royal Society research grant (RGS\R2\222357) are not listed in our online system, while RG\R1\241230 is not included in the manuscript text.
9. Please rename "Conflict of interest" section into "Disclosure and competing interests statement".
10. Please update references according to The EMBO Journal style - where there are more than 10 authors on a paper, the first 10 should be listed, followed by 'et al.' DOIs should only be used for preprints and datasets that have not been published yet.
11. The following figure panels are not mentioned in the manuscript text: Figure 5C, Figure 8C, Figure 9A, Figure 10A/D. Please add the corresponding callouts.
12. There is a reference to Table 1 in the manuscript text, please update to Appendix Table S1.
13. In the Appendix, please add a title page indicating "Appendix for "manuscript title"" and a brief table of contents including page numbers. Please update the nomenclature to Appendix Figure S1.
14. In our standard image check, we noticed potential image reuse between the following panels:
- Figure 1 and Figure 5A - if this was intentional, please indicate this in the figure legends.
- Within Figure 9A, panels 50% NSP5 CTR and 75% NSP5 CTR - please check. These images show almost identical background signal patterns.
15. During our standard source data check, I noticed some repetitive values in the source data for Figure 4D. I have attached the corresponding file with the detected duplications labelled in colour. Please take a look and correct if needed. A brief explanation would be very helpful.
16. Our data editors have flagged the following issues in figure legends that need correcting:
- Please provide the exact p values in the legends of figures 5C, 9B.
- Please define the box plots in terms of minima, maxima, centre, bounds of box and whiskers, and percentile in the legends of figures 2A, 5C.
- Please provide information on the number and nature of replicates in the legends of figures 2A, 3A, EV5 C.
- Please define the error bars in the legends of figures 9B, EV5 C".
- Please define the scale bar for figures 3D, 5A, EV3 A, B.
17. Papers published in The EMBO Journal are accompanied online by a 'Synopsis' to enhance discoverability of the manuscript. It consists of A) a short (1-2 sentences) summary of the findings and their significance, B) 3-4 bullet points highlighting key results and C) a synopsis image that is 550x300-600 pixels large (width x height, jpeg or png format). You can either show a model or key data in the synopsis image. Please note that the image size is rather small and that text needs to be readable at the final size. Please send us this information together with the revised manuscript.

Please let me know if you have any questions regarding any of these points. You can use the link below to upload the revised files.

Thank you again for giving us the chance to consider your manuscript for The EMBO Journal. I look forward to receiving the final

version.

With best wishes,

Ieva

Ieva Gailite, PhD
Senior Scientific Editor
The EMBO Journal
Meyerhofstrasse 1
D-69117 Heidelberg
Tel: +4962218891309
i.gailite@embojournal.org

We realize that it is difficult to revise to a specific deadline. In the interest of protecting the conceptual advance provided by the work, we recommend a revision within 3 months (10th Jun 2026). Please discuss the revision progress ahead of this time with the editor if you require more time to complete the revisions. Use the link below to submit your revision:

https://emboj.msubmit.net/cgi-bin/main.plex

Referee #1:

Acker et al. Have submitted their revision of the manuscript, "Phosphorylation enables allosteric control of a viral condensate" with accompanying responses to rviewers. The main comment from this reviewer indicated that there was no real direct correlation between phosphorylation and condensate formation. The reviewer also found the original manuscript complex, especially when examining the propensities of NSP5 from various strains of rotavirus to engineer phase separated condensates. The authors have added several new studies to support their claim that phosphor NSP5 promotes phase separation that relies on strain-specific phoshorylations events. These assays include some phylogeny, calculated propensities to phase phase separate by DeePhase, infectivity (PFU) scores, creation of mutant NSP proteins and their respective ability to cis regulate phosphorylation, imaging and western blotting, in vitro phosphorylation assays, PhaseScan blots, some preliminary Alphafold 2.0 structures, some relevant replication assays and mutational studies to show viroplasm formation, and finally, HDX studies to examine interactions that mediate phase separation.

To address the complex nature of the article, the authors have revised the manuscript. It still suffers, in this reviewer's opinion, from too complex writing without a clear and direct set of experiments. There are somewhat too many acronyms to be able to easily follow the progression of the manuscript. The authors should simplify this train of experimental thought to allow readers to gain a clearer meaning of the work.

The added experimental details do indeed add to the story but this reviewer is still somewhat confused about the nature of various Rotavirus strains that do not seem to harbor any conservation of NSP function. Is it just in just in the blue color-coded variants (Figure EV1) that we should be concerned about? Why? Is there a precedent to this type of difference between variants in other virus clades?

Despite some changes to phospho status, there still seems to be viroplasm assembly (re: Figure 4). The viroplasms are smaller and maybe more numerous. Are these functional? The authors note on page 30: ("Low-propensity NSP5 variants show an incomplete switch from CTR- to IDR-mediated interactions upon phosphorylation, potentially explaining the smaller condensate phenotype."). This statement may not completely explain this perplexing phenotype. The histograms' color coding in Figure 4 should also be defined in the body of the figure.

S67D failed to phase separate but his could be overcome with phosphorylation by CKII? Re: Figure EV3B. This reviewer does not see how this can be. Please elaborate. Noted again on page 32 by the authors but again this phenomenon was not explained entirely.

On page 25, the authors state "These results indicate distinct mechanisms of condensation for phosphorylated versus unphosphorylated NSP5 in the presence of NSP2." This type of statement confuses this reviewer. Have the authors determined if this phenomenon is due to cation-pi interactions as stated in the earlier sentence. Is that the only conclusion form the experiment using Arg peptides (Figure EV5)?

Referee #2:

The authors have done an admirable job addressing the comments from the previous review cycle. They have added experimental and in silico support for their conclusions. The revisions have improved the clarity and presentation of data. The manuscript makes a valuable contribution to the literature.

Dear Dr. Gailite,

Thank you for the opportunity to revise our manuscript entitled **"Phosphorylation enables allosteric control of a viral condensate."** We are grateful to you and the reviewers for the constructive and insightful feedback, which has helped us substantially improve the clarity and presentation of our work.

In this revision, we have carefully addressed all comments raised by the reviewers. In particular, we have:

- Reorganised the manuscript to provide a clearer and more direct experimental narrative, including explicit signposting of the key questions addressed in each Results section.
- Clarified the rationale for selecting NSP5 variants and their relevance to the study.
- Expanded the interpretation of viroplasm phenotypes to explicitly address their functional significance.
- Resolved the apparent discrepancy between S67D and CKII-mediated phosphorylation by introducing a threshold-based model of multi-site phosphorylation.
- Refined the mechanistic interpretation of the poly-arginine experiments to avoid overstatement and better align conclusions with the data.
- Simplified the Discussion and aligned the proposed model more closely with Figure 11 to improve accessibility.

These changes are highlighted in red throughout the revised manuscript.

We believe these revisions fully address the reviewers' concerns and significantly strengthen the manuscript. We are encouraged by the positive assessment from Referee #2 and hope that the revised version is now suitable for publication in *The EMBO Journal*.

Below we provide a detailed point-by-point response to the editorial comments and reviewer feedback. All changes in the manuscript are indicated in the revised version.

We thank you again for your consideration and would be happy to provide any further information if required.

With best wishes,

Dr Alex Borodavka, on behalf of all authors.

Referee #1

Response to Referee #1

We thank the reviewer for their careful and thoughtful assessment of our revised manuscript and for recognising the additional experimental work performed. We have taken the reviewer's comments seriously and have made substantial revisions to improve the clarity, structure, and interpretation of the manuscript. Below we address each of the reviewer's concerns in detail.

Reviewer comment:
The manuscript remains too complex and lacks a clear and direct set of experiments; too many acronyms make it difficult to follow.

Response:

We agree that improving clarity was essential. To address this:

1. We have restructured the Introduction to explicitly define the experimental logic and central questions guiding the study (lines 144-154).
2. We have reorganised the Results into a linear progression: sequence analysis - engineering of SC_{Low} - cellular phenotype - *in vitro* phase behaviour - mechanistic dissection.
3. We have reduced acronym usage and simplified terminology, ensuring that key terms are defined once and used consistently.

Together, these changes provide a clearer and more direct narrative linking the experiments to the central hypothesis. We hope these changes further improve the clarity of the narrative, and these changes are highlighted in red throughout the text.

2. Confusion regarding NSP5 variants and strain selection

Reviewer comment:
Unclear why specific variants (e.g., blue variants in Fig. EV1) are studied and whether they are representative.

Response:
We have clarified this point both in the main text and in the figure legend:

1. We now explicitly state that low-LLPS variants represent a minority of NSP5 sequences and serve as informative outliers that allow us to test how reduced intrinsic phase separation propensity affects condensate formation (lines 170-172).
2. We explain that these variants were specifically selected to test whether phosphorylation can compensate for diminished LLPS potential, providing a mechanistic contrast to high-LLPS variants (lines 172-173).
3. The Figure EV1 legend has been revised to clearly define the colour coding and to indicate which variants were selected for further study and why.

4. We have also added context noting that variability in intrinsically disordered regions is a common feature of viral proteins, supporting the biological relevance of studying such divergence (Lines 195-202).

These changes clarify that the selected variants are not arbitrary, but central to the experimental design.

3. Functionality of altered viroplasms (Figure 4).

Reviewer comment:
Smaller/more numerous viroplasms raise questions about functionality.

Response:
We have addressed this directly in the Results:

1. We now explicitly state that viral replication proceeds efficiently despite altered condensate morphology, indicating that these structures are functional (Lines 272-274).
2. We clarify that the phenotype reflects delayed viroplasm maturation rather than loss of function, supported by replication kinetics, plaque assays, and imaging data (Lines 272-274).

Additionally, we have clarified the histogram colour coding in Figure 4 within both the legend and figure annotations.

4. S67D vs CKII phosphorylation paradox

Reviewer comment:
Unclear why S67D fails to restore phase separation while CKII phosphorylation does.

Response:
We have clarified this point both in the Results and Discussion:

1. We now explicitly state that S67D represents a single phosphomimetic substitution, whereas CKII introduces multi-site phosphorylation across the IDR (Lines 310-313). We interpret this as evidence for a threshold-like mechanism, where multiple phosphorylation events are required to enable phase separation.
2. This interpretation is supported by analogy to well-characterised systems (e.g., Sic1, ETS-1) and is now clearly integrated into the Discussion (Lines 529-539).

This revision resolves the apparent contradiction and strengthens the mechanistic interpretation.

5. Interpretation of poly-arginine experiments and interaction mechanism

Reviewer comment:
The statement regarding “distinct mechanisms” and potential cation–π interactions is unclear.

Response:
We agree that the original wording may have overinterpreted these results. We have therefore:

1. Revised the text to avoid overstatement, clarifying that the poly-arginine experiments demonstrate that positively charged polymers can promote NSP5 IDR condensation, but do not define the precise interaction mechanism (Lines 411-422). We also replaced definitive language with more appropriate phrasing (e.g., "support a model", "suggest"), ensuring that conclusions are aligned with the data.
2. We also clarified that the key finding is NSP2 selectivity for the phosphorylated state, rather than the identification of a specific interaction type.

6. Mechanistic model and overall interpretation

In response to the reviewer's concerns about complexity, we have substantially revised the Discussion:

1. The proposed model is now presented in two clearly defined steps (unphosphorylated vs phosphorylated states), directly aligned with Figure 11.
2. Redundant explanations have been removed, and the model is now more concise and easier to follow.
3. We explicitly connect the molecular mechanism to functional consequences in the viral life cycle, improving biological relevance in the Discussion section (lines 553-589).

We believe these revisions address all of the reviewer's concerns improve the clarity and impact of the manuscript.

We thank the reviewer again for their constructive feedback, which has significantly strengthened the work.

Response to Referee #2

We thank the reviewer for their positive and encouraging assessment of our revised manuscript. We are pleased that the additional experimental and in silico analyses have improved the clarity and impact of the study.

We hope that these revisions address the concerns raised by the reviewers and improve the clarity and presentation of the manuscript. We thank the editor and reviewers again for their helpful comments and consideration of our work.

With best wishes,

Dr Alexander Borodavka

2nd Revision - Editorial Decision 7th May 2026

Dear Alex,

Thank you for submitting the final version of your manuscript with the editorial requests addressed. I am now pleased to inform you that your manuscript has been accepted for publication in the EMBO Journal. Congratulations with a nice study!

Please note that it is The EMBO Journal policy for the transcript of the editorial process (containing referee reports and your response letters) to be published as an online supplement to each paper. If you should prefer removal of any referee-only figures included in the point-by-point response(s), e.g. because they may still be used for future publication or because they have been reproduced from published work by others, please do let us know immediately via response email.
More information is available here: https://link.springer.com/partners/embo-press/editorial-policies#Peer%20review

Your manuscript will be processed for publication by EMBO Press. It will be copy edited and you will receive page proofs prior to publication. Please note that you will be contacted by Springer Nature Author Services to complete licensing and payment information.

You may qualify for financial assistance for your publication charges - either via a Springer Nature fully open access agreement or an EMBO initiative. Check your eligibility: https://link.springer.com/journal/44318/how-to-publish-with-us

Should you be planning a Press Release on your article, please get in contact with embo_production@springernature.com as early as possible in order to coordinate publication and release dates.

If you have any questions, please do not hesitate to contact the Editorial Office. Thank you for your contribution to The EMBO Journal., and I look forward to seeing your manuscript online!

Best wishes,

Ieva

Ieva Gailite, PhD
Senior Scientific Editor
The EMBO Journal
Meyerhofstrasse 1
D-69117 Heidelberg
i.gailite@embojournal.org

** Click here to be directed to your login page: https://emboj.msubmit.net

EMBO Press Author Checklist

Corresponding Author Name: Alexander Borodavka	
Journal Submitted to: The EMBO journal	
Manuscript Number: 2025-121475	

USEFUL LINKS FOR COMPLETING THIS FORM
The EMBO Journal - Author Guidelines
EMBO Reports - Author Guidelines
Molecular Systems Biology - Author Guidelines
EMBO Molecular Medicine - Author Guidelines

Reporting Checklist for Life Science Articles (updated January
This checklist is adapted from Materials Design Analysis Reporting (MDAR) Checklist for Authors. MDAR establishes a minimum set of requirements in transparent reporting in the life sciences (see Statement of Task: 10.31222/osf.io/9sm4x). Please follow the journal's guidelines in preparing your
Please note that a copy of this checklist will be published alongside your article.

Abridged guidelines for figures
1. Data
The data shown in figures should satisfy the following conditions:
- the data were obtained and processed according to the field's best practice and are presented to reflect the results of the experiments in an accurate and unbiased manner.
- ideally, figure panels should include only measurements that are directly comparable to each other and obtained with the same assay.
- plots include clearly labeled error bars for independent experiments and sample sizes. Unless justified, error bars should not be shown for technical
- if n<5, the individual data points from each experiment should be plotted. Any statistical test employed should be justified.
- Source Data should be included to report the data underlying figures according to the guidelines set out in the authorship guidelines on Data

2. Captions
Each figure caption should contain the following information, for each panel where they are relevant:
- a specification of the experimental system investigated (eg cell line, species name).
- the assay(s) and method(s) used to carry out the reported observations and measurements.
- an explicit mention of the biological and chemical entity(ies) that are being measured.
- an explicit mention of the biological and chemical entity(ies) that are altered/varied/perturbed in a controlled manner.
- the exact sample size (n) for each experimental group/condition, given as a number, not a range;
- a description of the sample collection allowing the reader to understand whether the samples represent technical or biological replicates (including how many animals, litters, cultures, etc.).
- a statement of how many times the experiment shown was independently replicated in the laboratory.
- definitions of statistical methods and measures:
  - common tests, such as t-test (please specify whether paired vs. unpaired), simple χ2 tests, Wilcoxon and Mann-Whitney tests, can be unambiguously identified by name only, but more complex techniques should be described in the methods section;
  - are tests one-sided or two-sided?
  - are there adjustments for multiple comparisons?
  - exact statistical test results, e.g., P values = x but not P values < x;
  - definition of 'center values' as median or average;
  - definition of error bars as s.d. or s.e.m.

Please complete ALL of the questions below.
Select "Not Applicable" only when the requested information is not relevant for your study.

Materials

Newly Created Materials	Information included in the manuscript?	In which section is the information available? (Reagents and Tools Table, Materials and Methods, Figures, Data Availability Section)
New materials and reagents need to be available; do any restrictions apply?	Not Applicable	

Antibodies	Information included in the manuscript?	In which section is the information available? (Reagents and Tools Table, Materials and Methods, Figures, Data Availability Section)
For **antibodies** provide the following information:<br>- Commercial antibodies: RRID (if possible) or supplier name, catalogue number and or/clone number<br>- Non-commercial: RRID or citation	Yes	Reagents and Tools Table, Materials and Methods

DNA and RNA sequences	Information included in the manuscript?	In which section is the information available? (Reagents and Tools Table, Materials and Methods, Figures, Data Availability Section)
**Short novel DNA or RNA including primers, probes**: provide the sequences.	Yes	Reagents and Tools Table, Materials and Methods

Cell materials	Information included in the manuscript?	In which section is the information available? (Reagents and Tools Table, Materials and Methods, Figures, Data Availability Section)
**Cell lines:** Provide species information, strain. Provide accession number in repository **OR** supplier name, catalog number, clone number, and/**OR** RRID.	Yes	Reagents and Tools Table, Materials and Methods
**Primary cultures:** Provide species, strain, sex of origin, genetic modification status.	Not Applicable	
Report if the cell lines were recently **authenticated** (e.g., by STR profiling) and tested for mycoplasma contamination.	Yes	MA104 cell line was purchased from a trusted provider (ATCC) and routinely tested for Mycoplasma contamination

Experimental animals	Information included in the manuscript?	In which section is the information available? (Reagents and Tools Table, Materials and Methods, Figures, Data Availability Section)
**Laboratory animals or Model organisms:** Provide species, strain, sex, age, genetic modification status. Provide accession number in repository **OR** supplier name, catalog number, clone number, **OR** RRID.	Not Applicable	
**Animal observed in or captured from the field:** Provide species, sex, and age where possible.	Not Applicable	
Please detail **housing and husbandry conditions**.	Not Applicable	

Plants and microbes	Information included in the manuscript?	In which section is the information available? (Reagents and Tools Table, Materials and Methods, Figures, Data Availability Section)
**Plants:** provide species and strain, ecotype and cultivar where relevant, unique accession number if available, and source (including location for collected wild specimens).	Not Applicable	
**Microbes:** provide species and strain, unique accession number if available, and source.	Yes	Reagents and Tools Table, Materials and Methods

Human research participants	Information included in the manuscript?	In which section is the information available? (Reagents and Tools Table, Materials and Methods, Figures, Data Availability Section)
If collected and within the bounds of privacy constraints report on age, sex and gender or ethnicity for all study participants.	Not Applicable	

Core facilities	Information included in the manuscript?	In which section is the information available? (Reagents and Tools Table, Materials and Methods, Figures, Data Availability Section)
If your work benefited from core facilities, was their service mentioned in the acknowledgments section?	Yes	Acknowledgements

Design

Study protocol	Information included in the manuscript?	In which section is the information available? (Reagents and Tools Table, Materials and Methods, Figures, Data Availability Section)
If study protocol has been **pre-registered, provide DOI in the manuscript**. For clinical trials, provide the trial registration number **OR** cite DOI.	Not Applicable	
Report the **clinical trial registration number** (at ClinicalTrials.gov or equivalent), where applicable.	Not Applicable	

Laboratory protocol	Information included in the manuscript?	In which section is the information available? (Reagents and Tools Table, Materials and Methods, Figures, Data Availability Section)
Provide DOI OR other citation details if **external detailed step-by-step protocols** are available.	Yes	Reagents and Tools Table, Materials and Methods

Experimental study design and statistics	Information included in the manuscript?	In which section is the information available? (Reagents and Tools Table, Materials and Methods, Figures, Data Availability Section)
Include a statement about **sample size** estimate even if no statistical methods were used.	Yes	Materials and Methods and Figure legends
Were any steps taken to minimize the effects of subjective bias when allocating animals/samples to treatment (e.g. **randomization procedure**)? If yes, have they been described?	Not Applicable	
Include a statement about **blinding** even if no blinding was done.	Not Applicable	
Describe **inclusion/exclusion criteria** if samples or animals were excluded from the analysis. Were the criteria pre-established?<br><br>If sample or data points were omitted from analysis, report if this was due to attrition or intentional exclusion and provide justification.	Not Applicable	
For every figure, are **statistical tests** justified as appropriate? Do the data meet the assumptions of the tests (e.g., normal distribution)? Describe any methods used to assess it. Is there an estimate of variation within each group of data? Is the variance similar between the groups that are being statistically compared?	Yes	Figure legends and Materials and Methods

Sample definition and in-laboratory replication	Information included in the manuscript?	In which section is the information available? (Reagents and Tools Table, Materials and Methods, Figures, Data Availability Section)
In the figure legends: state number of times the experiment was **replicated** in laboratory.	Yes	Figure legends, Materials and Methods
In the figure legends: define whether data describe **technical or biological replicates**.	Yes	Figure legends

Ethics

Ethics	Information included in the manuscript?	In which section is the information available? (Reagents and Tools Table, Materials and Methods, Figures, Data Availability Section)
Studies involving **human participants**: State details of **authority granting ethics approval** (IRB or equivalent committee(s), provide reference number for approval.	Not Applicable	
Studies involving **human participants**: Include a statement confirming that **informed consent** was obtained from all subjects and that the experiments conformed to the principles set out in the WMA Declaration of Helsinki and the Department of Health and Human Services Belmont Report.	Not Applicable	
Studies involving **human participants:** For publication of **patient photos**, include a statement confirming that consent to publish was obtained.	Not Applicable	
Studies involving experimental **animals**: State details of **authority granting ethics approval** (IRB or equivalent committee(s), provide reference number for approval. Include a statement of compliance with ethical regulations.	Not Applicable	
Studies involving **specimen and field samples:** State if relevant **permits** obtained, provide details of authority approving study; if none were required, explain why.	Not Applicable	

Dual Use Research of Concern (DURC)	Information included in the manuscript?	In which section is the information available? (Reagents and Tools Table, Materials and Methods, Figures, Data Availability Section)
Could your study fall under dual use research restrictions? Please check biosecurity documents and list of **select agents and toxins** (CDC): https://www.selectagents.gov/sat/list.htm	Not Applicable	
If you used a select agent, is the security level of the lab appropriate and reported in the manuscript?	Not Applicable	
If a study is subject to dual use research of concern regulations, is the name of the **authority granting approval and reference number** for the regulatory approval provided in the manuscript?	Not Applicable	

Reporting

The MDAR framework recommends adoption of discipline-specific guidelines, established and endorsed through community initiatives. Journals have their own policy about requiring specific guidelines and recommendations to complement MDAR.

Adherence to community standards	Information included in the manuscript?	In which section is the information available? (Reagents and Tools Table, Materials and Methods, Figures, Data Availability Section)
State if relevant guidelines or checklists (e.g., **ICMJE, MIBBI, ARRIVE, PRISMA**) have been followed or provided.	Not Applicable	
For **tumor marker prognostic studies,** we recommend that you follow the **REMARK** reporting guidelines (see link list at top right). See author guidelines, under 'Reporting Guidelines'. Please confirm you have followed these guidelines.	Not Applicable	
For **phase II and III randomized controlled trials**, please refer to the **CONSORT** flow diagram (see link list at top right) and submit the CONSORT checklist (see link list at top right) with your submission. See author guidelines, under 'Reporting Guidelines'. Please confirm you have submitted this list.	Not Applicable	

Data Availability

Data availability	Information included in the manuscript?	In which section is the information available? (Reagents and Tools Table, Materials and Methods, Figures, Data Availability Section)
Have **primary datasets** been deposited according to the journal's guidelines (see 'Data Deposition' section) and the respective accession numbers provided in the Data Availability Section?	Yes	Data availability section, accession numbers and URLs are provided
Were **human clinical and genomic datasets** deposited in a public access-controlled repository in accordance to ethical obligations to the patients and to the applicable consent agreement?	Not Applicable	
Are **computational models** that are central and integral to a study available without restrictions in a machine-readable form? Were the relevant accession numbers or links provided?	Not Applicable	
If publicly available data were reused, provide the respective **data citations in the reference list**.	Not Applicable	
